# *Cis*‐Chelating Diphosphanes for Intracavity Nickel(II)‐Catalyzed Ethylene Oligomerization

**DOI:** 10.1002/chem.202501188

**Published:** 2025-05-24

**Authors:** Yang Li, Sara Figueirêdo de Alcântara Morais, Mingyang Han, Tuan‐Anh Phan, Geordie Creste, Matthieu Jouffroy, Dominique Matt, Jean‐Pierre Djukic, Yann Cornaton, Pierre Braunstein, Katrin Pelzer, Dominique Armspach

**Affiliations:** ^1^ Équipe Confinement Moléculaire et Catalyse Institut de Chimie de Strasbourg UMR 7177 CNRS Université de Strasbourg 4, rue Blaise Pascal, CS90032 67081 Strasbourg cedex France; ^2^ Laboratoire de Chimie et Systémique Organo‐Métalliques Institut de Chimie de Strasbourg UMR 7177 CNRS Université de Strasbourg 4, rue Blaise Pascal, CS90032 67081 Strasbourg cedex France; ^3^ Laboratoire de Chimie Inorganique Moléculaire et Catalyse Institut de Chimie de Strasbourg UMR 7177 CNRS Université de Strasbourg 4, rue Blaise Pascal, CS90032 67081 Strasbourg cedex France

**Keywords:** cyclodextrin, ethylene oligomerization, nickel, palladium, phosphane

## Abstract

Four *cis*‐chelating diphosphanes derived from cyclodextrins (CDs), each featuring a distinct intracavity environment, compel Ni^II^ or Pd^II^ metal centers to reside within *α*‐ or *β*‐CD cavities. Nickel(II) complexes of these metal‐confining ligands act as active catalysts in ethylene oligomerization upon activation with modified methylaluminoxane (MMAO). The size of the cavity and the position of the P_2_Ni fragment relative to the cavity affect both the activity and selectivity of the reaction. In all instances, 1‐butene is the major product (up to 98% C4 products and 90% 1‐butene within the C_4_ fraction). Extensive theoretical studies with state‐of‐the‐art methods carried out on the most selective system suggest that the CD cavity restricts isomerization pathways by limiting the mobility of the coordinated olefin in this constrained supramolecular environment, thereby enhancing *α*‐olefin formation.

## Graphical abstract

Four cavity‐shaped diphosphanes have been synthesized from both *α*‐ and *β*‐cyclodextrins. The metal‐confining properties of these ligands were exploited in nickel‐catalyzed ethylene oligomerization.

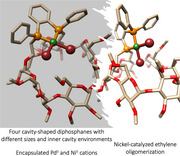

## 1 Introduction

Performing metal‐catalyzed reactions in confined spaces, whether in solids^[^ ^1^ ^]^ or in molecular systems^[^ ^2^ ^]^ is increasingly attractive because of the possibility of achieving unusual and high selectivities with minimal catalyst deactivation.^[^ ^3^ ^]^ It is widely accepted that confining a catalytic center within a restricted or enclosed space can provide more effective steric control over a catalytic reaction than simply grafting flexible, bulky entities onto the main ligand(s). However, substrate accessibility to the catalytically active site remains a prerequisite for catalytic activity. Nickel‐catalyzed ethylene oligomerization/polymerization is a perfect example of an industrial reaction in which steric hindrance around the catalytic center can significantly influence catalyst properties (chain length selectivity, double bond regioselectivity, branching, activity, and stability).^[^ ^1^, ^4^ ^]^ In particular, introducing steric bulk in the axial positions of nickel complexes incorporating dimines^[^ ^5^ ^]^ and other chelating ligands^[^ ^6^ ^]^ disfavors chain transfer and termination reactions^[^ ^7^ ^]^ and strongly influences chain walking mechanisms, which are responsible for branching and alkene isomerization reactions.^[^ ^8^ ^]^ Steric bulk can be positioned above and/or below the metal chelate plane, typically using semi‐rigid substituents containing aromatic units. To date, only one example of a macrocyclic ligand has been reported to achieve this feature, but in none of the corresponding complexes is the metal center fully encapsulated.^[^ ^4^, ^9^ ^]^ Although diphosphane ligands equipped with bulky substituents are not as commonly used for ethylene oligomerization/polymerization^[^ ^10^ ^]^ as the ubiquitous diimines, they have recently been shown to exhibit similar catalytic behavior in the case of their palladium complexes.^[^ ^11^ ^]^

Cavity‐shaped ligands based on cyclodextrins (CDs) displaying metal‐confining ability have demonstrated great potential in highly selective catalytic processes; however, their capacity to catalyze selective ethylene oligomerization/polymerization remains largely unexplored.^[^ ^12^ ^]^ Very recently, a *cis*‐chelating P^N ligand derived from a permethylated *α*‐CD was found to induce very high selectivity in nickel‐catalyzed ethylene dimerization yielding up to 91% 1‐butene.^[^ ^13^ ^]^ This remarkable result motivated us to explore other CD‐based metal‐confining bidentate ligands for this reaction.


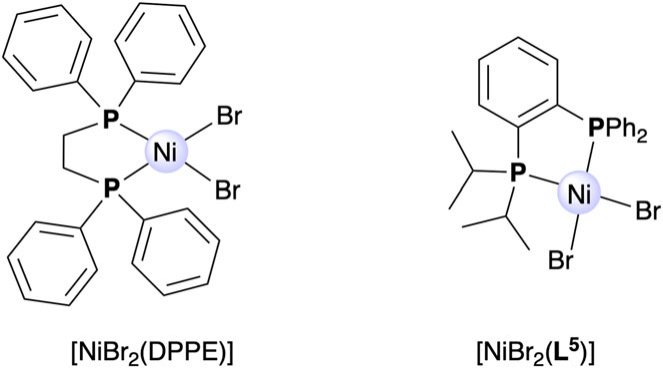


We report here the use of four cavity‐shaped *cis*‐chelating diphosphanes (**L^1^**‐**L^4^**), each with a unique inner space capable of encapsulating via chelation catalytically relevant Ni^II^ and Pd^II^ metal centers. Some of the resulting complexes ([NiBr_2_(**L^1^**‐**L^3^**)], [NiCl_2_(**L^4^**)], **6a,b** and **7a,b**), along with cavity‐free analogs [NiBr₂(DPPE)] (DPPE = 1,2‐bis(diphenylphosphano)ethane) and [NiBr₂(**L^5^**)], were tested in ethylene oligomerization to evaluate the influence of the cavity on the catalytic outcome.

## 2 Results and Discussion

### 2.1 Synthesis and coordination properties

**Scheme 1:**
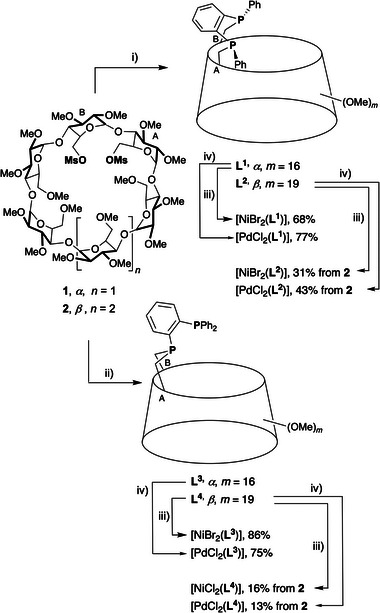
Synthesis of diphosphanes **L^1^**‐**L^4^** and *d* ^8^ complexes [NiBr_2_(**L^1^**‐**L^3^**)], [NiCl_2_(**L^4^**)] and [PdCl_2_(**L^1^**‐**L^4^**)] i) **4** (1.5 equiv.), THF, 25 °C, 8 h, then mesitylene, 160 °C, 4 h; ii) **5** (1.2 equiv.), THF, 12 h; iii) [NiBr_2_(DME)] or [NiCl_2_(DME)] (1 equiv.), CH_2_Cl_2_, 25 °C; iv) [PdCl_2_(COD)], (1 equiv.), CH_2_Cl_2_, 25 °C.

**Scheme 2:**
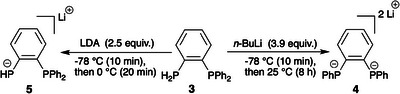
Reactivity of phosphane **3** in the presence of excess *n*‐BuLi or LDA.

Nickel complexes [NiBr_2_(**L^1^**)] and [NiBr_2_(**L^3^**)] (Scheme 1) were synthesized in 68% and 86% yield, respectively from [NiBr_2_(DME)] (DME = 1,2‐dimethoxyethane) and the previously reported *α*‐CD‐based diphosphanes **L^1^** and **L^3^**, which were themselves obtained from dimesylate **1**.^[^ ^14^ ^]^ Complexes [NiBr_2_(**L^2^**)] and [NiCl_2_(**L^4^**)], featuring a larger *β*‐CD cavity were not obtained from the pure ligands **L^2^** and **L^4^**, but from their crude mixtures. In the case of ligand **L^2^**, reacting dimesylate **2** with bis(phosphide) **4** – formed via a Smiles‐like rearrangement of diphosphane **3** upon treatment with excess *n*‐BuLi in THF (Scheme 2)^[^ ^14^, ^15^ ^]^ – yielded a mixture of three diastereomeric diphosphanes^[^ ^15^ ^]^ with the desired *cis*‐chelating bidentate ligand **L^2^** being the major product. Heating this mixture in mesitylene increased the proportion of **L^2^**, and subsequent treatment with [NiBr₂(DME)] enabled the desired chelate complex [NiBr_2_(**L^2^**)] to be separated from non‐chelate complexes by column chromatography. As with complexes [NiBr_2_(**L^1^**)] and [NiBr_2_(**L^3^**)] partial halide metathesis occurred during chromatographic purification, leading to a mixture of bromido and chlorido complexes. Treating this mixture with excess LiBr in MeOH quantitatively yielded the pure dibromido complex [NiBr_2_(**L^2^**)] in 31% yield starting from dimesylate **2**.

Ligand **L^4^** was synthesized by reacting dimesylate **2** with monophosphide **5**, which was obtained by treating diphosphane **3** with excess LDA (LDA = lithium diisopropylamide) (Scheme 2).^[^ ^14^ ^]^ Under these conditions, the previously observed Smiles‐like rearrangement did not occur. In stark contrast to the *α*‐CD analog **L^3^**, pure ligand **L^4^** could not be isolated, as the P‐bridging reaction in this case was not 100% stereoselective. Instead, an inseparable mixture of **L^4^** and its diastereomer (Figure S1) was obtained in an approximate 2:1 ratio. To prevent halide exchange during chromatographic purification, the chlorido complex was synthesized instead of its bromido analog by reacting the mixture of diasteromers containing **L^4^** with [NiCl₂(DME)] (Scheme 1). The desired complex [NiCl₂(**L^4^**)] was stable enough to be purified via column chromatography, yielding 16% starting from dimesylate **2**.

**Scheme 3:**
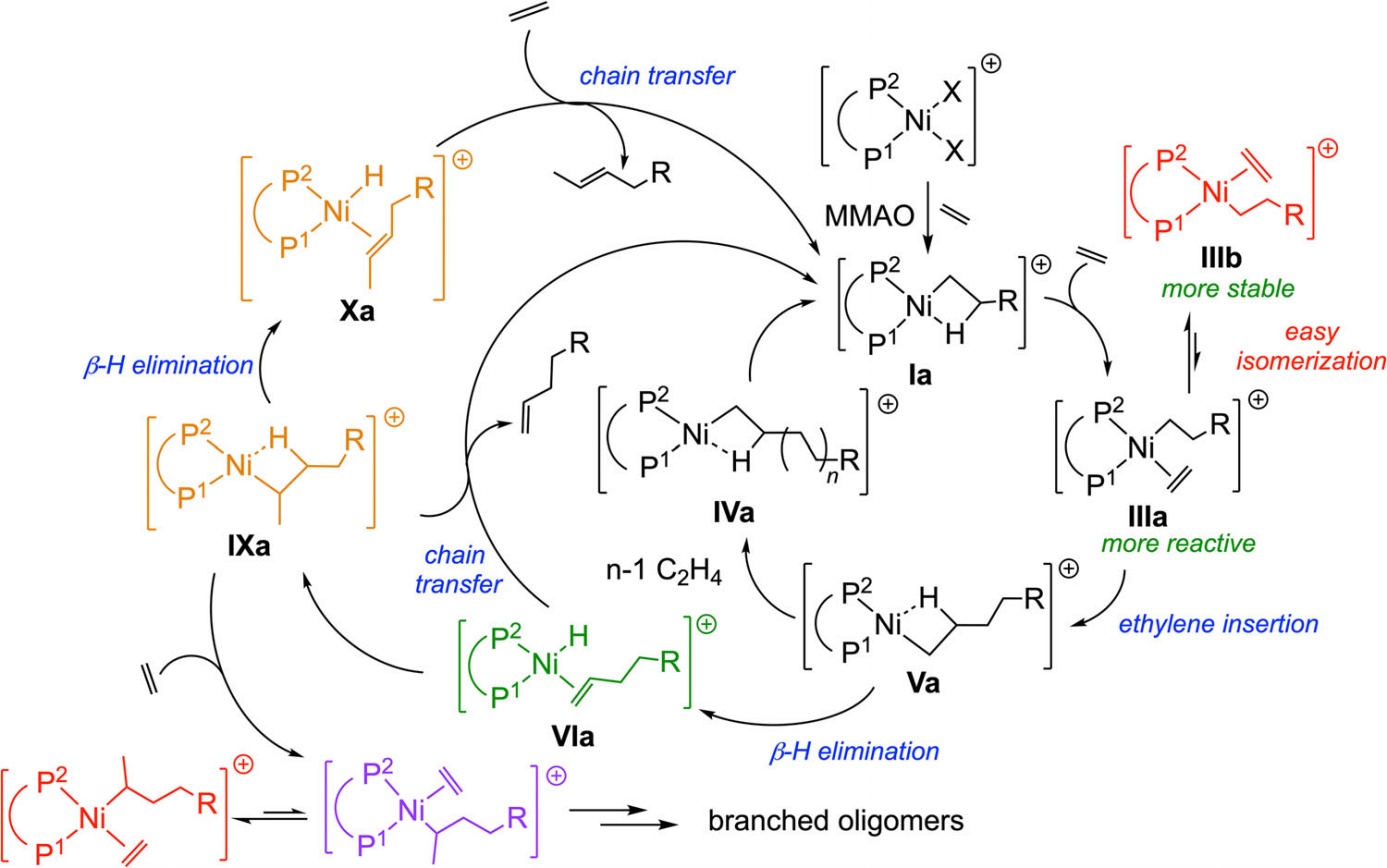
Ni(II)‐catalyzed ethylene oligomerization with the ethyl group coordinated in the active complex **I** *trans* to P^1^ (**a**, in black) or *trans* to P^2^ (**b**, in red). Not all intermediates are drawn.

**Figure 1:**
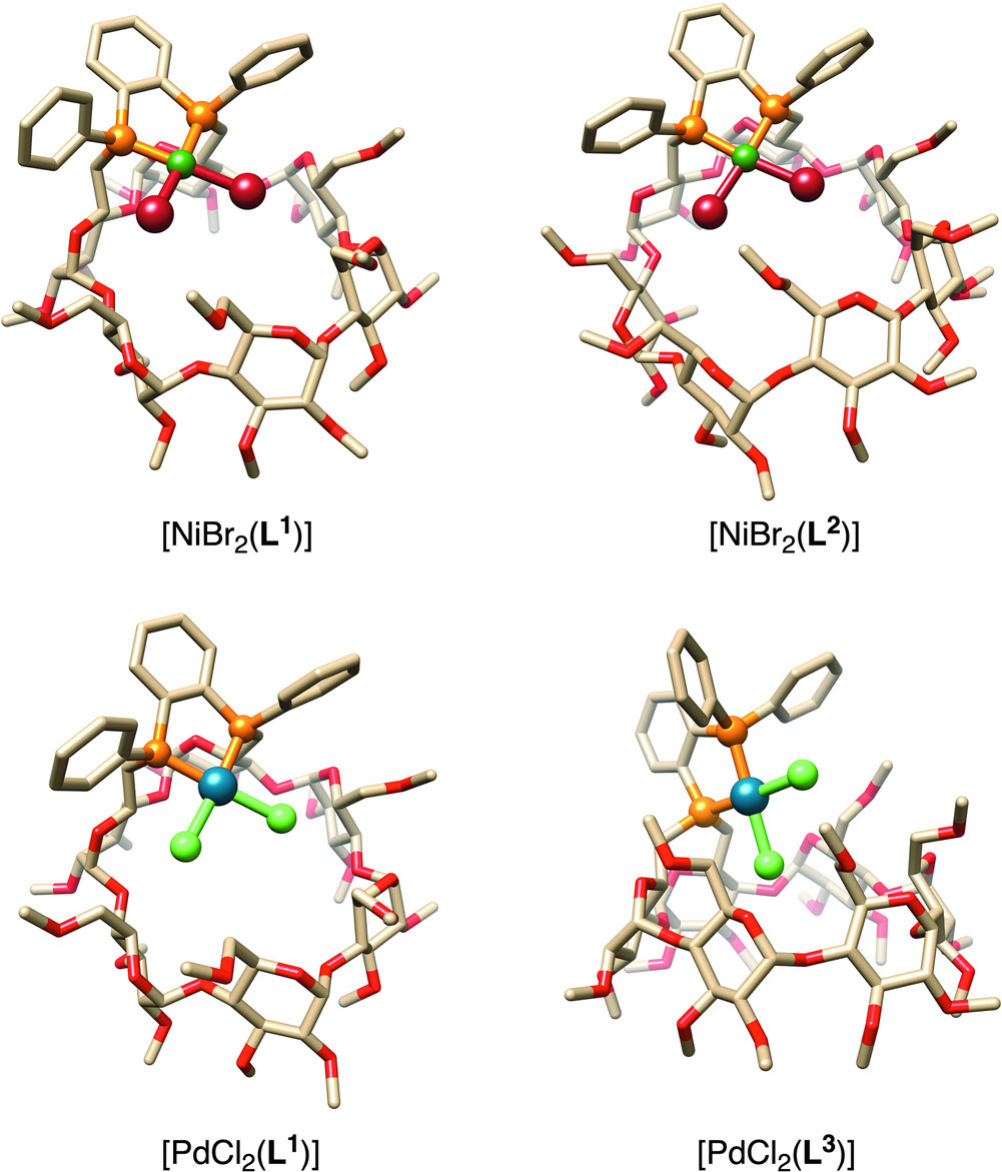
Molecular structures of complexes [NiBr_2_(**L^1^**)], [NiBr_2_(**L^2^**)], [PdCl_2_(**L^1^**)], and [PdCl_2_(**L^3^**)] featuring an encapsulated MX_2_ unit. Solvent molecules are omitted for clarity; C: fawn, O: red; P: orange, Cl: light green, Br: dark red, Ni: dark green, Pd: cyan.

The PdCl₂ complexes [PdCl_2_(**L^1^**‐**L^4^**)] were also synthesized by reaction of [PdCl₂(COD)] (COD = cyclooctadiene) with their respective ligands, whether pure or not (Scheme 1). As observed for their nickel and cavity‐shaped P^N counterparts,^[^ ^13^ ^]^ only *cis*‐complexes with a 1:1 metal/CD ligand ratio were formed, even in the presence of excess ligand. Encapsulation of the MX_2_ units in the square planar complexes [NiBr_2_(**L^1^**)], [NiBr_2_(**L^2^**)], [PdCl_2_(**L^1^**)], and [PdCl_2_(**L^3^**)] was confirmed by X‐ray diffraction analysis (Figure 1).^[^ ^16^ ^]^ In each case, the MX₂ unit is directed toward the cavity interior. In complex [PdCl_2_(**L^3^**)], the PdCl₂ moiety is nearly perpendicular to the mean O(4) plane of the CD with α angles between the P₂MX₂ unit and the O(4) plane of 86.5° and 86.1° for the two CD complexes present in the unit cell. In contrast, the MX₂ units in complexes [NiBr_2_(**L^1^**)], [NiBr_2_(**L^2^**)], and [PdCl_2_(**L^1^**)] tend to lie more parallel to the corresponding O(4) plane (*α* = 17.3° in [NiBr_2_(**L^1^**)]; 24.8° and 32.5° in the two complexes present in the unit cell of [NiBr_2_ **(L^2^**)] and 24.3° in [PdCl_2_(**L^1^**)]). The relatively small *α* angle of 17.3° in the complex [NiBr_2_(**L^1^**)] is best explained by the presence of the small *α*‐CD cavity, unsuited to accommodate the large NiBr₂ unit, whereas the smaller PdCl₂ unit fits better within the *α*‐CD cavity of complex [PdCl_2_(**L^1^**)]. The two M─P bonds have approximately the same length in complexes [NiBr_2_(**L^1^**)], [NiBr_2_(**L^2^**)], and [PdCl_2_(**L^1^**)]. The same trend is observed for M─X bonds with the bond closer to the CD inner wall always being shorter than the other. This effect is particularly pronounced in the more constrained complex [NiBr_2_(**L^1^**)] (2.3098(5) Å and 2.3291(5) Å, respectively). Surprisingly, in complex [PdCl_2_(**L^3^**)], both Pd–Cl and Pd–P bond lengths are nearly identical, even though, contrary to complexes [NiBr_2_(**L^1^**)], [NiBr_2_(**L^2^**)], and [PdCl_2_(**L^1^**)], the two phosphorus atoms are not electronically equivalent. Additionally, the two *cis*‐positioned chlorido ligands are located in very different steric environments in [PdCl_2_(**L^3^**)], a rare feature in cavity‐shaped complexes.^[^ ^17^ ^]^ In all the aforementioned complexes, the CD unit retains an undistorted circular shape, with all glucose units adopting the standard ⁴*C*₁ conformation.

The solution behavior of complexes [NiBr_2_(**L^1^**)], [NiBr_2_(**L^2^**)], [PdCl_2_(**L^1^**)], and [PdCl_2_(**L^3^**)] aligns with their solid‐state structural features. Close examination of the ¹H NMR spectra of [NiBr_2_(**L^3^**)], [NiCl_2_(**L^4^**)], [PdCl_2_(**L^3^**)], and [PdCl_2_(**L^4^**)] revealed a significant downfield shift of the H‐5 proton in the bridged unit A upon metal complexation (*δ* ranging from 5.21 to 5.34 ppm, compared to 4.21 ppm for ligand **L^3^**). This shift is characteristic of the presence of an M─X bond inside the CD cavity and is further supported by the short distance between the H‐5 proton of the bridged glucose unit A and the encapsulated chloride in the molecular structure of complex [PdCl_2_(**L^3^**)] (2.74 and 2.84 Å).^[^ ^18^ ^]^ Similar but less pronounced downfield shifts of other H‐5 protons (Δ*δ* ∼0.5 ppm) were observed for complexes [NiBr_2_(**L^1^**)], [PdCl_2_(**L^1^**)], [NiBr_2_(**L^2^**)], and [PdCl_2_(**L^2^**)] indicating a shallower metal inclusion. This observation is consistent with the molecular structures of [NiBr_2_(**L^1^**)], [NiBr_2_(**L^2^**)], and [PdCl_2_(**L^1^**)].


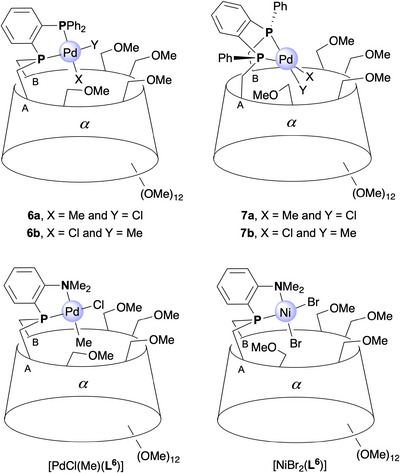


To obtain palladium complexes capable of functioning as precatalysts for ethylene oligomerization, the [PdCl(Me)(diphosphane)] complexes **6** and **7** were synthesized by reaction of [PdCl(Me)(COD)] with diphosphanes **L** **^3^** and **L** **^1^**, respectively in THF. Each reaction yielded an inseparable mixture of isomeric complexes, **6a,b** and **7a,b**, differing in the position of the Pd─Cl bond. For comparison, the Pd complex [PdCl(Me)(**L** **^6^**)] derived from a previously reported P^N cavity‐shaped ligand (**L^6^**), was also prepared.^[^ ^13^ ^]^

Careful analysis of the ¹H and ^3^¹P{¹H} NMR spectra (C_6_D_6_) of the **6a,b** mixture clearly confirmed the presence of two isomers. The ^3^¹P{¹H} NMR spectrum displays two distinct AB patterns, each corresponding to one isomer. In **6a**, the two doublets of the AB pattern are closely spaced (*δ*_31P_ = 41.0 and 41.7 ppm, with ^3 + 2^ *J*_P,P_ = 23.6 Hz), whereas in **6b**, they are significantly farther apart (*δ*_31P_ = 29.1 and 57.1 ppm, with ^3 + 2^ *J*_P,P_ = 25.7 Hz). An HMQC (¹H–^3^¹P) experiment (Figure S92) enabled the assignment of the more basic bridging phosphorus atom to the more upfield‐shifted doublets (41.0 ppm for **6a** and 29.1 ppm for **6b**). Clearly, the PdMeCl complexes **6a,b** adopt a *cis*‐chelate configuration similar to the PdCl₂ analog [PdCl_2_(**L^3^**)]. However, since the two exogenous ligands (chlorido and methyl) are no longer identical, two distinct isomers can form upon complexation. Each isomer was readily distinguished by 2D ROESY NMR. The ROESY spectrum of the mixture revealed a strong correlation between the Pd‐methyl protons of **6a** and the inner cavity H‐5 protons of several glucose units (Figure S94), confirming their location inside the CD cavity. In contrast, no such correlations were observed for **6b** but only between Pd‐methyl protons and the H‐6 protons, consistent with the methyl group being positioned outside the cavity. Additionally, in the ¹H–^31^P HMQC NMR spectrum, the bridging phosphorus atom in **6b** exhibits a strong correlation with the *trans*‐disposed palladium‐bound methyl protons (Figure S92), whereas the correlation between the same methyl group and the *cis*‐disposed *P*Ar₂Ar' atom appears significantly weaker. As expected, the opposite trend was observed for **6a**, consistent with the methyl group being *trans* to the *P*Ar₂Ar' atom and therefore positioned inside the CD cavity. This reflects the larger orbital overlap between the methyl group and the phosphorus atom via the metal d_x2__‐y2_ orbital when the donor atoms are *trans*‐disposed. Indeed, the methyl groups in both **6a** and **6b** resonate as doublets of doublets (*δ*_1H_ = 1.13 ppm with ^3^ *J*_P_ *_transH_* = 7.3 Hz and ^3^ *J*_P_ *_cisH_* = 2.5 Hz for **6a**, and *δ*_1H_ = 1.23 ppm ^3^ *J*_P_ *_transH_* = 8.7 Hz and ^3^ *J*_P_ *_cisH_* = 3.2 Hz for **6b**). In both cases, as expected, the coupling constant is significantly larger for the *trans*‐disposed P(III) and methyl groups.^[^ ^19^ ^]^ Further evidence supporting the location of the chlorido ligand inside the CD cavity in **6b** is the presence of a strongly downfield‐shifted H‐5 signal (*δ*_1H_ = 5.31 ppm) corresponding to one of the capped glucose units. In contrast, no such downfield shift is observed for **6a** (*δ*_1H_ = 4.77 ppm), where the methyl ligand is included in the cavity. The ^1^H NMR spectrum recorded in CD_2_Cl_2_ revealed a **6a**:**6b** ratio of approximately 23:77 at room temperature (Table 1). Knowing that the reaction temperature could influence this isomeric ratio^[^ ^20^ ^]^ the complexation was then conducted in the same solvent (THF) at low temperature (−78 °C). However, no change in the isomeric ratio was observed at this temperature. Changing the NMR solvent (CD_2_Cl_2_ instead of C_6_D_6_) also did not affect the isomeric ratio. Surprisingly, the **6a**:**6b** ratio varied significantly when the complexation was carried out in different solvents. When CH_2_Cl_2_ was used as the reaction solvent instead of THF, **6a** was found to be the major isomer (**6a**:**6b** = 68:32, at a reaction temperature of 25 °C), and the **6a**:**6b** ratio even increased slightly when the reaction was conducted at low temperature (**6a**:**6b** = 80:20, at −78 °C). Moreover, to determine whether isomers **6a** and **6b** are in equilibrium, three different reaction mixtures – obtained in CH_2_Cl_2_ at either 25 °C or −78 °C and in THF at 25 °C – were analyzed by NMR spectroscopy over an extended period of time at 25 °C. Their ^1^H and ^31^P{^1^H} NMR spectra recorded in CD_2_Cl_2_ remained unchanged over seven days, indicating the absence of any equilibrium, at least at room temperature. Note that this contrasts with the solution behavior previously reported for PdMeCl complexes derived from heterodiphosphanes.^[^ ^19^, ^21^ ^]^ Clearly, the formation of **6b** over **6a** is kinetically favored in THF, whereas the opposite is true in CH_2_Cl_2_. Determining which factors – electronic or steric – influence most the isomeric ratio is challenging, given the complexity of the ligand and the small energy differences involved. As for the PdMeCl complexes of **L^3^**, metal complexation of **L^1^** with [PdCl(Me)(COD)] in THF produced two different isomers (**7a,b**) in a **7a**:**7b** ratio of 60:40. In the ^31^P{^1^H} NMR spectrum of the mixture, the two phosphorus atoms in each compound resonate as a pair of doublets (*δ* ^31^P = 41.8 and 54.5 ppm with ^3 + 2^ *J*_P,P_ = 25.9 Hz for the major isomer **7a**, and *δ*_31P_ = 34.6 and 63.7 ppm with ^3 + 2^ *J*_P,P_ = 26.6 Hz for the minor isomer **7b**). Given the complexity of the ^1^H NMR spectra of the **7a,b** mixture, full assignment for each isomer could not be achieved, and the exact position of the methyl group within the cavity in both isomers could not be determined from NMR studies (see X‐ray studies below for the assignment of **7a** and **7b**).^[^ ^22^ ^]^ However, the ^1^H‐^31^P HMQC spectrum (Figure S99), as for **6a,b**, shows clearly that the phosphorus atom belonging to one of the bridged units in either **7a** or **7b** correlates strongly with the *trans*‐disposed palladium‐bound methyl protons, whereas the cross‐peaks are much less intense for correlations between the same methyl group and the second P(III) atom, which is *cis* to the methyl group. This methyl group resonates as a doublet of doublets at *δ*_1H_ = 0.39 ppm (^3^ *J*_P_ *_trans_*_H_ = 8.3 Hz and ^3^ *J*_P_ *_cis_*_H_ = 3.9 Hz) for **7a** and 0.47 ppm (^3^ *J*_P_ *_trans_*_H_ = 8.5 Hz and ^3^ *J*_P_ *_cis_*_H_ = 3.4 Hz) for **7b** with in both cases a much larger coupling constant for the mutually *trans* P(III) and methyl groups compared to the *cis* ones. As in the case of **L^3^**, metal complexation was carried out in different solvents and at different temperatures (Table 1). Similar to **6a,b**, the same proportion of the two isomers (**7a**:**7b** ratio = 60:40) was observed when the reaction was performed in THF at either room or low temperature (−78 °C). In contrast, the ratios were 47:53 and 42:58 at room temperature and −78 °C, respectively, when metal complexation was conducted in CH₂Cl₂. A 6:4 mixture of **7a** and **7b** was monitored by ^31^P and ¹H NMR spectroscopy in CD₂Cl₂ at 25 °C over an extended period. During the first few days, the proportion of **7a** and **7b** remained largely unchanged, but after seven days, some decomposition occurred, with the **7a**:**7b** ratio shifting to approximately 2:8. This observation suggests that isomer **7a** decomposes faster than **7b**. However, a slow equilibrium between the two isomers cannot be entirely ruled out.^[^ ^7^, ^19^ ^]^

**Table 1:** Isomeric ratios of **6a** to **6b** and **7a** to **7b** resulting from the metal complexation of cavity‐shaped diphosphanes 1 and 2 with [PdClMe(COD)] at different reaction conditions.

Solvent, temperature	**6a:6b**	**7a:7b**
THF, 25 °C	23:77	60:40
THF, −78 °C	23:77	60:40
CH_2_Cl_2_, 25 °C	68:32	47:53
CH_2_Cl_2_, −78 °C	80:20	42:58

**Figure 2:**
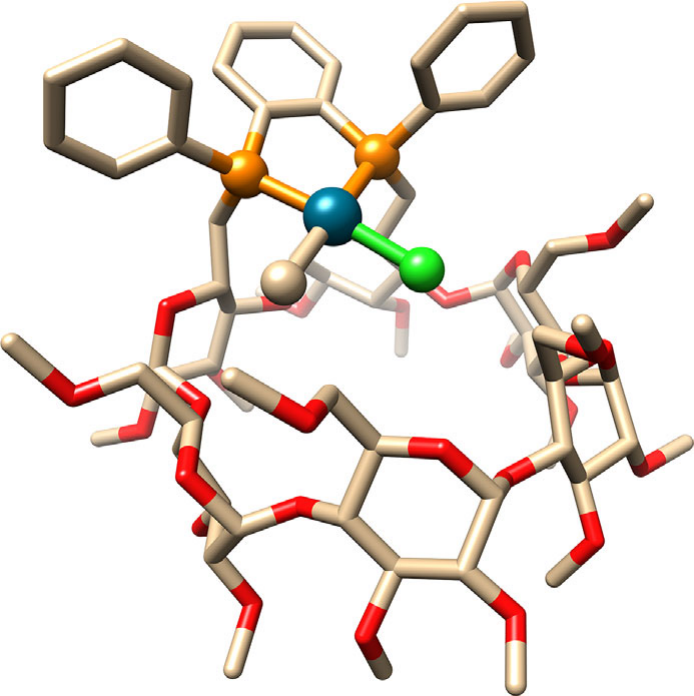
Molecular structure of complexes **7a,b** (70% Cl1/C78 (**7b**), 30% Cl1A/C78A (**7a**)). The C78/Cl1A and C78A/Cl1 atoms are located at the same positions. The Pd‐Cl1/Pd‐C78 and Pd‐Cl1A/Pd‐C78A distances are therefore averaged. Only the major isomer (**7b**) is depicted. Hydrogen atoms and solvent molecules are omitted for clarity; C: fawn, O: red, P: orange, Cl: light green, Pd: cyan).

Single crystals suitable for X‐ray diffraction analysis^[^ ^16^ ^]^ were obtained by slow diffusion of *n*‐pentane into a solution of **7a,b** in CH_2_Cl_2_. Both geometrical isomers, **7a** and **7b**, are present in the single crystal in a 3:7 ratio, respectively. Only the major isomer (**7b**) is depicted in Figure 2. The single crystals used for X‐ray diffraction analysis were redissolved in CD₂Cl₂, and the ¹H and ^3^¹P NMR spectra were quickly recorded. The same isomeric ratio as in the solid state (3:7) was observed in CD₂Cl₂ solution, allowing for the identification of both isomers in the NMR spectra.^[^ ^23^ ^]^ X‐ray crystal structure analysis of **7a,b** confirmed the chelating behavior of the diphosphane as well as the square planar geometry of the complexes. The angle between the PPPd plane and the CD O(4) atoms plane is 21.76°, a value nearly identical to that observed in [PdCl_2_(**L^1^**)]. Therefore, complexes **7a,b** and [PdCl_2_(**L^1^**)] experience a very similar steric environment within the CD cavity.

In the case of complex [PdCl(Me)(**L^6^**)], only one isomer was observed. Because the H‐5 proton of unit B is hardly downfield‐shifted upon complexation (*δ*_H_ = 4.41 ppm versus 4.31 ppm for the P^N ligand), the chlorido ligand is likely located outside the cavity.

The position of the Pd‐methyl fragment inside the CD was inferred from a ROESY experiment. Thus, the PdMe signal of [PdCl(Me)(**L** ^**6**^)], which resonates as a doublet at 0.78 ppm, gave rise to cross‐peaks corresponding to through‐space correlations involving all inner cavity H‐5 protons (Figure S108). The orientation of the methyl ligand does not result from its affinity for the *α*‐CD inner space but rather from the relative *trans* influence of the individual ligands present in [PdCl(Me)(**L** **^6^**)].^[^ ^24^ ^]^ Consistent with the experimentally established orienting series (*trans*‐influence order: CH_3_^–^ >> PR_3_ > Cl^–^ > NR_3_), the methyl group can only be *cis* to phosphorus and inside the CD cavity, whereas the chlorido ligand has to be *trans* to the phosphorus donor atom and outside the cavity.

### 2.2 Catalysis

**Figure 3:**
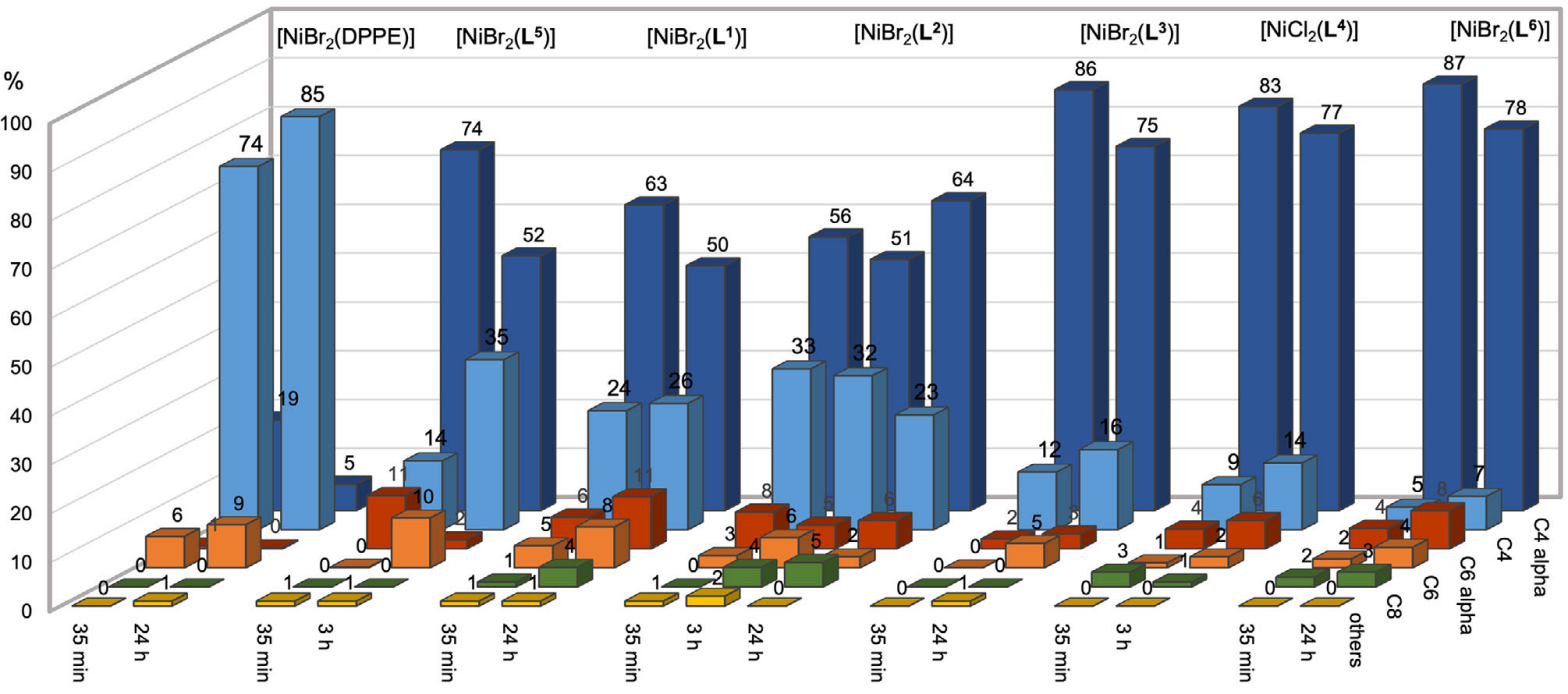
Selectivities of complexes [NiBr_2_(DPPE)], [NiBr_2_(**L^1^‐L^3^**)], [NiCl_2_(**L^4^**)] and [NiBr_2_(**L^5^‐L^6^**)] for C4 compounds and *α*‐olefins using MMAO as cocatalyst.

All cavity‐shaped Ni complexes [NiBr_2_(**L^1−^L^3^**)] and [NiCl_2_(**L^4^**)] were tested for their ability to catalyze the oligomerization of ethylene after activation with modified methylaluminoxane (MMAO). Their catalytic properties were compared to those of cavity‐free analogs [NiBr_2_(DPPE)]^[^ ^25^ ^]^ and [NiBr_2_(**L** **^5^**)]^[^ ^26^ ^]^ and P^N complex [NiBr_2_(**L^6^**)]^[^ ^13^ ^]^ (Table 2 and Figure 3). Complexes [NiBr_2_(**L^3^**)] and [NiCl_2_(**L^4^**)] primarily produced C₄ olefins (up to 98%) and 1‐butene (up to 90%), similar to the analogous cavity‐shaped P^N complex [NiBr_2_(**L^6^**)], albeit with slightly lower *α*‐olefin selectivity (Table 2, entries 10–15). Unsurprisingly, the larger *β*‐CD analog [NiCl_2_(**L^4^**)] is much more active than its *α*‐CD counterpart [NiBr_2_(**L^3^**)] with nearly identical selectivities (Table 2, entries 10–13). Compared to their cavity‐free analog [NiBr_2_(**L^5^**)], both complexes [NiBr_2_(**L^3^**)] and [NiCl_2_(**L^4^**)] are significantly more selective with only minor loss of activity in the case of complex [NiCl_2_(**L^4^**)] after prolonged reaction times. Again, as for the P^N complex [NiBr_2_(**L^6^**)], the *α*‐olefin content is high for both complexes [NiBr_2_(**L^3^**)] and [NiCl_2_(**L^4^**)] (Table 2, entries 10–13), but not for cavity‐free [NiBr_2_(DPPE)] and [NiBr_2_(**L^5^**)] (Table 2, entries 1–4) for prolonged reaction times, pointing to a scenario where chain‐walking and post‐isomerization processes are disfavored within the CD cavity. Complex [NiBr_2_(**L^1^**)] which displays an *α*‐CD cavity‐shaped environment very different from [NiBr_2_(**L^3^**)] is much more active (Table 2, entries 5, 6, 10 and 11) than the latter but tends to deactivate over prolonged reaction times. Surprisingly, the *β*‐CD‐based precatalyst [NiBr_2_(**L^2^**)] is less active than its smaller counterpart [NiBr_2_(**L** **^1^**)] and also tends to deactivate over time (Table 2, entries 7–9). Moreover, the *α*‐olefin selectivity for [NiBr_2_(**L^1^**)] and [NiBr_2_(**L^2^**)] is somewhat lower than that of [NiBr_2_(**L^3^**)], [NiCl_2_(**L^4^**)] and the P^N complex [NiBr_2_(**L^6^**)], (Table 2, entries 5–9) and is closer to the selectivity observed for the cavity‐free complex [NiBr_2_(**L^5^**)] (Table 2, entries 3 and 4), but much higher than the electronically similar complex [NiBr_2_(DPPE)] (Table 2, entries 1 and 2).

**Table 2:** Ethylene oligomerization with nickel complexes [NiBr_2_(DPPE)], [NiBr_2_(**L** **^1^**‐**L^3^**)], [NiCl_2_(**L** **^4^**)], [NiBr_2_(**L^5^**)], and [NiBr_2_(**L^6^**)] as precatalysts in the presence of MMAO^[^ ^a^ ^]^.

				Products weight distribution [%]^[^ ^c^ ^]^		
Entry	Precatalyst	Time	TOF^[^ ^b^ ^]^	C_4_ [α]^[^ ^d^ ^]^	C_6_ [*α*]^[^ ^e^ ^]^	C_8_
1	[NiBr_2_(DPPE)]	35 min	17 871	93(20)	7(8)	<1
2	[NiBr_2_(DPPE)]	24 h	8760	90(6)	9(2)	<1
3	[NiBr_2_(**L^5^**)]	35 min	80 816	88(84)	11(99)	<1
4	[NiBr_2_(**L^5^**)]	3 h	25 141	87(60)	12(15)	<1
5	[NiBr_2_(**L^1^**)]	35 min	21 824	87(72)	11(59)	<2
6	[NiBr_2_(**L^1^**)]	24 h	2432	76(66)	19(56)	4
7	[NiBr_2_(**L^2^**)]	35 min	7405	89(63)	10(75)	<1
8	[NiBr_2_(**L^2^**)]	3 h	5445	83(62)	11(44)	4
9	[NiBr_2_(**L^2^**)]	24 h	2661	87(73)	8(72)	5
10	[NiBr_2_(**L^3^**)]	35 min	2115	98(88)	2(100)	0
11	[NiBr_2_(**L^3^**)]	24 h	1050	91(82)	8(38)	<1
12	[NiCl_2_(**L^4^**)]	35 min	24 137	92(90)	5(80)	3
13	[NiCl_2_(**L^4^**)]	3 h	20 948	91(85)	8(72)	1
14^[^ ^13^ ^]^	[NiBr_2_(**L^6^**)]	35 min	5780	92(95)	6(70)	2
15^[^ ^13^ ^]^	[NiBr_2_(**L^6^**)]	24 h	4000	85(92)	12(65)	3

^[a]^ Conditions: amount of catalyst: 1 × 10^−5^ mol, amount of cocatalyst (MMAO‐12): 4 × 10^−3^ mol (400 equiv.), T = 30–35 °C, solvent: toluene, total volume: 20 mL, 10 bar C_2_H_4_, every test was repeated at least twice;

^[b]^ turnover frequency ([mol(C_2_H_4_) mol(Ni)^−1^ h^−1^]);

^[c]^ % calculated by GC analysis;

^[d]^ 1‐butene vs total butenes formed;

^[e]^ 1‐hexene vs total hexenes formed.

All attempts to oligomerize ethylene with PdMeCl complexes **6a,b**, **7a,b** and [PdCl(Me)(**L^6^**)]^[^ ^24^ ^]^ upon activation with NaBAr^F^_4_ failed, whether in toluene or in the more polar solvent CH_2_Cl_2_, with only trace amounts of butenes observed.

### 2.3 Theoretical studies

**Figure 4:**
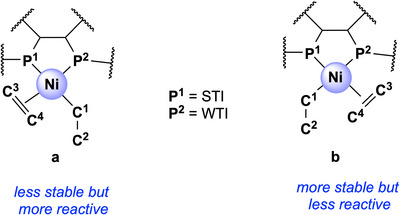
Atom labeling in a generic P^P complex with the ethylene group coordinated *trans* to P^2^ (**a**) or *trans* to P^1^ (**b**). In this scheme, P^1^ is the P with strong *trans*‐influence (STI) and P^2^ is the P with the weak *trans*‐influence (WTI).

To investigate the reactivity of *cis*‐chelating diphosphanes, as well as the influence of the CD cavity on the Ni^II^‐catalyzed ethylene dimerization, a computational study was performed on the key steps of the reaction by adopting a seldom‐used methodology for very large molecules based on the GFN2‐xTB density functional tight binding method.^[^ ^27^ ^]^ The complex selected for this study was the one with the highest *α*‐olefin selectivity in the *α*‐CD series, namely the cavity‐shaped complex [NiBr_2_(**L^3^**)], along with its cavity‐free analog [NiBr_2_(**L^5^**)]. The key atoms involved in the reactions catalyzed by Ni^II^‐complexes [NiBr_2_(**L^5^**)] (with **P^P** ligand) and [NiBr_2_(**L^3^**)] (with **P^P^CD^** ligand) after MMAO activation are shown in Figure 4. This scheme illustrates the two possible modes of ethylene coordination: *trans* to P^2^ (with the ethyl group *trans* to P^1^) or *trans* to P^1^ (with the ethyl group *trans* to P^2^). Goldman et al.^[^ ^28^ ^]^ emphasized the importance of *trans‐*influence in ethylene insertion into nickel‐alkyl bonds catalyzed by X^Y bidentate ligands. Their work demonstrated the roles of strong *trans*‐influence (STI) and weak *trans*‐influence (WTI) in stabilizing intermediates such as π‐complexes and agostic interactions, as well as their impact on system reactivity, particularly in the case of SHOP‐type P^O ligands. They showed that ethylene insertion occurs with lower energy barriers in the presence of STI^WTI bidentate ligands (such as P^O) compared to STI^STI ones (e.g., P^P). STI^WTI ligands stabilize the π‐complex, leading to an overall decrease in the ethylene insertion energy barrier. However, the present study reveals that functional groups bonded to the phosphorus atoms in a nonsymmetric diphosphane (P^P) ligand can alter the system's reactivity, mimicking STI^WTI behavior.

The reaction mechanism follows the established pathway outlined in Scheme 3, based on the general mechanism for oligomerization reactions.^[^ ^29^ ^]^ Since CD‐based diphosphanes are large ligands with around 200 atoms, it is challenging if not impossible to fully optimize these cavity‐shaped Ni^II^ complexes using density functional theory (DFT) methods. Therefore, the semiempirical tight‐binding method GFN2‐xTB^[^ ^27^ ^]^ was selected to model the Ni^II^‐catalyzed ethylene dimerization using CD‐based ligands. The GFN2‐xTB method enables rapid calculations of molecular systems containing up to 1000 atoms and has been successfully applied in studies of organometallic complexes, although it does have limitations when it comes to accurately modeling the coordination geometries of transition metal complexes.^[^ ^27^, ^30^ ^]^ In this study, the steps of ethylene insertion into the Ni‐C bond catalyzed by the cavity‐free complex [NiBr_2_(**L^5^**)] after MMAO activation (Figures S119–S122) were first calculated using both the PBE^[^ ^31^ ^]^‐D3(BJ)^[^ ^32^ ^]^/def2‐TZVP^[^ ^33^ ^]^/COSMO^[^ ^34^ ^]^ (toluene) level of theory and the GFN2‐xTB/ALPB^[^ ^35^ ^]^ (toluene) level of theory. Furthermore, the enthalpy profile of the reaction was calculated for the cavity‐free system for the **a**‐type and **b**‐type intermediates. The optimized geometries and energies obtained from both methods were compared to validate the results from the GFN2‐xTB method before applying it to the CD‐based Ni^II^ complexes. Enthalpy, rather than Gibbs free energy, was used to construct the comparative reaction coordinate energy profile. Enthalpies were also used in the final GFN2‐xTB calculations to provide a clearer visualization of the reaction energy profiles.

This initial benchmarking study demonstrated that GFN2‐xTB is well‐suited for investigating Ni^II^‐catalyzed ethylene dimerization, offering a good balance between computational cost and result quality. The energy difference between the activation barriers (Δ*H^‡^*) calculated using DFT and GFN2‐xTB methods was only of around 1 kcal/mol (Figures S119–S122, Tables S1–S5). Our calculations also revealed that the PPh_2_ coordinating unit acts as a weaker *trans*‐influencer (WTI) than the P*^i^* Pr_2_ unit, which means that the **L^5^‐IIa** intermediate is less stable but more reactive than **L^5^‐IIb**, with the **a**‐type intermediates driving the catalytic cycle (Figures S121–S122, Table S1). Details of this benchmarking study are provided in the Supporting Information.

**Figure 5:**
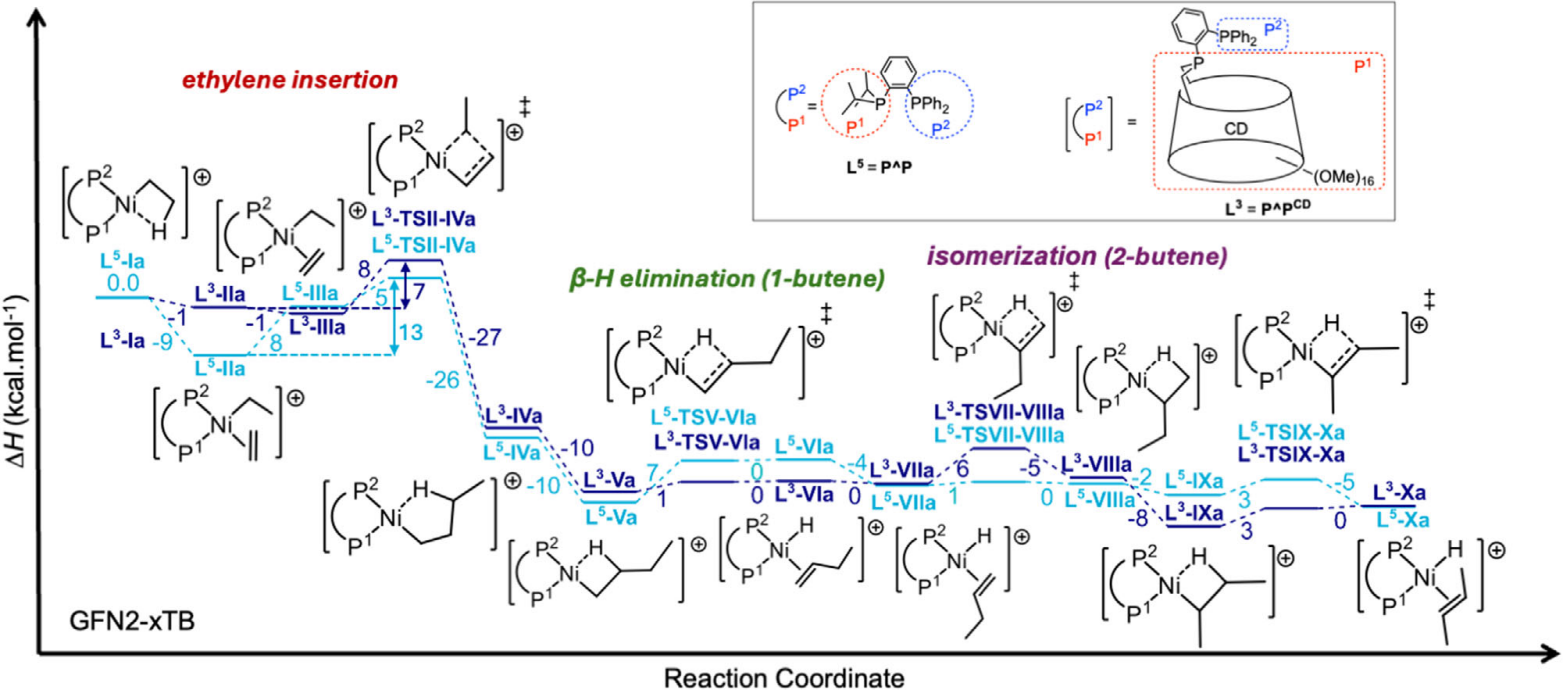
Enthalpy profiles for ethylene dimerization catalyzed by complex [NiBr_2_(**L^5^**)] (**P^P** ligand) in light blue and by complex [NiBr_2_(**L^3^**)] (**P^P^CD^** ligand) in dark blue after MMAO activation. Relative enthalpy values are given in kcal/mol relative to the respective active species (**P^P‐Ia** for complex [NiBr_2_(**L^5^**)] and **P^P^CD^‐Ia** for complex [NiBr_2_(**L^3^**)]) obtained from geometry optimizations at the GFN2‐xTB/ALPB(toluene) level of theory.

Thus, the GFN2‐xTB method was applied to study the reaction pathways involving complexes [NiBr_2_(**L^5^**)], (**P^P** ligand) and [NiBr_2_(**L^3^**)] (**P^P^CD^** ligand). The dimerization and isomerization steps follow the pathway outlined in Scheme 3. The reaction coordinate for ethylene dimerization was computed with the ethyl group coordinated *trans* to P^1^ (**a**‐type intermediate) (Figure 5) and *trans* to P^2^ (**b**‐type intermediate) (Figure S124). In the following discussion, the distinction between the **a** and **b** isomers will not always be made, especially when referring to the general steps. The reaction coordinate enthalpy profiles, shown in Figures 5, and S123, with enthalpies for all the steps listed in Table 3, indicate that ethylene coordination is more stabilized in the **P^P** system than in the **P^P^CD^** one, particularly for the **L^5^/L^3^‐II** pairs (Figures S125, and S127). The reaction profile begins with the active species **L^5^/L^3^‐I**, followed by ethylene coordination, which yields the *π*‐complex **L^3^/L^5^‐II**. The *π*‐complexes in the presence of cyclodextrin, **L^3^‐IIa** (Δ*H =* −1 kcal/mol) and **L^3^‐IIb** (Δ*H =* −5 kcal/mol), are significantly less stabilized than the corresponding cavity‐free complexes **L^5^‐IIa** (Δ*H =* −9 kcal/mol) and **L^5^‐IIb** (Δ*H =* −14 kcal/mol), respectively. However, the ligands have first to position themselves in a favorable conformation (*syn* coplanar to the metal‐alkyl bond), through a 90° rotation about the Ni‐olefin η^2^ bond, leading to structures **L^3^/L^5^‐III** (Figures S126, and S128). This rotation of the olefin from **L^3^/L^5^‐II** to **L^3^/L^5^‐III** is barrierless. The difference of enthalpy between these two rotamers in the **a**‐ and **b**‐type intermediates is significantly increased by the presence of the CD, **L^3^‐IIIa** (Δ*H =* −1 kcal/mol) versus **L^3^‐IIIb** (Δ*H* = 14 kcal/mol) and **L^5^‐IIIa** (Δ*H =* 8 kcal/mol) vs **L^5^‐IIIb** (Δ*H* = 13 kcal/mol). Overall, **L^3^/L^5^‐II** intermediate is more stable than **L^3^/L^5^‐III**. After the formation of the **L^3^/L^5^‐III** intermediate (*syn* coplanar with the metal‐alkyl bond), the system passes through a transition state for ethylene insertion. This process has an activation enthalpy of Δ*H^‡^ =* 5 kcal/mol from **L^5^‐IIIa** and **L^5^‐IIIb**, Δ*H^‡^ =* 8 kcal/mol from **L^3^‐IIIa** and is nearly barrierless from **L^3^‐IIIb**. The overall payload of the insertion of the ethylene into the Ni‐C1 bond should be considered as being the sum of the rotational enthalpy payload and the activation barrier of the insertion reaction. The energy barriers of ethylene insertion into the Ni‐C1 bond are lower for **L^3^‐TSII‐IVa** (Δ*H^‡^* = 7 kcal/mol) and **L^3^‐TSII‐IVb** (Δ*H^‡^* = 13 kcal/mol) than for **L^5^‐TSII‐IVa** (Δ*H^‡^* = 13 kcal/mol) and **L^5^‐TSII‐IVb** (Δ*H^‡^* = 18 kcal/mol), indicating that the environment conferred by the CD cavity lowers the activation barrier. Following **TSII‐IV**, the butyl group is formed with a *β*‐agostic interaction (**L^3^/L^5^‐IV**). The **L^3^‐IVa** intermediate is stabilized by approximately 1 kcal/mol more than the **L^5^‐IVa** intermediate, which lacks the CD ligand, while **L^3^‐IVb** is approximately 7 kcal/mol more stable than **L^5^‐IVb** (Figure S124). The butyl group in **L^5^/L^3^‐IV** subsequently rotates to form a more stabilizing interaction, specifically a *β*‐agostic one with the C4‐H (See Figure 4 for labeling) bond (**L^5^/L^3^‐V**). In this case, **L^3^‐Va** is approximately 1 kcal/mol less stable than **L^5^‐Va** (Figure 5), while **L^3^‐Vb** is about 5 kcal/mol more stable than **L^5^‐Vb** (Figure S124). From **L^3^/L^5^‐V**, the reaction can proceed along two possible pathways: a second ethylene insertion leading to chain growth (which is not explored in this study), or a *β*‐H elimination via **TSV‐VI**, forming the hydrido‐π‐complex **L^3^/L^5^‐VI**. Once **L^3^/L^5^‐VI** formed, 1‐butene can either undergo chain transfer, where a new ethylene molecule coordinates and 1‐butene is released, thereby restarting the catalytic cycle at **L^3^/L^5^‐I**, or the Ni‐bound olefin in **L^3^/L^5^‐VI** can rotate, in order to orient the olefin *syn* coplanar to the hydride to form **L^5^‐VII** (Δ*H =* −4 kcal/mol for **L^5^‐VIIa** and Δ*H* = 0 kcal/mol for **L^5^‐VIIb**) or **L^3^‐VII** (Δ*H* = 0 kcal/mol for **L^3^‐VIIa** and Δ*H* = ‐2 kcal/mol for **L^3^‐VIIb**). Similar to the rotation seen for coordinated ethylene, the rotation of 1‐butene is expected to proceed in a barrierless fashion. However, in the presence of a CD, steric hindrance imposed by the cavity makes this rotation energetically demanding, as the cavity must undergo conformational changes to make the rotation possible. The transition state of this rotation remained elusive despite efforts to locate it.

**Table 3:** Relative enthalpy (Δ*H*) in kcal/mol of the stationary points of ethylene insertion in the Ni‐C1 bond catalyzed by complex [NiBr_2_(**L^5^**)] (**P^P** ligand) and complex [NiBr_2_(**L^3^**) (**P^P^CD^** ligand) after MMAO activation with the ethyl group coordinated in the active complex *trans* to P^1^ (**a**) and *trans* to P^2^ (**b**) computed at the GFN2‐xTB/ALPB (toluene) level of theory.

	Δ*H* [kcal/mol]			
Intermediate [Y]	L^5^‐Ya^[^ ^a^ ^]^	L^5^‐Yb^[^ ^a^ ^]^	L^3^‐Ya^[^ ^b^ ^]^	L^3^‐Yb^[^ ^b^ ^]^
**I**	0.0	0.9	0.0	3.5
**II**	−9.3	−13.4	−1.5	−4.9
**III**	−1.4	−0.4	−2.5	8.6
**TSII‐IV**	3.1	4.5	6.0	8.1
**IV**	−22.6	−23.3	−21.0	−26.4
**V**	−33.0	−32.5	−31.2	−34.8
**TSV‐VI**	−26.2	−31.3	−29.7	−29.2
**VI**	−26.1	−32.5	−29.5	−28.6
**VII**	−30.3	−32.9	−30.0	−30.9
**TSVII‐VIII**	−29.6	−32.5	−24.2	−31.5
**VIII**	−29.9	−35.4	−28.9	−37.6
**IX**	−31.8	−35.8	−36.8	−35.3
**TSIX‐X**	−29.2	−32.6	−34.0	−31.1
**X**	−33.7	−32.9	−33.5	−31.0

^[a]^ Enthalpy relative to complex **L^5^‐Ia** + ethylene;

^[b]^ Enthalpy relative to complex **L^3^‐Ia** + ethylene.

To assess the impact of the CD cavity on 1‐butene rotation, we then conducted a study (See Supporting Information, section 6.3) where we calculated the energy of complexes **L^3^/L^5^‐VI** with rotated 1‐butene, but without relaxing the geometry. As expected, no significant hindrance was observed for complex **L^5^‐VI**. However, in complex **L^3^‐VI**, rotation of 1‐butene resulted in structural clashes and high energy, indicating that the CD cavity restricts 1‐butene rotation towards **L^3^‐VII**. A third pathway passes back through **TSVI‐V** via a 1,2‐insertion, thereby returning to **L^3^/L^5^‐V** with minimal activation energy.

Additionally, **L^3^/L^5^‐VII** can undergo a 2,1‐insertion via **TSVII‐VIII**, with Δ*H^‡^* = 1 kcal/mol for **L^5^‐TSVII‐VIIIa**, Δ*H^‡^* = 0 kcal/mol for **L^5^‐TSVII‐VIIIb**, and Δ*H^‡^* = 6 kcal/mol for **L^3^‐TSVII‐VIIIa** and Δ*H^‡^* = −1 kcal/mol for **L^3^‐TSVII‐VIIIb** forming **L^3^‐VIIIa** and **L^3^‐VIIIb**, respectively. This result indicates that the hydride insertion is barrierless in the **b** path in stark contrast to the **a** path. In **L^3^‐VIIIb**, where the chain is positioned outside the CD cavity, the olefin is already *syn* coplanar oriented to the hydride, closely resembling the geometry of **L^3^‐TSVII‐VIIIb**. Conversely, in **L^3^‐TSVII‐VIIIa**, where the chain is inside the CD cavity, the olefin is perpendicular to the hydride due to the steric constraints imposed by the CD cavity, thereby requiring more energy to reach **L^3^‐TSVII‐VIIIa**. **L^3^/L^5^‐VIII** exhibit a *β*‐agostic interaction involving the C3─H bond (See Figure 4 for labeling), where H is the hydrogen atom originally bonded to C4, which underwent *β*‐H elimination, and was subsequently inserted into the C3 carbon via **L^3^/L^5^‐TSVII‐VIII**. The more active pathway via **L^3^‐TSVII‐VIIIa** is approximately 5 kcal/mol higher in energy than **L^5^‐TSVII‐VIIIa** indicating that isomerization is less favored within the CD cavity. The enthalpy profile suggests that once the **L^3^/L^5^**‐**VI** intermediate has rotated to form **L^3^/L^5^‐VII**, the barrier to 2,1‐insertion is considerably low. This very small difference in enthalpy does not explain the higher yield of *α*‐C4 olefin observed for the **P^P^CD^** system. On the other hand, the restricted 1‐butene rotation on going from complex **L^3^**‐**VI** to **L^3^**‐**VII** is clearly inhibiting 1‐butene isomerization within the CD cavity. After the formation of intermediates **L^3^/L^5^‐VIII**, the butyl group can rotate to establish a *β*‐agostic interaction between H1 and the nickel center (Figure 4), yielding **L^3^/L^5^‐IX**. These intermediates then undergo *β*‐H elimination via **TSIX‐X**, with an energy barrier of approximately 3 kcal/mol for both the cavity‐free and cavity‐shaped complexes, ultimately leading to the formation of the coordinated isomerized product, namely 2‐butene (**L^3^/L^5^‐X**).

**Figure 6:**
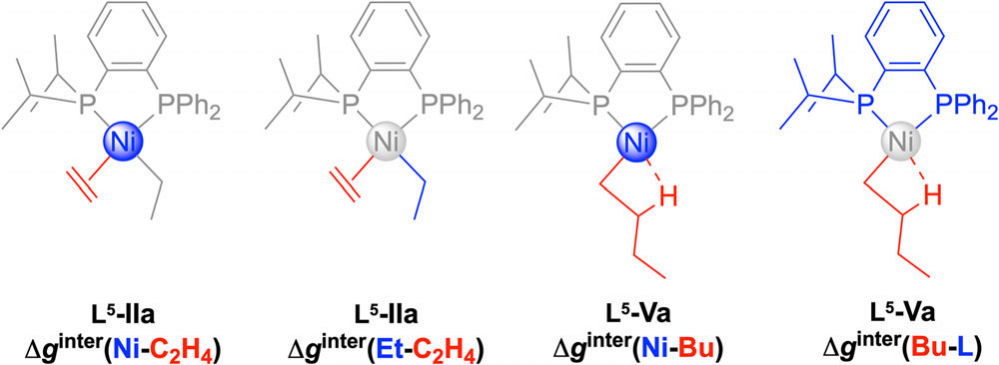
Fragments used for the IGM‐Δ*g* ^inter^ analysis of **L^5^‐IIa** and **L^5^‐Va**. For Δ*g* ^inter^_Bu‐L_ (right), the first fragment is the C4 substrate in red and the second one is the ligand in blue. In grey are the not interacting atoms in the respective Δ*g* ^inter^ score. L is the ligand (**L^3^** or **L^5^**), and Bu is the C4 substrate, which in **L^5^‐Va** is 1‐butyl. The Δ*g* ^inter^ score for the interaction between the C4 substrate and ligand is referred to as Δ*g* ^inter^_Bu‐L_.

To investigate the effect of the CD ligand and its interactions with both the substrate and the Ni metal center, an electron density‐based analysis was performed using the Independent Gradient Model (IGM).^[^ ^36^ ^]^ The local descriptor Δ*g* ^inter^ which quantifies electron density (ED) sharing between two user‐specified fragments was computed to assess the interaction strength between the **L^5^** or **L^3^** ligand and the substrate. IGM analysis was carried out for both cavity‐free and cavity‐shaped complexes. Throughout the reaction, the substrates coordinated to the metal vary (e.g., ethyl + ethylene in **L^3^/L^5^‐II**, **L^3^/L^5^‐TSII‐IV**, or butyl in **L^3^/L^5^‐V**). The C2 substrates are referred to as Et or C_2_H_4_ while the C4 substrate is named Bu throughout the reaction. On the other hand, the ligand (**L^3^** or **L^5^**) is denoted as L. Figure 6 illustrates the two defined fragments in the Δ*g* ^inter^ analysis for the **L^5^** ligand only.

**Figure 7:**
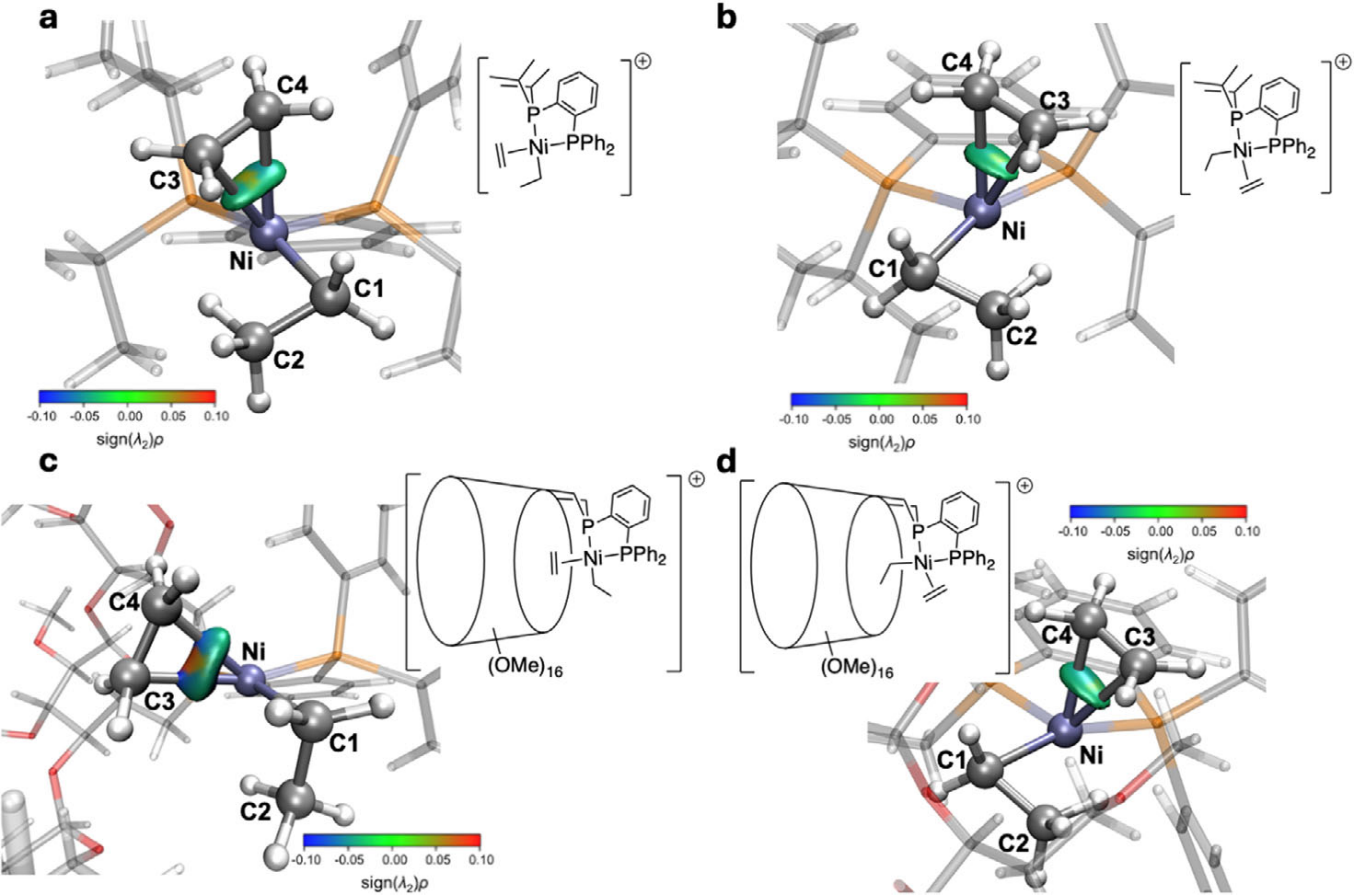
Zoomed‐in view of the δ*g* ^inter^ isosurface plot (cutoff: δ*g* ^inter^ = 0.04 a_0_^−4^ and Blue Green Red color scale in the range 0.1 < sign(*λ_2_*)*ρ* < 0.1 a_0_^−3^) displaying the interaction between Ni and C_2_H_4_ for (a) **L^5^‐IIa**, (b) **L^5^‐IIb**, (c) **L^3^‐IIa**, and (d) **L^3^‐IIb**. Electron density was computed at the PBE‐D3(BJ)/def2‐TZVP/CPCM(toluene) level of theory from geometry optimized at the GFN2‐xTB/ALPB(toluene) level of theory. Surface color code: blue = attractive interaction, green = nonbonding interactions, red = repulsive interactions. Atom color code: hydrogen (white), carbon (silver), phosphorus (orange), and nickel (ice blue).

The interaction between the diphosphane, whether cavity‐shaped or cavity‐free, and Ni (Δ*g* ^inter^_Ni‐L_) was also evaluated (Table S18). The Δ*g* ^inter^ analysis revealed that intermediate **L^3^‐II** exhibits weaker interactions between Ni and the C4 substrate (Δ*g* ^inter^_Ni‐Bu_ = 2.082 *a*_0_^−1^ (*a*_0_ = Bohr radius) for **L^3^‐IIa** and Δ*g* ^inter^_Ni‐Bu_ = 1.655 *a*_0_^−1^ for **L^3^‐IIb**) compared to intermediate **L^5^‐II** (Δ*g* ^inter^_Ni‐Bu_ = 2.169 a_0_^−1^ for **L^5^‐IIa** and Δ*g* ^inter^_Ni‐Bu_ = 2.131 a_0_^−1^ for **L^5^‐IIb**). In contrast, the Δ*g* ^inter^ for the interaction between Ni and ethylene was found to be stronger in the presence of the **P^P^CD^** ligand in intermediate **L^3^‐II** (Δ*g* ^inter^_Ni‐C2H4_ = 0.925 *a*_0_^−1^ for **L^3^‐IIa** and Δ*g* ^inter^_Ni‐C2H4_ = 0.722 *a*_0_^−1^ for **L^3^‐IIb**) compared to intermediate **L^5^‐II** (Δ*g* ^inter^_Ni‐C2H4_ = 0.737 *a*_0_^−1^ for **L^5^‐IIa** and Δ*g* ^inter^_Ni‐C2H4_ = 0.711 *a*_0_^−1^ for **L^5^‐IIb**). The interaction involving ethylene coordinated inside the CD cavity (**L^3^‐IIa**) is particularly strong (Figure 7 and Figure S137). These results indicate that ethylene is more strongly activated through its interaction with Ni when it is located inside the CD cavity. This activation is promoting ethylene insertion by strengthening the interaction with the ethyl ligand.

**Figure 8:**
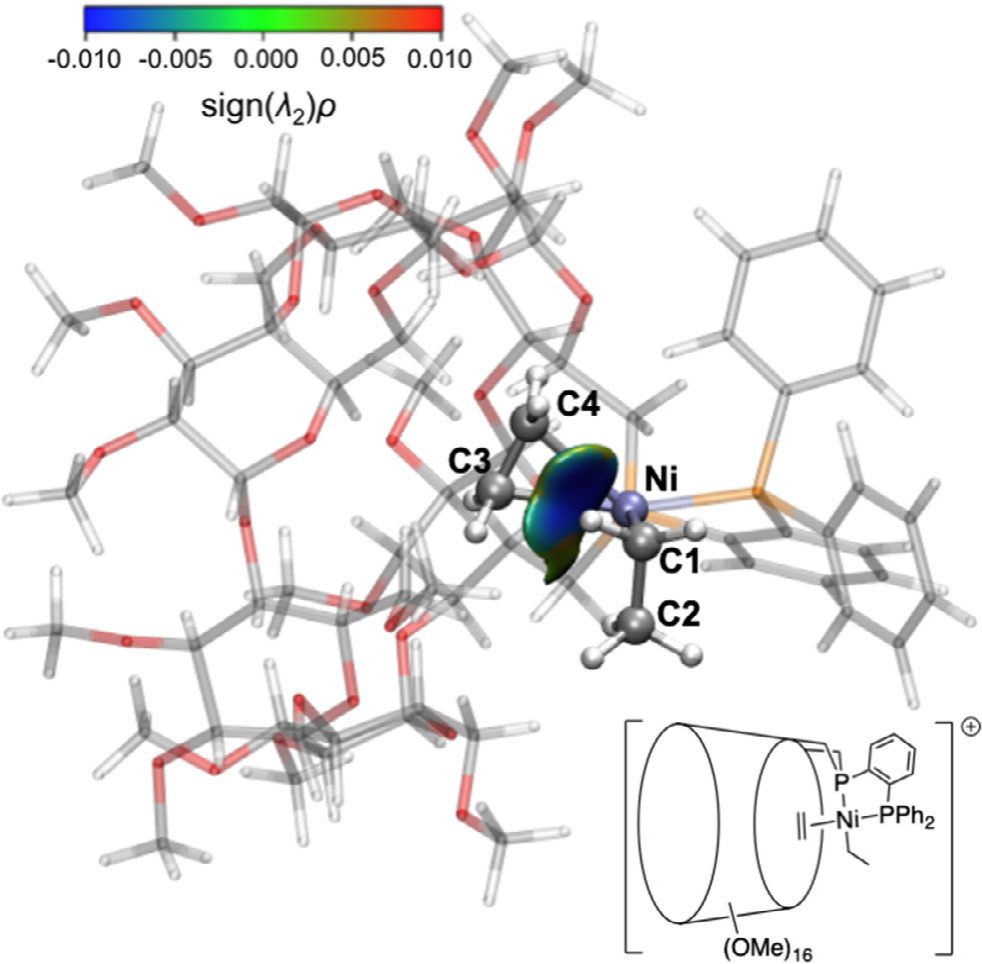
δ*g* ^inter^ isosurface plot (cutoff: δ*g* ^inter^ = 0.005 a_0_^−4^ and Blue Green Red color scale in the range 0.1 < sign(*λ_2_*)*ρ* < 0.1 a_0_^−3^) displaying the interaction between ethyl and C_2_H_4_ in **L^3^‐IIa**. Electron density was computed at the PBE‐D3(BJ)/def2‐TZVP/CPCM(toluene) level of theory from geometry optimized at the GFN2‐xTB/ALPB(toluene) level of theory. Surface color code: blue = attractive interaction, green = nonbonding interactions, red = repulsive interactions. Atom color code: hydrogen (white), carbon (silver), phosphorus (orange), and nickel (ice blue).

The interaction between the ethyl and ethylene ligands was also investigated for complexes **L^3^/L^5^‐II**. The ethyl‐C_2_H_4_ interaction is stronger for the cavity‐shaped complex (Δ*g* ^inter^_Et‐C2H4_ = 0.150 *a*_0_^−1^ for **L^3^** **‐** **IIa** (Figure 8 and Figure S138) and Δ*g* ^inter^_Et‐C2H4_ = 0.113 *a*_0_^−1^ for **L^3^‐IIb**, Figure S139) compared to the cavity‐free complex (Δ*g* ^inter^_Et‐C2H4_ = 0.127 *a*_0_^−1^ for **L^5^‐IIa**, Figure S138, and Δ*g* ^inter^_Et‐C2H4_ = 0.107 *a*_0_^−1^ for **L^5^‐IIb**, Figure S139). This demonstrates that the confined environment provided by the CD enhances the ethylene‐Ni interaction consequently strengthening the interaction between ethylene and the alkyl group, which facilitates ethylene insertion and reduces the corresponding activation barrier.

Additionally, the interaction between the C4 substrate (Bu) and the **P^P^CD^** ligand (Δ*g* ^inter^_Bu‐L_ = 1.483*a*_0_^−1^ for **L^3^‐IIa**) is nearly twice as strong as that of the CD‐free **P^P** ligand (Δ*g* ^inter^_Bu‐L_ = 0.845 a_0_^−1^ for **L^5^‐IIa**). These larger Δ*g* ^inter^ values are, at least in part, due to the bigger size of the **P^P^CD^** ligand compared to the **P^P** ligand, offering a larger “contact surface” between the two fragments at stake.

**Figure 9:**
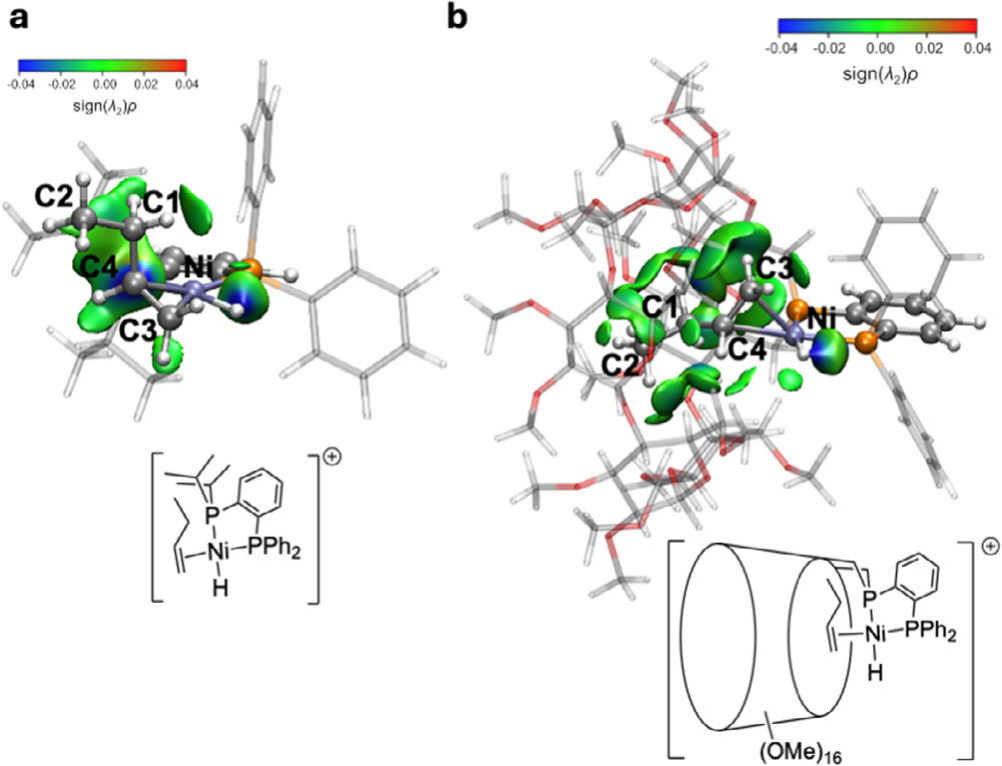
δ*g* ^inter^ isosurface plot (cutoff: δ*g* ^inter^ = 0.005 a_0_^−4^ and Blue Green Red color scale in the range 0.04 < sign(*λ_2_*)*ρ* < 0.04 a_0_^−3^) displaying the interaction between (a) Bu and **P^P** ligand in intermediate **L^5^‐VIIa** and (b) between Bu and **P^P^CD^** ligand in intermediate **L^3^‐VIIa**. Electron density was computed at the PBE‐D3(BJ)/def2‐TZVP/CPCM(toluene) level of theory from geometry optimized at the GFN2‐xTB/ALPB(toluene) level of theory. Surface color code: blue = attractive interaction, green = nonbonding interactions, red = repulsive interactions. Atom color code: hydrogen (white), carbon (silver), phosphorus (orange), and nickel (ice blue).

Further investigation into the interaction between the **P^P^CD^** ligand and the substrate (Bu) was carried out using the δ*g* ^inter^_Bu‐L_ isosurface plot (Figure 9 and Figures S131–S136) and the Δ*g* ^inter^_Ni‐L_ attractive and repulsive scores (Table S19). The δ*g* ^inter^ isosurface plot revealed that the **P^P^CD^** ligand displays larger non‐bonding interaction areas with the substrate than the **P^P** ligand. These interactions are likely to be responsible for the constrained, and thus less favorable, rotation of the olefin in the CD‐system (**L^3^‐VI** to **L^3^‐VII**) than in the cavity‐free one (**L^5^‐VI** to **L^5^‐VII**). These analyses indicate that the attractive interactions between the substrate and the ligands are stronger than the repulsive interactions in the cavity‐shaped complexes. The δ*g* ^inter^ isosurface plot shows that attractive interactions between the **P^P^CD^** ligand and C1‐H and C2‐H bonds (See Figure 4 for labeling) are notably stronger (evidenced by the blue color parts in the isosurface plot) than in the case of the **P^P** ligand. This behavior suggests that these attractive interactions, which stabilize the substrate, play a crucial role in inhibiting isomerization within the CD cavity.

The IGM analysis indicates that the CD environment not only influences isomerization through steric hindrance, but also affects electron density distribution in the system, thereby modifying Ni reactivity, its interactions with the substrates, and ligand‐substrate interactions. These effects are reflected in the energy profile, which aligns with the experimental results.

## 3 Conclusion

Four *cis*‐chelating, CD‐based diphosphanes have been shown to exhibit unique coordination behavior with various *d* ^8^ cations within their well‐defined cavity. The study of the ligands' coordination properties was facilitated by the fact that CD cavities are lined with protons, which can serve as NMR probes for the encapsulated metal center. The ability to easily modulate sterics in these cavity‐shaped ligands enabled us to investigate the impact of metal confinement on nickel‐catalyzed ethylene oligomerization. In this context, it was demonstrated that metal confinement, as seen in a previously studied cavity‐shaped P^N Ni^II^ complex, positively affects catalyst robustness and *α*‐olefin selectivity by inhibiting olefin isomerization. Theoretical studies have shown that the restricted rotation of the olefinic substrate within the confined environment of the CD cavity is responsible for the high *α*‐olefin selectivity observed in the most selective cavity‐shaped systems. Additionally, it was found that activity can remain reasonably high without compromising selectivity if the cavity is large enough, as exemplified by the *β*‐CD complex [NiCl_2_(**L^4^**)]. Further catalytic studies involving metal‐confining and *cis*‐chelating ligands with various donor atoms are currently underway.

## 4 Experimental Section

Detailed experimental materials and methods as well as theoretical data can be found in the Supporting Information. The authors have cited additional references within the Supporting Information.^[^ ^37^ ^]^

## Conflict of Interests

The authors declare no conflict of interest.

## Supporting information



Supporting Information

## Data Availability

The data that support the findings of this study are available in the supplementary material of this article.
