# Supplementary material for: Cis‐Chelating Diphosphanes for Intracavity Nickel(II)‐Catalyzed Ethylene Oligomerization
Source: Chemistry. 2025 May 24;31(34):e202501188. doi: 10.1002/chem.202501188 (PMC12172601; doi:10.1002/chem.202501188)
Supplement: Supplementary file 1 — Supporting Information [file CHEM-31-e202501188-s001.pdf]

Table of Contents

Structures of ligands **L**[1]-**L**[6] and their complexes S3

Experimental Details S4

1. General methods S4

2. Synthesis and characterization of complexes [$NiBr_2$(**L**^{1}-**L**^{3})], [$NiCl_2$(**L**^{4})], [$NiBr_2$(**L**^{5})], [$PdCl_2$(**L**^{1}-**L**^{4})], [PdCl(Me)(**L**^{6})], **6a,b** and **7a,b** S5

3. Procedure for ethylene oligomerization reactions S19

4. NMR and mass spectra of complexes [$NiBr_2$(**L**^{1}-**L**^{3})], [$NiCl_2$(**L**^{4})], [$NiBr_2$(**L**^{5})], [$PdCl_2$(**L**^{1}-**L**^{4})], [PdCl(Me)(**L**^{6})], **6a,b** and **7a,b** S21

5. Crystal structure analyses S79

6. Computational details S86

References S113

Structures of ligands L^1-L^6 and their complexes

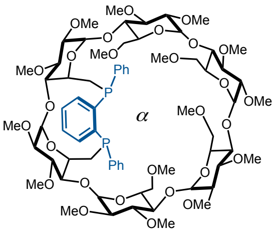

L^1

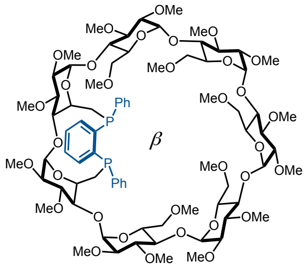

L^2

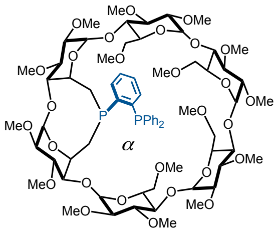

L^3

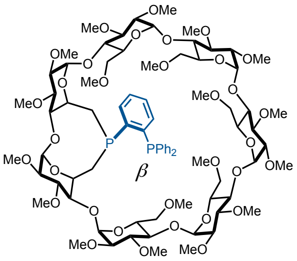

L^4

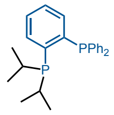

L^5

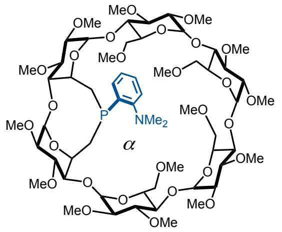

L^6

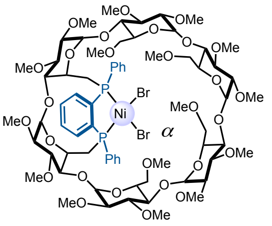

[$NiBr_2$(**L^1**)]

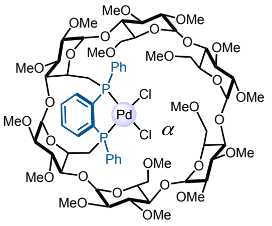

[$PdCl_2$(**L^1**)]

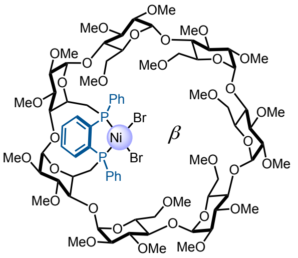

[$NiBr_2$(**L^2**)]

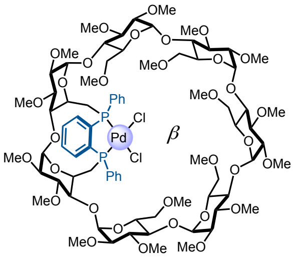

[$PdCl_2$(**L^2**)]

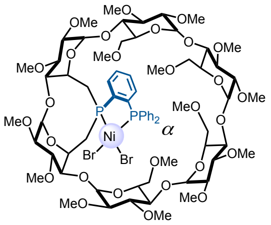

[$NiBr_2$(**L^3**)]

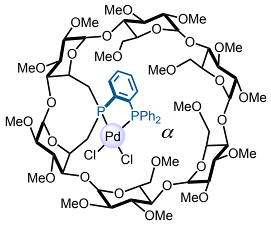

[$PdCl_2$(**L^3**)]

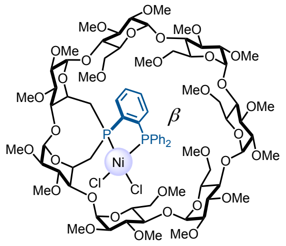

[$NiCl_2$(**L^4**)]

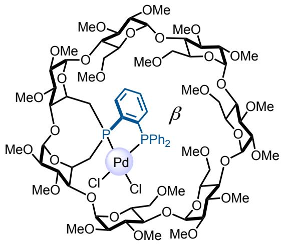

[$PdCl_2$(**L^4**)]

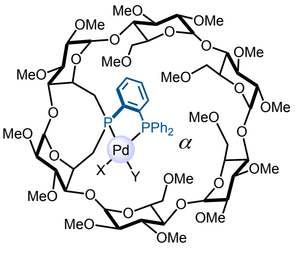

6a, X = Me and Y = Cl
6b, X = Cl and Y = Me

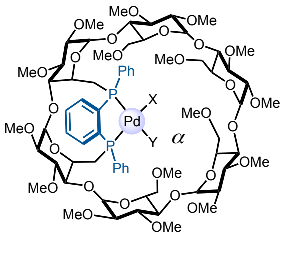

7a, X = Cl and Y = Me
7b, X = Me and Y = Cl

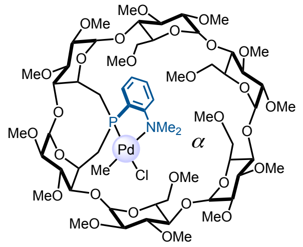

[PdCl(Me)(**L^6**)]

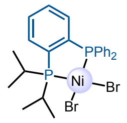

[$NiBr_2$(**L^5**)]

Experimental Details

1. General methods

All reactions and manipulations were carried out under an inert atmosphere (nitrogen or argon) using standard Schlenk techniques. All glassware was stored in the oven prior to use under an inert atmosphere of gas (Argon). All commercial reagents were used as supplied unless otherwise stated. Solvents were dried by conventional methods and distilled immediately prior to use. Deuterated solvents were passed through a 5 cm-thick alumina column and stored under nitrogen over molecular sieves (4 Å). Column chromatography was performed on silica gel 60 (particle size 40-63 µm, 230-240 mesh). Routine ^{1}H, $^{31}P\{^{1}H\}$ and $^{13}C\{^{1}H\}$ NMR spectra were recorded on Bruker FT instruments (AVANCE 300, 400, 500, 600 spectrometers) at room temperature unless otherwise stated. ^{1}H NMR spectral data were referenced to residual protiated solvents (δ = 7.26 ppm for $CDCl_3$, 7.15 ppm for C_6D_6 and 5.32 ppm for CD_2Cl_2), $^{13}C\{^{1}H\}$ chemical shifts are reported relative to deuterated solvents (δ = 77.16 ppm for $CDCl_3$, 128.02 ppm for C_6D_6 and 53.84 ppm for CD_2Cl_2) and the $^{31}P\{^{1}H\}$ NMR data are given relative to external H_3PO_4. Mass spectra were recorded on a Bruker MicroTOF spectrometer (ESI-TOF) using CH_2Cl_2, CH_3CN or CH_3OH as the solvent. Elemental analyses were performed by the Service de Microanalyse, Institut de Chimie UMR 7177, Strasbourg. Dimesylates **1** and **2**[1], diphosphines **L^1**, **L^2**[2], **L^3**[3], and [2-(diisopropylphosphanyl)phenyl]diphenylphosphane[4] (**L^5**) were synthesized according to procedures published previously.

In this publication, the cyclodextrins are depicted as seen from the secondary face, the glucose units being ranged counterclockwise in the following order: A, B, C, D, E, F, G. The numbering of the atoms within a glucose unit is as follows:

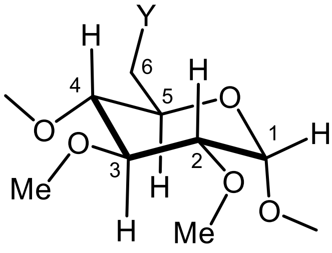

2. Synthesis and characterization of complexes [$NiBr_2$(**L**[1]-**L**[3])], [$NiCl_2$(**L**[4])], [$NiBr_2$(**L**[5])], [$PdCl_2$(**L**[1]-**L**[4])], [PdCl(Me)(**L**[6])], **6a,b** and **7a,b**

Dibromido-[{6^A,6^B-dideoxy-6^A,6^B-[(1*R*,2*S*)-1,2-phenylenebis(phenylphosphanyl)] -2^A,2^B,2^C,2^D,2^E,2^F,3^A,3^B,3^C,3^D,3^E,3^F,6^C,6^D,6^E,6^F-hexadeca-*O*-methyl-α-cyclodextrin}-κ^2-*P*,*P*]nickel(II) (complex [$NiBr_2$(**L**[1])])

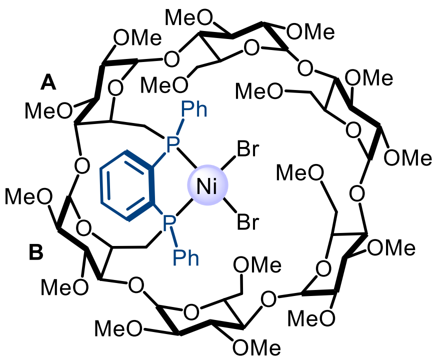

A solution of [$NiBr_2$(DME)] (0.062 g, 0.17 mmol) in CH_2Cl_2 (3 mL) was added to a solution of **L**[1] (0.258 g, 0.17 mmol) in CH_2Cl_2 (3 mL). The reaction mixture was stirred for 2 h at room temperature and then evaporated to dryness under reduced pressure. The resulting orange residue was subjected to column chromatography (SiO_2, CH_2Cl_2/MeOH, 97/3, *v/v*). The solution was dried under vacuum and the resulting solid was redissolved with MeOH and reacted with LiBr for half an hour before adding water (20 mL). The reaction mixture was extracted with CH_2Cl_2 (3 x 25 mL) and the organic extract dried over $MgSO_4$. Removal of the solvent *in vacuo* afford pure [$NiBr_2$(**L**[1])] (0.202 g, 68 %) as an orange solid. A crystalline material was obtained by slow diffusion of *n*-pentane into a benzene solution of [$NiBr_2$(**L**[1])].

^{1}H NMR (500 MHz, CD_2Cl_2, 25 °C): δ (partial assignment by combined COSY, ROESY, TOCSY and HSQC) = 2.44-2.59 (3H, H-$6^{A,B}$), 3.18 (s, 3H, OMe), 3.24 (s, 3H, OMe), 3.26 (s, 3H, OMe), 3.37 (s, 3H, OMe), 3.39 (s, 6H, OMe), 3.40 (s, 3H, OMe), 3.43 (s, 3H, OMe), 3.48 (s, 3H, OMe), 3.51 (s, 3H, OMe), 3.52 (s, 3H, OMe), 3.55 (s, 3H, OMe), 3.59 (s, 3H, OMe), 3.61 (s, 3H, OMe), 3.64 (s, 3H, OMe), 3.67 (s, 3H, OMe), 2.97-3.91 (28H, H-2, H-3, H-4, H-5, H-6), 4.09-4.14 (2H, H-5, H-6), 4.39 (m, H-5), 4.59 (br d, 1H, H-1), 4.92 (br d, 1H, H-1), 4.95 (d, 1H, $^3J_{H\text{-}2,\,H\text{-}1}$ = 3.9 Hz, H-1), 5.00 (d, 1H, $^3J_{H\text{-}2,\,H\text{-}1}$ = 3.2 Hz, H-1), 5.06 (d, 1H, $^3J_{H\text{-}2,\,H\text{-}1}$ = 2.6 Hz, H-1), 5.10 (d, 1H, $^3J_{H\text{-}2,\,H\text{-}1}$ = 3.3 Hz, H-1), 5.09 (m, 1H, H-$5^{B\text{ or }A}$), 5.27 (m, 1H, H-$5^{A\text{ or }B}$), 6.98 (m, 1H, aromatic H), 7.34-7.60 (9H, aromatic H), 7.86 (m, 2H, aromatic H), 7.96 (m, 2H, aromatic H) ppm; ^{13}C{^{1}H} NMR (126 MHz, CD_2Cl_2, 25°C): δ (partial assignment by HSQC) = 31.68 (d, $^1J_{P,C}$ = 35.0 Hz, C-$6^{A\text{ or }B}$), 37.12 (br, C-$6^{B\text{ or }A}$), 57.23, 57.69, 58.40, 58.58, 58.90 [x2], 58.92 [x2], 59.21, 59.26, 60.94, 61.76, 61.90, 61.96, 61.98, 62.11 (OMe), 71.29, 71.36, 71.68, 71.88 (C-$5^{C,D,E,F}$), 71.60 (br), 72.96 (br) (C-$6^{A,B}$) 71.76, 72.02, 72.52 [x2] (C-$6^{C,D,E,F}$), 80.17, 80.54, 81.06, 81.10, 81.53, 81.68, 81.77 [x2], 81.97, 82.69 [x2], 82.85 [x2], 83.10,

83.37, 83.38, 89.51 (br), 91.33 (br) (C-2, C-3, C-4), 99.70, 99.72, 100.13, 100.64, 101.11 (br), 102.24 (br) (C-1), 128.09, 128.49 (quat. aromatic C) 127.98, 128.17, 128.66 (d, $J_{P,C}$ = 10.8 Hz, aromatic C), 129.28 (d, $J_{P,C}$ = 10.7 Hz, aromatic C), 131.47 [x2], 132.45 (d, $J_{P,C}$ = 9.3 Hz, aromatic C), 132.50, 132.63, 132.68, 133.00 (aromatic C), 133.59 (d, $J_{P,C}$ = 9.9 Hz, aromatic C), ppm (2 broad quaternary aromatic C not detected); $^{31}P\{^1H\}$ NMR (202 MHz, CD_2Cl_2, 25°C): δ = 62.4 (d, $^2J_{P,P}$ = 58.1 Hz), 72.9 (d, $^2J_{P,P}$ = 58.1 Hz) ppm; elemental analysis (%) calcd for $C_{70}H_{104}O_{28}P_2NiBr_2$: C 50.22, H 6.26, found: C 50.63, H 6.15; MS (ESI-TOF) for $C_{70}H_{104}O_{28}P_2NiBr_2$: *m/z* (%): 808.23 (5) $[M - Br + Na]^{2+}$, 1593.47 (100) $[M - Br]^+$, 1695.18 (10) $[M + Na]^+$.

Dibromido-[{6^A,6^B-dideoxy-6^A,6^B-[(*R*)-2-(diphenylphosphanyl)phenyl phosphinidene]-2^A,2^B,2^C,2^D,2^E,2^F,3^A,3^B,3^C,3^D,3^E,3^F,6^C,6^D,6^E,6^F-hexadeca-*O*-methyl-*α*-cyclodextrin}-*κ*²-*P*,*P*]nickel(II) ([$NiBr_2(\mathbf{L^3})$].)

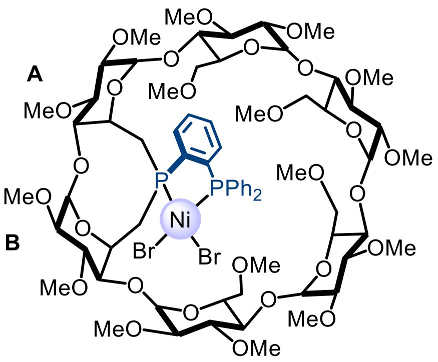

A solution of [$NiBr_2$(DME)] (0.053 g, 0.16 mmol) in CH_2Cl_2 (5 mL) was added to a solution of **L³** (0.227 g, 0.16 mmol) in CH_2Cl_2 (5 mL). The reaction mixture was stirred for 2 h at room temperature and then evaporated to dryness under reduced pressure. The resulting orange residue was subjected to column chromatography (SiO_2, CH_2Cl_2/MeOH, 97/3, *v/v*). The solution was dried under vacuum and the resulting solid was redissolved with MeOH and reacted with LiBr for 30 min hour before adding water (20 mL). The reaction mixture was extracted with CH_2Cl_2 (3 x 25 mL) and the organic extract dried over $MgSO_4$. Removal of the solvent *in vacuo* afforded pure ([$NiBr_2(\mathbf{L^3})$] (0.226 g, 86 %) as an orange solid. ^1H NMR (500 MHz, C_6D_6, 25 °C): δ (partial assignment by combined COSY, ROESY, TOCSY and HSQC) = 2.22 (m, 1H, H-6a^B), 2.42 (m, 1H, H-6a^A), 3.00 (m, 1H, H-4^B), 3.02 (m, 1H, H-6b^A), 3.07 (m, 1H, H-4^A), 3.29 (s, 3H, OMe), 3.29 (s, 3H, OMe), 3.30 (s, 3H, OMe), 3.31 (s, 3H, OMe), 3.33 (s, 3H, OMe), 3.36 (s, 3H, OMe), 3.40 (s, 3H, OMe), 3.53 (s, 3H, OMe), 3.57 (s, 3H, OMe),3.59 (s, 3H, OMe), 3.74 (s, 3H, OMe), 3.78 (s, 3H, OMe), 3.84 (s, 3H, OMe), 3.84 (s, 3H, OMe), 3.84 (s, 3H, OMe), 3.84 (s, 3H, OMe), 3.21-4.73 (28H, H-2, H-3, H-4^{C,D,E,F}, H-5^{C,D,E,F}, H-6b^B, H-6a^C, H-6^{D,E,F}), 4.74 (m, 1H, H-5^B), 4.76 (d, 1H, $^3J_{H-2, H-1}$ = 4.3 Hz, H-1), 4.85 (d, 1H, $^3J_{H-2, H-1}$ = 3.5 Hz, H-1), 5.10 (m, 1H, H-6b^C), 5.31 (d, 1H, $^3J_{H-2, H-1}$ = 3.4 Hz, H-1), 5.33 (d, 1H, $^3J_{H-}$

$_{2,\,H\text{-}1}$ = 3.3 Hz, H-1), 5.35 (d, 1H, $^{3}J_{H\text{-}2,\,H\text{-}1}$ = 3.4 Hz, H-1), 5.44 (d, 1H, $^{3}J_{H\text{-}2,\,H\text{-}1}$ = 3.2 Hz, H-1), 5.60 (m, 1H, H-5^{A}), 6.88 (m, 1H, aromatic H), 6.98-7.14 (8H, aromatic H), 7.82 (m, 1H, aromatic H), 7.86-7.96 (4H, aromatic H) ppm; ^{13}C{^{1}H} NMR (126 MHz, C_6D_6, 25°C): δ (partial assignment by HSQC) = 35.49 (d, $^{1}J_{P,C}$ = 31.8 Hz, C-6^{B}), 43.45 (d, $^{1}J_{P,C}$ = 25.5 Hz, C-6^{A}), 57.22, 57.31 [x2], 57.34, 57.61, 57.67, 58.99, 59.04, 59.14, 59.34, 61.71, 61.89, 61.91 [x2], 62.21, 62.31 (OMe), 66.83 (d, $^{2}J_{P,C}$ = 8.4 Hz, C-5^{B}), 68.64 (C-5^{A}), 71.14, 71.30, 71.64, 71.14, 71.30, 71.64, 71.72, 72.38, 72.78, 72.93, 73.74, 80.91, 81.89, 81.93, 81.96, 82.33, 82.59, 82.90, 82.97, 83.13, 83.29, 83.43, 83.56, 83.66 (C-2, C-3, C-4^{C,D,E,F}, C-5^{C,D,E,F}, C-6$^{C.D,E,F}$), 88.60 (d, $^{3}J_{P,C}$ = 8.9 Hz, C-4^{B}), 91.04 (C-4^{A}), 98.46, 99.92, 100.12, 100.60, 100.62, 101.69 (C-1), 128.34 (aromatic C), 128.68 (d, $J_{P,C}$ = 11.4 Hz, aromatic C), 129.01 (d, $J_{P,C}$ = 10.8 Hz, aromatic C), 131.14 (d, $J_{P,C}$ = 16.4.4 Hz, aromatic C), 131.21 ($^{1}J_{P,C}$ = 53.8 Hz, quat. aromatic C), 131.44 (d, $J_{P,C}$ = 9.5 Hz, aromatic C), 132.23 ($^{1}J_{P,C}$ = 57.3 Hz, quat. aromatic C), 132.43 (d, $J_{P,C}$ = 11.4 Hz, aromatic C), 132.61 (d, $J_{P,C}$ = 14.6 Hz, aromatic C), 132.97 (d, $J_{P,C}$ = 6.3 Hz, aromatic C), 134.15 (d, $J_{P,C}$ = 10.0 Hz, aromatic C), 134.24 (d, $J_{P,C}$ = 10.0 Hz, aromatic C), 141.66 (dd, $^{1}J_{P,C}$ = 48.7 Hz, $^{2}J_{P,C}$ = 37.2 Hz, quat. aromatic C), 147.38 (dd, $^{1}J_{P,C}$ = 46.0 Hz, $^{2}J_{P,C}$ = 39.4 Hz, quat. aromatic C) ppm; ^{31}P{^{1}H} NMR (202 MHz, C_6D_6, 25°C): δ = 54.83 (d, $^{2}J_{P,P}$ = 57.9 Hz), 65.22 (d, $^{2}J_{P,P}$ = 57.9 Hz) ppm; elemental analysis (%) calcd for $C_{70}H_{104}O_{28}P_2NiBr_2$ • 1.3 C_5H_{12}: C 51.98, H 6.82, found: C 51.92, H 6.93; MS (ESI-TOF) for $C_{70}H_{104}O_{28}P_2NiBr_2$: *m/z* (%): 1591.47 (100) [*M* - Br]$^+$, 1695.18 (15) [*M* + Na]$^+$.

Dibromido[{6^{A},6^{B}-dideoxy-6^{A},6^{B}-[(1*R*,2*S*)-1,2-phenylenebis(phenylphosphanyl)]-2^{A},2^{B},2^{C},2^{D},2^{E},2^{F},2^{G},3^{A},3^{B},3^{C},3^{D},3^{E},3^{F}, 3^{G},6^{C},6^{D},6^{E},6^{F},6^{G}-nonadeca-*O*-methyl-*β*-cyclodextrin}-*κ*^{2}-*P*,*P*]nickel(II) (complex [$NiBr_2$(**L^{2}**)])

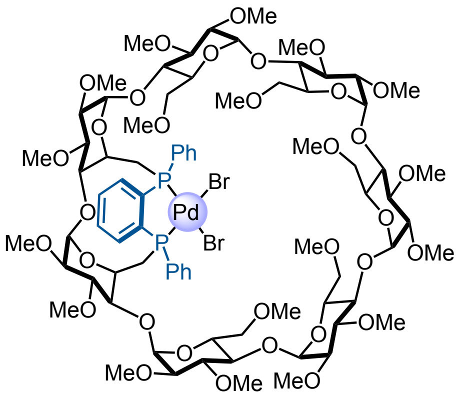

A solution of [$NiBr_2$(DME)] (0.062 g, 0.17 mmol) in CH_2Cl_2 (3 mL) was added to a solution of a mixture of **L^{2}** and two of its diastereomers (0.258 g, 0.17 mmol) obtained via a previously described procedure[2] in CH_2Cl_2 (3 mL). The reaction mixture was stirred for 2 h at room temperature and then evaporated to dryness under reduced pressure. The resulting orange residue was subjected to column

chromatography (SiO_2, CH_2Cl_2/MeOH, 98/2 to 95/5, *v/v)*. The solution was dried under vacuum and the resulting solid was redissolved with MeOH and reacted with LiBr for half an hour before adding water (20 mL). The reaction mixture was extracted with CH_2Cl_2 (3 x 25 mL) and the organic extract dried over $MgSO_4$. The solvent in organic layer was removed and afford pure **7** (0.130 g, 41 %) as an orange solid. A crystalline material was obtained by slow diffusion of *n*-pentane into a CH_2Cl_2 solution of [$NiBr_2$(**L²**)].

^{1}H NMR (500 MHz, C_6D_6, 25 °C): δ (partial assignment by combined COSY, ROESY, TOCSY and HSQC) = 2.48 (m, 1H, H-6a$^{A\text{ or }B}$), 2.53 (m, 1H, H-6a$^{B\text{ or }A}$), 2.65 (m, 1H, H-6b$^{A\text{ or }B}$), 3.06 (s, 3H, OMe), 3.27 (s, 3H, OMe), 3.28 (s, 3H, OMe), 3.36 (s, 3H, OMe), 3.38 (s, 3H, OMe), 3.41 (s, 3H, OMe), 3.43 (s, 3H, OMe), 3.44 (s, 3H, OMe), 3.45 (s, 3H, OMe), 3.50 (s, 3H, OMe), 3.56 (s, 3H, OMe), 3.57 (s, 3H, OMe), 3.59 (s, 3H, OMe), 3.68 (s, 3H, OMe), 3.70 (s, 3H, OMe), 3.73 (s, 3H, OMe), 3.74 (s, 3H, OMe), 3.80 (s, 3H, OMe), 3.81 (s, 3H, OMe), 2.81-4.19 (32H, H-2, H-3, H-4, H-5, H-6), 4.38 (m, 1H, H-5), 4.42-4.48 (2H, H-6), 4.56 (m, 1H, H-6), 4.69 (m, 1H, H-5), 4.76 (m, 1H, H-5), 5.33 (d, 1H, $^3J_{H\text{-}2,\,H\text{-}1}$ = 3.7 Hz, H-1), 5.36 (d, 1H, $^3J_{H\text{-}2,\,H\text{-}1}$ = 3.6 Hz, H-1), 5.41 (d, 1H, $^3J_{H\text{-}2,\,H\text{-}1}$ = 3.6 Hz, H-1), 5.47-5.50 (4H, H-1), 5.63 (2H, m, H-5^A, H-5^B), 6.69-7.34 (10H, aromatic H), 8.03 (m, 2H, aromatic H), 8.17 (m, 2H, aromatic H) ppm; ^{13}C{^{1}H} NMR (126 MHz, CD_2Cl_2, 25°C): δ (partial assignment by HSQC) = 33.33 (d, $^1J_{P,C}$ = 37.2 Hz, C-6$^{A\text{ or }B}$), 34.68 (d, $^1J_{P,C}$ = 28.5 Hz, C-6$^{B\text{ or }A}$), 57.15, 57.47, 58.14, 58.30, 58.55, 58.64, 58.95, 59.13, 59.18, 59.46, 60.02, 60.13, 62.21, 61.38, 61.46, 61.89, 62.03, 62.07, 62.09 (OMe), 71.15 [x2], 71.34, 71.61, 71.71, 71.79, 72.10 (C-5), 72.07, 71.16 [x2], 72.26, 72.87 (C-6^{C,D,E,F,G}), 79.77, 80.03, 81.14, 81.62, 81.76, 81.88, 81.99, 82.02, 82.37, 82.41, 82.45, 82.58, 82.72, 83.01, 83.43, 83.50 [x2], 83.83, 84.03, 86.07 (d, $J_{P,C}$ = 13.3 Hz), 90.98 (br d) (C-2, C-3, C-4), 99.10 [x2], 99.23, 100.22, 100.50, 101.40, 101.97 (C-1), 128.63 (aromatic C), 128.92 (d, $J_{P,C}$ = 10.8 Hz, aromatic C), 130.42 (d, $J_{P,C}$ = 16.1 Hz, aromatic C), 131.07, 131.20, 131.31, 131.89 (d, $^1J_{P,C}$ = 56.7 Hz, quat. aromatic C), 131.97, 132.17 (aromatic C), 132.97 (d, $J_{P,C}$ = 9.6 Hz, aromatic C), 133.92 (d, $J_{P,C}$ = 10.4 Hz, aromatic C), 135.07 (d, $^1J_{P,C}$ = 64.2 Hz, quat. aromatic C), 141.67, 145.61 (m, quat. aromatic C) ppm; ^{31}P{^{1}H} NMR (121 MHz, C_6D_6, 25°C): δ = 64.8 (d, $^2J_{P,P}$ = 57.5 Hz), 69.7 (d, $^2J_{P,P}$ = 57.5 Hz) ppm; elemental analysis (%) calcd for $C_{79}H_{120}Br_2NiO_{33}P_2 \cdot 6\,H_2O$: C 47.77, H 6.70, found: C 47.71, H 6.45; MS (ESI-TOF) for $C_{79}H_{120}Br_2NiO_{33}P_2$: *m/z* (%): 1811.60 (100) [*M* - Br + O]$^+$, 1827.57 (40) [*M* -Br + 2O]$^+$.

Dichlorido[{6^A,6^B-dideoxy-6^A,6^B-[(*R*)-2-(diphenylphosphanyl)phenylphosphinidene]-2^A,2^B,2^C,2^D,2^E,2^F,2^G,3^A,3^B,3^C,3^D,3^E,3^F,3^G,6^C,6^D,6^E,6^F,6^G-nonadeca-*O*-methyl-*β*-cyclodextrin}-*κ*^2-*P*,*P*]nickel(II) (complex [$NiCl_2$(**L**^{4})])

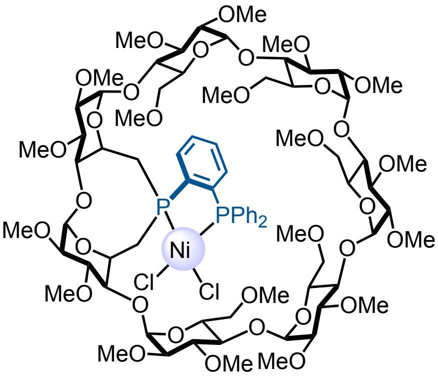

A solution of LDA in THF/heptane/ethyl benzene (2.0 M, 0.38 mL, 0.77 mmol) was added dropwise to a stirred solution of H_2PAr (**3**) (0.091 g, 0.31 mmol) in THF (6 mL) at –78°C. The red solution was stirred at –78 °C for 10 min before being allowed to reach 0 °C over 20 min. The resulting red suspension maintained at 0 °C was cannulated within 5 min into a stirred solution of dimesylate **2** (0.4 g, 0.26 mmol) in THF (10 mL) at 0 °C. The reaction mixture was stirred for 12 h at room temperature. The solvent was then removed *in vacuo* and excess $LiHPArPPh_2$ was protonated with MeOH (5 mL). Removal of the solvent *in vacuo*, afforded a colourless solid, which was subjected to column chromatography (SiO_2, CH_2Cl_2/MeOH, 97:3, *v*/*v*) to afford the mixture of **L**^{4} and its diastereomer in a 2:1 ratio together with residual dimesylate **2** (206 mg). A solution of [$NiCl_2$(DME)] (0.027 g, 0.12 mmol) in CH_2Cl_2 (5 mL) was added to a solution of the two diastereomers and dimesylate **2** (0.206 g) in CH_2Cl_2 (8 mL). The reaction mixture was stirred for 2 h at room temperature and then evaporated to dryness under reduced pressure. The resulting orange residue was subjected to column chromatography (SiO_2, CH_2Cl_2/MeOH, 97/3, *v*/*v*) to afford pure complex [$NiCl_2$(**L**^{4})]) (yield: 73 mg, 40.8 mmol, 16 %) as an orange solid. ^{1}H NMR (500 MHz, CD_2Cl_2, 25 °C): δ (partial assignment by combined COSY, ROESY and HSQC) = 2.12 (m, 1H, H-6a^B), 2.23 (m, 1H, H-6a^A), 2.96 (s, 3H, OMe), 3.26 (s, 3H, OMe), 3.28 (s, 3H, OMe), 3.31(s, 3H, OMe), 3.32 (s, 3H, OMe), 3.43 (s, 3H, OMe), 3.45 (s, 3H, OMe), 3.46 (s, 3H, OMe), 3.47 (s, 3H, OMe), 3.49 (s, 3H, OMe), 3.50 (s, 3H, OMe), 3.52 (s, 3H, OMe), 3.56 (s, 3H, OMe), 3.58 (s, 3H, OMe), 3.59 (s, 3H, OMe), 3.60 (s, 3H, OMe), 3.62 (s, 3H, OMe), 3.64 (s, 3H, OMe), 3.67 (s, 3H, OMe), 3.04-3.97 (35H, H-2, H-3, H-4, H-5, H-6), 4.17 (m, 1H, H-6b^B), 4.20 (dd, 1H,$^3J_{H\text{-}6a,H\text{-}6b}$ = 11.9 Hz, $^3J_{H\text{-}6,H\text{-}5}$ = 1.5 Hz, H-6), 4.39 (dd, 1H,$^3J_{H\text{-}6a,H\text{-}6b}$ = 11.8 Hz, $^3J_{H\text{-}6,H\text{-}5}$ = 1.8 Hz, H-6), 4.59 (m, 1H, H-5^B), 4.89 (d, 1H, $^3J_{H\text{-}1,H\text{-}2}$ = 3.7 Hz, H-1), 4.99 (d, 1H, $^3J_{H\text{-}1,H\text{-}2}$ = 3.4 Hz, H-1), 5.05 (d, 1H, $^3J_{H\text{-}1,H\text{-}}$

$_2$ = 3.0 Hz, H-1), 5.07 (d, 1H, $^3J_{H-1,H-2}$ = 3.4 Hz, H-1), 5.09 (d, 1H, $^3J_{H-1,H-2}$ = 4.08 Hz, H-1), 5.14 (d, 1H, $^3J_{H-1,H-2}$ = 4.3 Hz, H-1), 5.32 (m, 1H, H-5^A), 5.38 (d, 1H, $^3J_{H-1,H-2}$ = 3.9 Hz, H-1), 7.29-7.90 (14H, H_{arom}) ppm; $^{13}C\{^1H\}$ NMR (126 MHz, CD_2Cl_2, 25 °C): δ(partial assignment by HSQC) = 33.22 (d, $^1J_{P,C}$ = 30.4 Hz, C-6^B), 42.12 (d, $^1J_{P,C}$ = 26.2 Hz, C-6^A, 57.86, 57.90, 58.45, 58.73, 58.88 [x2], 59.12, 59.14, 59.22, 59.40, 59.53, 59.57, 60.93, 61.13, 61.56, 61.66, 61.72, 61.81, 62.32 (OMe), 66.88 (d, $^2J_{P,C}$ = 8.5 Hz, C-5$_B$), 68.67 (C-5$_A$), 71.36, 71.45, 71.49, 71.64, 71.98 (C-6^{C,D,E,F,G}), 70.94, 71.16, 71.41 [x2], 71.51 [x2], 75.15, 79.14, 79.95, 80.61, 81.34, 81.50, 82.16, 82.21, 82.25, 82.29 [x2], 82.42, 82.49, 82.54, 82.89, 83.09, 83.10, 83.57 (C-2, C-3, C-4^{C,D,E,F,G}, C-5^{C,D,E,F,G}), 86.87 (d, $^3J_{P,C}$ = 8.3 Hz, C-4^B), 90.77 (d, $^3J_{P,C}$ = 3.2 Hz, C-4^A), 98.10, 99.48, 99.50, 99.25, 99.33, 99.82, 99.88 (C-1), 129.04 (d, $J_{P,C}$ = 11.1 Hz, aromatic C), 129.13 (d, $J_{P,C}$ = 11.1 Hz, aromatic C), 129.86 ($^1J_{P,C}$ = 52.9 Hz, quat. aromatic C), 131.23 ($^1J_{P,C}$ = 55.4 Hz, quat. aromatic C), 131.51 (d, $J_{P,C}$ = 13.8 Hz, aromatic C), 131.82, 131.85 (aromatic C) 132.78 (d, $J_{P,C}$ = 15.0 Hz, aromatic C), 133.02 (d, $J_{P,C}$ = 6.0 Hz, aromatic C), 133.73 (d, $J_{P,C}$ = 5.8 Hz, aromatic C), 133.96 (d, $J_{P,C}$ = 9.8 Hz, aromatic C), 134.30 (d, $J_{P,C}$ = 10.7 Hz, aromatic C), 140.19 (dd, $^1J_{P,C}$ = 52.3 Hz, $^2J_{P,C}$ = 36.3 Hz, quat. aromatic C), 145.93 (dd, $^1J_{P,C}$ = 48.5 Hz, $^2J_{P,C}$ = 38.4 Hz, quat. aromatic C) ppm; $^{31}P\{^1H\}$ NMR (121 MHz, CD_2Cl_2 25 ℃): δ = 48.2 (d, $^2J_{P,P}$ = 70.7 Hz), 56.1 (d, $^2J_{P,P}$ = 13.6 Hz) ppm; HR-MS (ESI-TOF) for $C_{79}H_{120}Cl_2NiO_{33}P_2$: *m/z* (%): 1753.6214 (100) [*M* - Cl]$^+$; calcd m/z = 1753.6225 [M - Cl]$^+$; error = 1.1 ppm.

Dibromido-[{[2-(diisopropylphosphanyl)phenyl]diphenylphosphane}-κ^2-*P*,*P*]nickel(II) (complex [$NiBr_2$(**L^5**)])

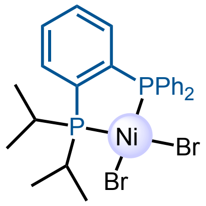

[$NiBr_2$(DME)] (63 mg, 0.20 mmol) was added to a solution of [2-(diisopropylphosphanyl)phenyl]diphenylphosphane[4] (**L^5**) (75 mg, 0.20 mmol) in CH_2Cl_2 (8 mL). The mixture was stirred at room temperature for 4 h and then filtered through Celite. Concentration under vacuum gave the crude filtrate which was dried under reduced pressure to afford [$NiBr_2$(**L^5**)] as an orange powder (118 mg, 100 %). ^1H NMR (500 MHz, CD_2Cl_2, 25 °C) : δ = 7.85-7.72 (m, 5H), 7.68-7.54 (m, 2H), 7.58-7.50 (m, 2H), 7.48-7.38 (m, 5H), 2.97 (m, 2H), 1.52 (d, *J* = 7.0 Hz, 3H), 1.48 (d, *J* = 7.0 Hz, 3H), 1.37 (d, *J* = 7.1 Hz, 3H), 1.34 (d, *J* = 7.1 Hz, 3H); $^{13}C\{^1H\}$ NMR (126 MHz, CD_2Cl_2, 25 °C): δ = 144.38 (dd, *J* = 51.2, 32.5 Hz), 138.46 (t, *J* = 38.7 Hz),

134.47 (d, J = 10.1 Hz), 133.79 (dd, J = 14.0, 1.8 Hz), 133.28 (dd, J = 6.2, 1.9 Hz), 132.65 (dd, J = 5.2, 2.0 Hz), 132.02, 131.88, 131.75 (d, J = 2.9 Hz), 131.14, 130.70, 128.91 (d, J = 11.1 Hz), 28.95 (d, J = 27.4 Hz), 19.94 (d, J = 1.8 Hz), 18.85 (d, J = 2.0 Hz). $^{31}P\{^1H\}$ NMR (121 MHz, CD_2Cl_2, 25 °C): δ = 91.44 (d, $^3J_{P,P}$ = 59.9 Hz), 63.36 (d, $^3J_{P,P}$ = 59.9 Hz) ppm; elemental analysis (%) calcd for $C_{24}H_{28}Br_2NiP_2$: C 48.29, H 4.73, found: C 48.00, H 4.65; HRMS (ESI-TOF): *m/z* (%): 515.0198 (100) [M−Br]$^+$.

Dichlorido-[{6^A,6^B-dideoxy-6^A,6^B-[(1*R*,2*S*)-1,2-phenylenebis(phenylphosphanyl)]-2^A,2^B,2^C,2^D,2^E,2^F,3^A,3^B,3^C,3^D,3^E,3^F,6^C,6^D,6^E,6^F-hexadeca-*O*-methyl-*α*-cyclodextrin}-*κ*^{2}-*P*,*P*]palladium(II) ([$PdCl_2$(**L^1**)])

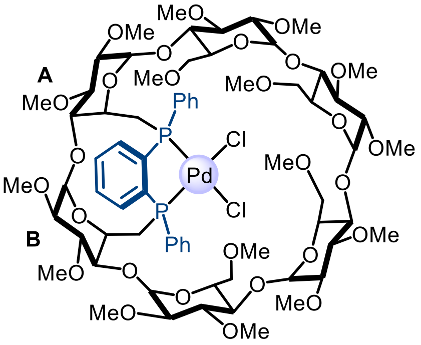

A solution of [$PdCl_2$(COD)] (0.04 g, 0.16 mmol) in CH_2Cl_2 (5 mL) was added to a solution of **L^1** (0.228 g, 0.16 mmol) in CH_2Cl_2 (5 mL). The reaction mixture was stirred for 2 h at room temperature and then evaporated to dryness under reduced pressure. The resulting yellow residue was subjected to column chromatography (SiO_2, CH_2Cl_2/MeOH, 97/3, *v/v*) to afford pure [$PdCl_2$(**L^1**)] (0.198 g, 77%) as a yellow solid. A crystalline material was obtained by slow diffusion of *n*-pentane into a dichloromethane solution of [$PdCl_2$(**L^1**)]. ^1H NMR (500 MHz, $CDCl_3$, 25 °C): δ (partial by combined COSY, ROESY, TOCSY and HSQC) = 2.53 (m, 1H, H-6a^A), 2.58 (m, 1H, H-6a^B), 2.65 (m, 1H, H-6b^A), 2.99 (m, 1H, H-2^B), 3.01 (m, 1H, H-4^A), 3.15 (s, 3H, OMe), 3.29 (s, 3H, OMe), 3.31 (s, 3H, OMe), 3.34 (s, 3H, OMe), 3.40 (s, 3H, OMe), 3.43 (s, 3H, OMe), 3.45 (s, 3H, OMe), 3.49 (s, 3H, OMe), 3.51 (s, 3H, OMe), 3.54 (s, 3H, OMe), 3.60 (s, 3H, OMe), 3.60 (s, 3H, OMe), 3.63 (s, 3H, OMe), 3.63 (s, 3H, OMe), 3.68 (s, 3H, OMe), 3.69 (s, 3H, OMe), 3.08-4.03 (27H, H-2^{A,C,D,E,F}, H-3^{A,B,C,D,E,F}, H-4^{B,C,D,E,F}, H-5^{D,E}, H-6b^B, H-6^{C,D,E,F}), 4.30 (m, 1H, H-5^F), 4.40 (m, 1H, H-5^C), 4.58 (d, $^3J_{H-2,H-1}$ = 3.2 Hz, 1H, H-1), 4.75 (m, 1H, H-5^B), 4.84 (m, 1H, H-5^A), 4.97 (d, $^3J_{H-2,H-1}$ = 3.2 Hz, 1H, H-1), 5.06-5.09 (2H, H-1), 5.10 (d, $^3J_{H-2,H-1}$ = 3.1 Hz, 1H, H-1), 5.14 (d, $^3J_{H-2,H-1}$ = 3.2 Hz, 1H, H-1), 7.07 (m, 1H, aromatic H), 7.41-7.59 (9H, aromatic H), 7.82 (m, 2H, aromatic H), 7.91 (m, 2H, aromatic H) ; $^{13}C\{^1H\}$ NMR (126 MHz, $CDCl_3$, 25 °C): δ (partial assignment by HSQC) = 31.74 (d, $^1J_{P,C}$ = 34.2 Hz, C-6^B), 36.77 (d, $^1J_{P,C}$ = 31.7 Hz, C-6^A), 57.46, 57.59, 58.02, 58.09, 58.16, 58.21, 58.99, 59.08, 59.16, 59.41, 61.27, 61.90, 62.03, 62.05, 62.08, 62.18 (OMe), 71.26, 71.64,

71.70, 72.10 (C-6^{C,D,E,F}), 71.03, 71.15, 71.22, 71.83, 72.14, 72.57 (C-5^{A,B,C,D,E,F}) 77.30, 79.51 [x2], 79.86, 80.69, 81.19, 81.36, 81.63, 81.98 [x2], 82.08, 82.22 [x2], 82.53, 82.93, 82.94 (C-2^{A,B,C,D,E,F}, C-3^{A,B,C,D,E,F}, C-4^{C,D,E,F}), 89.72 (d, $^3J_{P,C}$ = 13.5 Hz, C-4^B), 91.33 (d, $^3J_{P,C}$ = 13.3 Hz C-4^A), 99.74, 100.12, 100.43, 100.52, 101.34, 102.15 (C-1), 128.56 (d, $^1J_{P,C}$ = 57.0 Hz, quat. aromatic C), 128.82 (d, $J_{P,C}$ = 12.1 Hz, aromatic C), 130.90, 131.07 (aromatic C), 131.29 (d, $^1J_{P,C}$ = 61 Hz, quat. aromatic C), 131.71, 131.95, 132.06 (aromatic C), 132.64 (d, $J_{P,C}$ = 8.8 Hz, aromatic C), 132.72 (d, $J_{P,C}$ = 10.7 Hz, aromatic C), 132.97 (d, $J_{P,C}$ = 5.2 Hz, aromatic C), 133.43 (d, $J_{P,C}$ = 11.6 Hz, aromatic C), 140.77 (dd, $^1J_{P,C}$ = 50.4 Hz, $^2J_{P,C}$ = 36.5 Hz, quat. aromatic C), 144.28 (dd, $^1J_{P,C}$ = 55.6 Hz, $^2J_{P,C}$ = 37.7 Hz, quat. aromatic C) ; $^{31}P\{^1H\}$ NMR (202 MHz, $CDCl_3$, 25°C): δ = 62.4 (d, $^2J_{P,P}$ = 9.3 Hz), 69.0 (d, $^2J_{P,P}$ = 9.3 Hz) ppm ; Elemental analysis (%) calcd for $C_{70}H_{104}Cl_2O_{28}P_2Pd$: C 51.49, H 6.42, found: C 51.49, H 6.42 ; MS (ESI-TOF) for $C_{70}H_{104}Cl_2O_{28}P_2Pd$: *m/z* (%): 1597.49 (15) [M - Cl]$^+$, 1655.45 (100) [*M* + Na]$^+$.

Dichlorido-[{6^A,6^B-dideoxy-6^A,6^B-[(*R*)-2-(diphenylphosphanyl)phenyl phosphinidene]-2^A,2^B,2^C,2^D,2^E,2^F,3^A,3^B,3^C,3^D,3^E,3^F,6^C,6^D,6^E,6^F-hexadeca-*O*-methyl-α-cyclodextrin}-κ^2-*P*,*P*]palladium(II) (complex [$PdCl_2$(**L**^{3})])

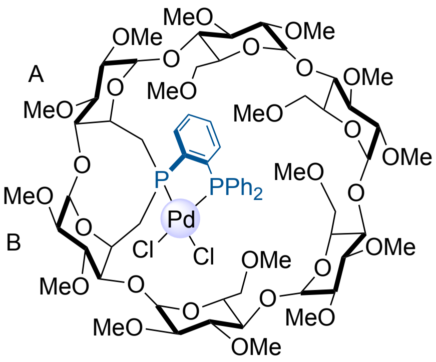

A solution of [$PdCl_2$(COD)] (0.098 g, 0.34 mmol) in CH_2Cl_2 (6 mL) was added to a solution of **2** (0.5 g, 0.34 mmol) in CH_2Cl_2 (6 mL). The reaction mixture was stirred for 2 h at room temperature and then evaporated to dryness under reduced pressure. The resulting yellow residue was subjected to column chromatography (SiO_2, CH_2Cl_2/MeOH, 97/3, *v/v*) to afford pure [$PdCl_2$(**L**^{3})]) (0.42 g, 75 %) as a yellow solid. A crystalline material was obtained by slow diffusion of *n*-pentane into a benzene solution of [$PdCl_2$(**L**^{3})]. ^1H NMR (500 MHz, $CDCl_3$, 25 °C): δ (partial assignment by combined COSY, ROESY, TOCSY and HSQC) = 2.15 (m, 1H, H-6a^A), 2.34 (m, 1H, H-6a^B), 2.67 (s, 3H, OMe), 3.30 (s, 3H, OMe), 3.36 (s, 3H, OMe), 3.38 (s, 3H, OMe), 3.43 (s, 3H, OMe), 3.45 (s, 3H, OMe), 3.46 (s, 9H, OMe), 3.52 (s, 3H, OMe), 3.61 (s, 3H, OMe), 3.62 (s, 3H, OMe), 3.64 (s, 9H, OMe), 3.69 (s, 3H, OMe), 3.04-4.30 (32H, H-2, H-3, H-4, H-5^{C,D,E,F}, H-6^{C,D,E,F}, H-6b^{A,B}), 4.21 (m, 1H, H-5), 4.30 (m, 1H, H-5), (4.80 (m, 1H, H-5^B), 4.95 (m, 2H, H-1),

5.05 (d, 1H, $^{3}J_{H-2, H-1}$ = 3.5 Hz, H-1), 5.05-5.07 (2H, H-1), 5.16 (d, 1H, $^{3}J_{H-2, H-1}$ = 4.2 Hz, H-1), 5.30 (m, 1H, H-5^{A}), 7.38-7.45 (5H, aromatic H), 7.46-7.53 (2H, aromatic H), 7.62 (m, 1H, aromatic H), 7.67-7.76 (5H, aromatic H), 7.98 (m, 1H, aromatic H) ppm; $^{13}C\{^{1}H\}$ NMR (126 MHz, $CDCl_3$, 25 °C): δ(assignment by HSQC) = 33.57 (d, $^{1}J_{P,C}$ = 30.1 Hz, C-6^{B}), 43.01 (d, $^{1}J_{P,C}$ = 26.5 Hz, C-6^{A}), 57.34, 57.46, 57.51, 57.71, 57.77, 57.87, 58.31, 58.86, 58.98, 59.22, 61.78, 61.83, 61.89, 61.93, 62.20, 62.51 (OMe), 66.07 (d, $^{2}J_{P,C}$ = 7.8 Hz, C-5^{B}), 67.52 (C-5^{A}), 70.05, 70.63, 70.71, 70.81 (C-5^{C,D,E,F}), 71.19, 71.51, 71.69, 72.33 (C-6^{C,D,E,F}), 77.36, 80.20, 81.08, 81.16, 81.19, 81.30, 81.33, 81.64, 82.07 [x2], 82.23, 82.30, 82.39, 82.42 [x2], 82.59, 88.32 (d, J = 9.9 Hz), 90.51 (C-2, C-3, C-4), 98.12, 99.70, 99.78, 99.89, 100.42, 101.48 (C-1), 128.92 aromatic C), 129.18 (d, J = 11.5 Hz, aromatic C), 129.21 (d, $^{1}J_{P,C}$ = 55.4 Hz, quat. aromatic C), 129.86 (d, $^{1}J_{P,C}$ = 60.0 Hz, quat. aromatic C), 131.90 (d, $J_{P,C}$ = 17.6 Hz, aromatic C), 132.04, 132.28 (aromatic C), 133.16 (d, $J_{P,C}$ = 7.0 Hz, aromatic C), 133.42 (d, $J_{P,C}$ = 10.7 Hz, aromatic C), 133.49 (aromatic C), 133.86 (d, $J_{P,C}$ = 7.2 Hz, aromatic C), 134.09 (d, $J_{P,C}$ = 11.8 Hz, aromatic C), 138.92 (dd, $^{1}J_{P,C}$ = 54.3 Hz, $^{2}J_{P,C}$ = 35.8 Hz, quat. aromatic C) 144.55 (dd, $^{1}J_{P,C}$ = 53.1 Hz, $^{2}J_{P,C}$ = 37.4 Hz, quat. aromatic C), ppm; $^{31}P\{^{1}H\}$ NMR (162 MHz, $CDCl_3$, 25 ℃): δ = 55.53 (d, $^{2}J_{P,P}$ = 13.6 Hz), 61.65 (d, $^{2}J_{P,P}$ = 13.6 Hz) ppm; elemental analysis (%) calcd for $C_{70}H_{104}Cl_2O_{28}P_2Pd \cdot 2\ CH_2Cl_2$: C 49.64, H 6.22, found: C 49.63, H 6.33; MS (ESI-TOF) for $C_{70}H_{104}Cl_2O_{28}P_2Pd$: m/z (%): 1597.49 (15) [M -Cl]$^{+}$, 1655.45 (100) [M + Na]$^{+}$.

Dichlorido[{6^{A},6^{B}-dideoxy-6^{A},6^{B}-[(1*R*,2*S*)-1,2-phenylenebis(phenylphosphanyl)]-2^{A},2^{B},2^{C},2^{D},2^{E},2^{F},2^{G},3^{A},3^{B},3^{C},3^{D},3^{E},3^{F},3^{G},6^{C},6^{D},6^{E},6^{F},6^{G}-nonadeca-*O*-methyl-*β*-cyclodextrin}-κ^{2}-*P*,*P*]palladium(II) (complex [$PdCl_2$(**L^{2}**)])

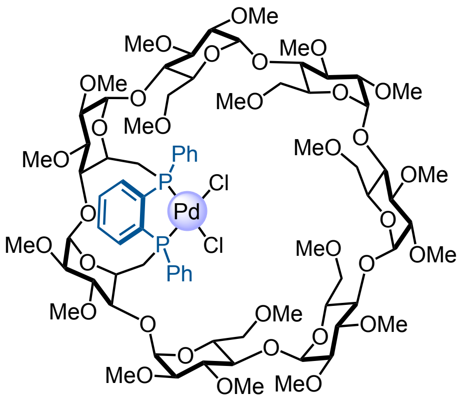

A solution of [$PdCl_2$(COD)] (0.0096 g, 0.036 mmol) in CH_2Cl_2 (3 mL) was added to a solution of **L^{2}** and two of its diastereomers (0.056 g, 0.036 mmol) in CH_2Cl_2 (3 mL) obtained via a previously described procedure.[2] The reaction mixture was stirred for 2 h at room temperature and then evaporated to dryness under reduced pressure. The resulting yellow residue was subjected to column

chromatography (SiO_2, CH_2Cl_2/MeOH, 98/2 to 95/5, *v/v)* to afford pure [$PdCl_2$(**L^2**)]) (0.036 g, 58 %) as a yellow solid.

^{1}H NMR (500 MHz, $CDCl_3$, 25 °C): δ (partial assignment by combined COSY, ROESY, and HSQC) 2.57 (m, 1H, H-6a$^{A\ or\ B}$), 2.69 (m, 1H, H-6b$^{A\ or\ B}$), 2.71 (m, 1H, H-6a$^{B\ or\ A}$), 3.22 (s, 3H, OMe), 3.25 (s, 3H, OMe), 3.30 (s, 6H, OMe), 3.38 (s, 3H, OMe), 3.43 (s, 3H, OMe), 3.44 (s, 3H, OMe), 3.46 (s, 3H, OMe), 3.47 (s, 3H, OMe), 3.49 (s, 3H, OMe), 3.50 (s, 3H, OMe), 3.51 (s, 3H, OMe), 3.56 (s, 3H, OMe), 3.58 (s, 3H, OMe), 3.59 (s, 3H, OMe), 3.60 (s, 3H, OMe), 3.61 (s, 3H, OMe), 3.65 (s, 6H, OMe), 2.92-3.70 (35H, H-2, H-3, H-4, H-5, H-6), 3.77 (m, 1H, H-6), 3.81 (m, 1H, H-6), 3.95 (m, 1H, H-6), 3.98 (m, 1H, H-5), 4.24 (m, 1H, H-5), 4.63 (d, 1H, $^3J_{H-2,\ H-1}$ = 3.2 Hz, H-1), 4.96 (d, 1H, $^3J_{H-2,\ H-1}$ = 3.7 Hz, H-1), 4.96 (m, 1H, H-5$^{B\ or\ A}$), 5.00 (d, 1H, $^3J_{H-2,\ H-1}$ = 3.6 Hz, H-1), 5.04 (d, 1H, $^3J_{H-2,\ H-1}$ = 3.2 Hz, H-1), 5.06 (d, 1H, $^3J_{H-2,\ H-1}$ = 3.4 Hz, H-1), 5.08 (m, 1H, H-5$^{A\ or\ B}$), 5.09 (d, 1H, $^3J_{H-2,\ H-1}$ = 4.1 Hz, H-1), 5.15 (d, 1H, $^3J_{H-2,\ H-1}$ = 3.5 Hz, H-1), 7.16-7.64 (10H, aromatic H), 7.97 (m, 2H, aromatic H), 8.12 (m, 2H, aromatic H) ppm, ^{13}C{^{1}H} NMR (126 MHz, $CDCl_3$, 25°C): δ (partial assignment by HSQC) = 33.03 (d, $^{1J}{}_{P,C}$ = 35.9 Hz, C-6$^{A\ or\ B}$), 34.57 (d, $^{1J}{}_{P,C}$ = 31.8 Hz, C-6$^{B\ or\ A}$), 57.94, 58.06, 58.18, 58.44, 58.62, 57.81, 58.82, 58.96, 59.13, 59.35, 59.66, 59.75, 61.15, 61.36, 61.72, 62.00, 62.14 [x2], 62.37(OMe), 70.20, 70.55, 70.61, 70.71 [x2], 70.90, 72.29 (C-5), 70.57, 71.02 [x2], 71.07 71.87 (C-6^{C,D,E,F,G}), 77.36, 79.36, 79.86, 80.63, 80.77, 80.84, 81.28, 81.38, 81.51, 81.69, 81.79, 81.83 [x2], 82.04, 82.16, 82.38, 82.72, 82.90, 83.16, (C-2, C-3, C-4^{C,D,E,F,G}), 86.52 (d, $^3J_{P,C}$ = 13.8 Hz, C-4$^{B\ or\ A}$), 90.40 (d, $^3J_{P,C}$ = 12.0 Hz, C-4$^{A\ or\ B}$), 98.69, 99.26, 99.40, 99.66, 100.22, 101.58, 101.63 (C-1), 127.26 (d, $^{1J}{}_{P,C}$ = 54.5 Hz, quat. aromatic C), 128.91 (d, $J_{P,C}$ = 11.9 Hz, aromatic C), 129.11 (d, $J_{P,C}$ = 11.5 Hz, aromatic C), 130.54 (d, $J_{P,C}$ = 18.8 Hz, aromatic C), 131.28 (d, $^{1J}{}_{P,C}$ = 62.1 Hz, quat. aromatic C), 131.82, 131.93, 132.26 (aromatic C), 132.78 (d, $J_{P,C}$ = 6.7 Hz, aromatic C), 133.00 (d, $J_{P,C}$ = 6.4 Hz, aromatic C), 133.19 (d, $J_{P,C}$ = 10.8 Hz, aromatic C), 134.15 (d, $J_{P,C}$ = 11.9 Hz, aromatic C), 139.05 (dd, $^1J_{P,C}$ = 51.0 Hz, $^2J_{P,C}$ = 36.5 Hz, quat. aromatic C), 143.74 (dd, $^1J_{P,C}$ = 55.6 Hz, $^2J_{P,C}$ = 37.9 Hz, quat. aromatic C) ppm; ^{31}P{^{1}H} NMR (202 MHz, $CDCl_3$, 25°C): δ = 66.6 (d, $^2J_{P,P}$ = 11.4 Hz), 67.3 (d, $^2J_{P,P}$ = 11.4 Hz) ppm ; elemental analysis (%) calcd for $C_{79}H_{120}Cl_2O_{33}P_2Pd$ • 2 CH_2Cl_2: C 49.64, H 6.22, found: C 49.63, H 6.33; MS (ESI-TOF) for $C_{79}H_{120}Cl_2O_{33}P_2Pd$: *m/z* (%): 941.28 (25) [M + 2 Na]$^{2+}$, 1859.56 (100) [*M* + Na]$^+$, 1875.53 (30) [*M* + Na]$^+$

Dichloro[{6^A,6^B-dideoxy-6^A,6^B-[*R*)-2-(diphenylphosphanyl)phenylphosphinidene]-2^A,2^B,2^C,2^D,2^E,2^F,2^G,3^A,3^B,3^C,3^D,3^E,3^F,3^G,6^C,6^D,6^E,6^F-nonadeca-*O*-methyl-*β*-cyclodextrin}-*κ*^{2}-*P*,*P*]palladium(II) (complex [$PdCl_2$(**L**^{4})])

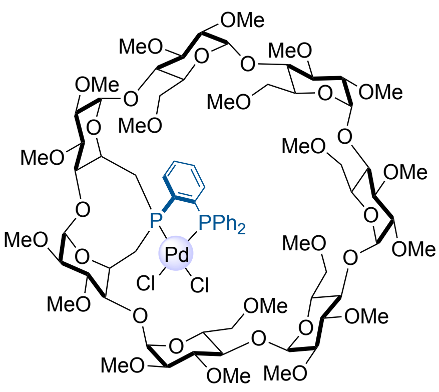

A solution of LDA in THF/heptane/ethyl benzene (2.0 M, 0.19 mL, 0.385 mmol) was added dropwise to a stirred solution of H_2PAr (**3**) (0.0453 g, 0.154 mmol) in THF (3 mL) at –78°C. The red solution was stirred at –78 °C for 10 min before being allowed to reach 0 °C over 20 min. The resulting red suspension maintained at 0 °C was cannulated within 5 min into a stirred solution of dimesylate **2** (0.2 g, 0.128 mmol) in THF (5 mL) at 0 °C. The reaction mixture was stirred for 12 h at room temperature. The solvent was then removed *in vacuo* and excess LiHPArPPh_2 was protonated with MeOH (5 mL). Removal of the solvent *in vacuo*, afforded a colorless solid, which was subjected to column chromatography (SiO_2, CH_2Cl_2/MeOH, 97:3, *v*/*v*) to afford the **L**^{4} and its diastereomer in a 2:1 ratio together with residual dimesylate **2** (96 mg). A solution of [$PdCl_2$(COD)] (0.0165 g, 0.057 mmol) in CH_2Cl_2 (2 mL) was added to a solution of the mixture of diphosphanes and dimesylate **2** in CH_2Cl_2 (4 mL). The reaction mixture was stirred for 12 h at room temperature and then filtrate over celite™ before being evaporated to dryness under reduced pressure. The resulting yellow residue was subjected to column chromatography (SiO_2, CH_2Cl_2/MeOH, 97/3, *v/v*) to afford pure [$PdCl_2$(**L**^{2})] (0.030 g, 13%) as a yellow solid. ^{1}H NMR (500 MHz, CD_2Cl_2, 25 °C): δ (partial assignment by combined COSY, ROESY, TOCSY and HSQC) = 2.22 (m, 1H, H-6a^A), 2.32 (m, 1H, H-6a^B), 2.65 (s, 3H, OMe), 3.34 (s, 3H, OMe), 3.38 (s, 3H, OMe), 3.39 (s, 3H, OMe), 3.41 (s, 3H, OMe), 3.44 (s, 3H, OMe), 3.45 (s, 3H, OMe), 3.46 (s, 3H, OMe), 3.47 (s, 3H, OMe), 3.49 (s, 3H, OMe), 3.51 (s, 3H, OMe), 3.55 (s, 3H, OMe), 3.58 (s, 3H, OMe), 3.60 (s, 3H, OMe), 3.61 (s, 3H, OMe), 3.64 (s, 3H, OMe), 3.65 (s, 3H, OMe), 3.68 (s, 3H, OMe), 3.72 (s, 3H, OMe), 3.01-3.85 (32H, H-2, H-3, H-4, H-5, H-6), 3.89 (m, 1H, H-6), 3.94 (m, 1, H-5), 3.98 (m, 1H, H-6), 4.05 (m, 1H, H-5), 4.12 (m, 1H, H-6), 4.19 (m, 1H, H-6b^B), 4.83 (m, 1H, H-5^B), 4.87 (d, 1H, $^3J_{H\text{-}1,H\text{-}2}$ = 3.7 Hz, H-1), 4.95 (d, 1H, $^3J_{H\text{-}1,H\text{-}2}$ = 3.1 Hz, H-1), 5.06 (d, 1H, $^3J_{H\text{-}1,H\text{-}2}$ = 3.6 Hz, H-1), 5.08, (d, 1H, $^3J_{H\text{-}1,H\text{-}2}$ = 3.4 Hz, H-1), 5.15 (d, 1H, $^3J_{H\text{-}1,H\text{-}2}$ = 4.4 Hz, H-1), 5.24 (d, 1H, $^3J_{H\text{-}1,H\text{-}2}$ = 4.1 Hz, H-1), 5.25 (d, 1H, $^3J_{H\text{-}1,H\text{-}2}$ = 4.1 Hz, H-1), 5.34 (m, 1H, H-5^A), 7.96 (m, 1H,

H_{arom}), 7.73-7.80 (3H, H_{arom}), 7.60-7.64 (3H, H_{arom}), 7.37-7.53 (7H, H_{arom}) ppm; $^{13}C\{^1H\}$ NMR (126 MHz, CD_2Cl_2, 25°C): δ (partial assignment by HSQC) = 33.58 (d, $^1J_{P,C}$ = 30.4 Hz, C-6^B), 43.98 (d, $^1J_{P,C}$ = 26.6 Hz, C-6^A), 57.86, 58.10, 58.24, 58.55, 58.60, 58.66, 58.69, 58.80, 59.24, 59.27, 59.30, 59.36, 61.11, 61.66, 61.67, 61.79, 61.87, 61.89, 62.61 (OMe), 66.65 (d, $^3J_{P,C}$ = 8.0 Hz, C-5^B), 68.70 (C-5^A), 70. 56, 70. 94, 71.00, 71.23, 71.34 (C-$5^{C,D,E,F,G}$), 71.00, 71.09 [x2], 71.62, 71.88 (C-$6^{C,D,E,F,G}$), 77.34, 77.94, 78.04, 79.69, 80.64, 80.93, 81.28, 81.50, 81.82, 82.06 [x2], 82.19 [x2], 82.33, 82.39, 82.54, 82.61, 82.71, 82.96 (C-2, C-3, C-$4^{C,D,E,F,G}$), 87.42 (d, $^3J_{P,C}$ = 9.4 Hz, C-4^B), 91.21 (C-4^A), 98.35, 99.71, 98.85, 99.11, 99.82, 100.54, 100.65 (C-1), 129.10 (d, $J_{P,C}$ = 5.2 Hz, aromatic C), 129.19 (d, $J_{P,C}$ = 4.4 Hz, aromatic C), 129.24 ($^1J_{P,C}$ = 52.9 Hz, quat. aromatic C), 129.86 ($^1J_{P,C}$ = 59.5 Hz, quat. aromatic C), 131.97 (aromatic C), 131.98 (d, $J_{P,C}$ = 16.4 Hz, aromatic C), 132.31 (aromatic C), 133.16 (d, $J_{P,C}$ = 6.7 Hz, aromatic C), 133.45 (d, $J_{P,C}$ = 10.5 Hz, aromatic C), 133.59 (aromatic C), 133.88 (d, $J_{P,C}$ = 6.5 Hz, aromatic C), 134.40 (d, $J_{P,C}$ = 12.0 Hz, aromatic C), 139.23 (dd, $^1J_{P,C}$ = 54.3 Hz, $^2J_{P,C}$ = 36.1 Hz, quat. aromatic C), 145.93 (dd, $^1J_{P,C}$ = 52.1 Hz, $^2J_{P,C}$ = 37.7 Hz, quat. aromatic C) ppm; $^{31}P\{^1H\}$ NMR (202 MHz, $CDCl_3$ 25 ℃): δ = 57.0 (d, $^2J_{P,P}$ = 16.3 Hz), 62.0 (d, $^2J_{P,P}$ = 16.3 Hz) ppm; elemental analysis (%) calcd for $C_{79}H_{120}Cl_2O_{33}P_2Pd$ • CH_2Cl_2: C 49.99, H 6.40, found: C 49.99, H 6.44; MS (ESI-TOF) for $C_{79}H_{120}Cl_2O_{33}P_2Pd$: *m/z* (%): 941.27 (25) [M + 2 Na]$^{2+}$, 1859.56 (100) [*M* + Na]$^+$, 1875.52 (30) [*M* + Na]$^+$

Methylchlorido-[{6^A,6^B-dideoxy-6^A,6^B-[(*R*)-2-(diphenylphosphanyl)phenyl phosphinidene]-2^A,2^B,2^C,2^D,2^E,2^F,3^A,3^B,3^C,3^D,3^E,3^F,6^C,6^D,6^E,6^F-hexadeca-*O*-methyl-α-cyclodextrin}-κ^2-*P,P*]palladium(II) (Isomers **6a** and **6b)**

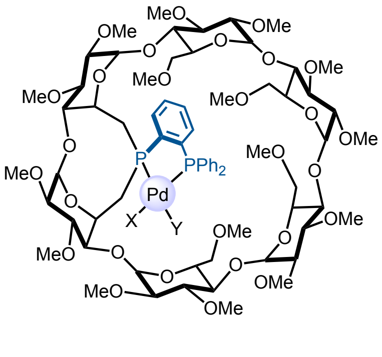

6a, X = Me and Y = Cl
6b, X = Cl and Y = Me

A solution of [PdMeCl(COD)] (0.025 g, 0.096 mmol) in THF (1 mL) was added to a solution of **2** (0.14 g, 0.096 mmol) in THF (1 mL). The reaction mixture was stirred for 2 h at room temperature and then evaporated to dryness under reduced pressure. The resulting yellow residue was subjected to column chromatography (SiO_2, CH_2Cl_2/MeOH, 97/3, *v/v*) to afford the product as a yellow solid (0.124 g, 80 %). The ^1H NMR spectrum shows two isomers in a ca. 25/75 ratio of **6a**/**6b** in C_6D_6 at room temperature. ^1H NMR (500 MHz, C_6D_6, 25 ℃): δ (partial assignment by combined COSY, ROESY, TOCSY and HSQC)

= 1.13 (dd, 3H, $^3J_{H,P}$ = 7.3 Hz, $^3J_{H,P}$ = 2.5 Hz, PdMe, **6a**), 1.23 (dd, 3H, $^3J_{H,P}$ = 8.7 Hz, $^3J_{H,P}$ = 3.2 Hz, PdMe, **6b**), 2.06 (m, 1H, H-6a$^{A\ or\ B}$, **6b**), 2.11 (m, 1H, H-6a$^{A\ or\ B}$, **6a**), 2.13 (m, 1H, H-6a$^{B\ or\ A}$, **6b**), 2.27 (m, 1H, H-6a$^{B\ or\ A}$, **6b**), 3.14 (s, 3H, OMe, **6b**), 3.25 (s, 3H, OMe, **6b**), 3.30 (s, 3H, OMe, **6b**), 3.32 (s, 3H, OMe, **6b**), 3.34 (s, 6H, OMe, **6b**), 3.39 (m, 1H, H-6b$^{A\ or\ B}$, **6a**), 3.41 (s, 3H, OMe, **6b**), 3.48 (s, 3H, OMe, **6b**), 3.49 (m, 1H, H-6b$^{A\ or\ B}$, **6b**), 3.50 (s, 3H, OMe, **6b**), 3.51 (s, 3H, OMe, **6b**), 3.61 (m, 1H, H-6a$^{B\ or\ A}$, **6a**), 3.67 (s, 3H, OMe, **6b**), 3.69 (s, 3H, OMe, **6b**), 3.84 (s, 6H, OMe, **6b**), 3.87 (s, 3H, OMe, **6b**), 3.89 (s, 3H, OMe, **6b**), 3.91 (m, 1H, H-6a$^{B\ or\ A}$, **6b**), 2.92-4.96 (H-2, H-3, H-4, H-5^{C,D,E,F}, H-6 ^{C,D,E,F} of **6b** and H-2, H-3, H-4, H-5^{C,D,E,F}, H-6^{C,D,E,F}, OMe of **6a**), 4.45 (m, 1H, H-5$^{B\ or\ A}$, **6a**), 4.63 (m, 1H, H-5$^{B\ or\ A}$, **6b**), 4.77 (m, 1H, H-5$^{A\ or\ B}$, **6a**), 4.79 (d, 1H, $^3J_{H\text{-}1,H\text{-}2}$ = 3.3 Hz, H-1, **6b**), 4.84 (d, 1H, $^3J_{H\text{-}1,H\text{-}2}$ = 4.0 Hz, H-1, **6b**), 5.30 (d, 1H, $^3J_{H\text{-}1,H\text{-}2}$ = 3.4 Hz, H-1, **6b**), 5.33 (d, 1H, $^3J_{H\text{-}1,H\text{-}2}$ = 3.5 Hz, H-1, **6b**), 5.33 (m, 1H, H-5$^{A\ or\ B}$, **6b**), 5.35 (d, 1H, $^3J_{H\text{-}1,H\text{-}2}$ = 3.2 Hz, H-1, **6b**), 5.38 (d, 1H, $^3J_{H\text{-}1,H\text{-}2}$ = 3.0 Hz, H-1, **6b**), 5.26-5.40 (6H, H-1, **6a**), 6.91-7.95 (aromatic H) ppm; $^{31}P\{^1H\}$ NMR (202 MHz, C_6D_6, 25 ℃): δ (assignment by ^1H-$^{13}C\{^1H\}$ HSQC) = 29.1 (d, $^2J_{P,P}$ = 25.7 Hz, $Palk_2Ar$, **6b**), 41.0 (d, $^2J_{P,P}$ = 23.7 Hz, $Palk_2Ar$, **6a**), 41.7 (d, $^2J_{P,P}$ = 23.7 Hz, PAr_3, **6a**), 57.1 (d, $^2J_{P,P}$ = 25.7 Hz, PAr_3, **6b**) ppm; elemental analysis (%) calcd for $C_{71}H_{107}ClO_{28}P_2Pd$ • 2 H_2O: C 51.73, H 6.79, found: C 51.59, H 6.57; MS (ESI-TOF) for $C_{71}H_{107}ClO_{28}P_2Pd$: *m/z* (%): 1575.55 (100) [*M* - Cl]$^+$, 1633.50 (16) [M + Na]$^+$, 1651.49 [M + Na+ H_2O]$^+$

Methylchlorido[{6^A,6^B-dideoxy-6^A,6^B-[(1*R*,2*S*)-1,2-phenylenebis (phenylphosphanyl)]-2^A,2^B,2^C,2^D,2^E,2^F,3^A,3^B,3^C,3^D,3^E,3^F,6^C,6^D,6^E,6^F-hexadeca-*O*-methyl-*α*-cyclodextrin}-*κ*^{2}-*P*,*P*]palladium(II) (Isomers **7a** and **7b**)

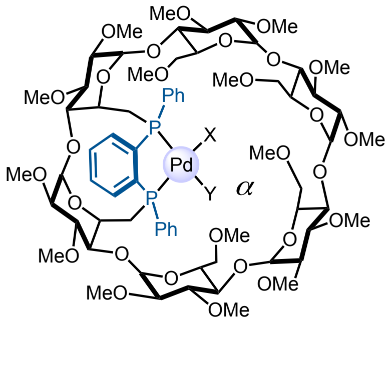

7a, X = Cl and Y = Me
7b, X = Me and Y = Cl

A solution of [PdMeCl(COD)] (0.013 g, 0.05 mmol) in THF (1 mL) was added to a solution of **1** (0.073 g, 0.05 mmol) in THF (1 mL). The reaction mixture was stirred for 2 h at room temperature and then evaporated to dryness under reduced pressure. The resulting yellow residue was subjected to column chromatography (SiO_2, CH_2Cl_2/MeOH, 97/3, *v/v*) to afford the product as a yellow solid (0.06 g, 74 %). The ^1H and $^{31}P\{^1H\}$ NMR spectra show two isomers in a *ca*. 60:40 ratio in CD_2Cl_2 at room temperature. A crystalline material of the mixture **7a,b** was obtained by slow diffusion of *n*-pentane into a dichloromethane solution of **7a,b**. $^{31}P\{^1H\}$ NMR (162 MHz, CD_2Cl_2,

25 °C): δ = 34.9 (d, $^{3+2}J_{P,P}$ = 26.7 Hz, $P_{\mathbf{7a}}$), 41.00 (d, $^{3+2}J_{P,P}$ = 26.1 Hz, $P_{\mathbf{7b}}$), 54.7 (d, $^{3+2}J_{P,P}$ = 26.1 Hz, $P_{\mathbf{7b}}$), 63.9 (d, $^{3+2}J_{P,P}$ = 26.7 Hz, $P_{\mathbf{7a}}$) ppm; elemental analysis (%) calcd for $C_{71}H_{107}ClO_{28}P_2Pd$ • 2 H_2O: C 51.73, H 6.79, found: C 51.41, H 6.50; MS (ESI-TOF) for $C_{71}H_{107}ClO_{28}P_2Pd$: *m/z* (%): 829.25 (13) [*M* + 2Na]$^{2+}$, 1575.55 (20) [*M* - Cl]$^{+}$, 1635.51 (16) [M + Na]$^{+}$, 1651.48 (8) [*M* + Na + H_2O]$^{+}$

Methylchlorido[{6^{A},6^{B}-dideoxy-6^{A},6^{B}-[(*R*)-2-(*N,N*-dimethylaminophenyl) phosphinidene]-2^{A},2^{B},2^{C},2^{D},2^{E},2^{F},3^{A},3^{B},3^{C},3^{D},3^{E},3^{F},6^{C},6^{D},6^{E},6^{F}-hexadeca-*O*-methyl-*α*-cyclodextrin})-*κ*^{2}-*P,N*]palladium(II) (complex [NiBr$_2$(**L^{6}**)])

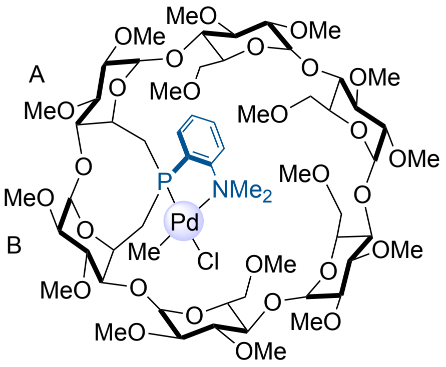

To a solution of the cavity-shaped P^N ligand **L^{6}**[5] (298 mg, 0.22 mmol) in CH_2Cl_2 (2 mL) was added dropwise a solution of PdMeCl(COD) (60.1 mg, 0.22 mmol) in CH_2Cl_2. The reaction mixture was stirred for 2 hours at room temperature. The solution was concentrated under reduced pressure and the resulting yellow residue was subjected to column chromatography (SiO_2, CH_2Cl_2/MeOH, 97/3, *v/v*) to afford pure [NiBr$_2$(**L^{6}**)]) (yield: 197 mg, 59%) as a pale yellow solid; R_f (SiO_2, CH_2Cl_2/MeOH, 95:5, *v/v*) = 0.28; m.p. dec. >250℃; ^{1}H NMR (500 MHz, $CDCl_3$, 25°C): δ (partial assignment by combined COSY, ROESY, TOCSY and HSQC) = 0.78 (d, 3H, $^{3}J_{HP}$ = 2.5 Hz, -PdMe), 1.90 (m, 1H, H-6a^{A}), 2.26 (m, 1H, H-6a^{B}), 3.07 (s, 3H, -NMe), 3.08 (m, 1H, H-2^{A}), 3.15-3.19 (4H, H-2^{C,D,E,F}), 3.20 (2H, H-4^{A,B}), 3.22 (s, 3H, -NMe), 3.30 (m, 1H, H-2^{B}), 3.31 (s, 3H, OMe), 3.39 (s, 3H OMe), 3.39-3.48 (2H, H-6b^{A,B}), 3.44 (m, 1H, H-3^{C}), 3.44 (s, 3H, OMe), 3.45 (s, 3H OMe), 3.46 (9H, OMe), 3.48 (s, 3H, OMe), 3.49 (s, 3H, OMe), 3.49-3.55 (2H, H-3^{A,B}), 3.52(s, 3H, OMe), 3.53-3.60 (3H, H-3^{D,E,F}), 3.60 (s, 3H, OMe), 3.62 (s, 3H, OMe), 3.64 (s, 6H, OMe), 3.65 (1H, H-4^{C}), 3.67 (s, 3H, OMe), 3.69 (s, 3H, OMe=), 3.72 (m, 1H, H-4^{F}), 3.78-3.83 (m, 3H, H-4^{D}, H-6a^{C,D}), 3.81 (m, 1H, H-5^{D}), 3.84-3.89 (2H, H-4^{E}, H-6a^{E}), 3.88 (m, 1H, H-5^{C}), 3.92 (m, 1H, H-5^{E}), 3.99-4.04 (2H, H-6b^{D}, H-6a^{F}), 4.03 (m, 1H, H-5^{F}), 4.07 (m, 1H, H-6b^{C}), 4.19 (m, 1H, H-6b^{E}), 4.29 (m, 1H, H-5^{B}), 4.41 (m, 1H, H-5^{A}), 4.44 (m, 1H, H-6b^{F}), 4.96 (d, $^{3}J_{H-2,H-1}$ = 3.4 Hz, 1H, H-1^{C}), 5.02 (d, $^{3}J_{H-2,H-1}$ = 3.1 Hz, 1H, H-1^{A}), 5.07 (d, $^{3}J_{H-2,H-1}$ = 3.2 Hz, 1H, H-1^{D}), 5.09 (d, $^{3}J_{H-2,H-1}$ = 4.4 Hz, 1H, H-1^{B}), 5.09-5.12 (2H, H-1$^{E,\ F}$), 7.45 (m, 1H, aromatic H), 7.52-7.60 (m, 3H, aromatic H) ppm; $^{13}C\{^{1}H\}$ NMR (126 MHz, $CDCl_3$, 25°C): δ (partial assignment by HSQC) = -12.06

(d, $^{2}J_{p,c}$ = 4.4 Hz, PdMe), 30.08 (m, C-6^{A}), 40.72 (d, C-6^{B}), 51.33, 51.78 (-NMe$_2$), 57.58, 57.69, 57.73, 57.91, 57.97, 57.99 (OMe-2), 59.24, 59.27, 59.30, 59.37 (OMe-6), 61.78, 61.80, 61.91, 61.98, 62.24, 62.57 (CH_3O-3), 65.67 (d, $^{2}J_{P,C}$ = 6.7 Hz, C-5^{A}), 68.58 (d, $^{2}J_{P,C}$ = 4.6 Hz, C-5^{B}), 71.16, 70.71, 71.42, 70.93 (C-5^{C,D,E,F}), 71.44, 71.68, 71.92, 72.49 (C-6^{C,D,E,F}), 80.41, 81.15, 81.26, 81.46, 81.57, 81.65, 81.70, 81.94, 82.10 [x3], 82.34, 82.37. 82.51, 82.62, 83.07 (C-2, C-3, C-4^{C,D,E,F}), 88.03 (d, $^{2}J_{P,C}$ = 10.2 Hz, C-4^{A}), 89.80 (d, $^{2}J_{P,C}$ = 4.3 Hz, C-4^{B}), 98.09, 99.87, 99.92, 100.41, 100.82, 101.33 (C-1), 122.72 (d, $^{2}J_{P,C}$ = 9.9 Hz, C-6$_{arom}$), 129.25 (d, $^{2}J_{P,C}$ = 6.1 Hz, C-5$_{arom}$), 131.68 (C-4$_{arom}$), 133.30 (C-3$_{arom}$), 133.81 (d, $^{1}J_{P,C}$ = 46.1 Hz, C-1$_{arom}$), 158.19 (d, $^{3}J_{P,C}$ = 16.4 Hz, C-2$_{arom}$) ppm; ^{31}P{^{1}H} NMR (162 MHz, $CDCl_3$, 25°C): δ = 17.9 ppm; elemental analysis (%) calcd for $C_{61}H_{103}ClNO_{28}PPd$: C 49.80, H 7.06, N 0.95, found: C 49.79, H 7.22, N 0.91; MS (ESI-TOF) for $C_{61}H_{103}ClNO_{28}PPd$: *m/z* (%): 1434.54 (5) [*M* - Cl]$^+$, 1461.56 (40) [*M* - Cl + CN + H]$^+$, 1683.54 (100) [*M* - Cl + CN + Na]$^+$

3. Procedure for ethylene oligomerization reactions

The catalytic reactions were performed in a magnetically stirred (1200 rmp) 145 mL stainless steel autoclave. A 125 mL glass container was used to avoid corrosion of the autoclave walls. The precatalyst solution was prepared by dissolving 1 x 10^{-5} mol of the complex in dry toluene. This solution was injected into the reactor under an ethylene flux, followed by the cocatalyst solution (400 equiv. of MMAO-12 in toluene). After injection of the catalyst and cocatalyst solutions under a constant low flow of ethylene, which is considered as the t_0 time, the reactor was immediately pressurized to 10 bar of ethylene. The 10 bar working pressure was maintained through a continuous feed of ethylene from a bottle placed on a balance to allow monitoring of the ethylene uptake. The reaction mixture was stirred for the given reaction time. At the end of each test, a dry ice bath was used to rapidly cool the reactor. When the inner temperature reached 0 ℃, the ice bath was removed, allowing the temperature to slowing rise to 18 ℃. The gaseous phase was then transferred into a 10 L polyethylene tank filled with water. An aliquot of this gaseous phase was transferred into a Schlenk flask, previously evacuated, for GC analysis. The amount of ethylene consumed was thus determined by differential weighting of the bottle (accuracy of the scale: 0.01g). To this amount of ethylene, the remaining ethylene (calculated using the GC analysis) in the gaseous phase was subtracted. Although this method is of limited

accuracy, it was used throughout, and it gave satisfactory reproducibility and a correct mass balance. The reaction mixture in the reactor was quenched *in situ* by the addition of ethanol (5 mL), transferred into a Schlenk flask, and separated from the metal complexes by trap-to-trap evaporation into a second Schlenk flask previously immersed in liquid nitrogen in order to avoid loss of product for GC analysis. Each catalytic test was performed at least twice to ensure the reproducibility of the results.

4. NMR and mass spectra

All NMR spectra were recorded at 25 °C.

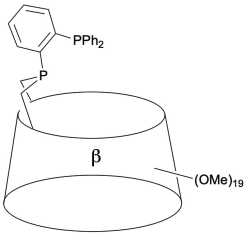

$\mathbf{L^4}$

$\mathbf{L^4}$ (•): δ = -27.3 (d, $^3J_{P,P}$ = 157.2 Hz), -13.6 (d, $^3J_{P,P}$ = 157.2 Hz) ppm

diastereomer of $\mathbf{L^4}$ (*): δ = -52.7 (d, $^3J_{P,P}$ = 181.1 Hz), -13.3 (d, $^3J_{P,P}$ = 181.1 Hz) ppm

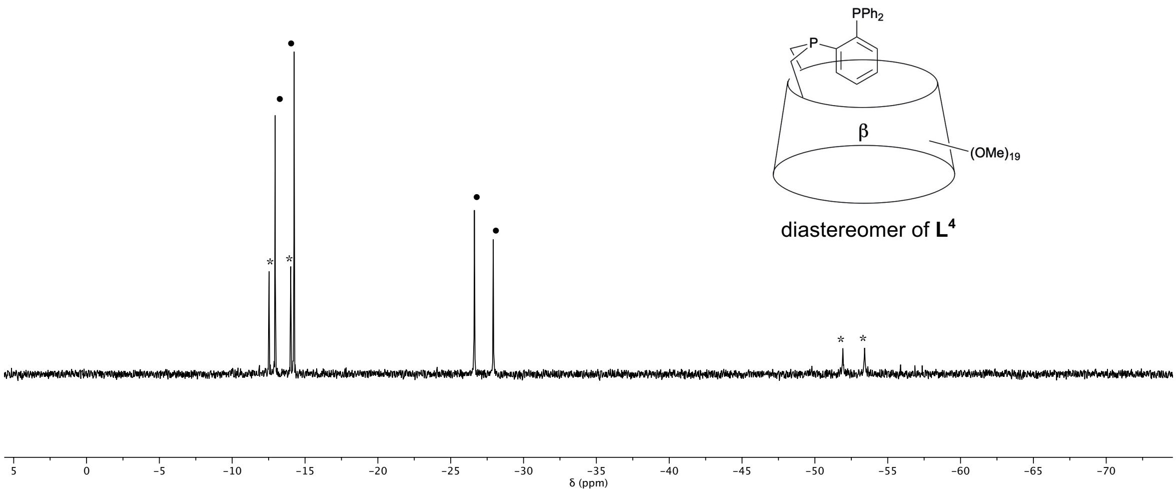

Figure S1. ^{31}P NMR (121 MHz, $CDCl_3$) spectrum of the mixture of diphosphane $\mathbf{L^4}$, its diastereomer (70:30) and residual dimesylate **2**.

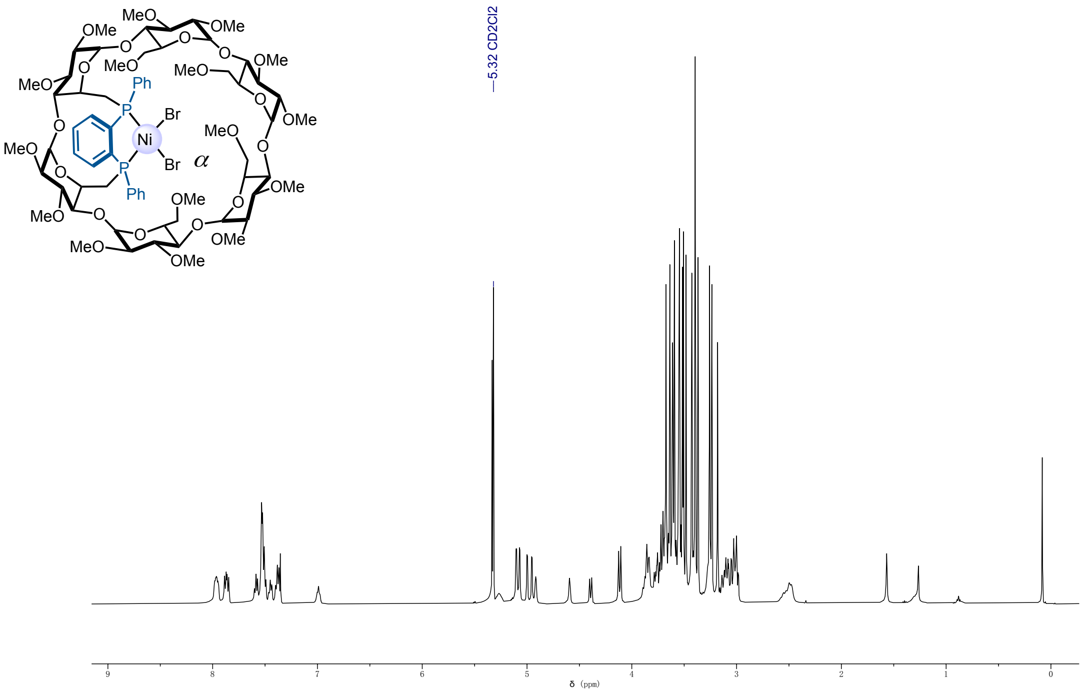

Figure S2. ^{1}H NMR (500 MHz, CD_2Cl_2) spectrum of $[NiBr_2(\mathbf{L^1})]$.

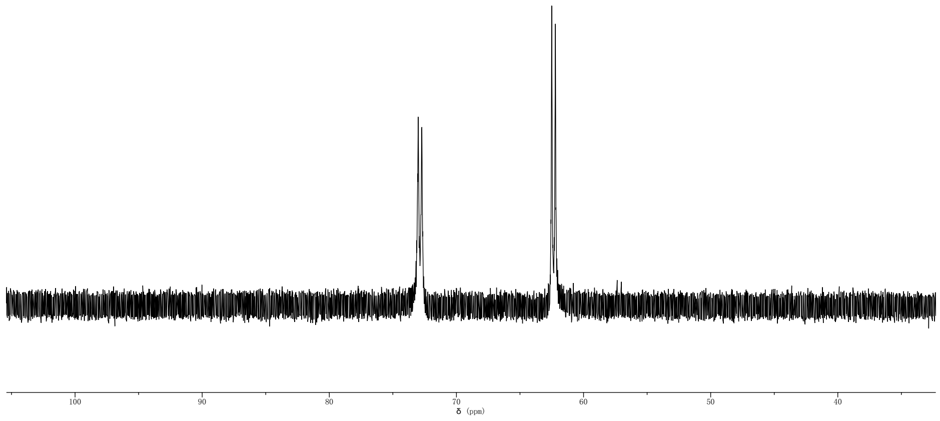

Figure S3. $^{31}P\{^1H\}$ NMR (202 MHz, CD_2Cl_2) spectrum of [$NiBr_2$(**L¹**)].

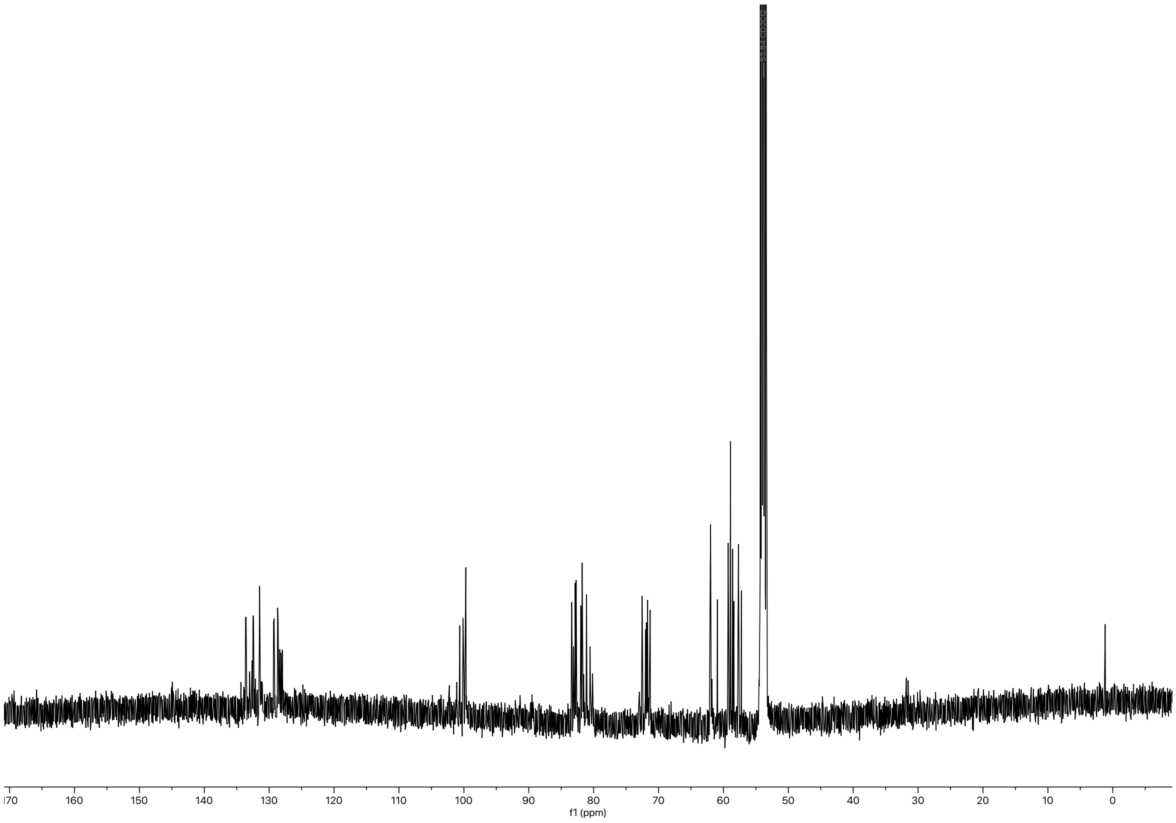

Figure S4. $^{13}C\{^1H\}$ NMR (126 MHz, CD_2Cl_2) spectrum of [$NiBr_2$(**L¹**)].

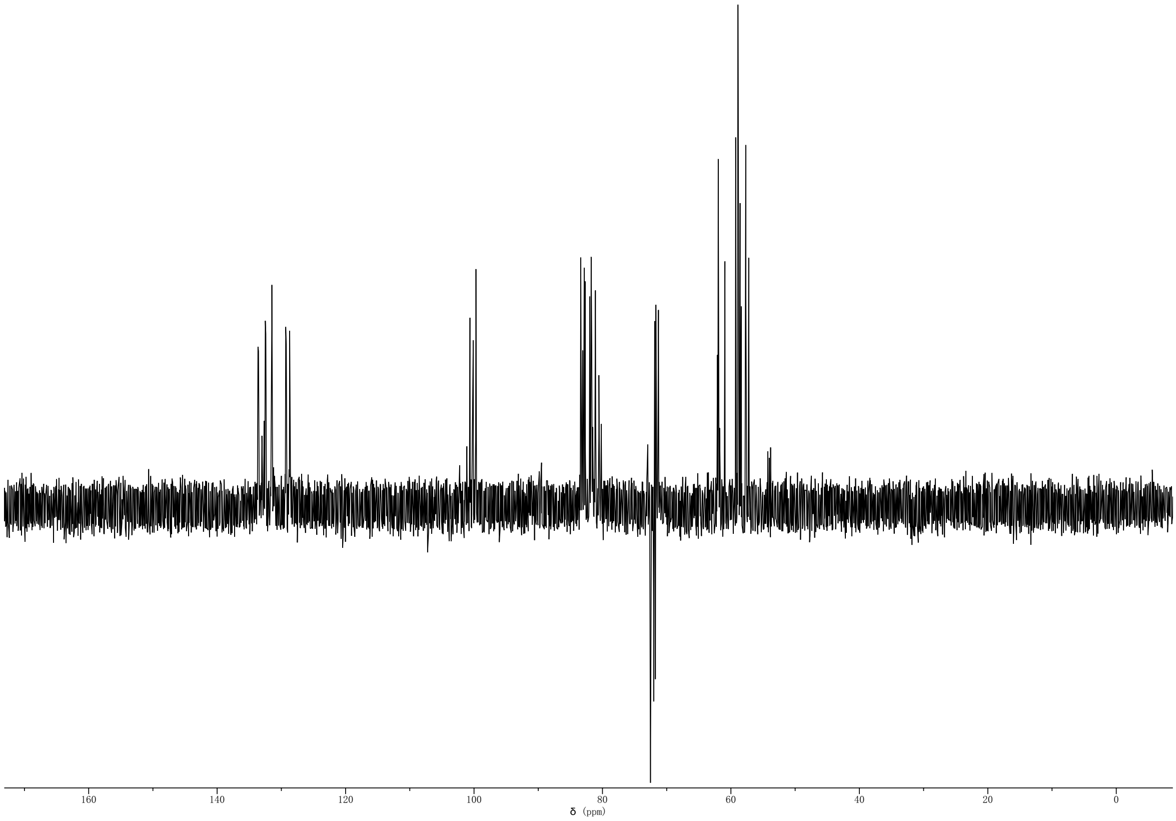

Figure S5. DEPT 135 NMR (126 MHz, CD_2Cl_2) spectrum of [$NiBr_2$(**L[1]**)].

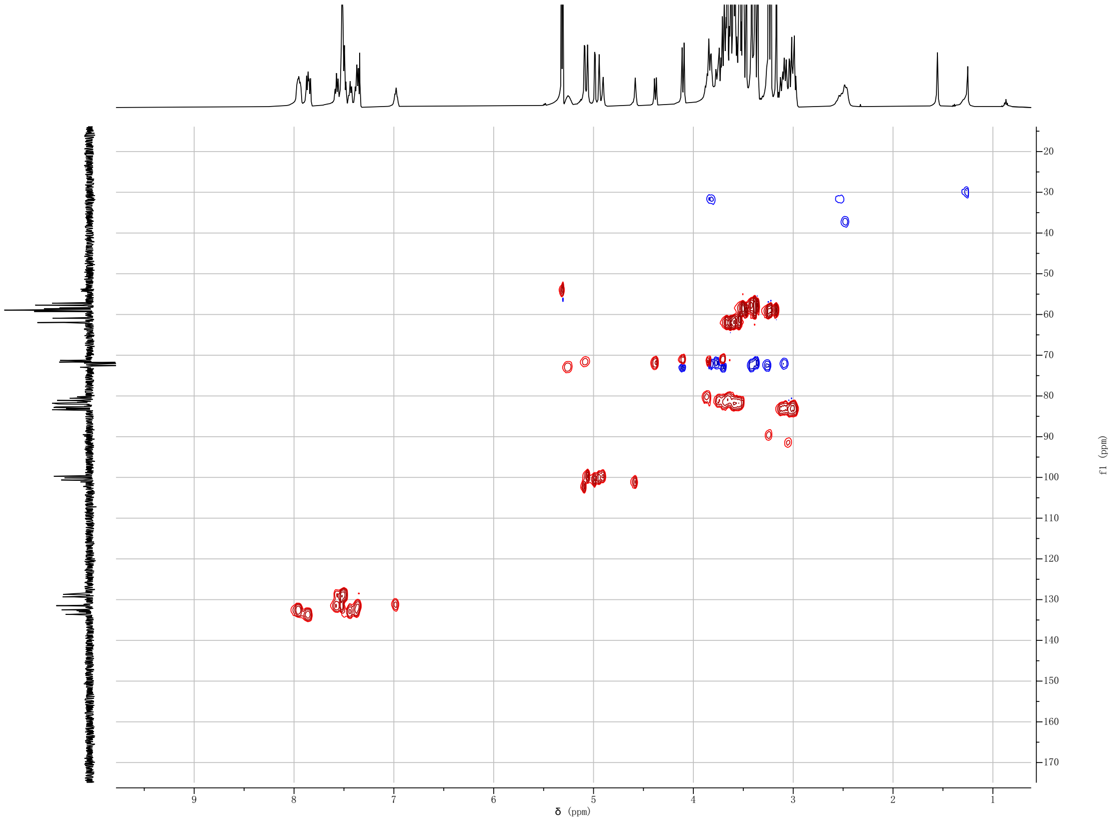

Figure S6. ^1H-$^{13}C\{^1H\}$ edited HSQC NMR (500 MHz, CD_2Cl_2) spectrum of [$NiBr_2$(**L[1]**)].

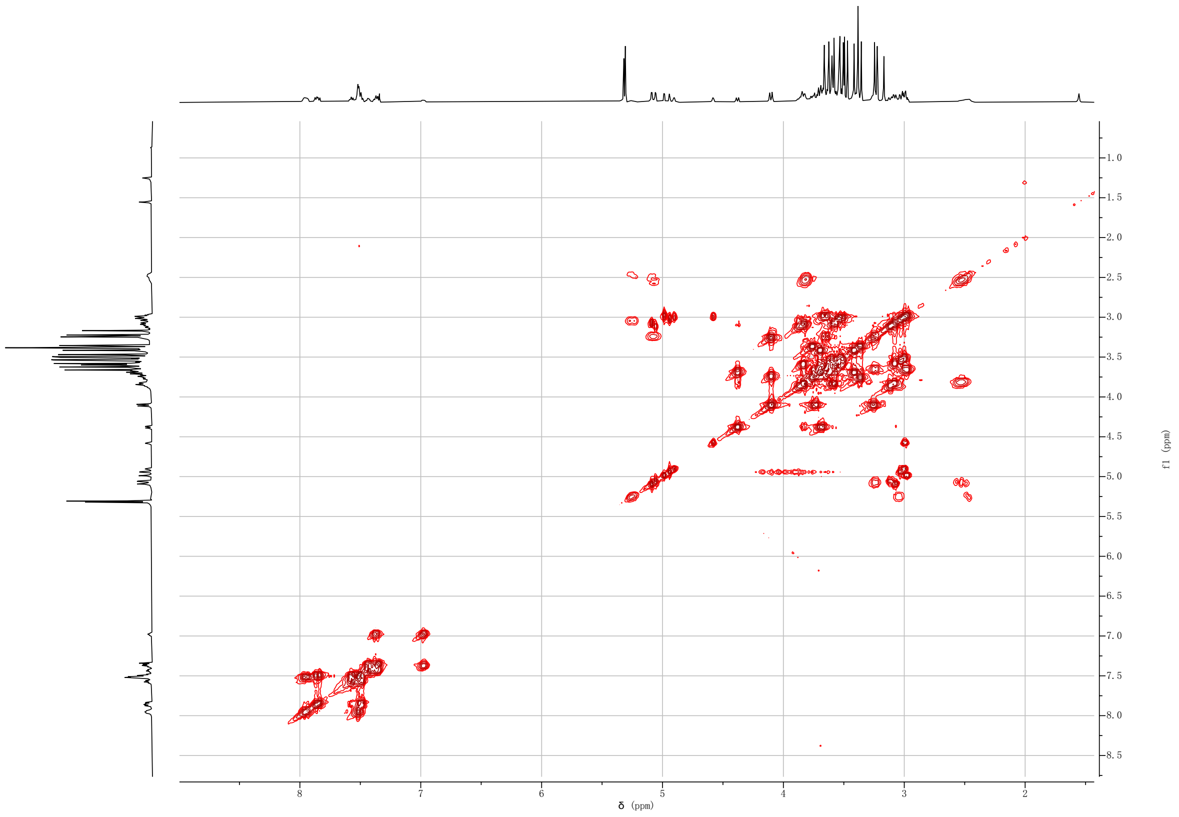

Figure S7. ^{1}H-^{1}H COSY NMR (500 MHz, CD_2Cl_2) spectrum of [$NiBr_2$(**L^1**)].

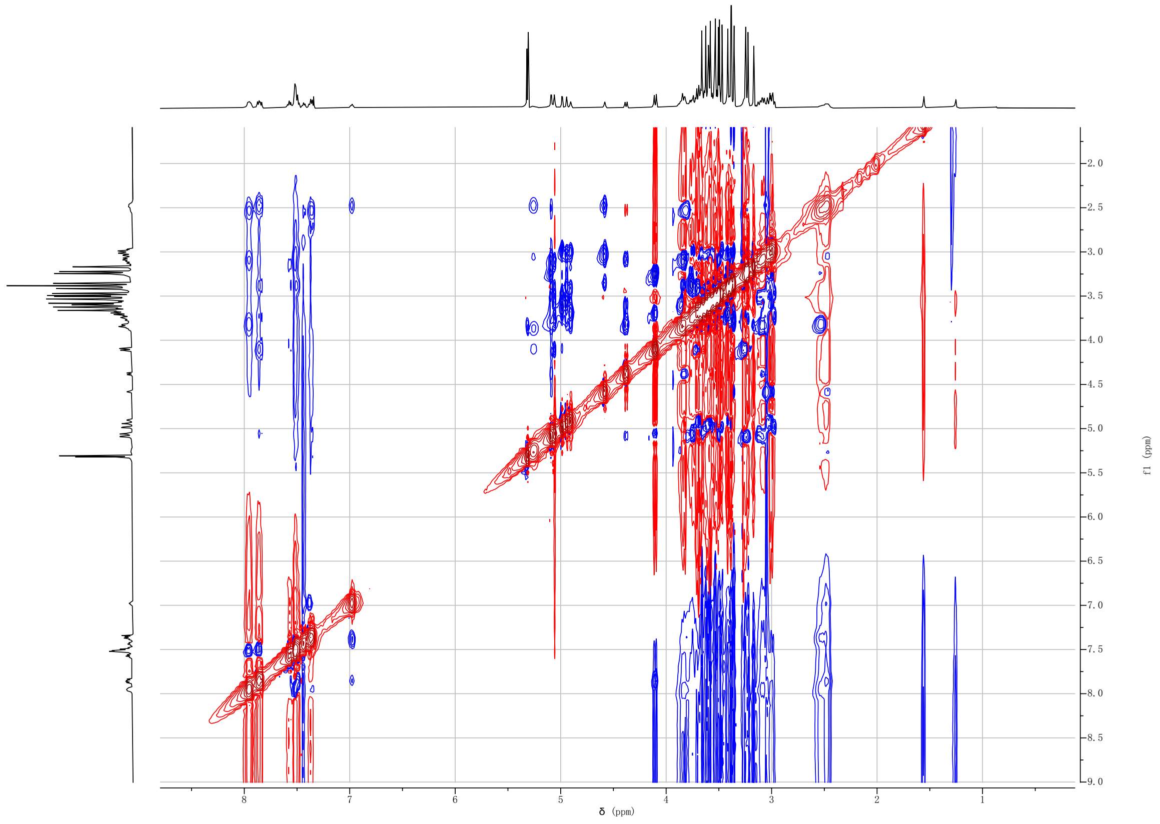

Figure S8. ^{1}H-^{1}H ROESY NMR (500 MHz, CD_2Cl_2) spectrum of [$NiBr_2$(**L^1**)]

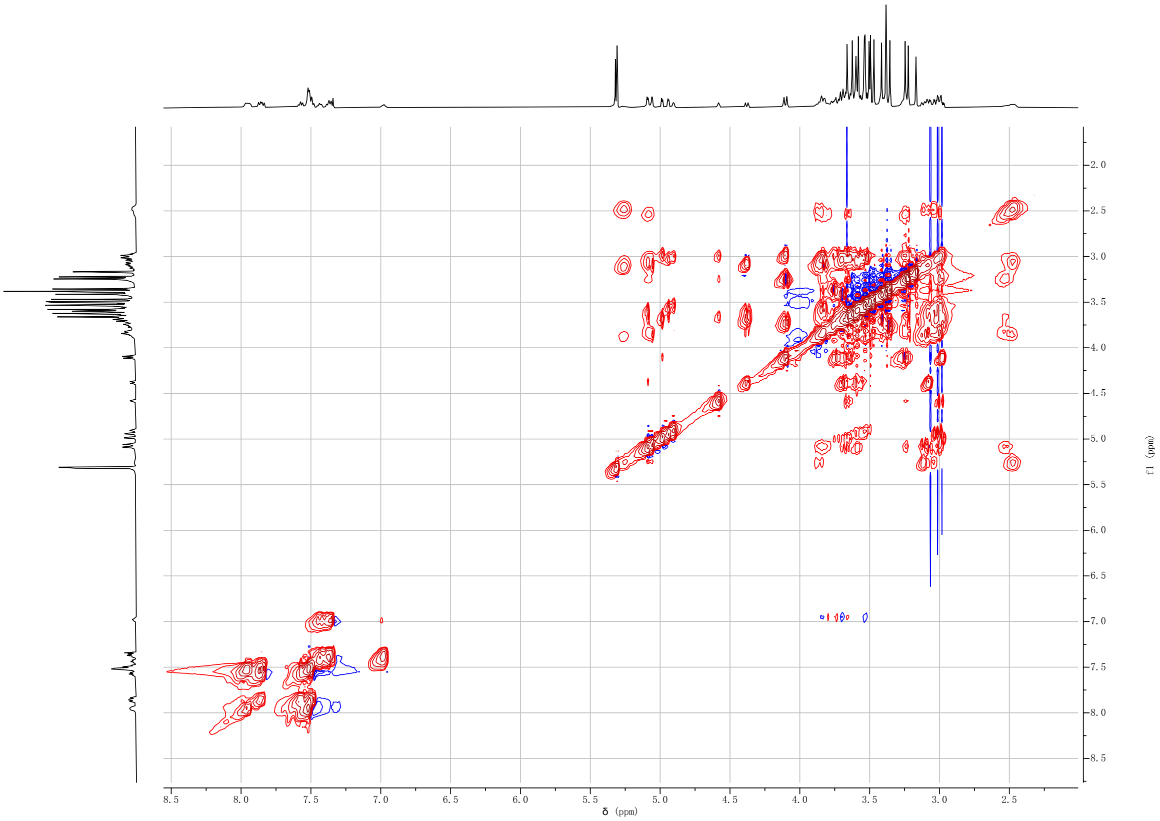

Figure S9. ^1H-^1H TOCSY NMR (500 MHz, CD_2Cl_2) spectrum of [$NiBr_2$(**L**[1])].

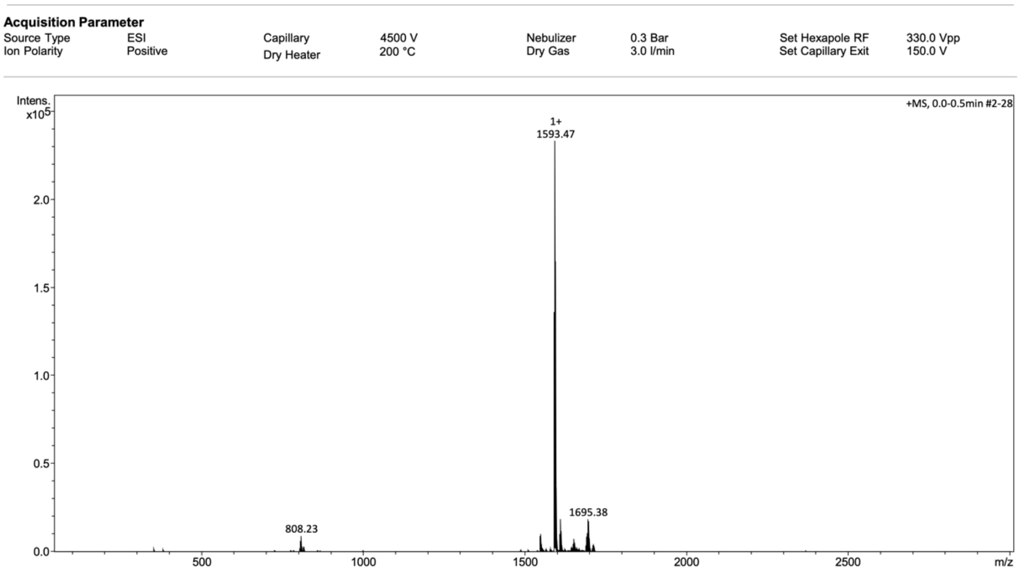

Figure S10. Full ESI-MS spectrum of [$NiBr_2$(**L**[1])].

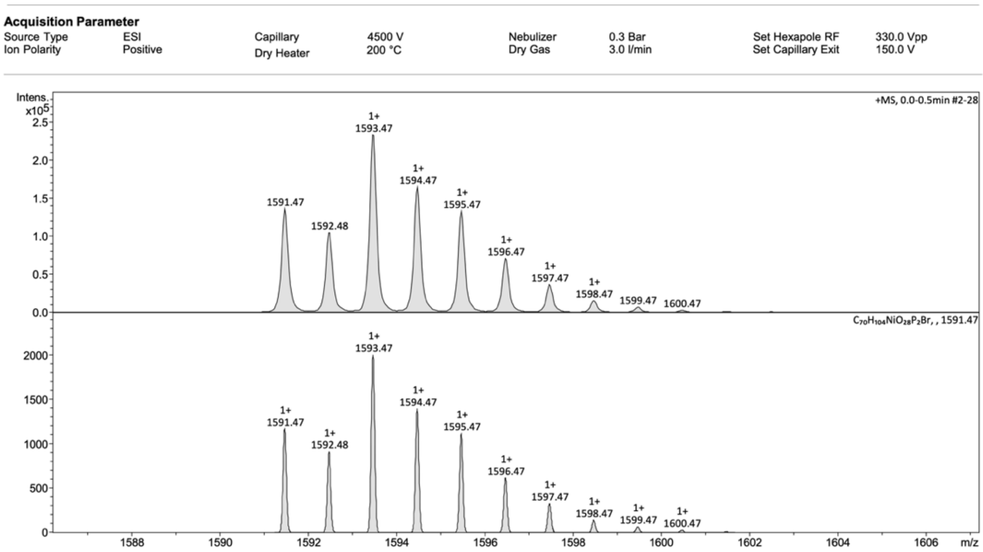

Figure S11. Experimental and simulated partial ESI-MS spectrum of [NiBr$_2$(**L^1**)].

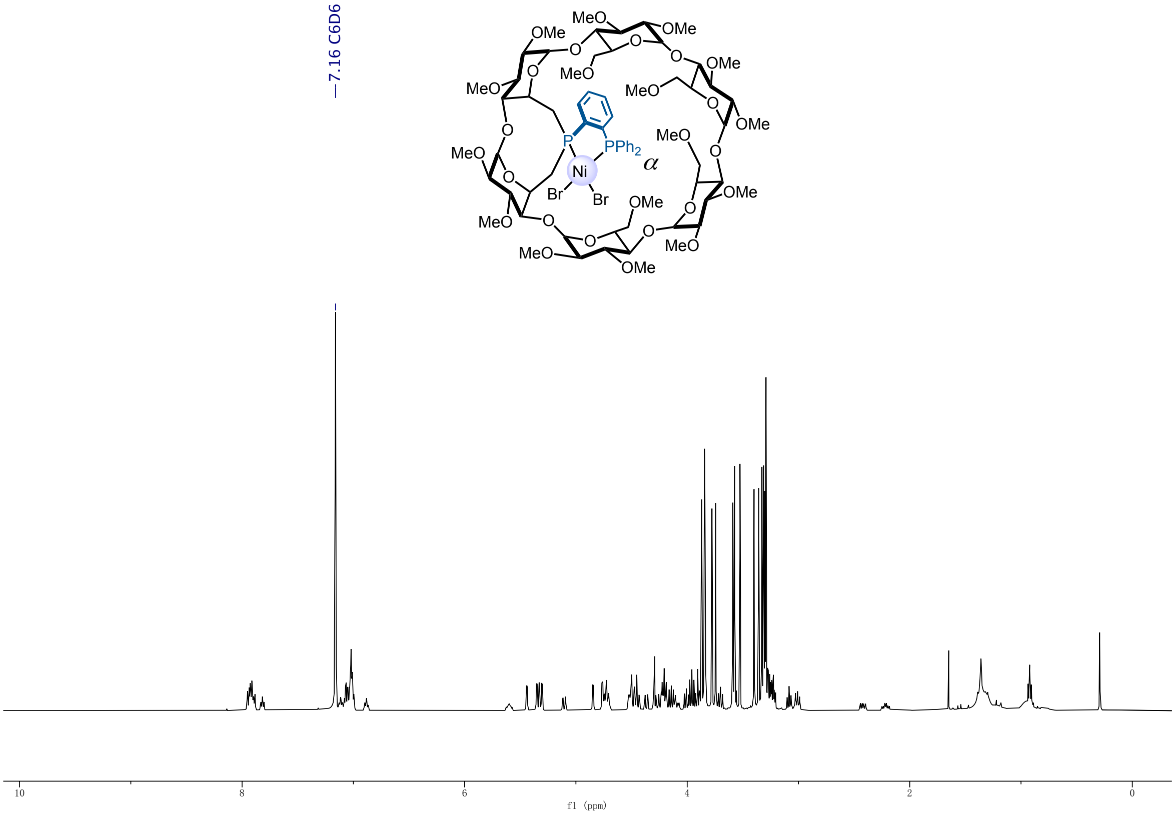

Figure S12. ^{1}H NMR (500 MHz, C_6D_6) spectrum of [NiBr$_2$(**L^3**)].

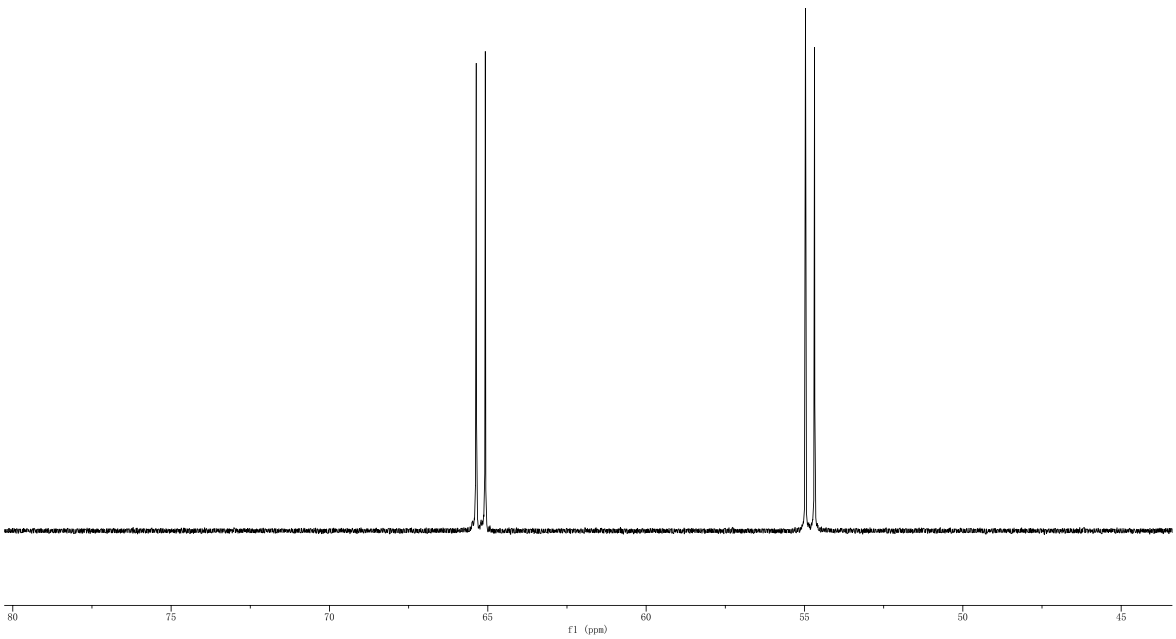

Figure S13. $^{31}P\{^1H\}$ NMR (202 MHz, C_6D_6) spectrum of [$NiBr_2$(**L^3**)].

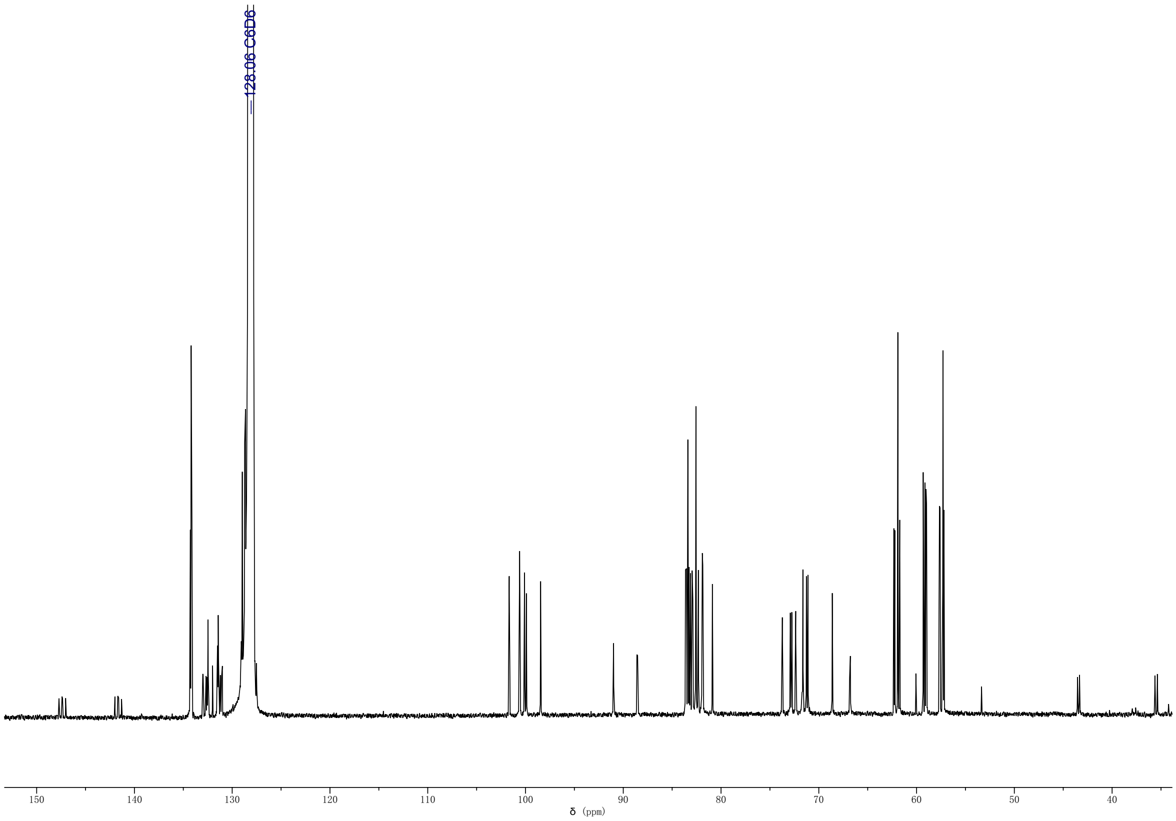

Figure S 14. $^{13}C\{^1H\}$ NMR (126 MHz, C_6D_6) spectrum of [$NiBr_2$(**L^3**)].

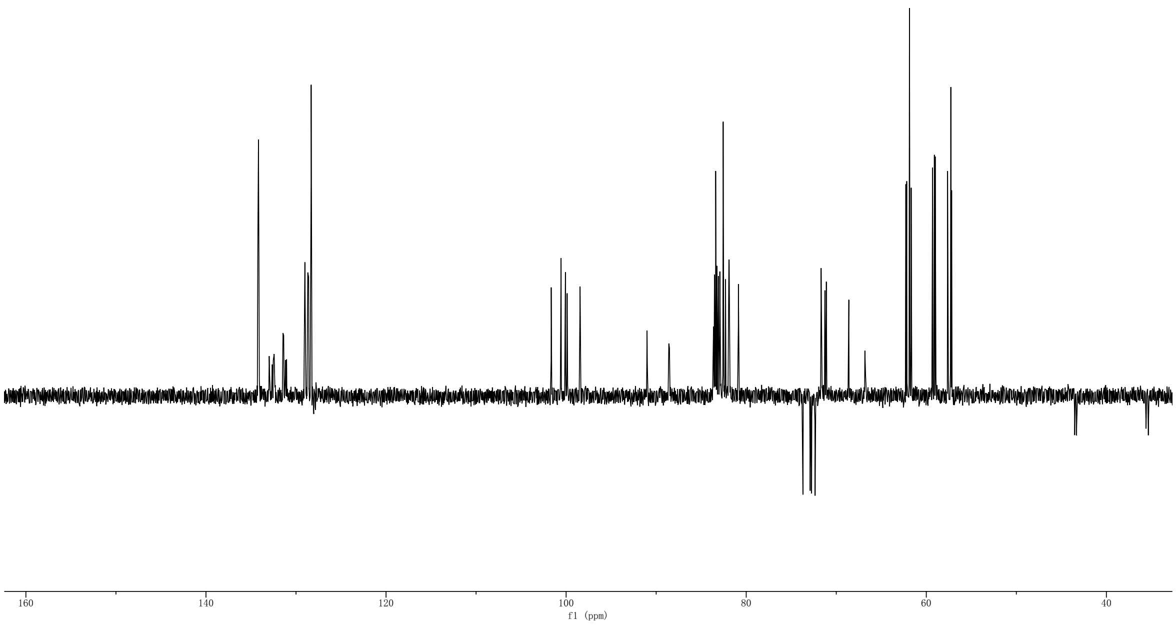

Figure S15. DEPT 135 NMR (126 MHz, C_6D_6) spectrum of [$NiBr_2$(**L**^{3})].

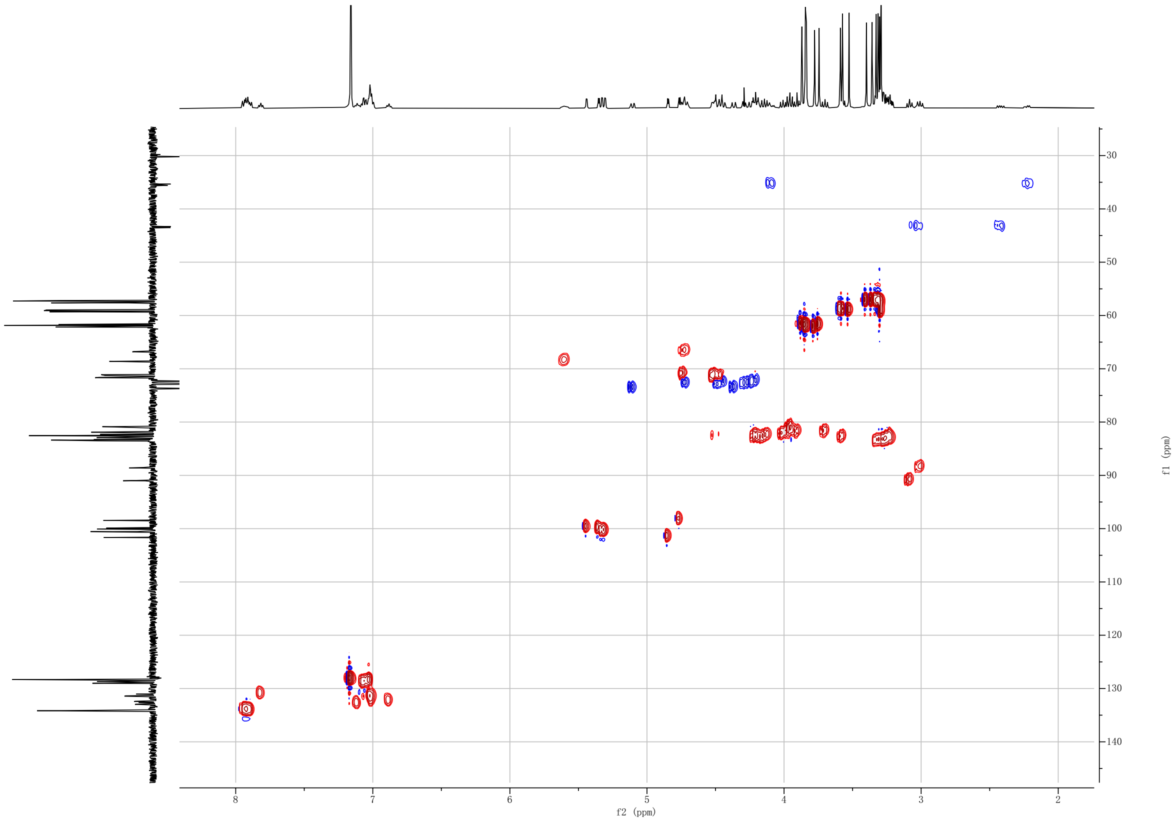

Figure S16. ^1H-$^{13}C\{^1H\}$ edited HSQC NMR (500 MHz, C_6D_6) spectrum of [$NiBr_2$(**L**^{3})].

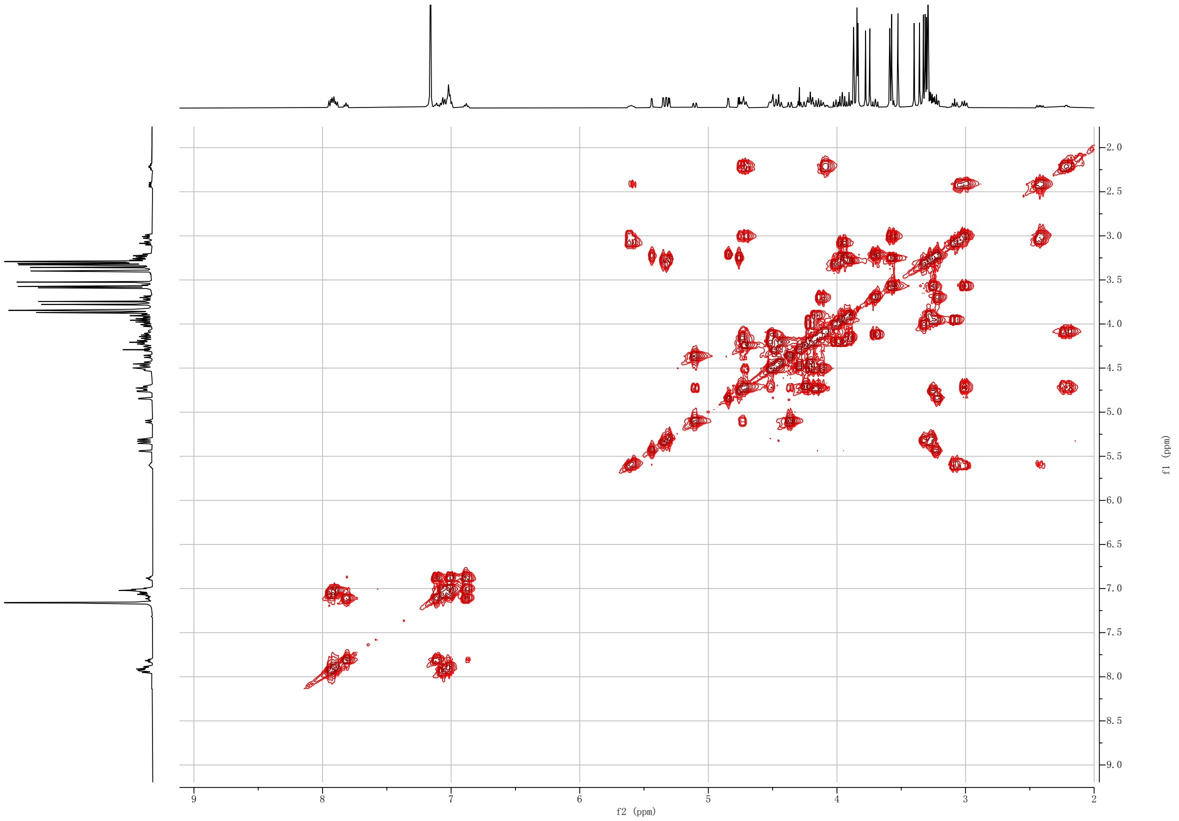

Figure S17. ^{1}H-^{1}H COSY NMR (500 MHz, C_6D_6) spectrum of [$NiBr_2$($\mathbf{L^3}$)].

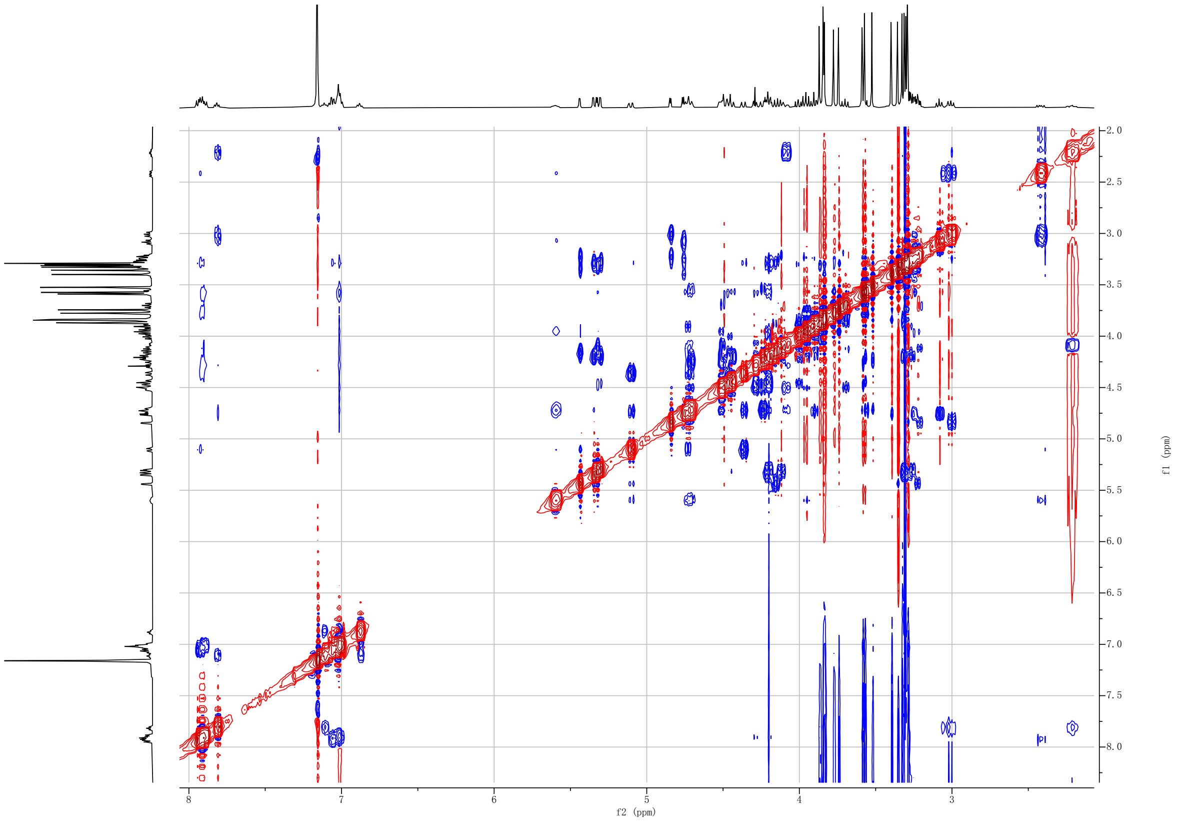

Figure S18. ^{1}H-^{1}H ROESY NMR (500 MHz, C_6D_6) spectrum of [$NiBr_2$($\mathbf{L^3}$)].

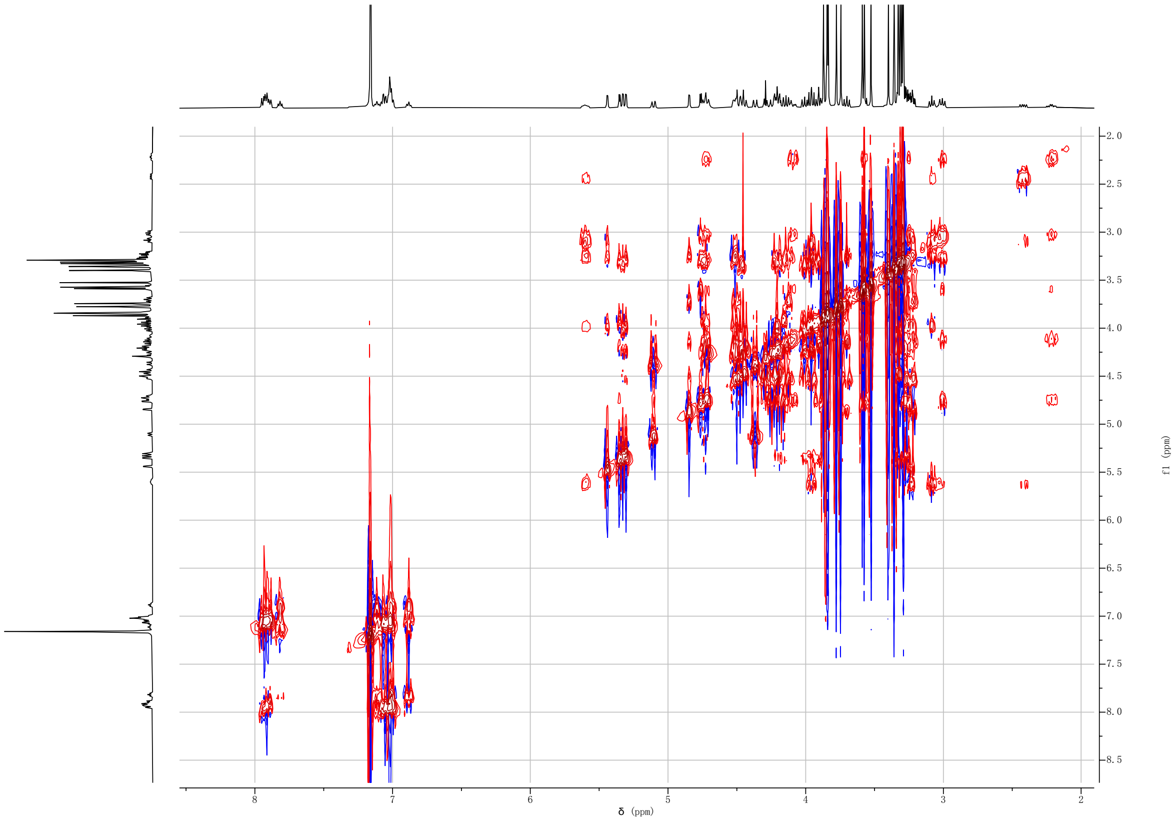

Figure S19. ^1H-^1H TOCSY NMR (500 MHz, C_6D_6) spectrum of $[NiBr_2(\mathbf{L^3})]$.

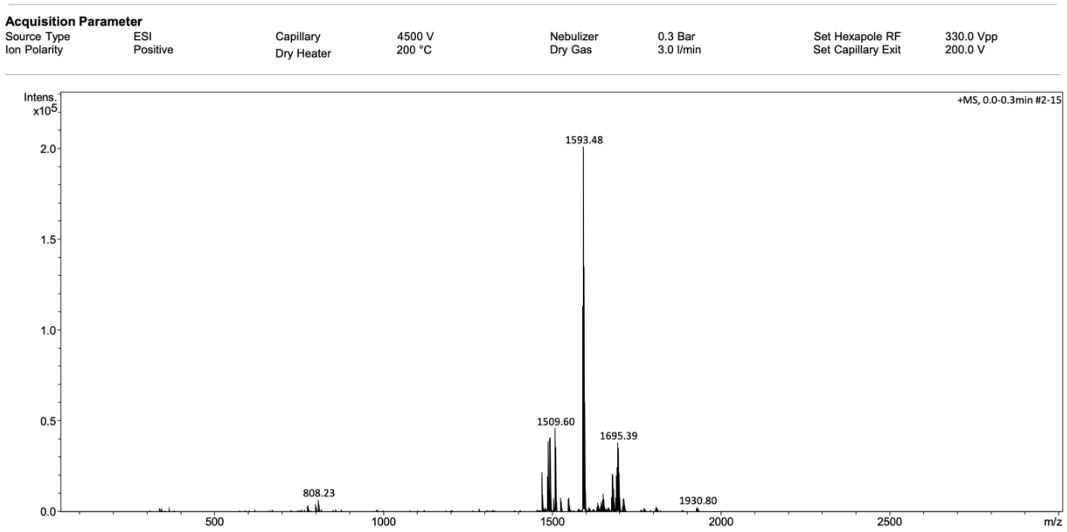

Figure S20. Full ESI-MS spectrum of $[NiBr_2(\mathbf{L^3})]$.

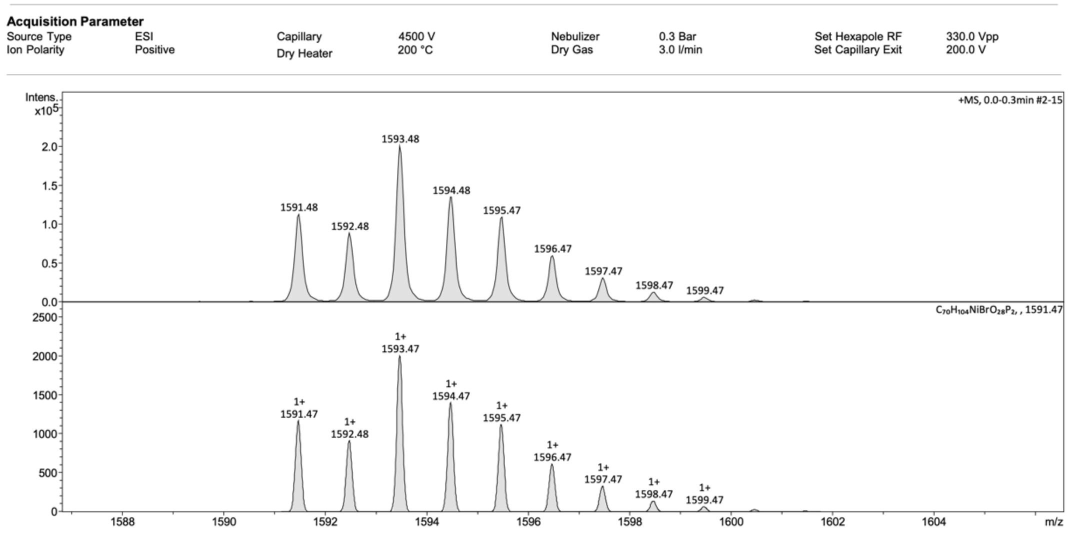

Figure S21. Experimental and simulated partial ESI-MS spectrum of [$NiBr_2$(**L**$^{\mathbf{3}}$)].

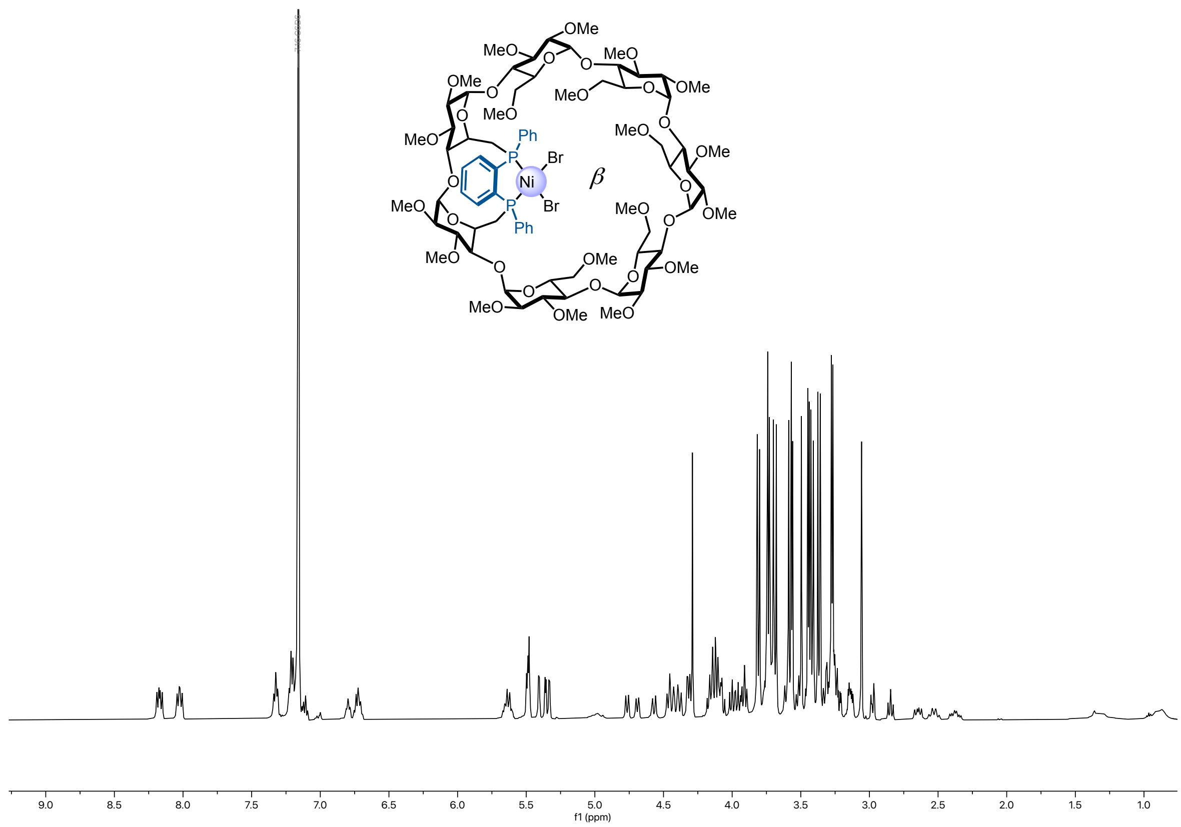

Figure S22. ^{1}H NMR (500 MHz, C_6D_6) spectrum of [$NiBr_2$(**L**$^{\mathbf{2}}$)].

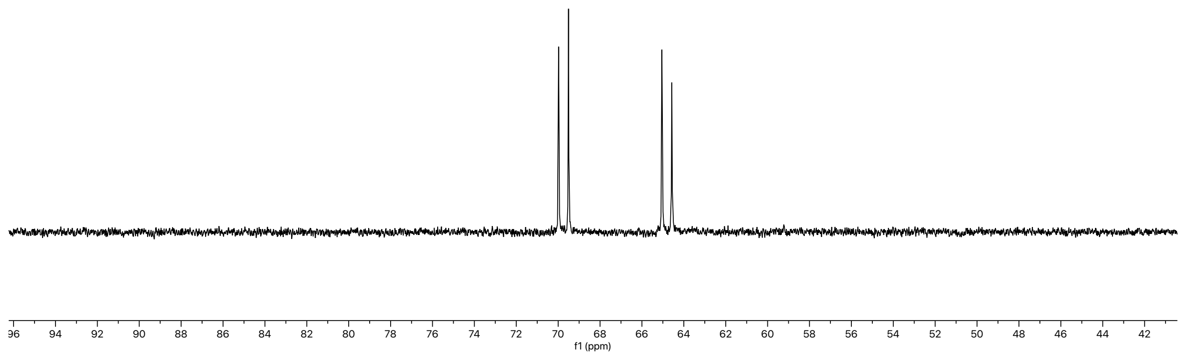

Figure S23. $^{31}P\{^{1}H\}$ NMR (121 MHz, C_6D_6) spectrum of [$NiBr_2$(**L**$^{\mathbf{2}}$)].

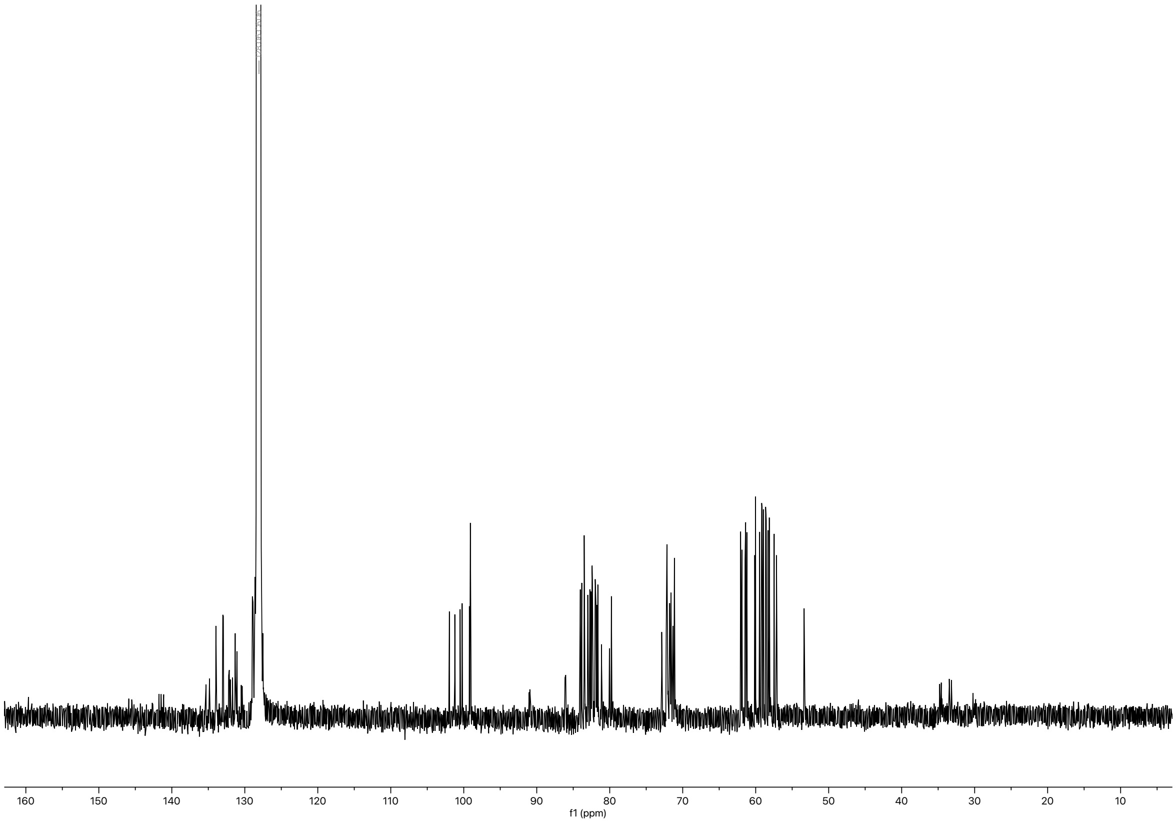

Figure S24. $^{13}C\{^{1}H\}$ NMR (126 MHz, C_6D_6) spectrum of [$NiBr_2$(**L^2**)].

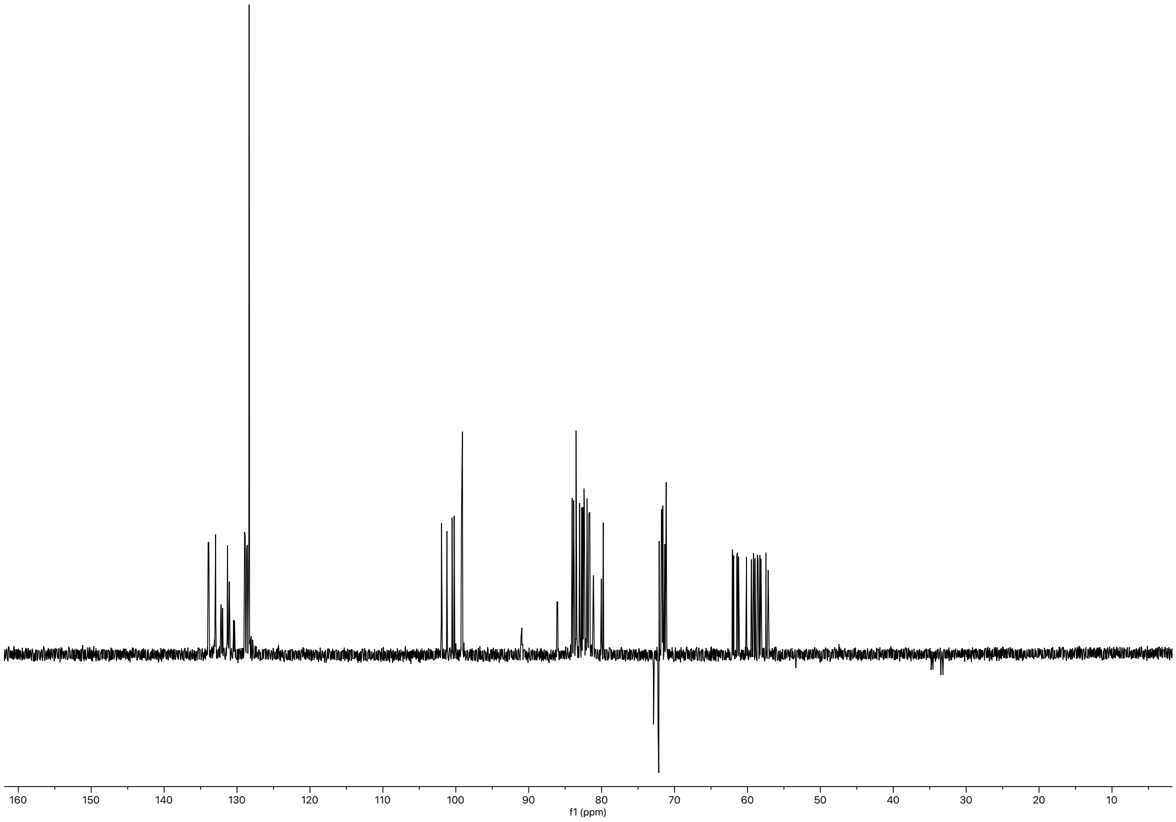

Figure S25. DEPT 135 NMR (126 MHz, C_6D_6) spectrum of [$NiBr_2$(**L^2**)].

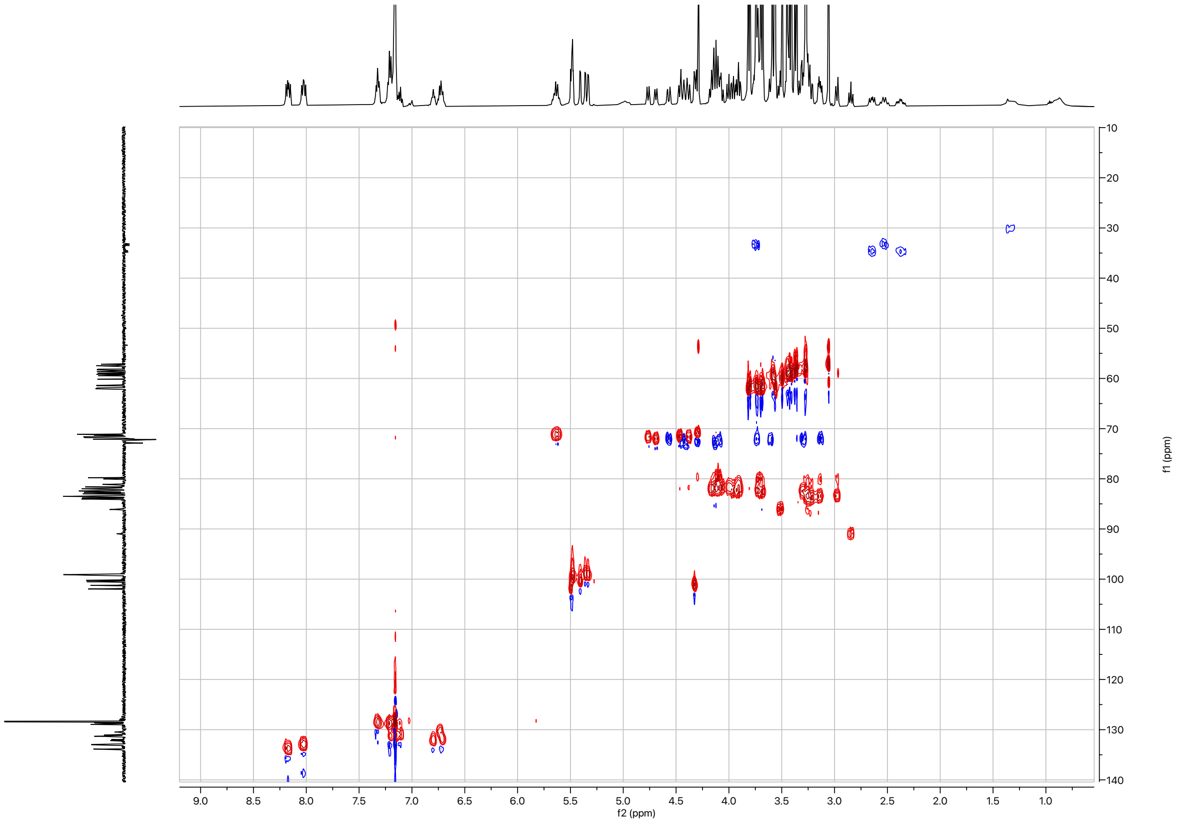

Figure S26. ^{1}H-^{13}C{^{1}H} edited HSQC NMR (500 MHz, C_6D_6) spectrum of [$NiBr_2$($\mathbf{L^2}$)].

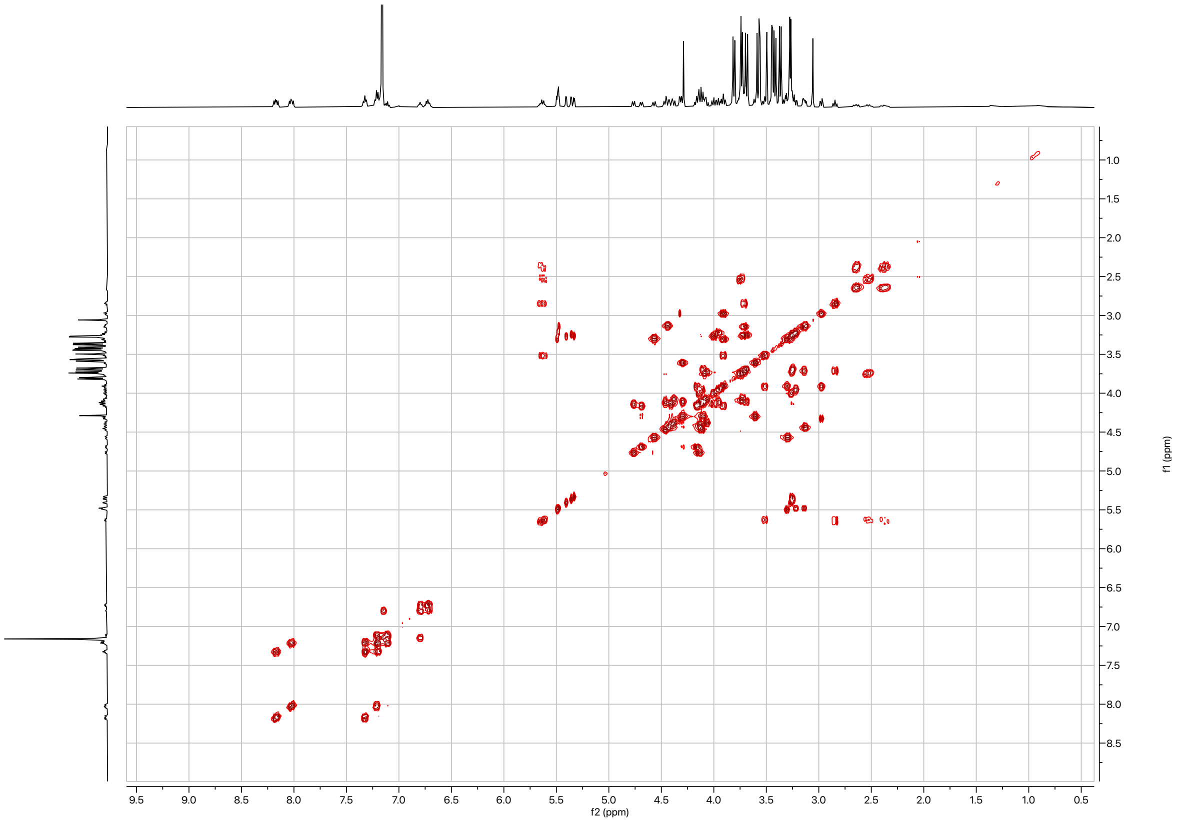

Figure S27. ^{1}H-^{1}H COSY NMR (500 MHz, C_6D_6) spectrum of [$NiBr_2$($\mathbf{L^2}$)].

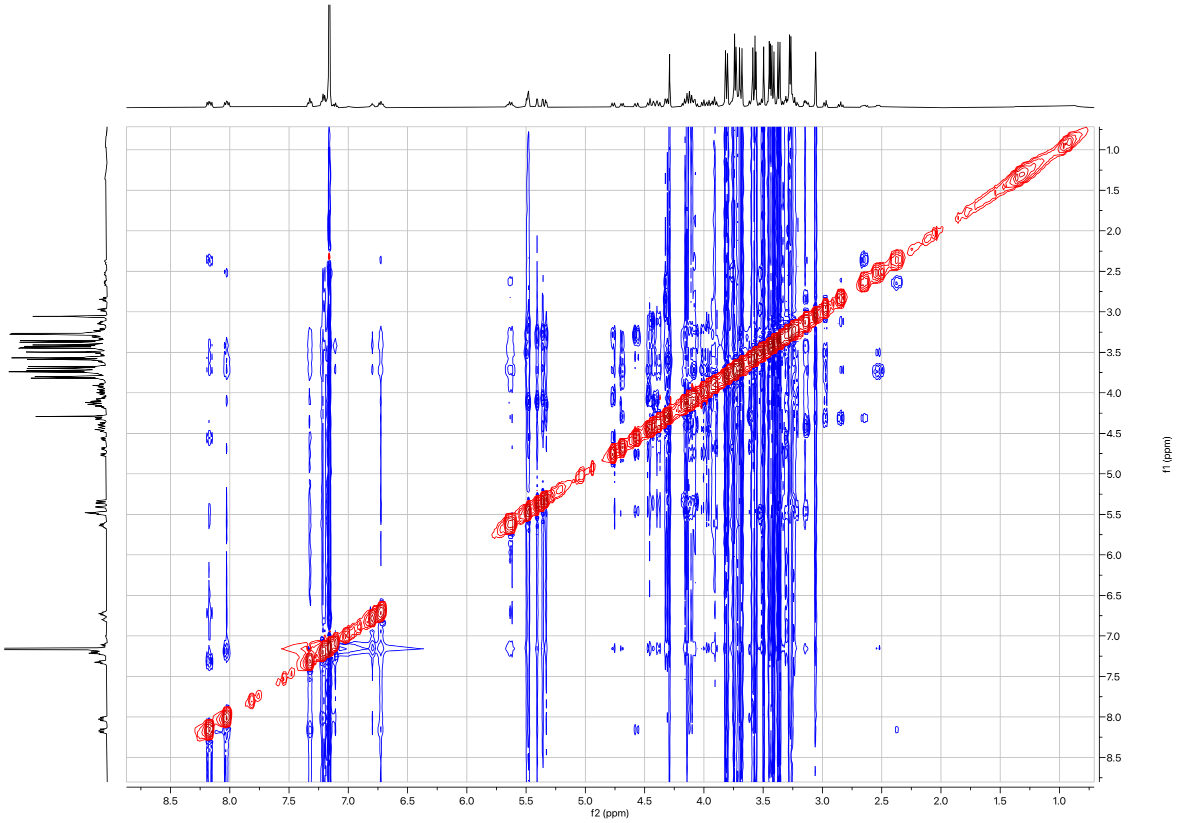

Figure S28. ^{1}H-^{1}H ROESY NMR (500 MHz, C_6D_6) spectrum of [$NiBr_2$(**L^2**)].

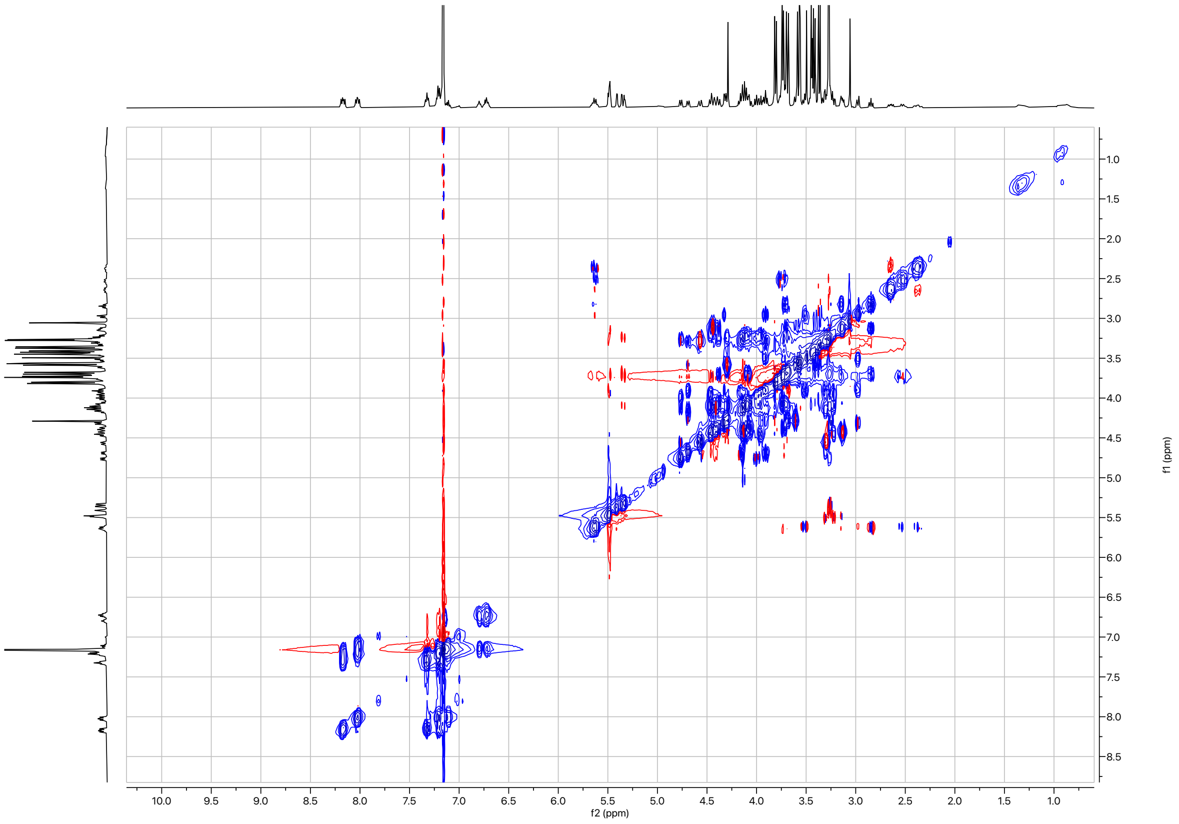

Figure S29. ^{1}H-^{1}H phase sensitive TOCSY (DIPSI-2) NMR (500 MHz, C_6D_6) spectrum of [$NiBr_2$(**L^2**)].

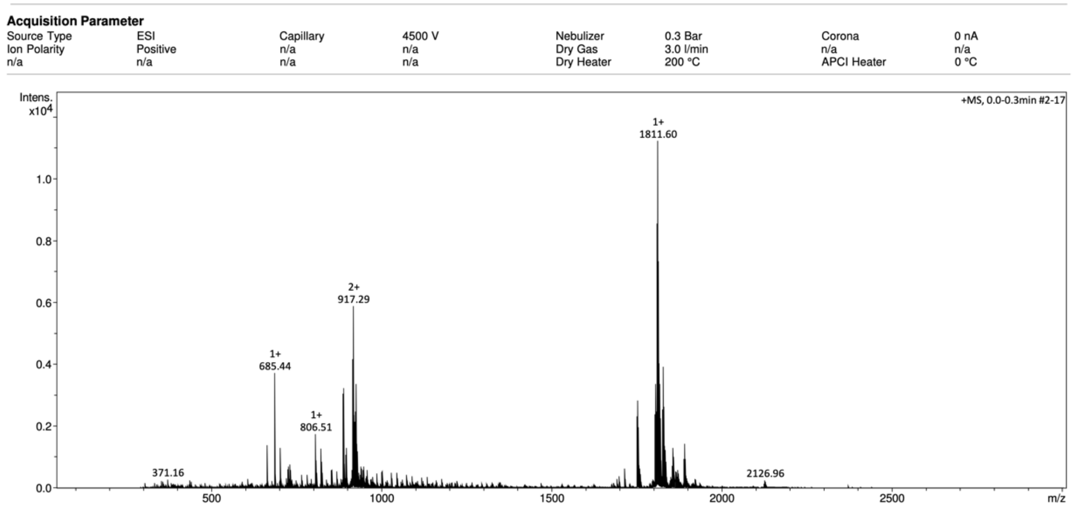

Figure S30. Full ESI-MS spectrum of [NiBr$_2$(**L^2**)].

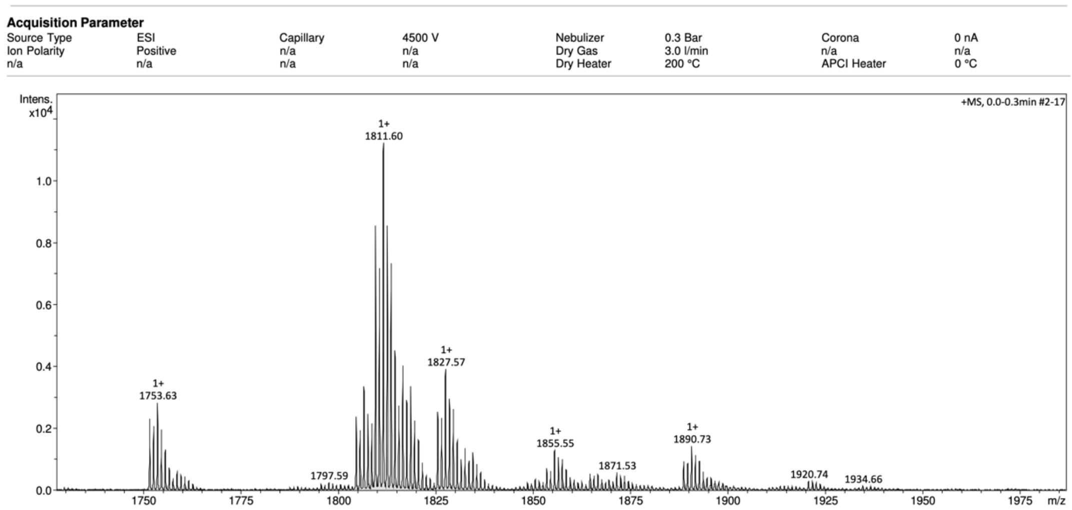

Figure S31. Partial ESI-MS spectrum of [NiBr$_2$(**L^2**)].

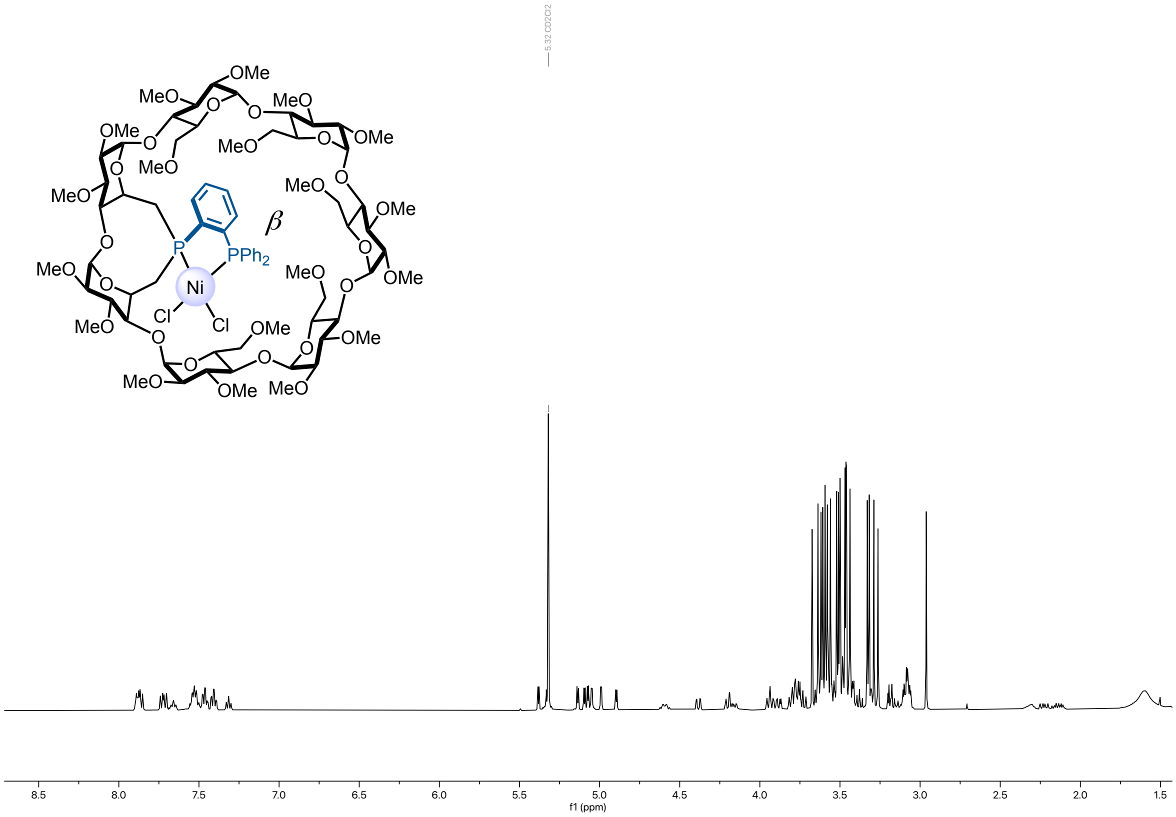

Figure S32. ^{1}H NMR (500 MHz, CD_2Cl_2) spectrum of [$NiCl_2$($\mathbf{L^4}$)].

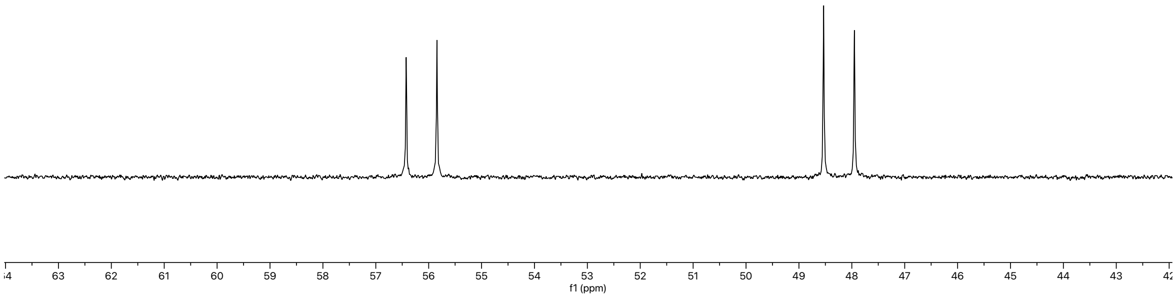

Figure S33. $^{31}P\{^{1}H\}$ NMR (121 MHz, CD_2Cl_2) spectrum of [$NiCl_2$($\mathbf{L^4}$)].

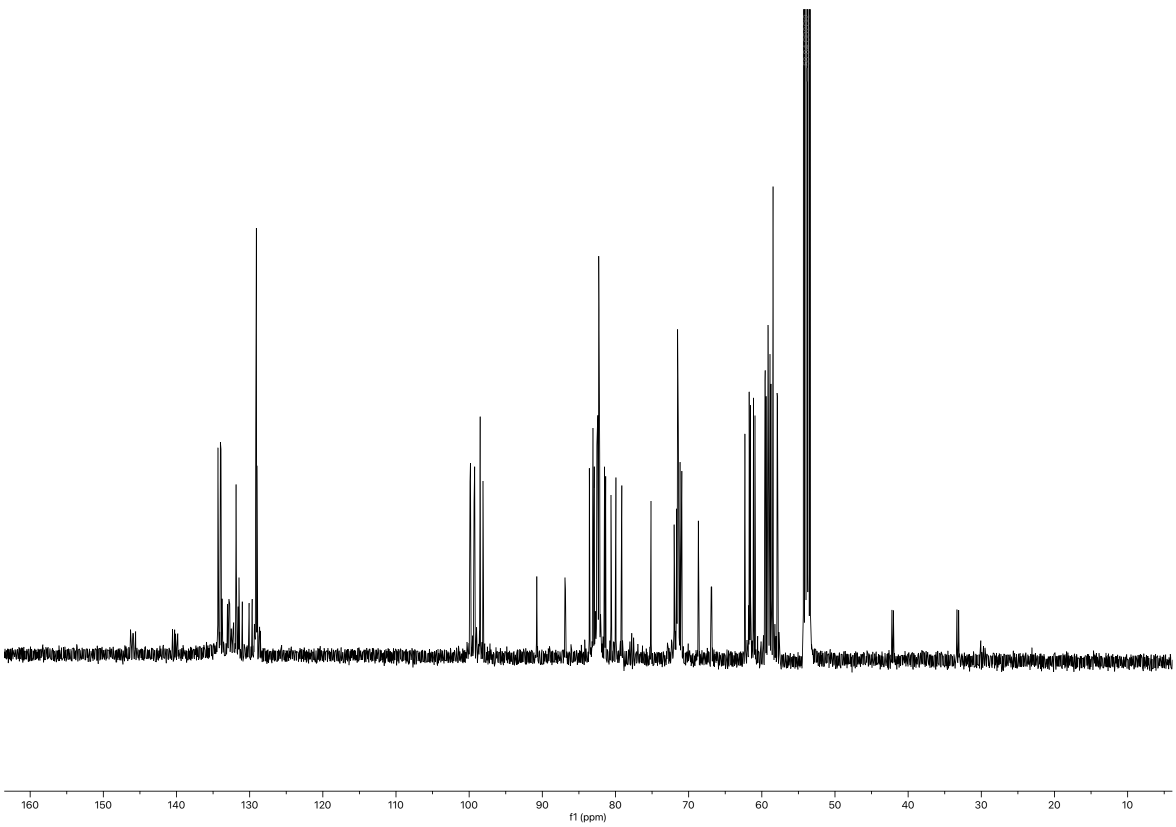

Figure S34. $^{13}C\{^{1}H\}$ NMR (126 MHz, CD_2Cl_2) spectrum of [$NiCl_2$(**L⁴**)].

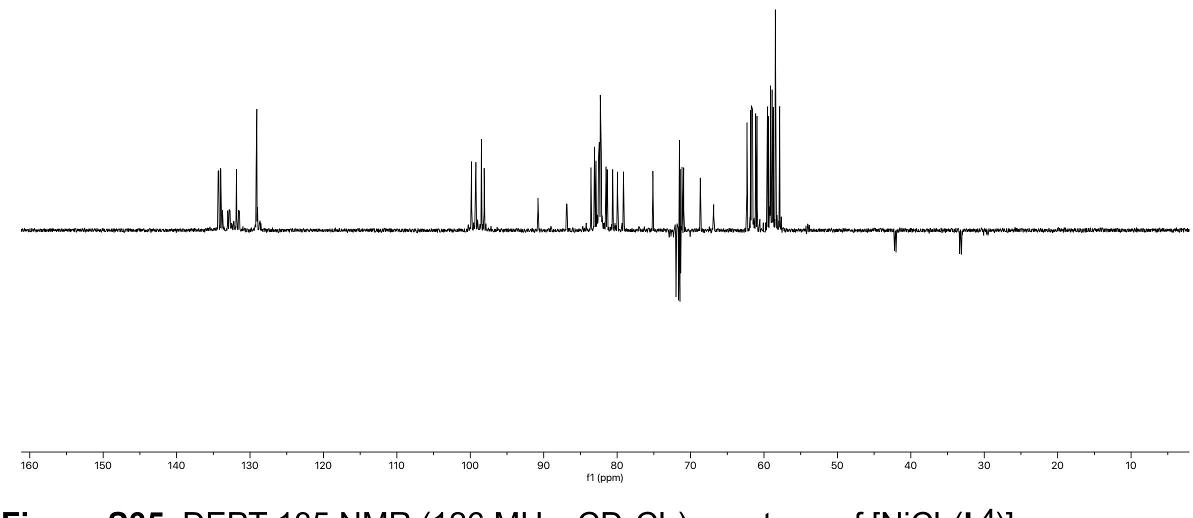

Figure S35. DEPT 135 NMR (126 MHz, CD_2Cl_2) spectrum of [$NiCl_2$(**L⁴**)].

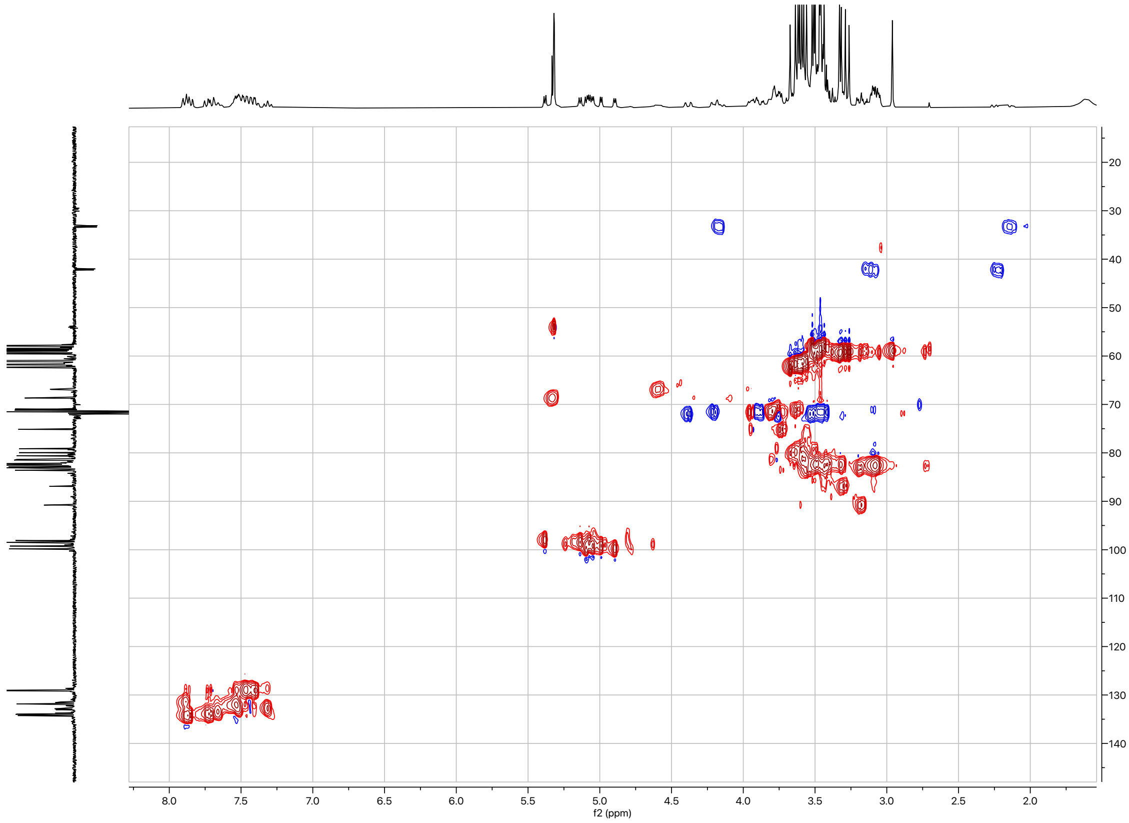

Figure S36. ^{1}H-$^{13}C\{^{1}H\}$ edited HSQC NMR (500 MHz, CD_2Cl_2) spectrum of [$NiCl_2$(**L**^{4})].

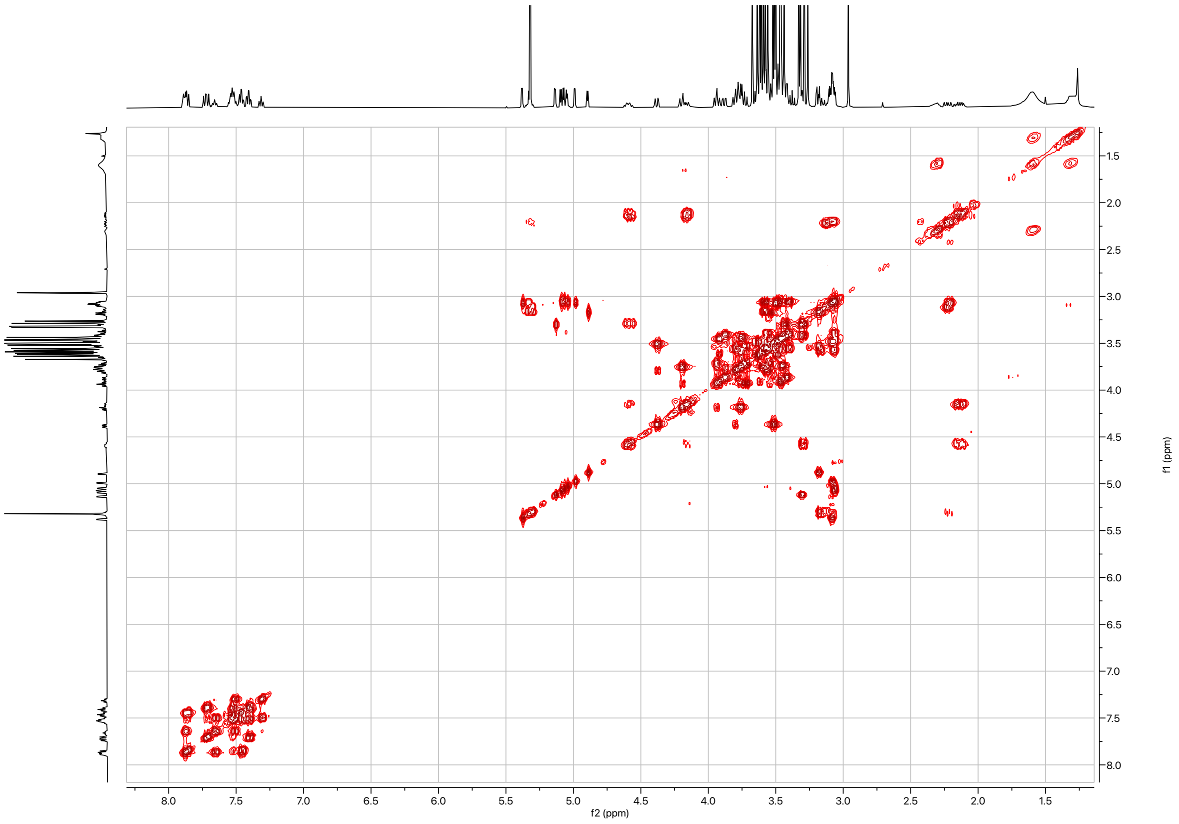

Figure S37. ^{1}H-^{1}H COSY NMR (500 MHz, CD_2Cl_2) spectrum of [$NiCl_2$(**L**^{4})].

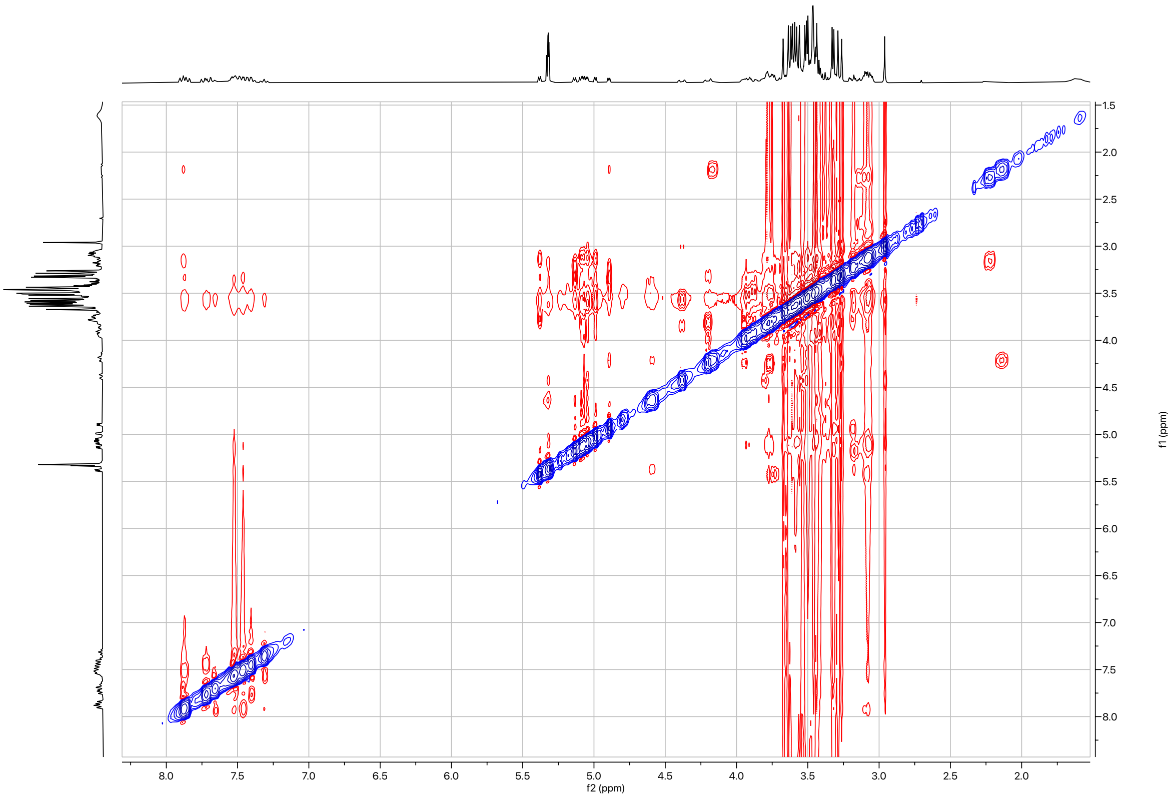

Figure S38. ^{1}H-^{1}H ROESY NMR (500 MHz, CD_2Cl_2) spectrum of [$NiCl_2$(**L^4**)].

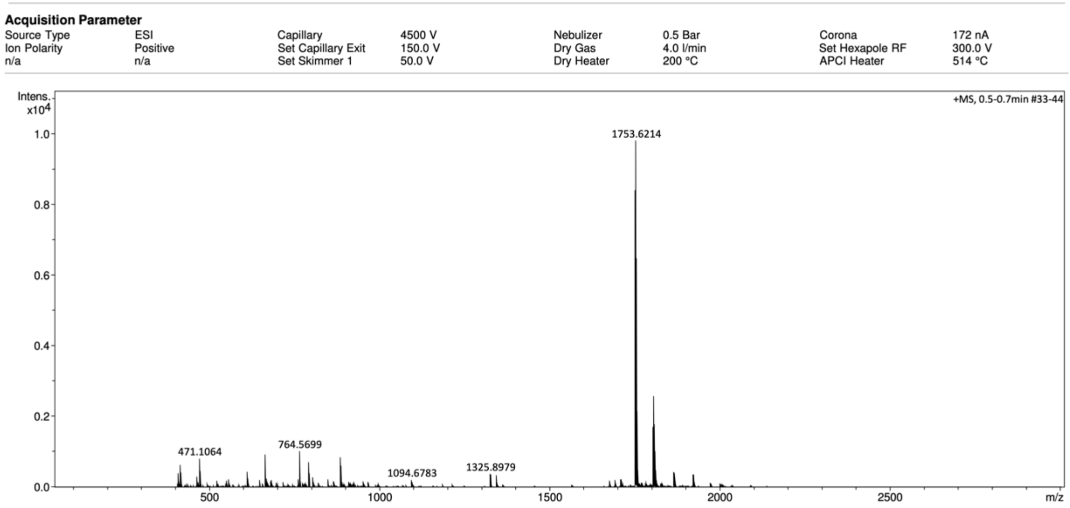

Figure S39. Full ESI-MS spectrum of [$NiCl_2$(**L^4**)].

**Acquisition Parameter**				Set Corrector Fill	74 V
Source Type	ESI	Ion Polarity	Positive	Set Pulsar Pull	799 V
n/a	n/a	Set Capillary Exit	150.0 V	Set Pulsar Push	799 V
Scan Begin	50 m/z	Set Hexapole RF	300.0 V	Set Reflector	1700 V
Scan End	3000 m/z	Set Skimmer 1	50.0 V	Set Flight Tube	8600 V
		Set Hexapole 1	24.3 V	Set Detector TOF	2311 V

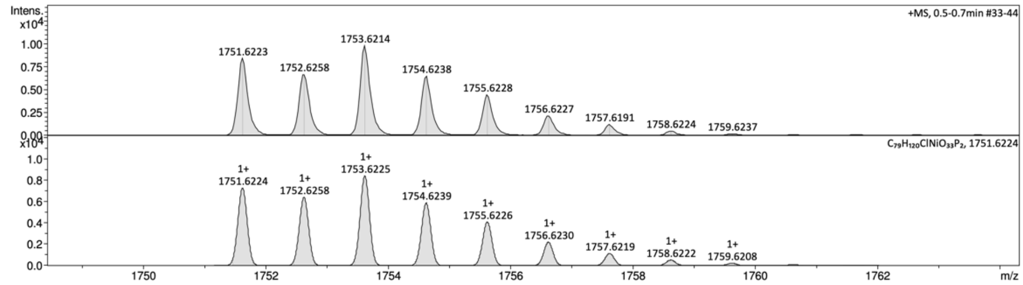

Meas. m/z	#	Ion Formula	m/z	err [ppm]	mSigma	# mSigma	Score	rdb	e⁻ Conf	N-Rule
1751.6223	1	C79H120ClNiO33P2	1751.6224	0.1	35.5	1	100.00	20.5	even	ok

Figure S40. Experimental and simulated partial ESI-MS spectrum of [$NiCl_2$(**L**$^{\mathbf{4}}$)].

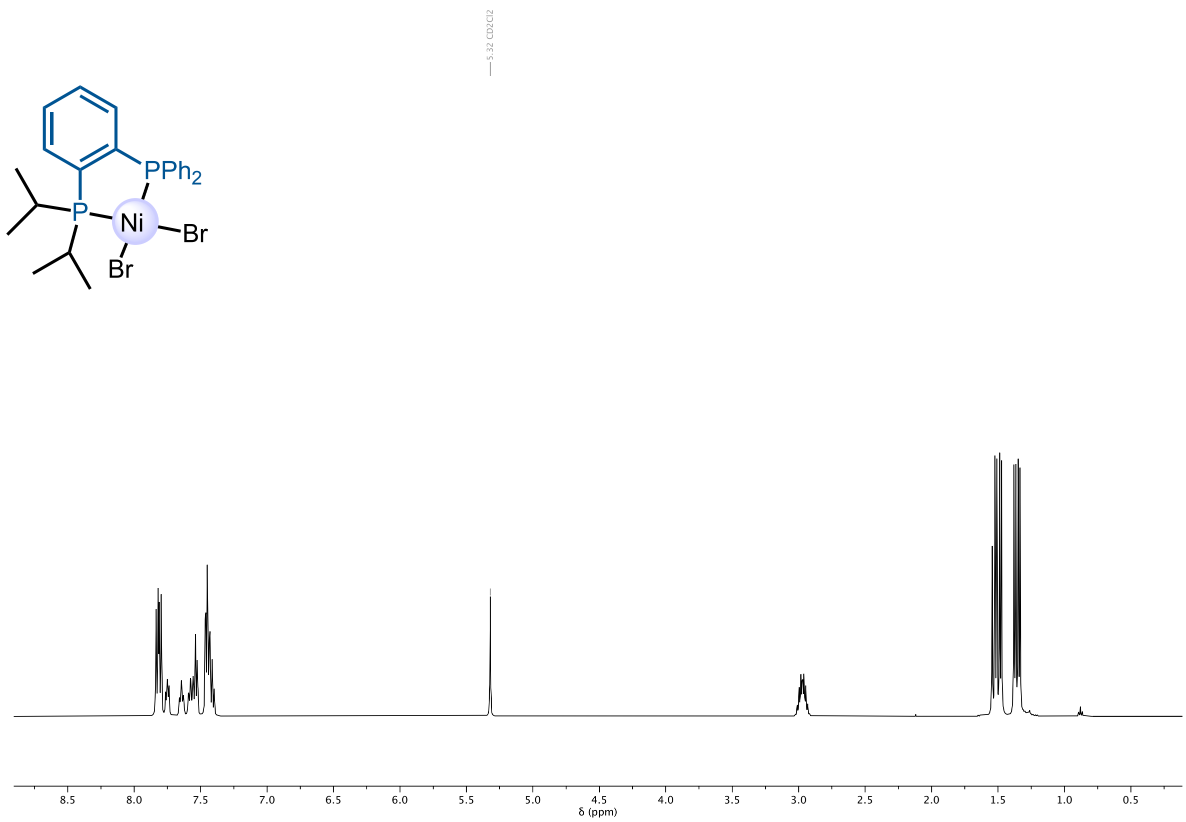

Figure S41. ^{1}H NMR (500 MHz, C_6D_6) spectrum of [$NiBr_2$($\mathbf{L^5}$)].

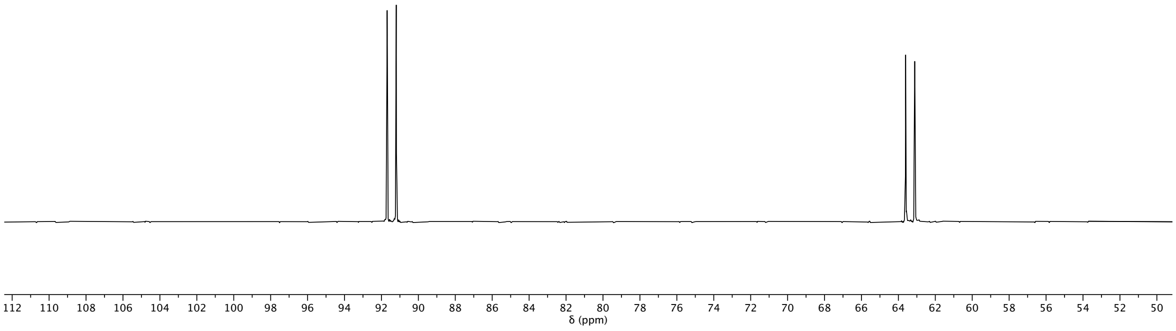

Figure S42. ^{31}P{^{1}H} NMR (202 MHz, C_6D_6) spectrum of [$NiBr_2$($\mathbf{L^5}$)].

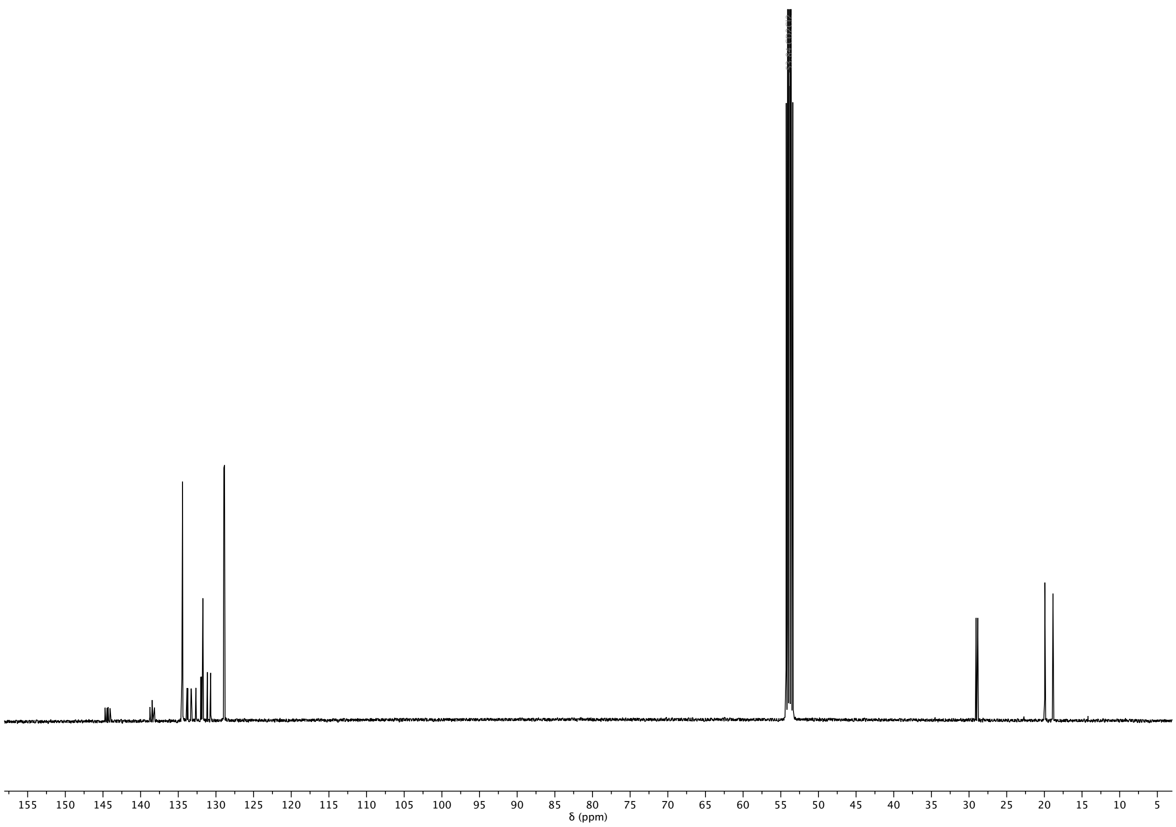

Figure S43. $^{13}C\{^{1}H\}$ NMR (126 MHz, C_6D_6) spectrum of [$NiBr_2$(**L^5**)].

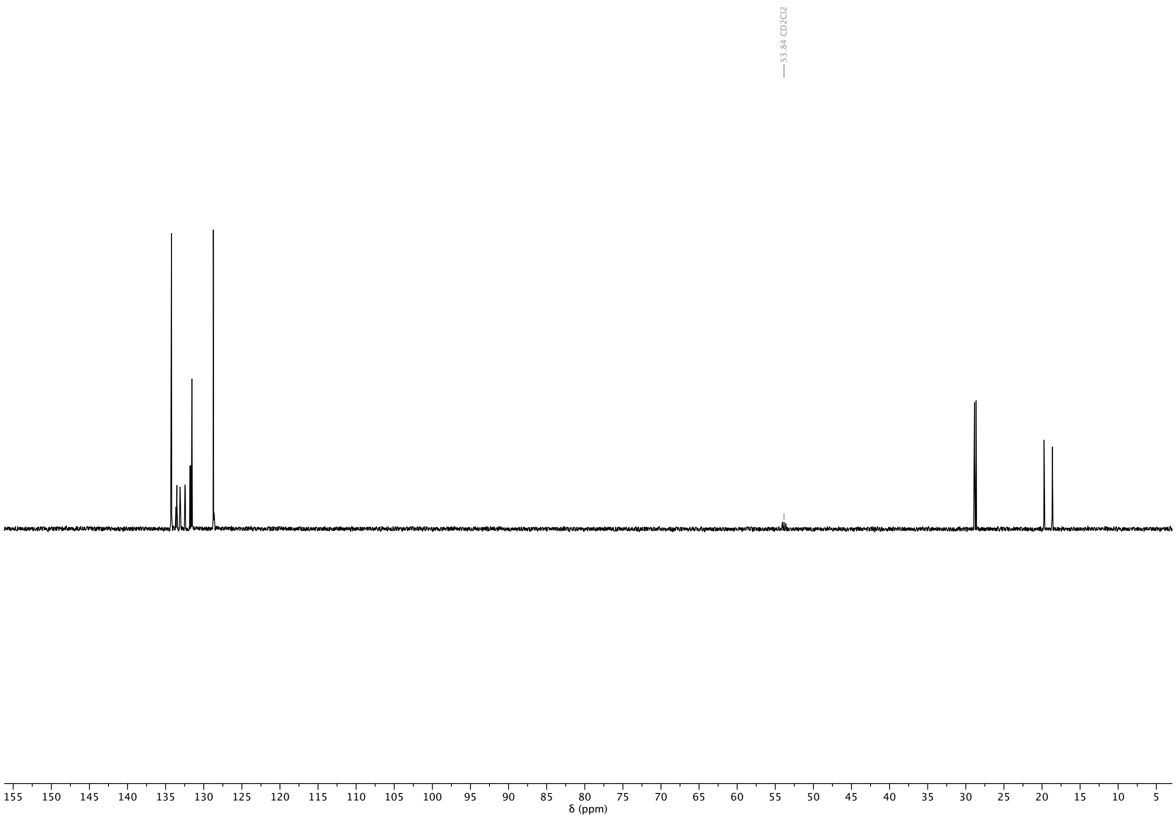

Figure 44. DEPT 135 NMR (126 MHz, C_6D_6) spectrum of [$NiBr_2$(**L^5**)].

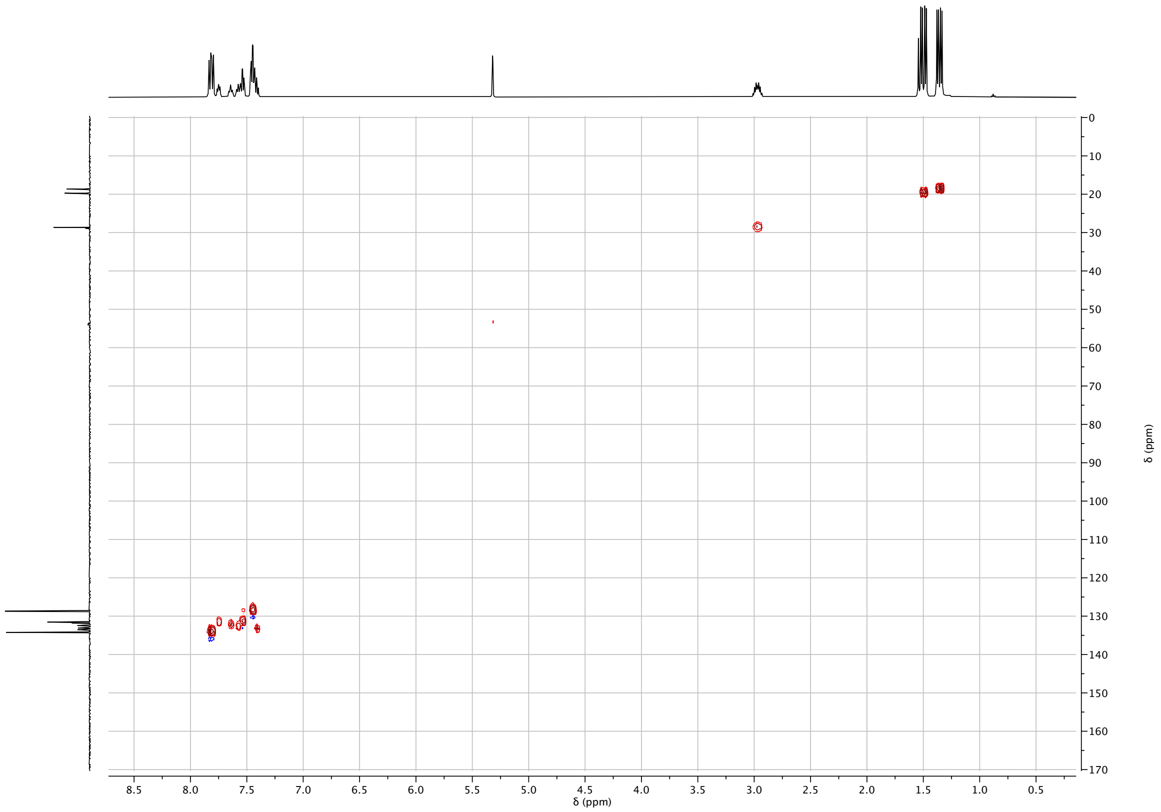

Figure S45. ^{1}H-$^{13}C\{^{1}H\}$ edited HSQC NMR (500 MHz, C_6D_6) spectrum of [$NiBr_2$(**L^5**)].

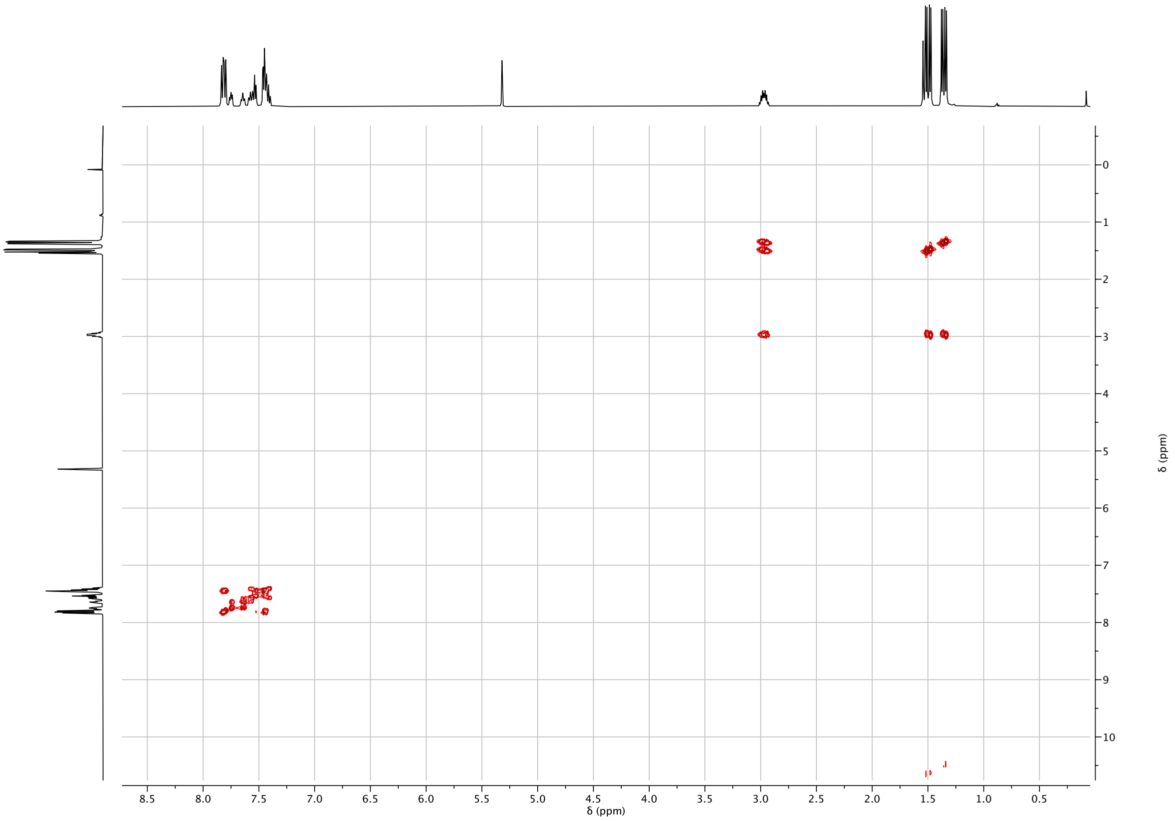

Figure S46. ^{1}H-^{1}H COSY NMR (500 MHz, C_6D_6) spectrum of [$NiBr_2$(**L^5**)].

Acquisition Parameter

				Set Corrector Fill	50.9 V
Source Type	ESI	Ion Polarity	Positive	n/a	n/a
n/a	n/a	n/a	n/a	n/a	n/a
Scan Begin	50 m/z	n/a	n/a	Set Reflector	1800.0 V
Scan End	3000 m/z	n/a	n/a	Set Flight Tube	8600.0 V
		n/a	n/a	Set Detector TOF	2008.9 V

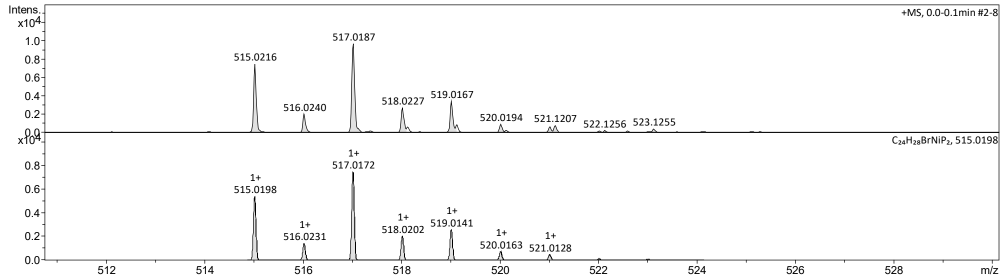

Meas. m/z	#	Ion Formula	m/z	err [ppm]	Mean err [ppm]	rdb	N-Rule	e⁻ Conf	mSigma	Std I	Std Mean m/z	Std I VarNorm	Std m/z Diff	Std Comb Dev
515.021566	1	C24H28BrNiP2	515.019756	-3.5	2284.1	11.5	ok	even	19.8	17.6	n.a.	n.a.	n.a.	n.a.

Figure S47. Experimental and simulated partial HR-MS spectrum of [$NiBr_2$(**L^5**)].

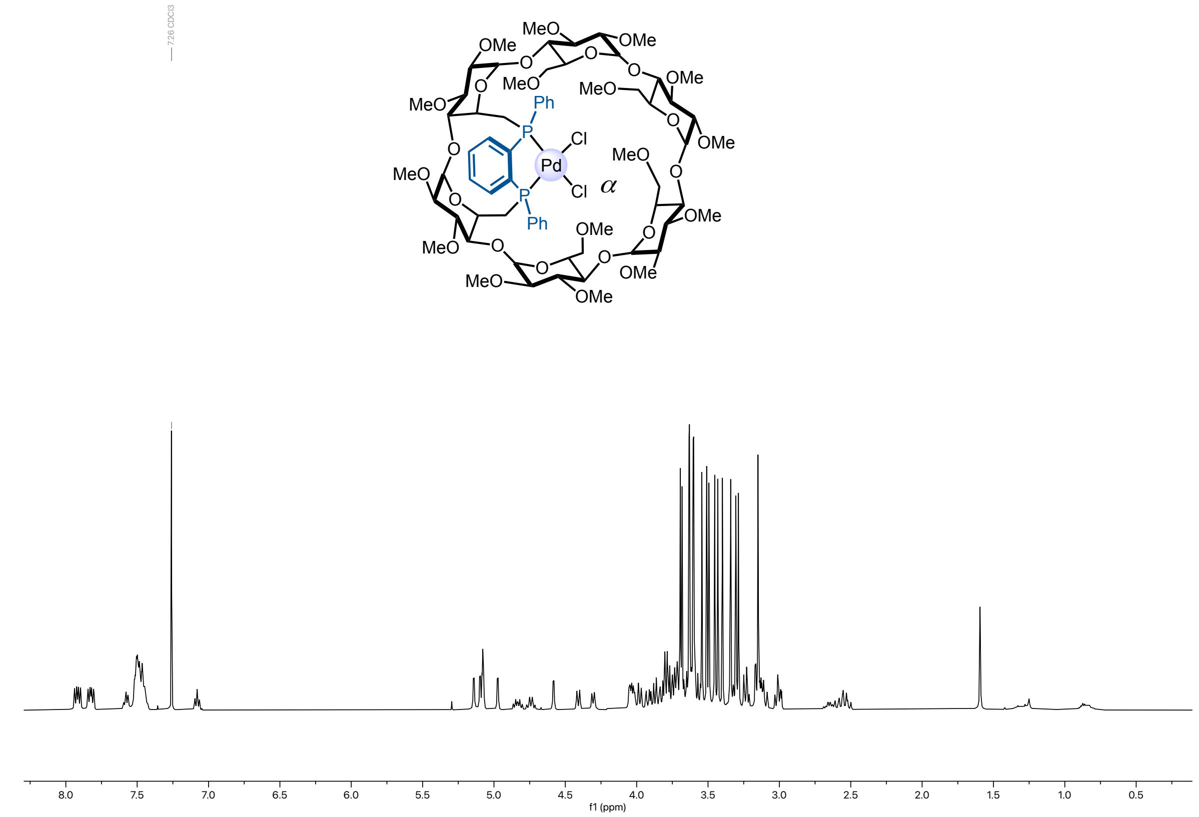

Figure S48. ^{1}H NMR (500 MHz, $CDCl_3$) spectrum of [$PdCl_2$(**L^1**)].

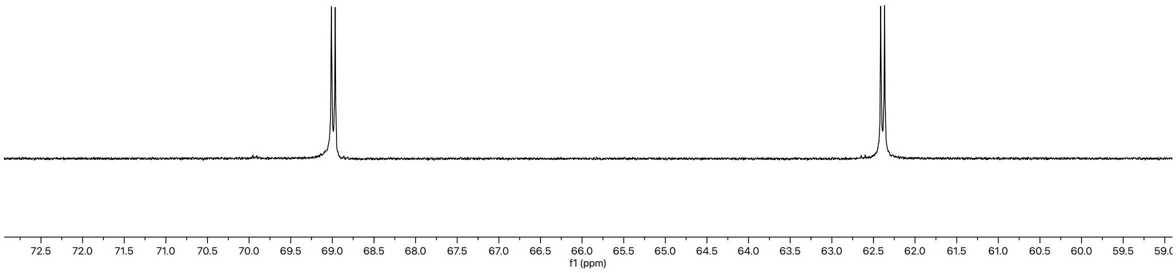

Figure S49. $^{31}P\{^{1}H\}$ NMR (202 MHz, $CDCl_3$) spectrum of [$PdCl_2$(**L^1**)].

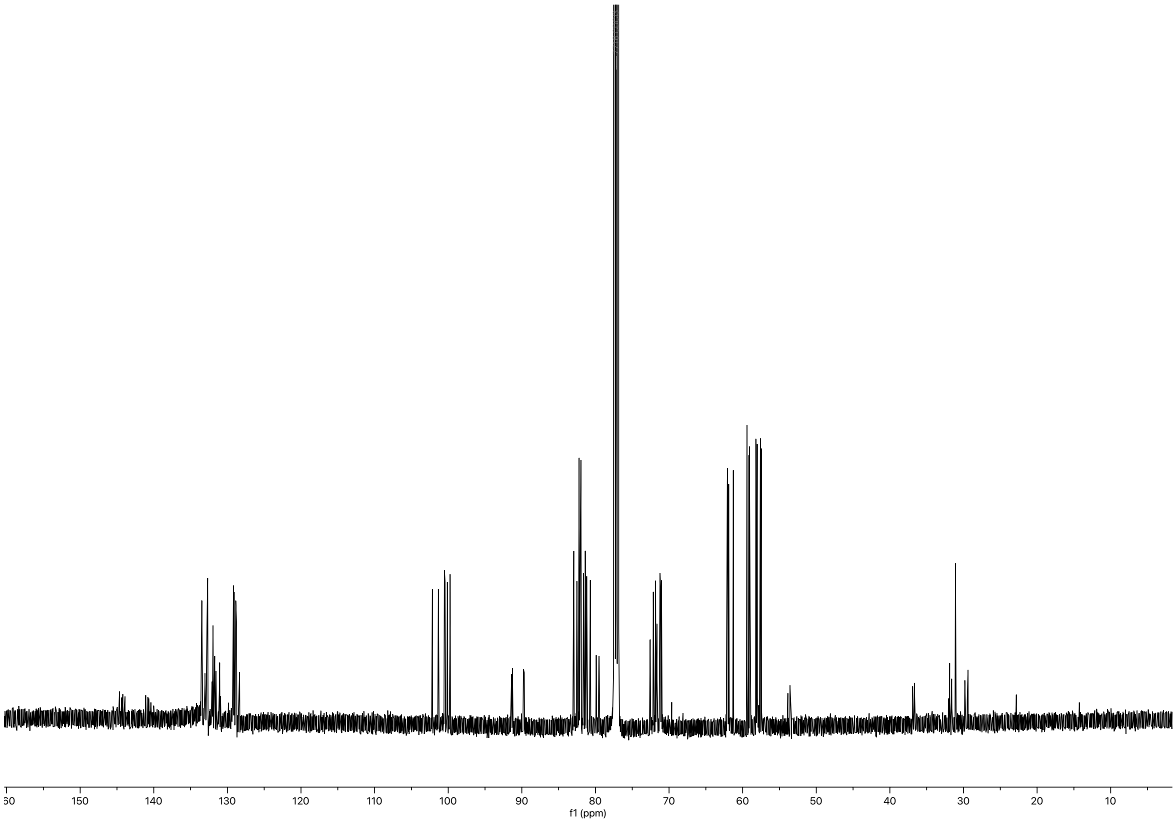

Figure S50. $^{13}C\{^{1}H\}$ NMR (126 MHz, $CDCl_3$) spectrum of [$PdCl_2$(**L^1**)].

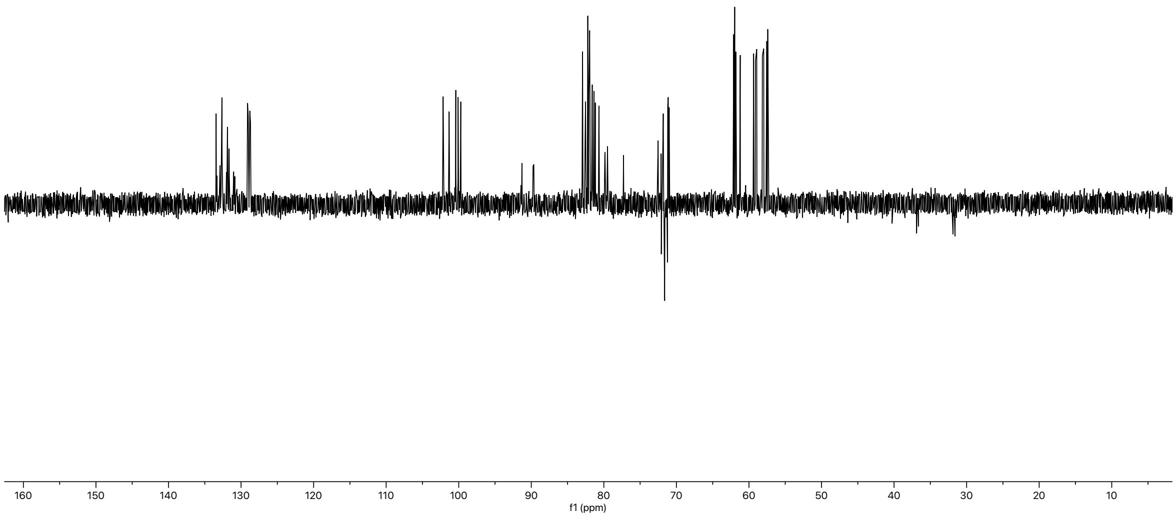

Figure S51. DEPT 135 NMR (126 MHz, $CDCl_3$) spectrum of [$PdCl_2$(**L^1**)].

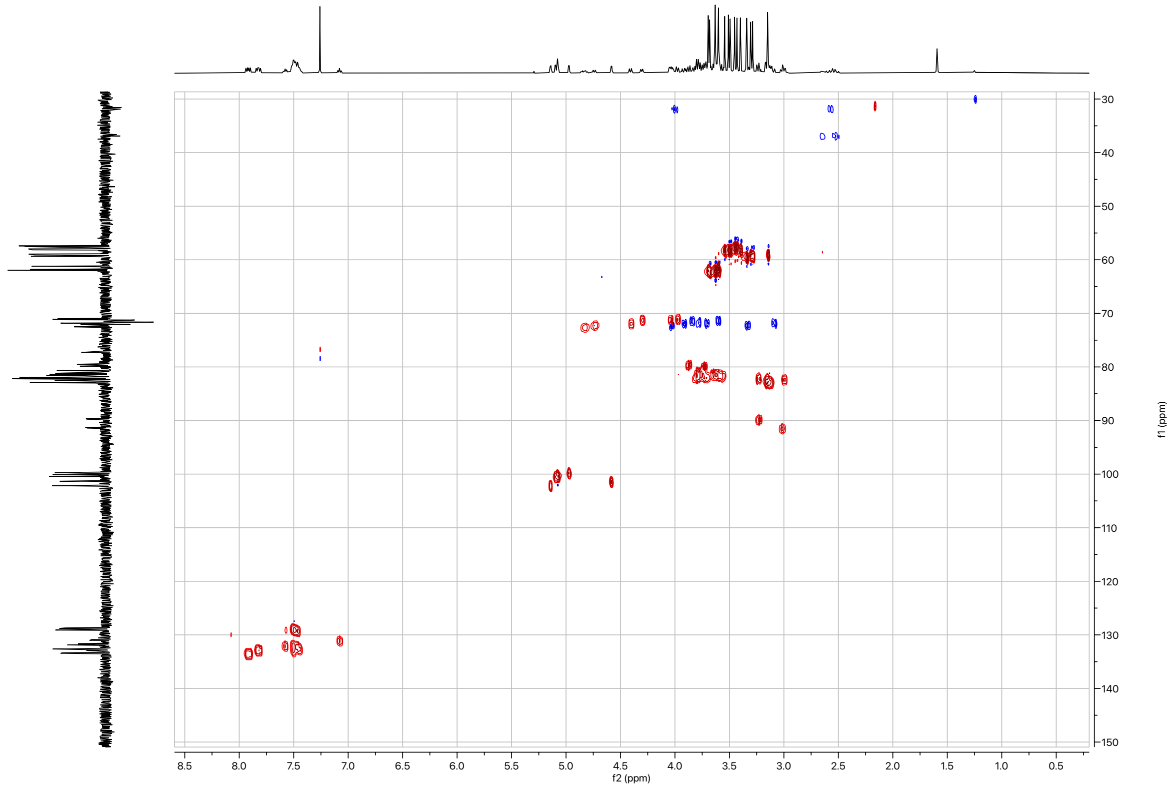

Figure S52. ^{1}H-^{13}C{^{1}H} edited HSQC NMR (500 MHz, $CDCl_3$) spectrum of [$PdCl_2$(**L^1**)].

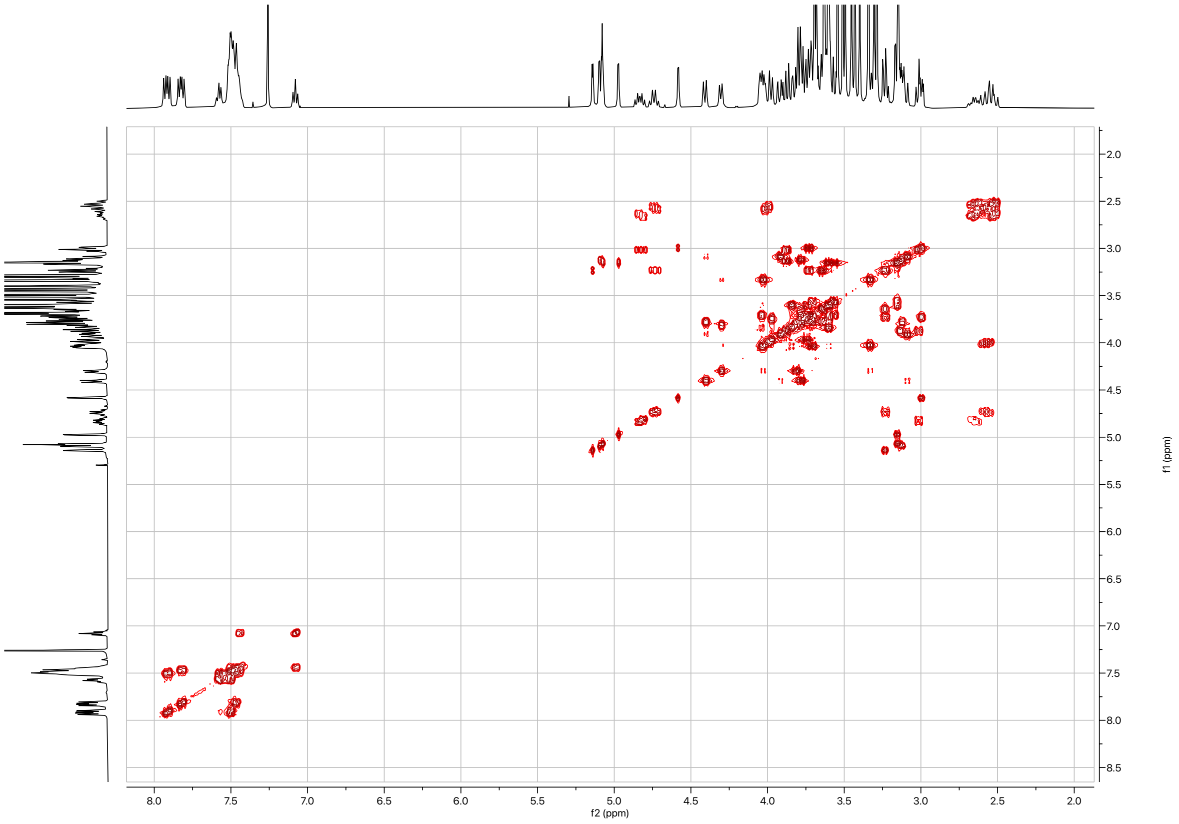

Figure S53. ^{1}H-^{1}H COSY NMR (500 MHz, $CDCl_3$) spectrum of [$PdCl_2$(**L^1**)].

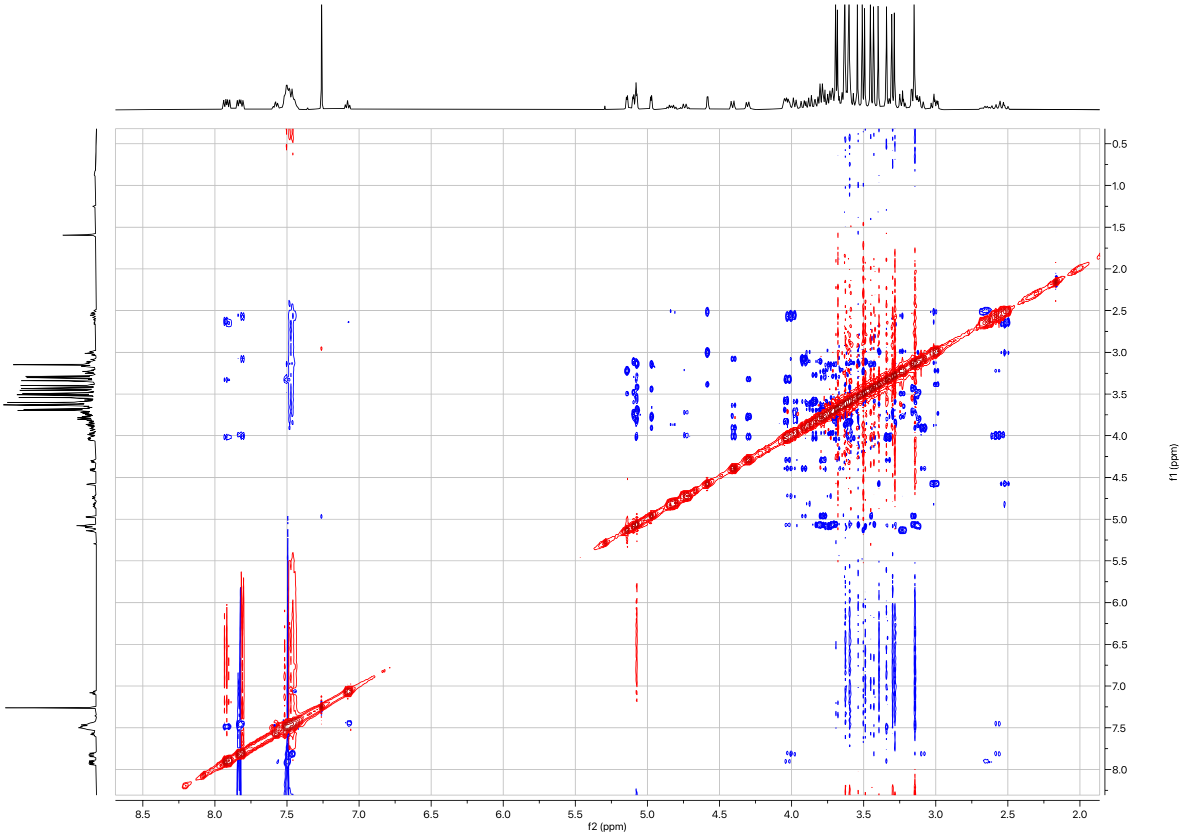

Figure S54. ^{1}H-^{1}H ROESY NMR (500 MHz, $CDCl_3$) spectrum of [$PdCl_2$(**L**^{1})].

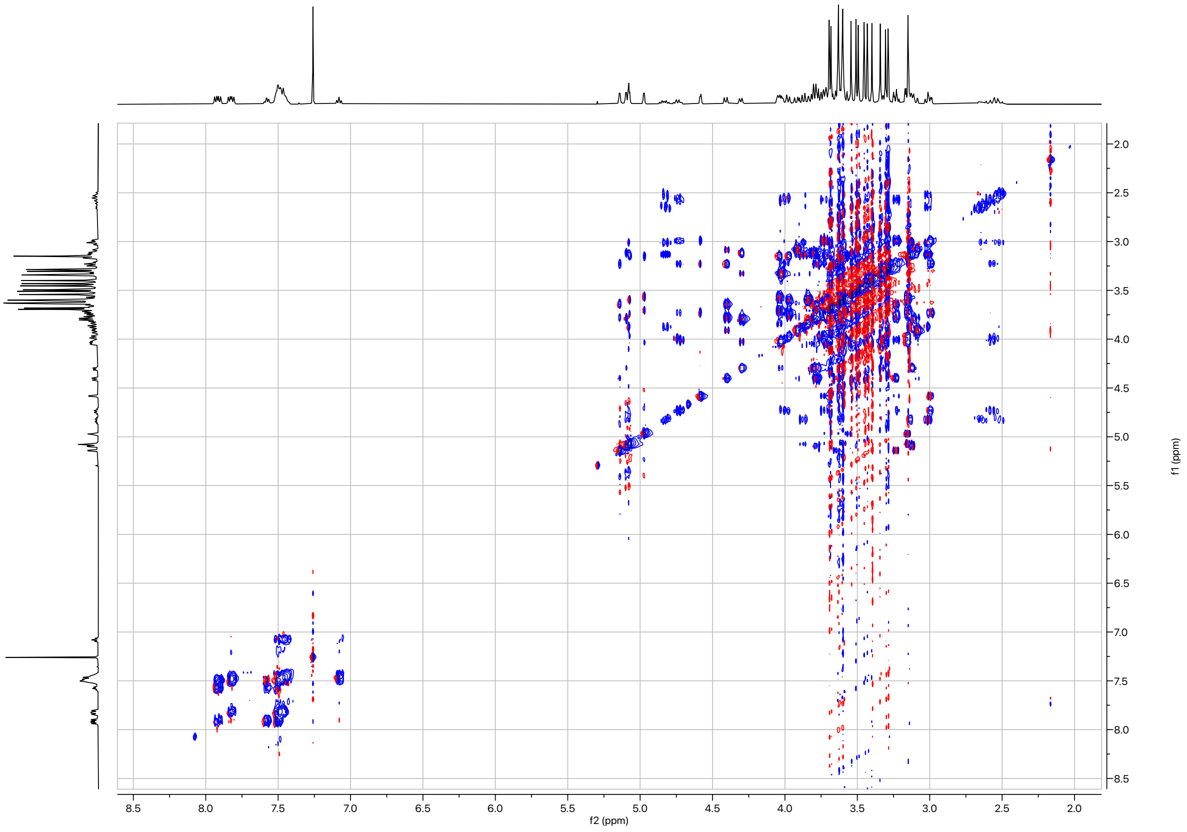

Figure S55. ^{1}H-^{1}H TOCSY NMR (500 MHz, $CDCl_3$) spectrum of [$PdCl_2$(**L**^{1})].

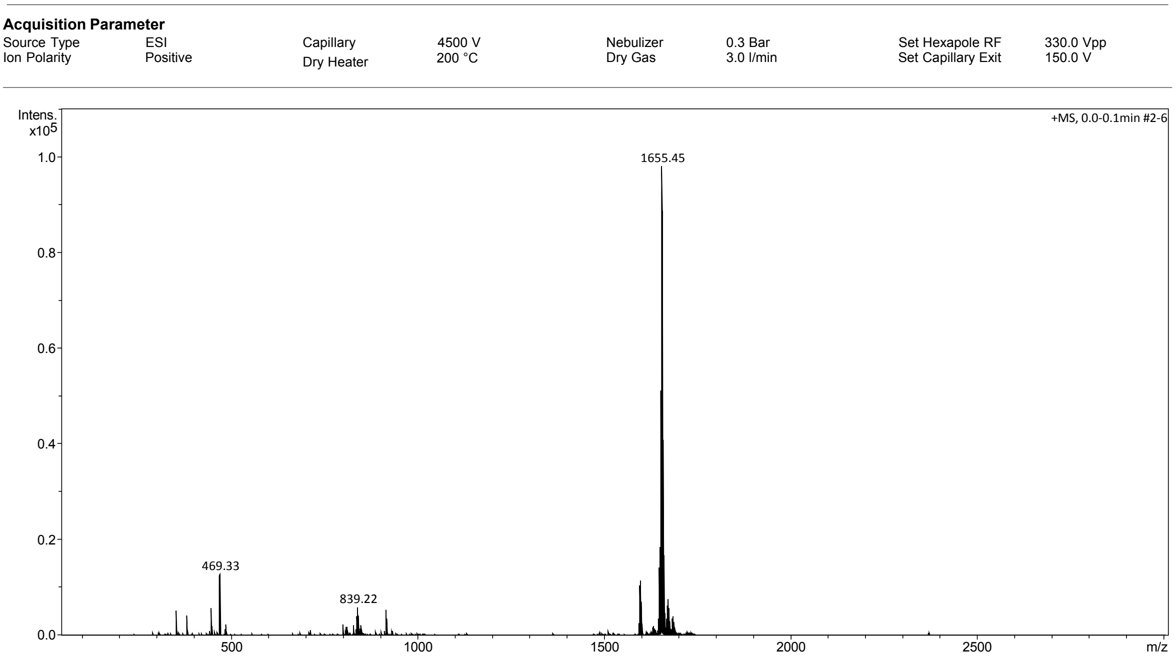

Figure S56. Full ESI-MS spectrum of [$PdCl_2$(**L[1]**)].

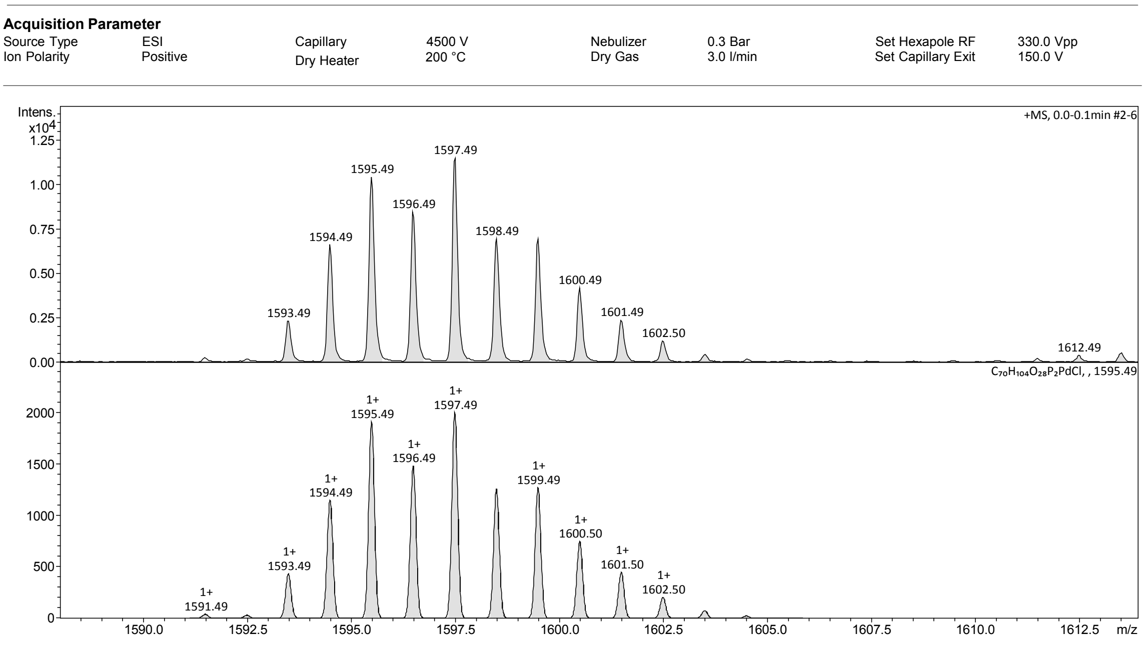

Figure S57. Experimental and simulated partial ESI-MS spectrum of [$PdCl_2$(**L[1]**)].

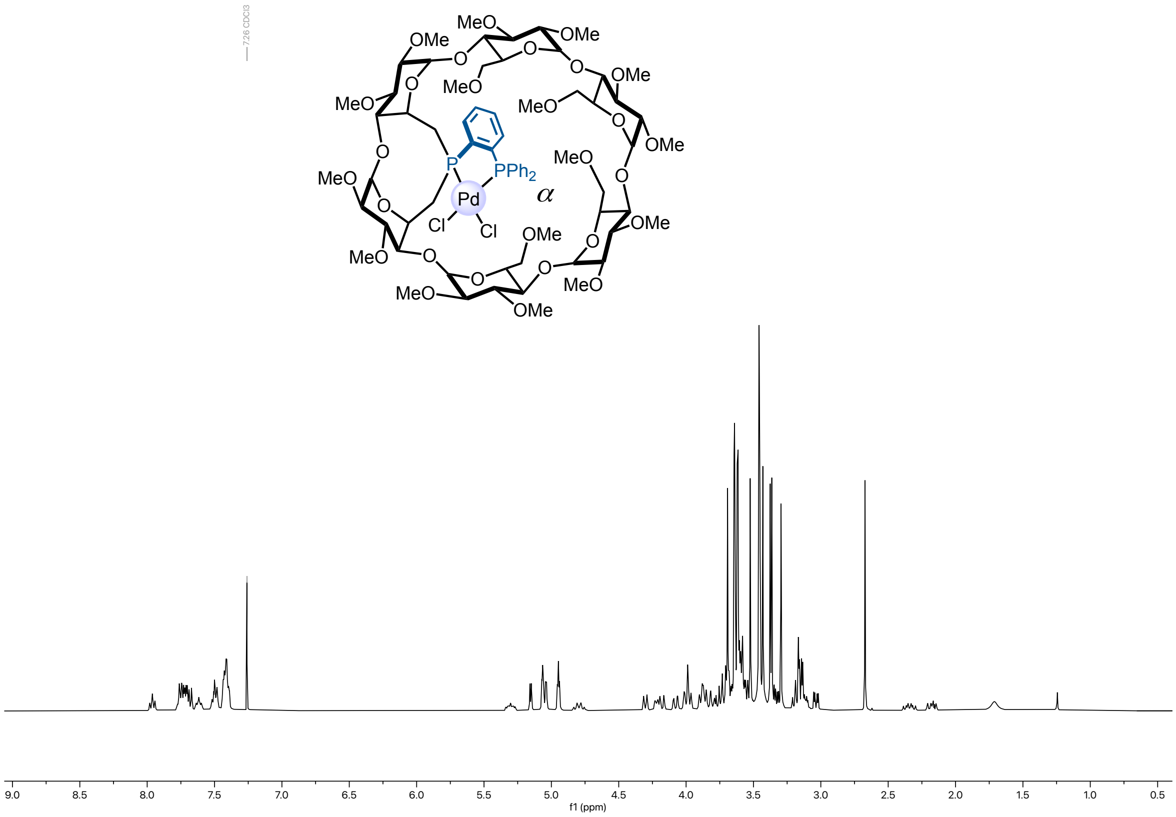

Figure S58. ^{1}H NMR (400 MHz, $CDCl_3$) spectrum of [$PdCl_2$(**L**^{3})].

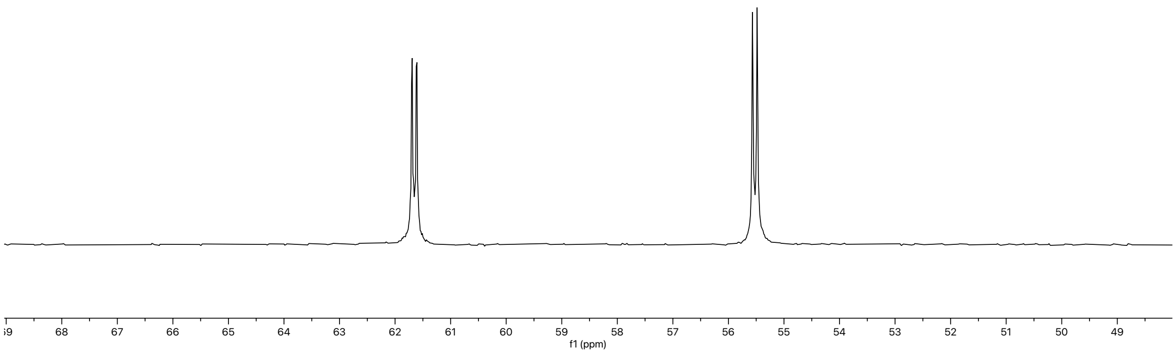

Figure S59. $^{31}P\{^{1}H\}$ NMR (162 MHz, $CDCl_3$) spectrum of [$PdCl_2$(**L**^{3})].

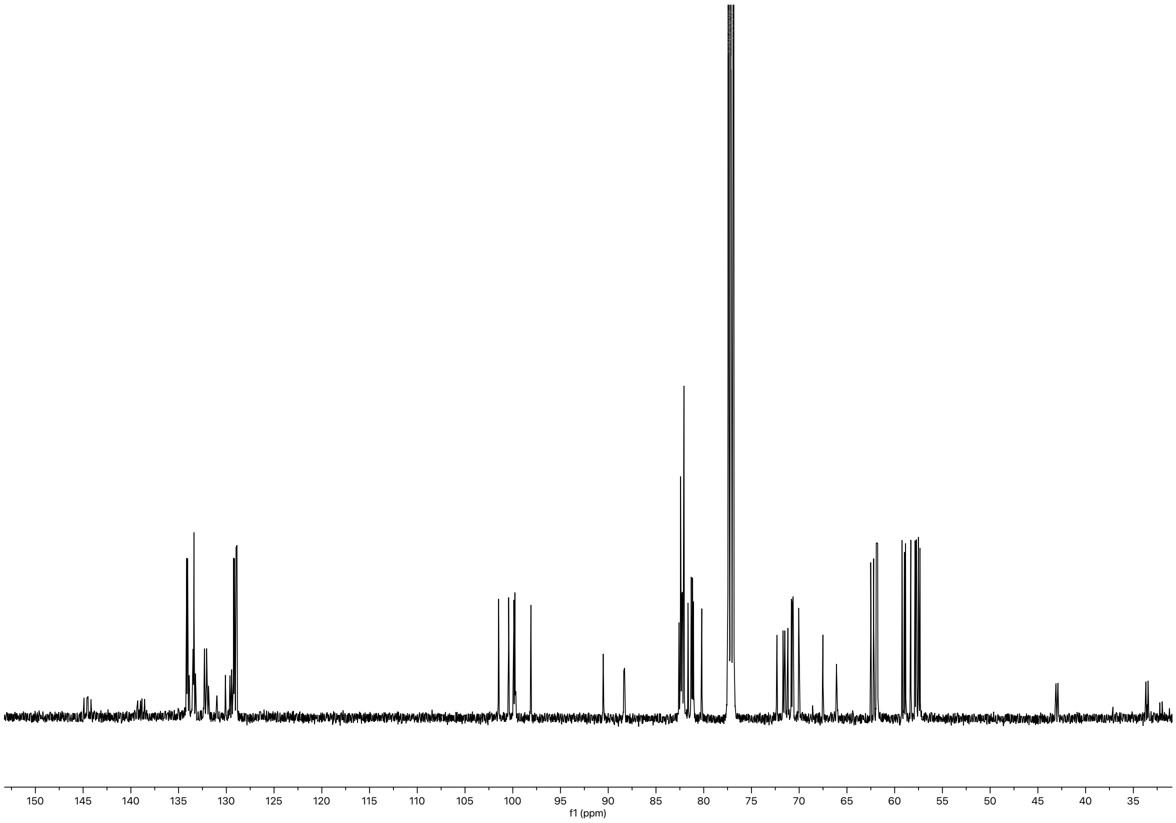

Figure S60. $^{13}C\{^{1}H\}$ NMR (126 MHz, $CDCl_3$) spectrum of [$PdCl_2$(**L^3**)].

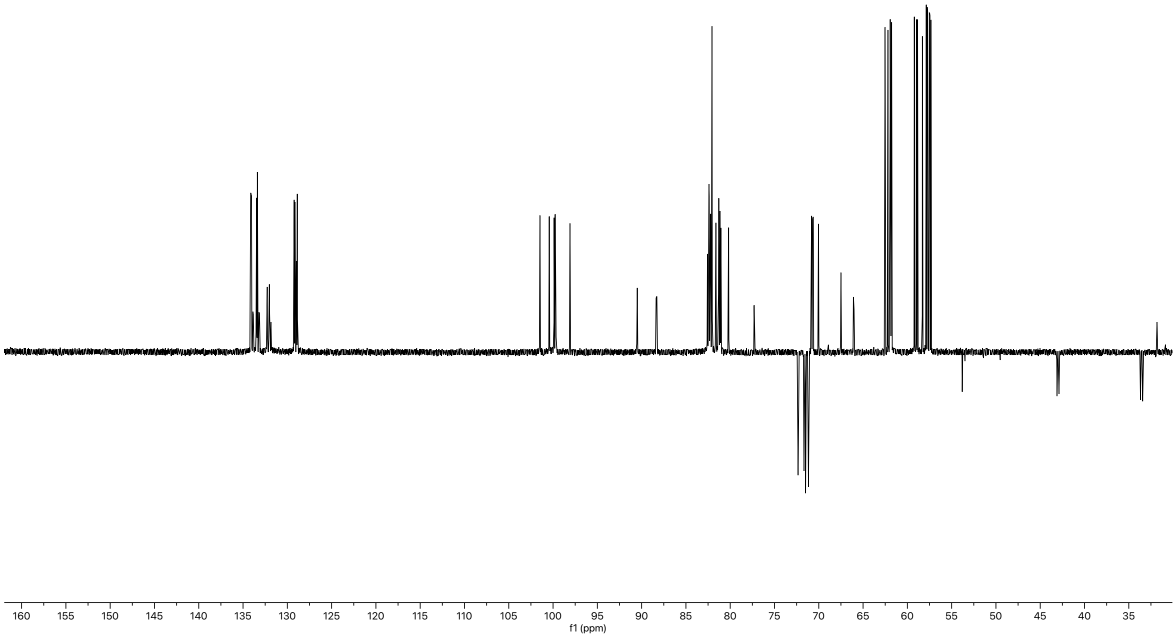

Figure S61. DEPT 135 NMR (126 MHz, $CDCl_3$) spectrum of [$PdCl_2$(**L^3**)].

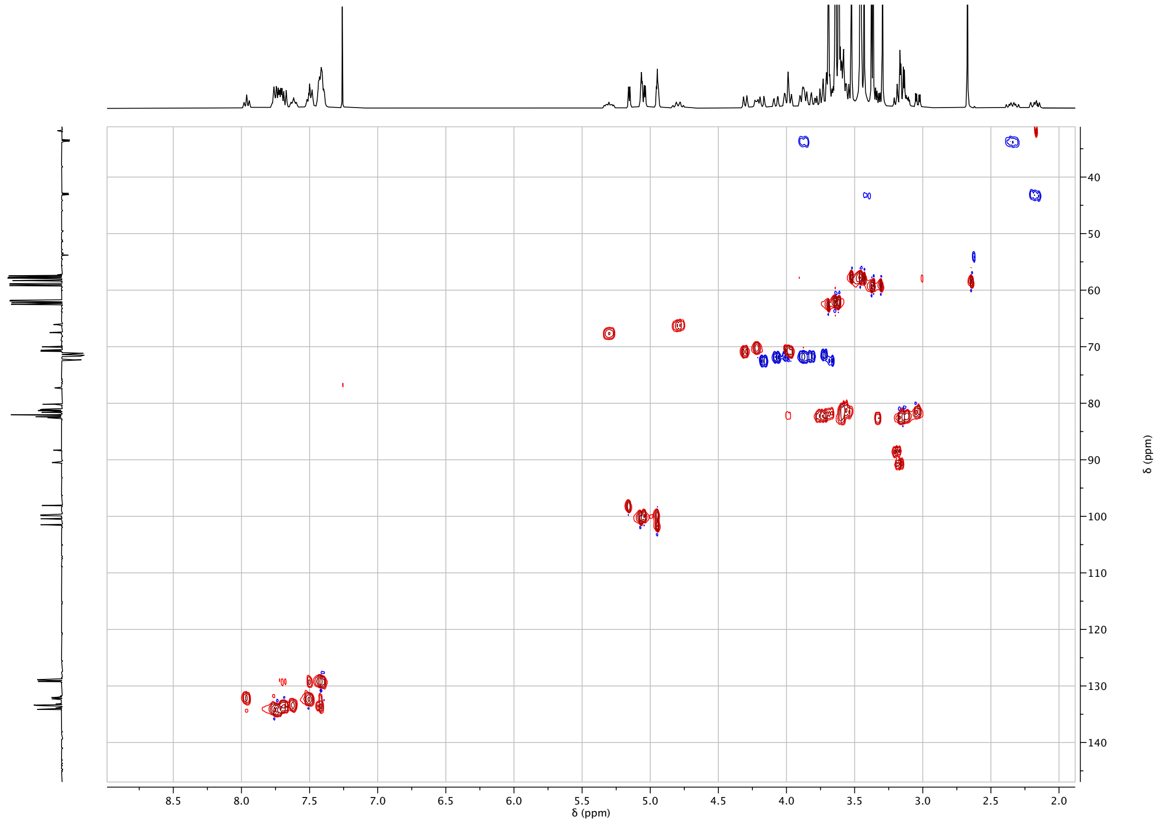

Figure S62. ^{1}H-$^{13}C\{^{1}H\}$ edited HSQC NMR (500 MHz, $CDCl_3$) spectrum of $[PdCl_2(\mathbf{L^3})]$.

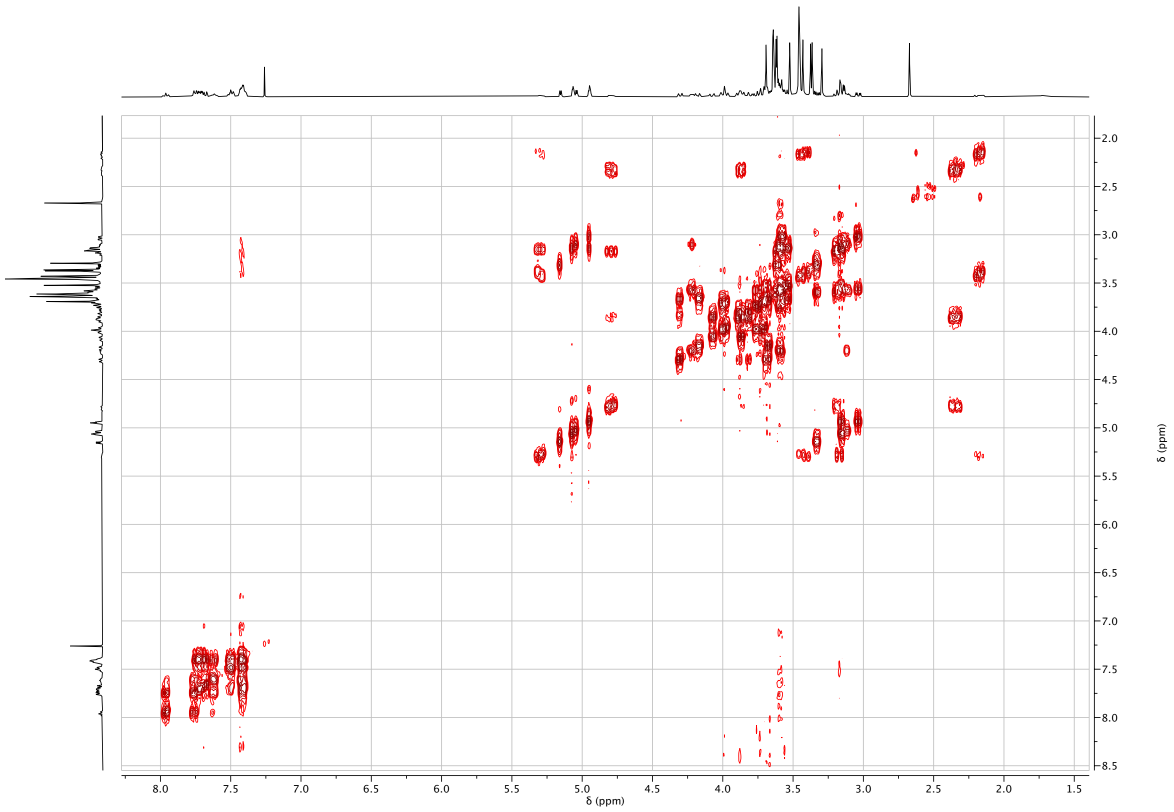

Figure S63. ^{1}H-^{1}H COSY NMR (500 MHz, $CDCl_3$) spectrum of $[PdCl_2(\mathbf{L^3})]$.

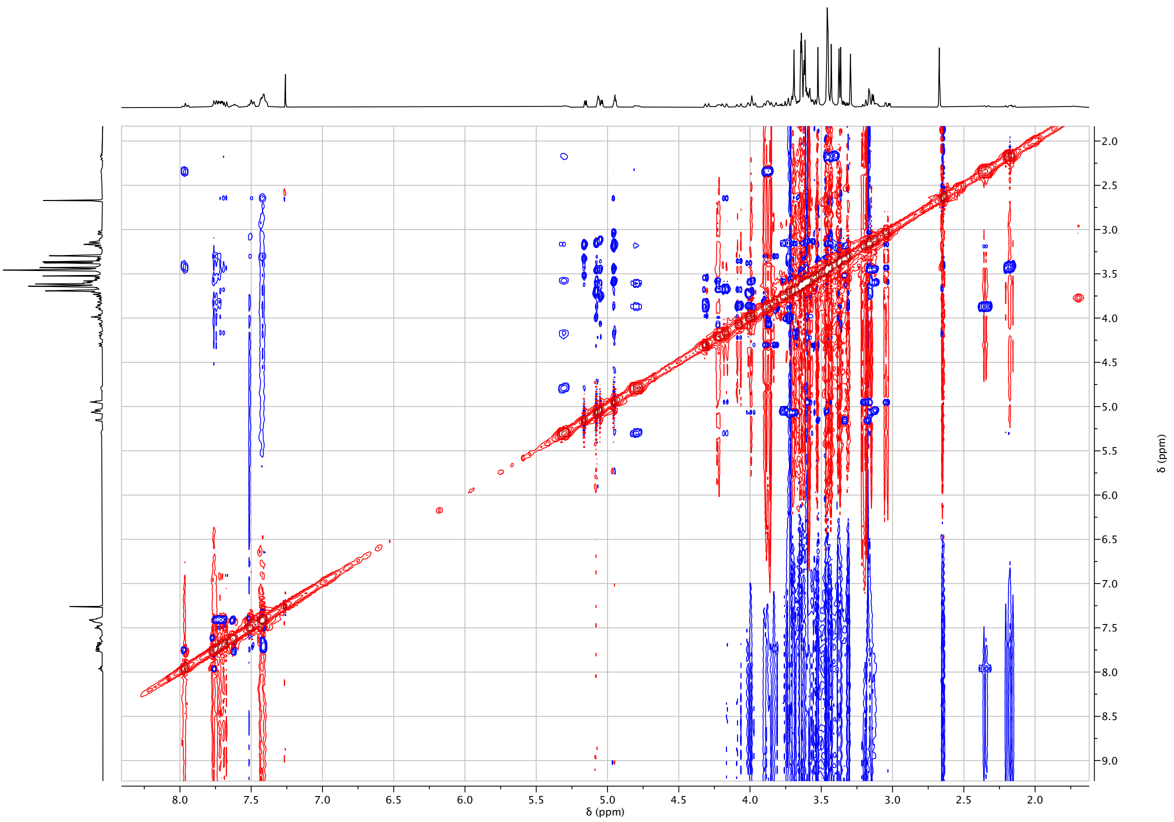

Figure S64. ^{1}H-^{1}H ROESY NMR (500 MHz, $CDCl_3$) spectrum of [$PdCl_2$(**L^3**)].

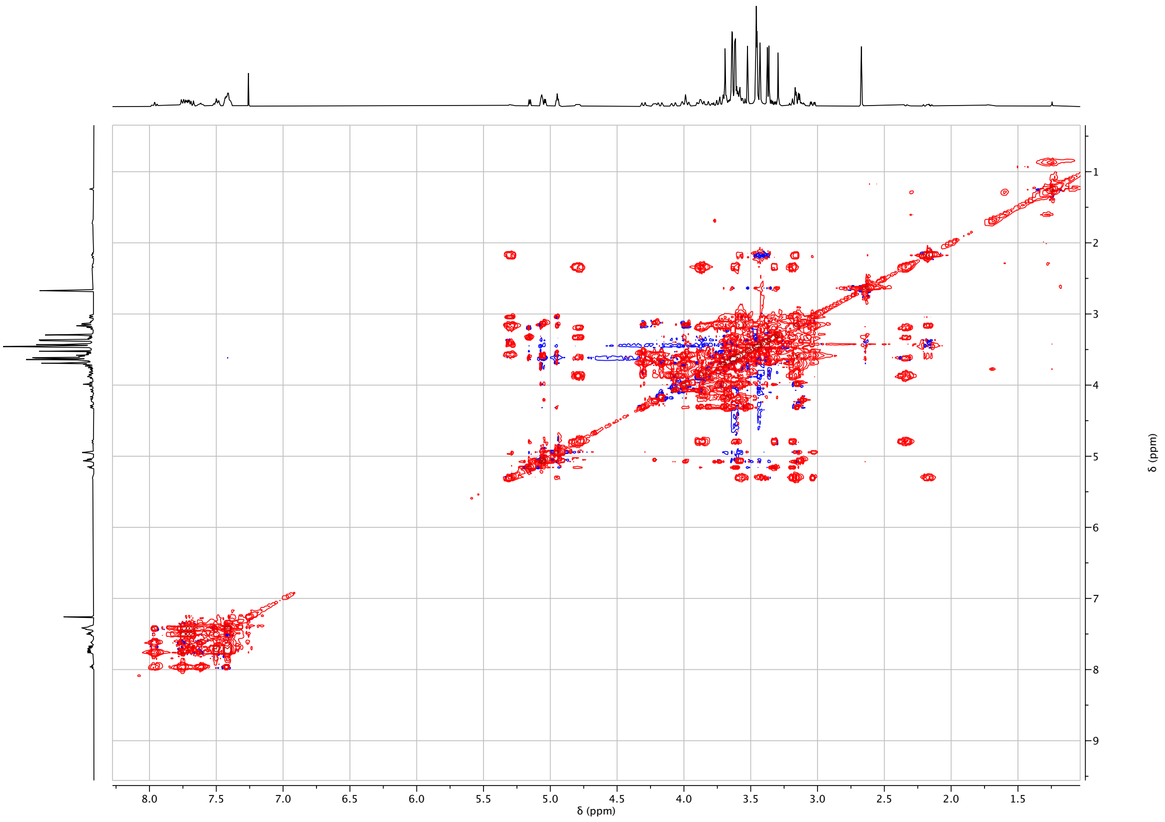

Figure S65. ^{1}H-^{1}H TOCSY NMR (500 MHz, $CDCl_3$) spectrum of [$PdCl_2$(**L^3**)].

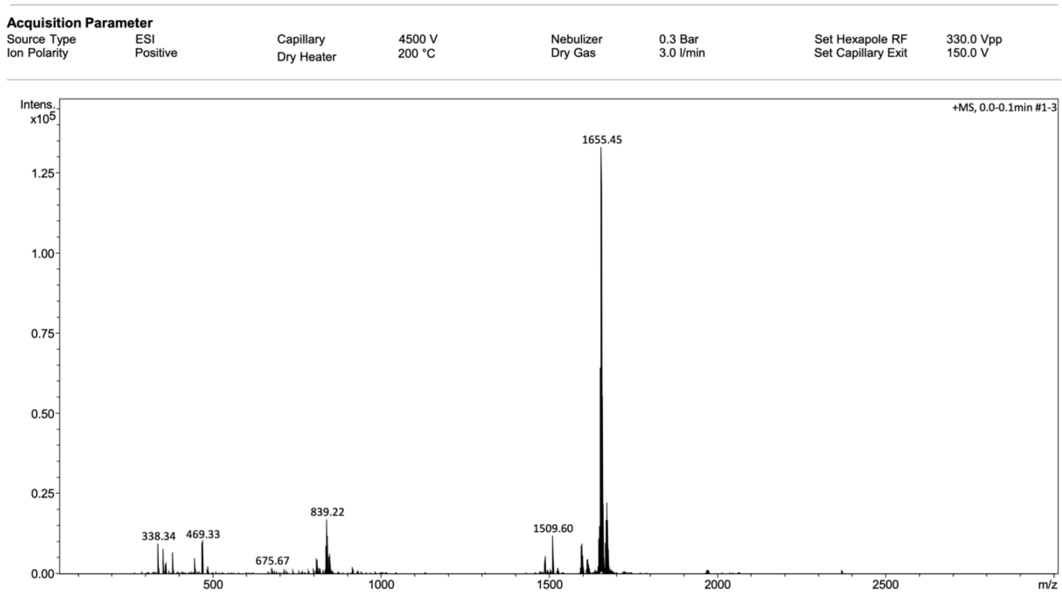

Figure S66. Full ESI-MS spectrum of [$PdCl_2$(**L**^{3})].

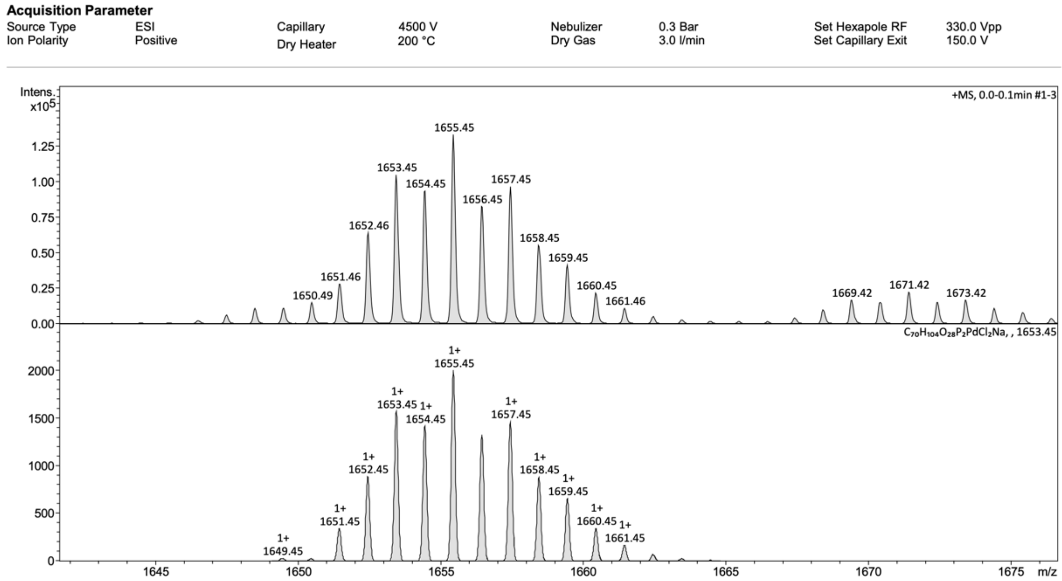

Figure S67. Experimental and simulated partial ESI-MS spectrum of [$PdCl_2$(**L**^{3})].

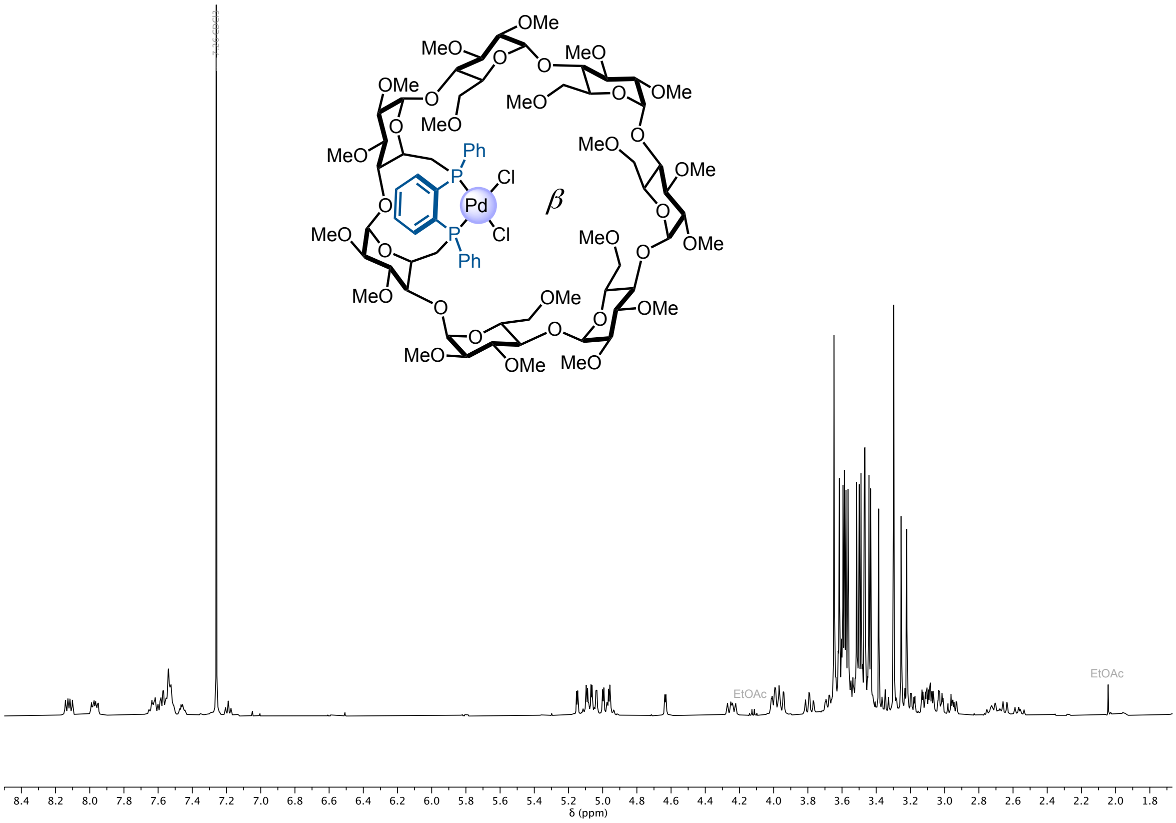

Figure S68. ^{1}H NMR (500 MHz, C_6D_6) spectrum of [$PdCl_2$(**L^2**)].

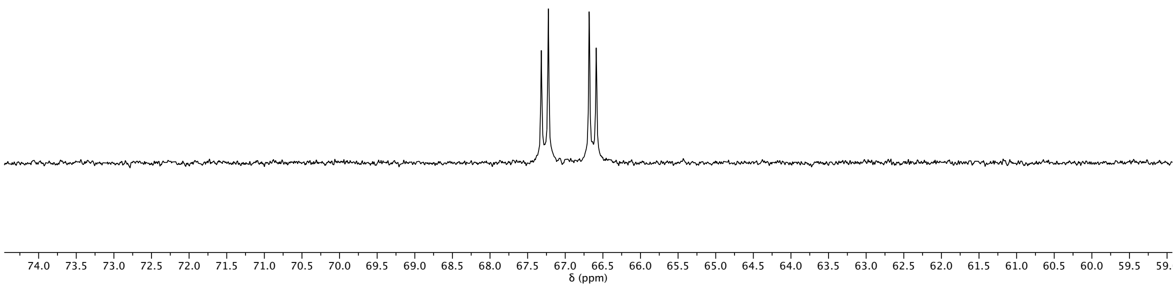

Figure S69. $^{31}P\{^{1}H\}$ NMR (121 MHz, C_6D_6) spectrum of [$PdCl_2$(**L^2**)].

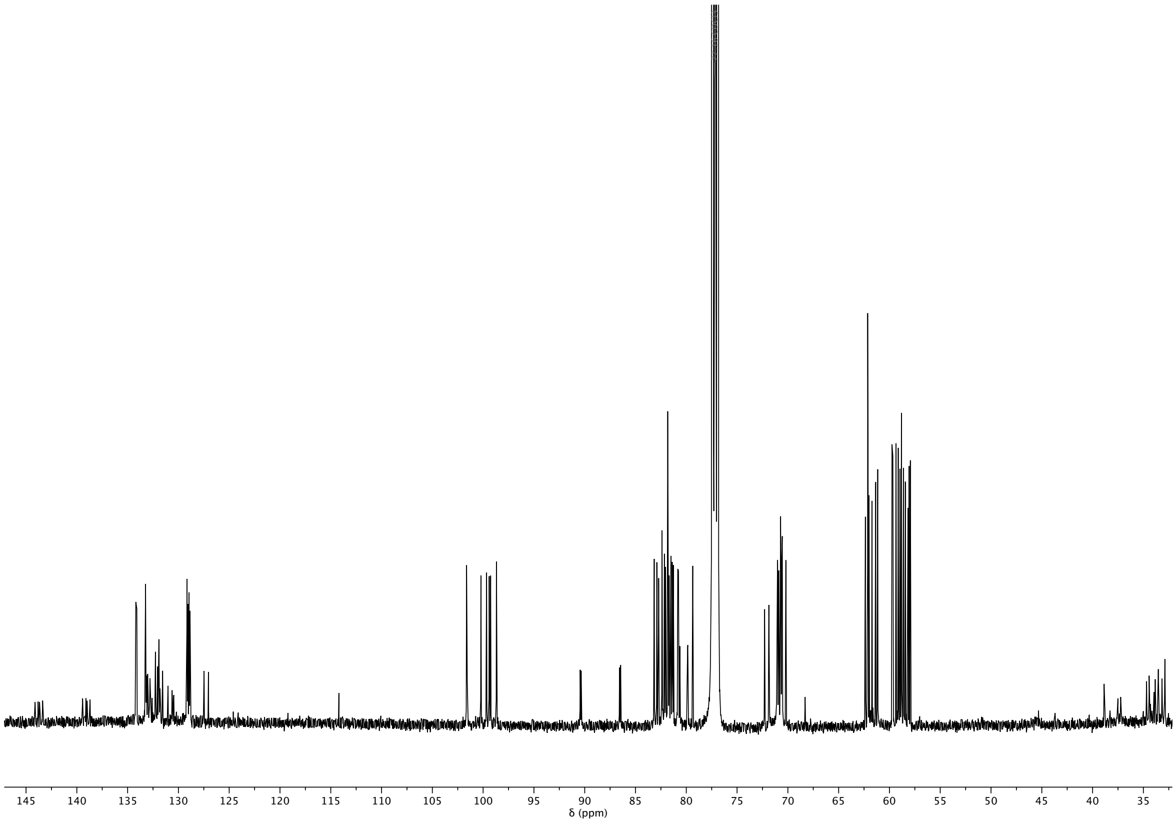

Figure S70. $^{13}C\{^{1}H\}$ NMR (126 MHz, C_6D_6) spectrum of [$PdCl_2$(**L²**)].

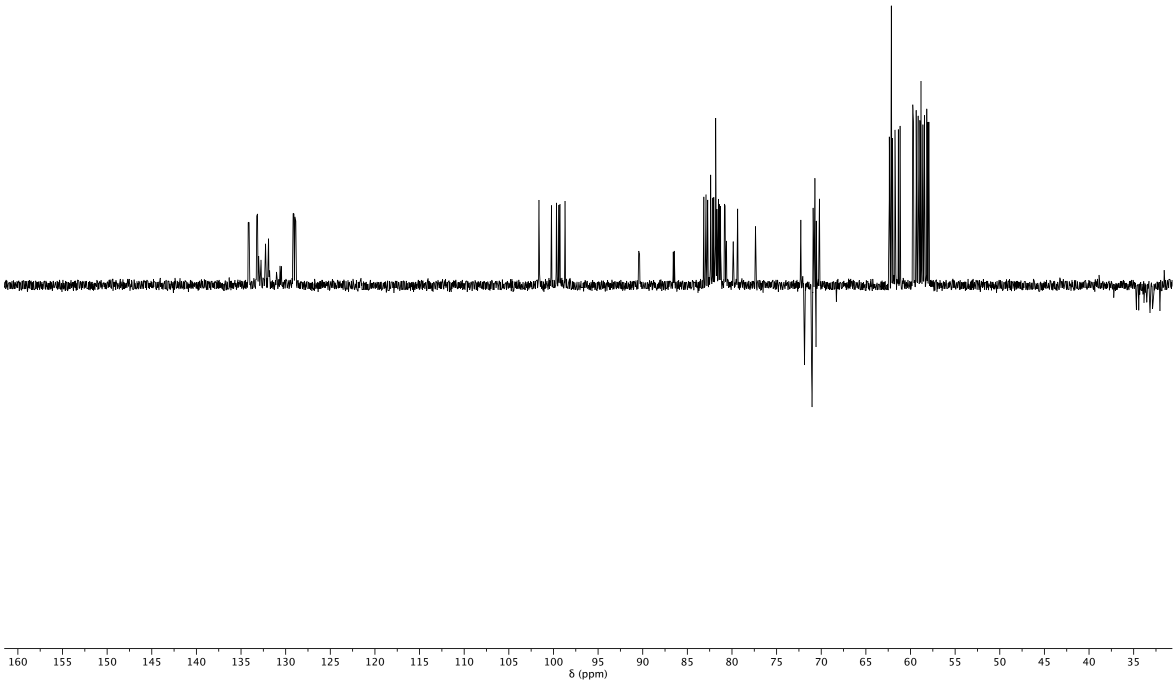

Figure S71. DEPT 135 NMR (126 MHz, C_6D_6) spectrum of [$PdCl_2$(**L²**)].

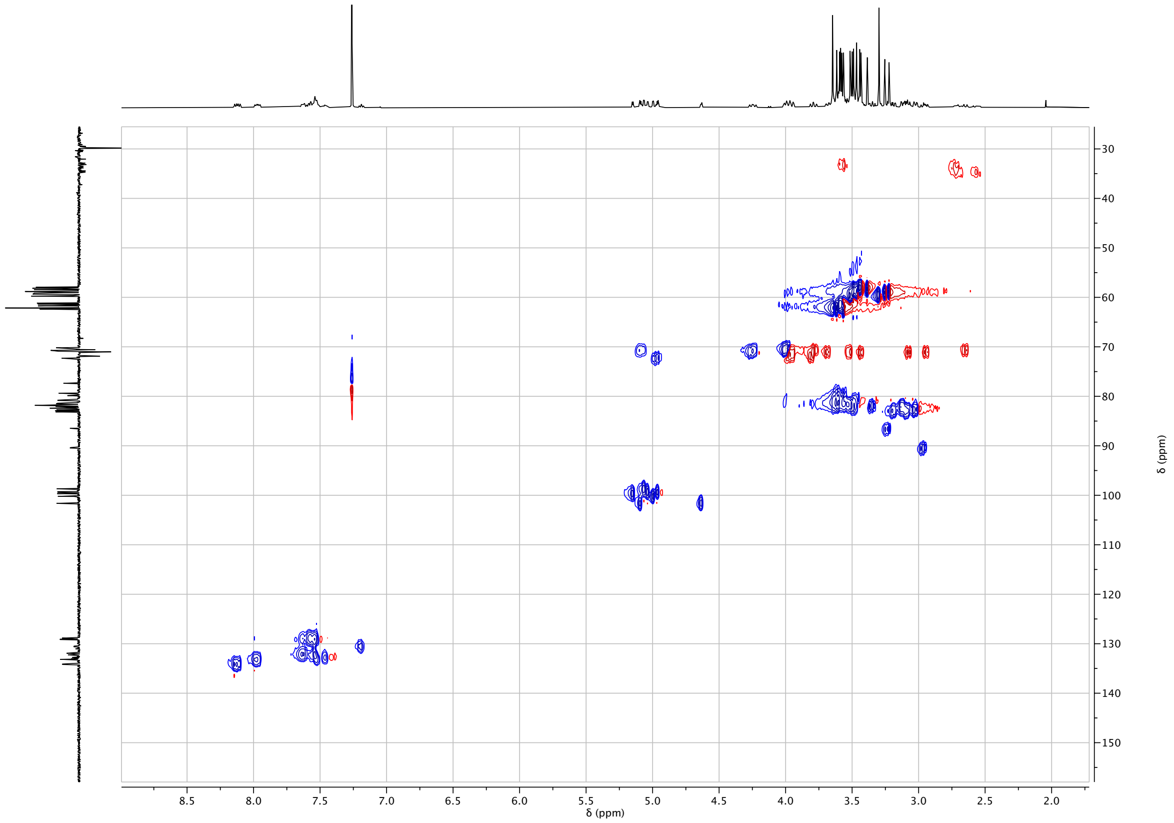

Figure S72. ^{1}H-$^{13}C\{^{1}H\}$ edited HSQC NMR (500 MHz, C_6D_6) spectrum of [$PdCl_2$(**L^2**)].

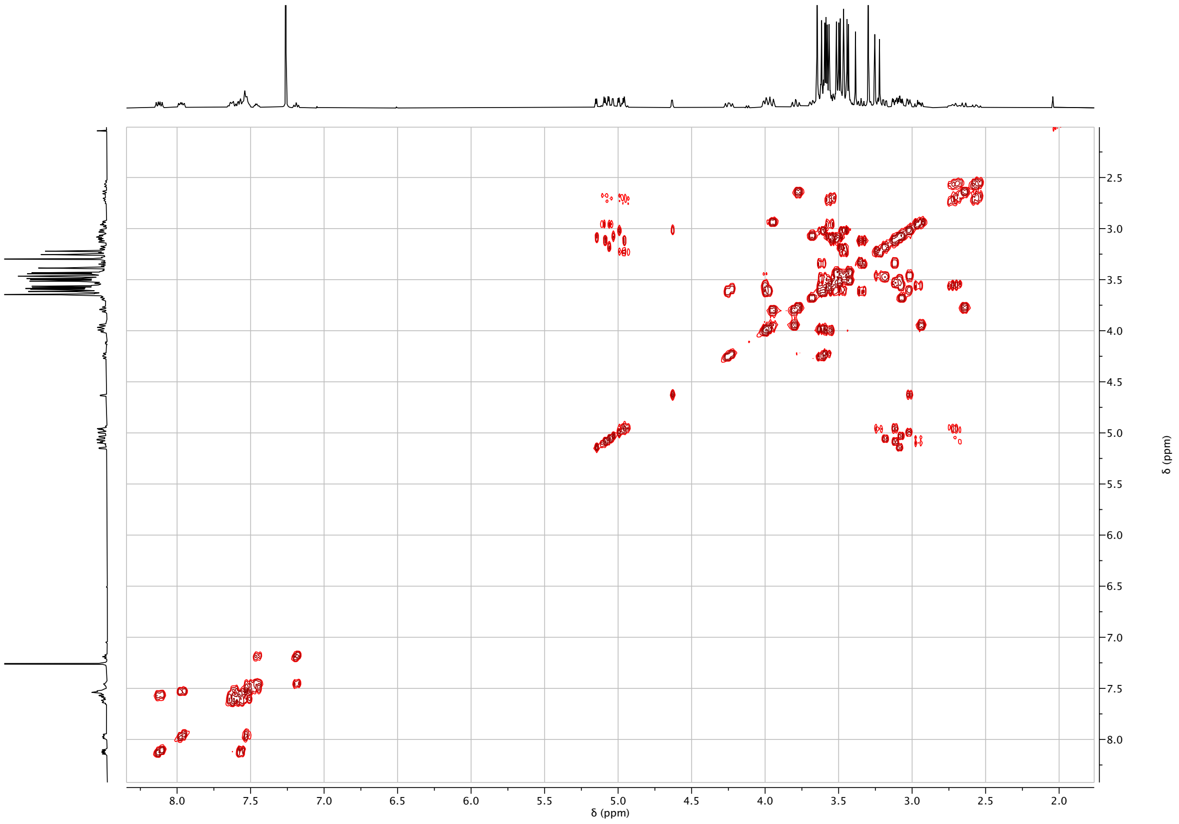

Figure S73. ^{1}H-^{1}H COSY NMR (500 MHz, C_6D_6) spectrum of [$PdCl_2$(**L^2**)].

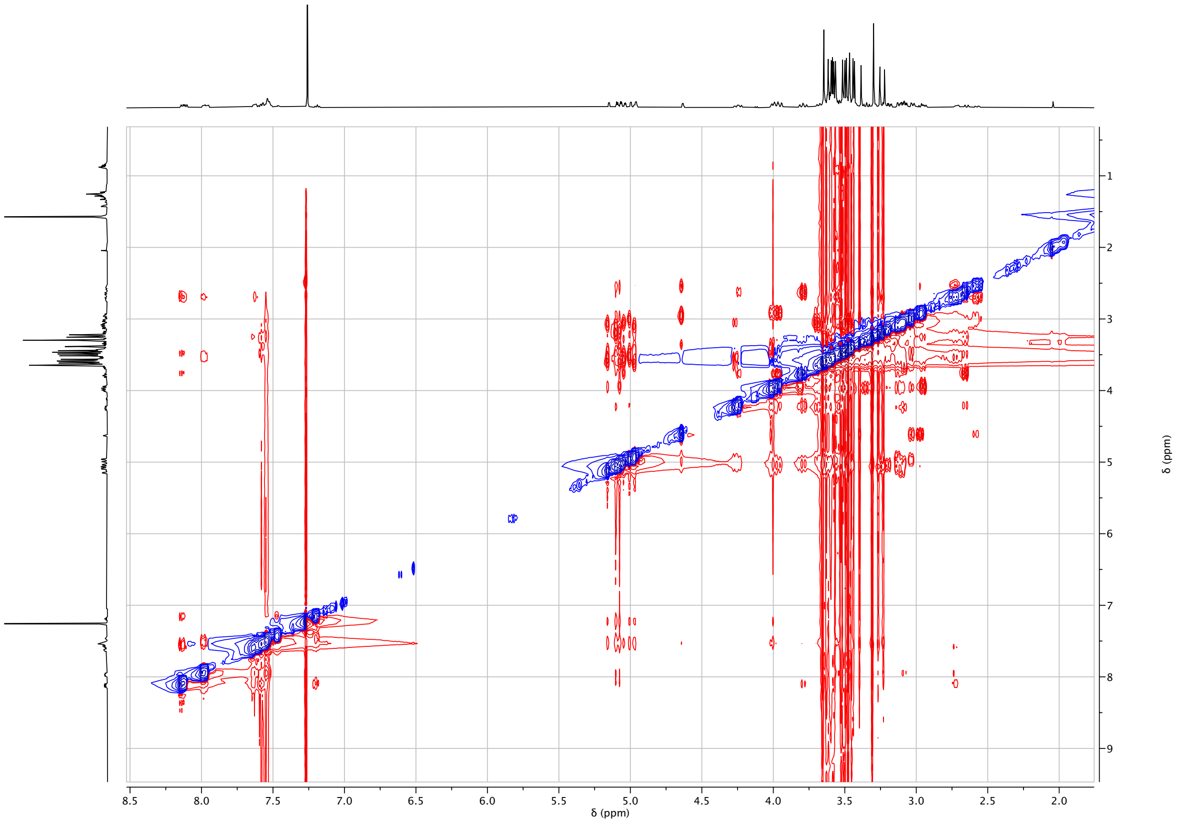

Figure S74. ^{1}H-^{1}H ROESY NMR (500 MHz, C_6D_6) spectrum of [$PdCl_2$(**L²**)].

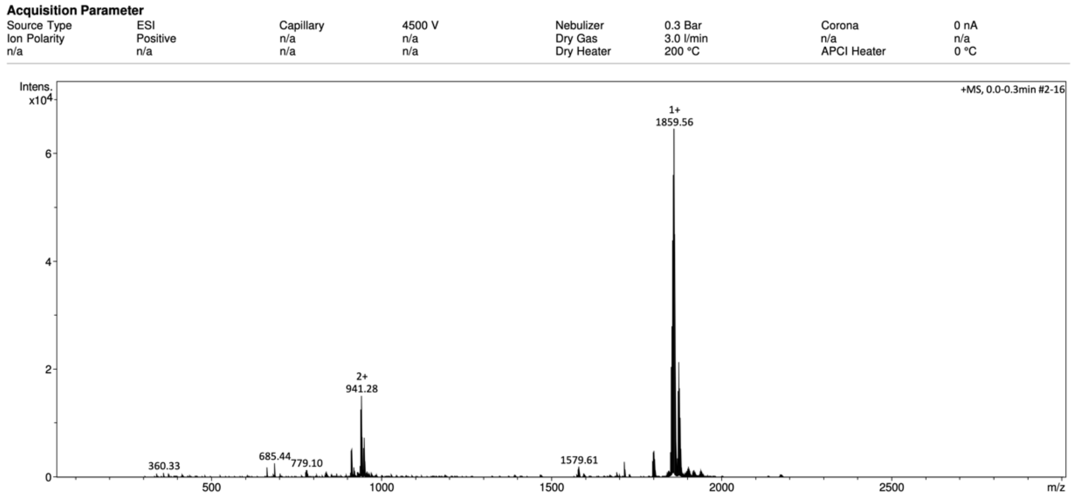

Figure S75. Full ESI-MS spectrum of [$PdCl_2$(**L²**)].

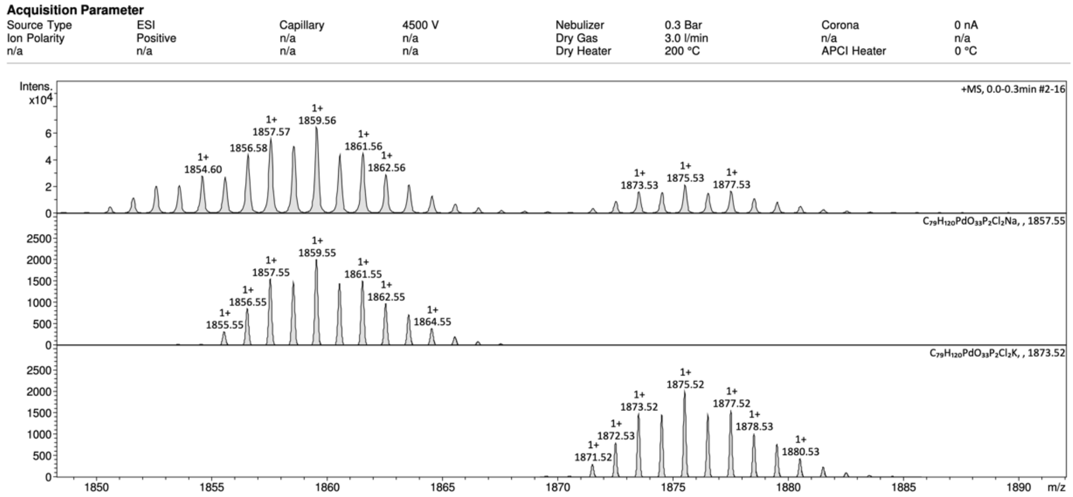

Figure S76. Experimental and simulated partial ESI-MS spectrum of [PdCl$_2$(**L^2**)].

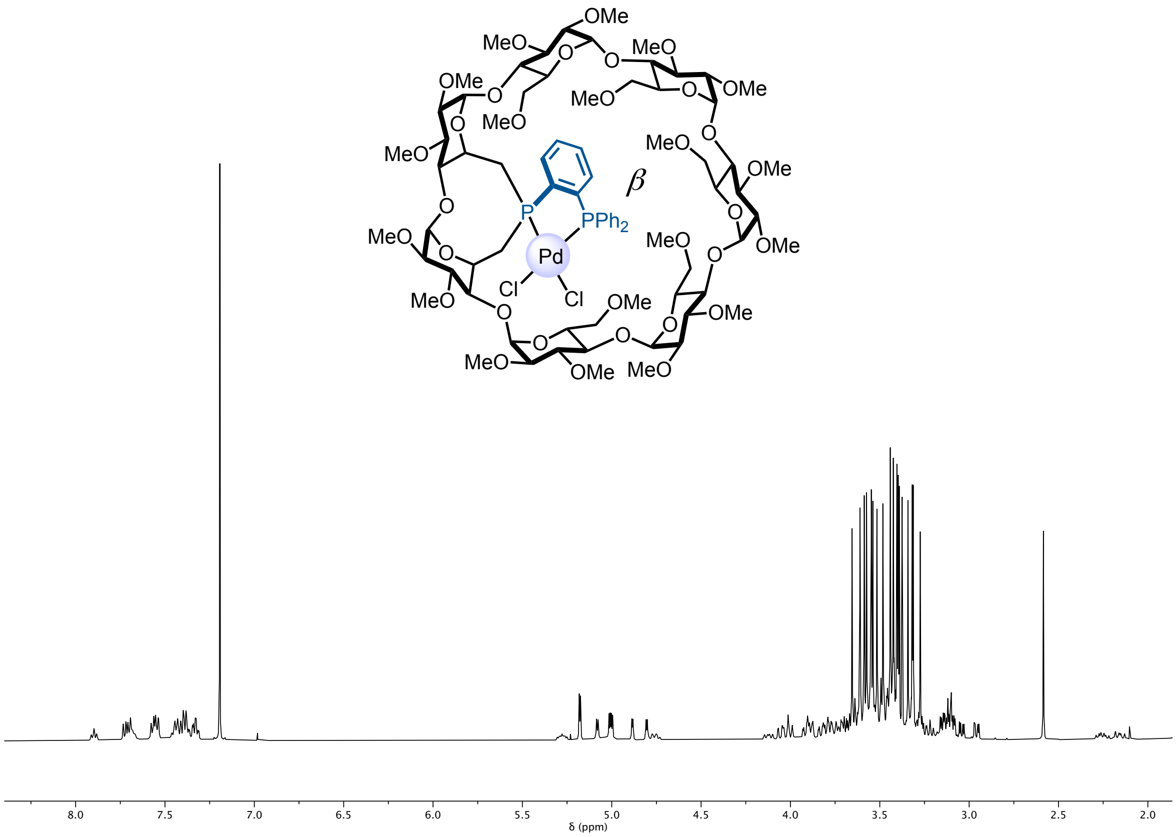

Figure S77. ^{1}H NMR (500 MHz, $CDCl_3$) spectrum of $[PdCl_2(\mathbf{L^4})]$.

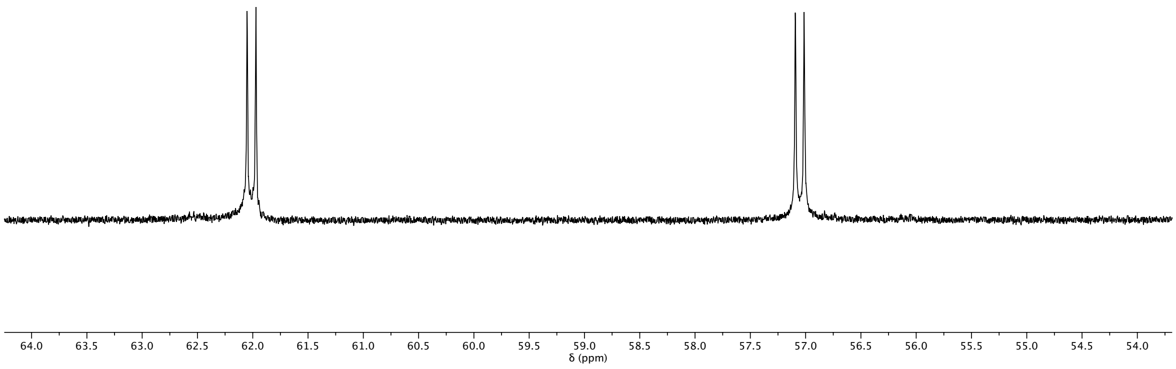

Figure S78. $^{31}P\{^{1}H\}$ NMR (202 MHz, $CDCl_3$) spectrum of $[PdCl_2(\mathbf{L^4})]$.

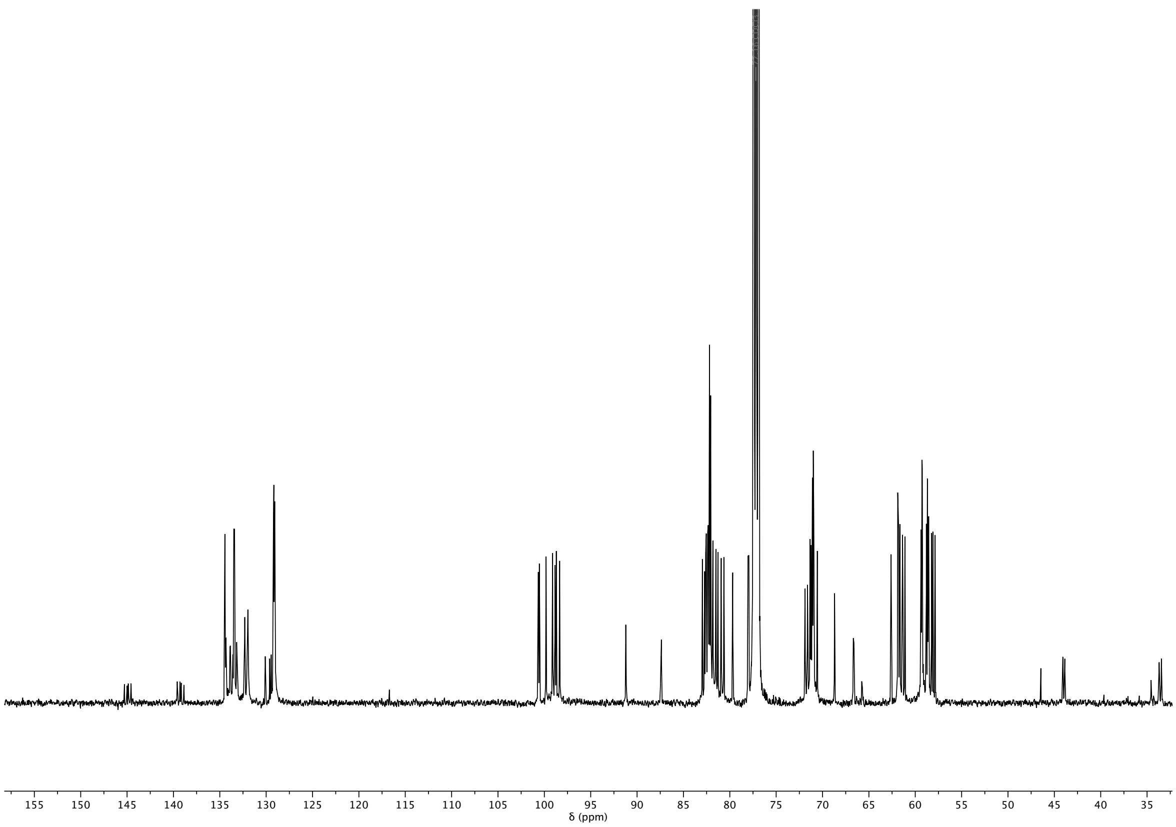

Figure S79. $^{13}C\{^{1}H\}$ NMR (126 MHz, $CDCl_3$) spectrum of [$PdCl_2$(**L**^{4})].

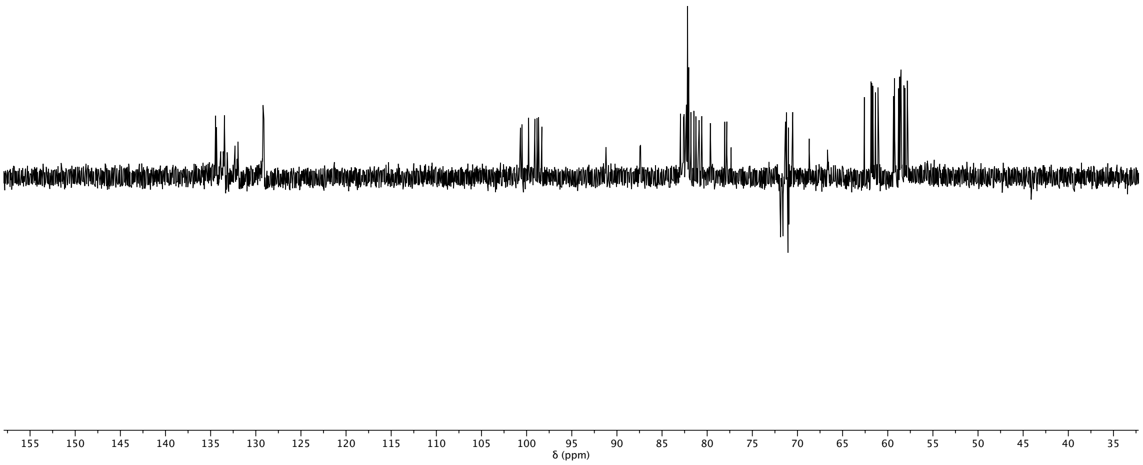

Figure S80. DEPT 135 NMR (126 MHz, $CDCl_3$) spectrum of [$PdCl_2$(**L**^{4})].

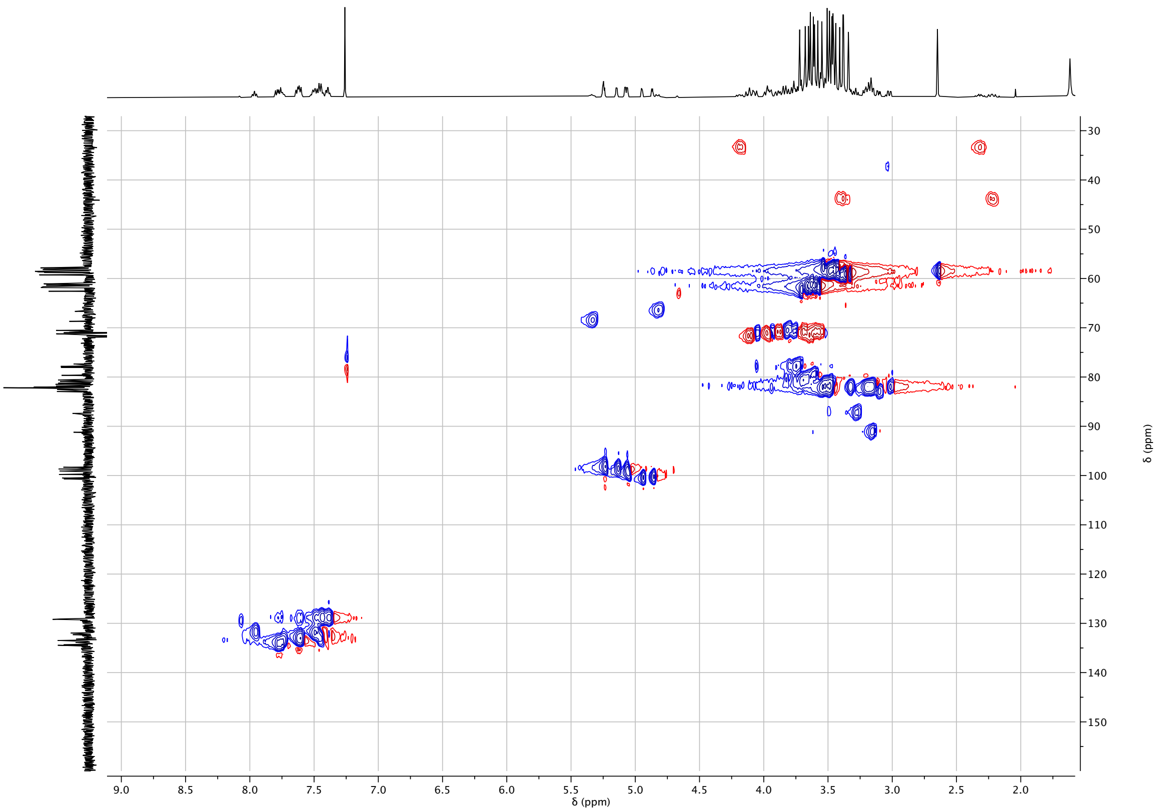

Figure S81. ^{1}H-$^{13}C\{^{1}H\}$ edited HSQC NMR (500 MHz, $CDCl_3$) spectrum of [$PdCl_2$(**L**^{4})].

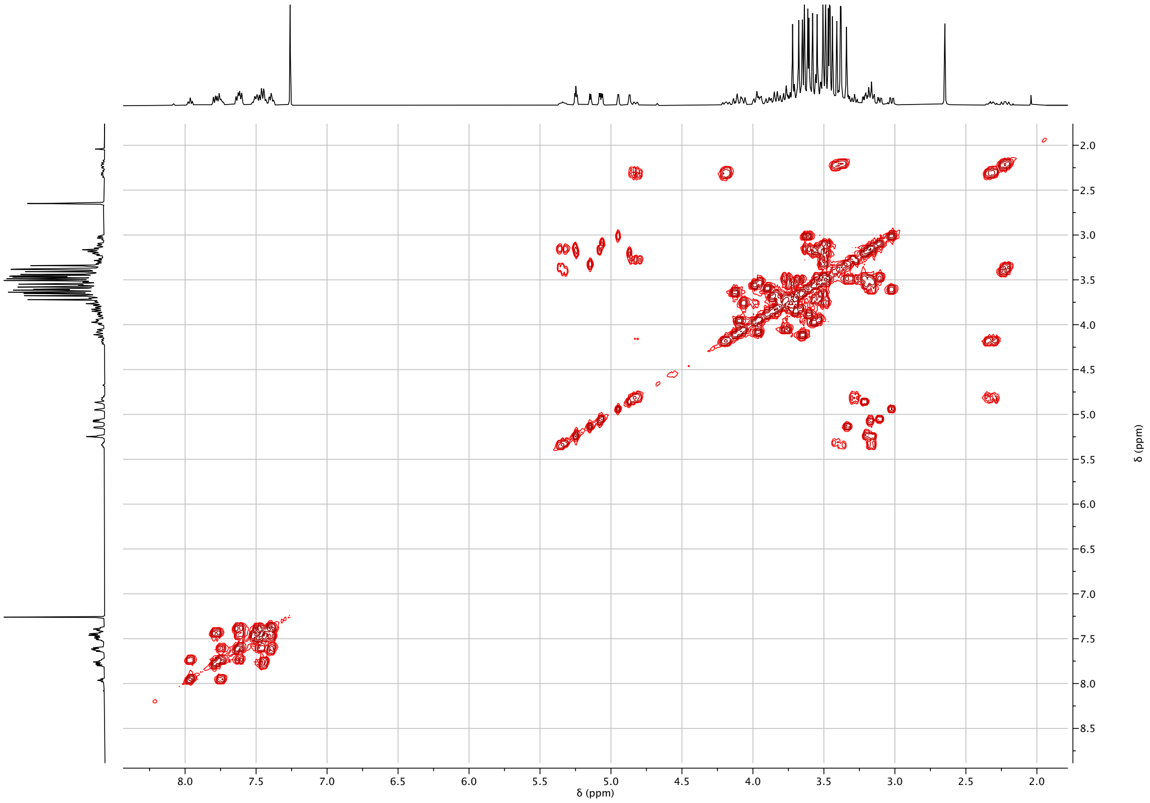

Figure S82. ^{1}H-^{1}H COSY NMR (500 MHz, $CDCl_3$) spectrum of [$PdCl_2$(**L**^{4})].

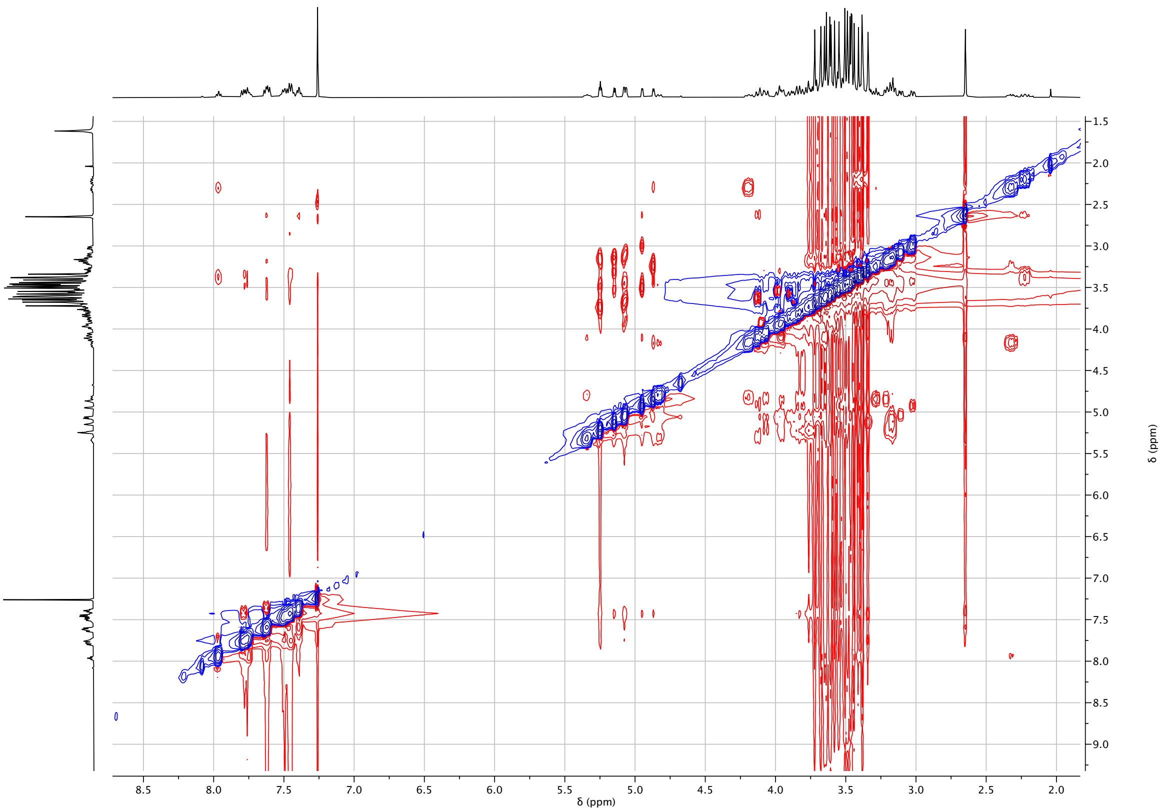

Figure S83. ^{1}H-^{1}H ROESY NMR (500 MHz, $CDCl_3$) spectrum of [$PdCl_2$(**L**^{4})].

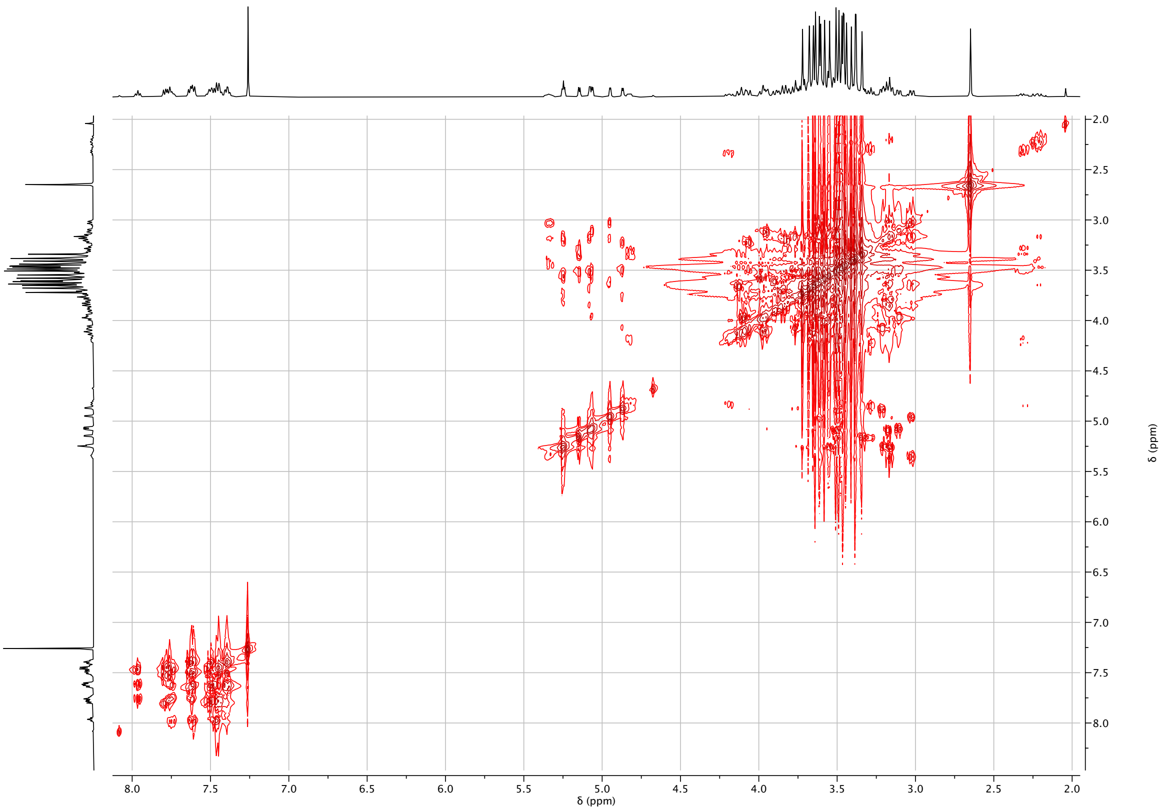

Figure S84. ^{1}H-^{1}H TOCSY NMR (500 MHz, $CDCl_3$) spectrum of [$PdCl_2$(**L**^{4})].

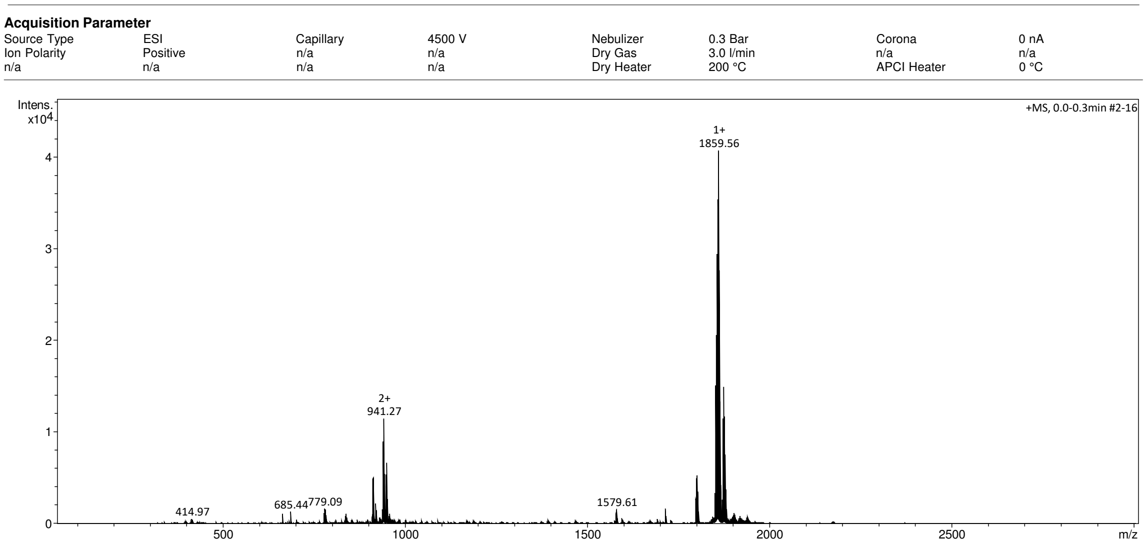

Figure S85. Full ESI-MS spectrum of [PdCl$_2$(**L^4**)].

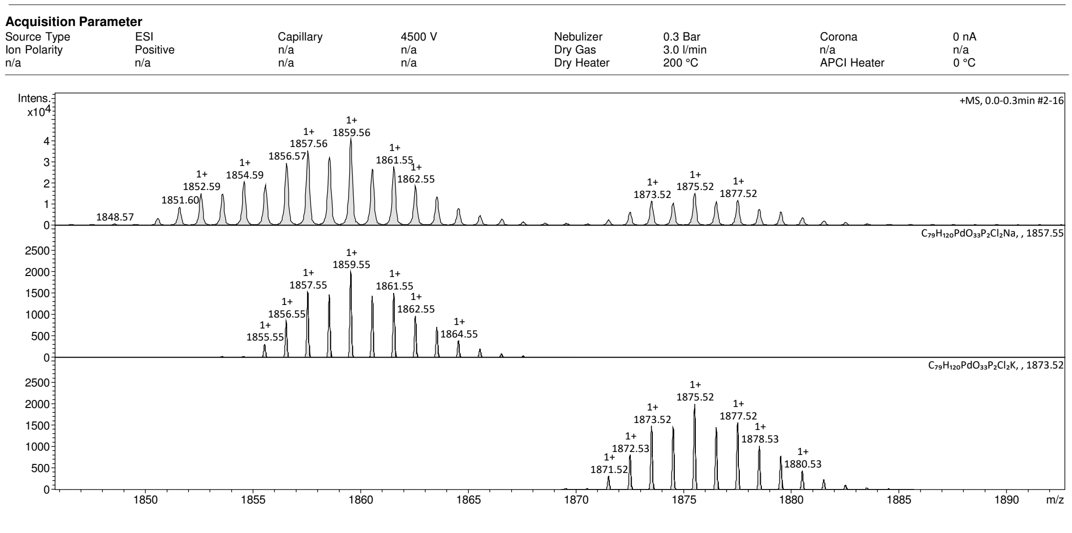

Figure S86. Experimental and simulated partial ESI-MS spectrum of [PdCl$_2$(**L^4**)].

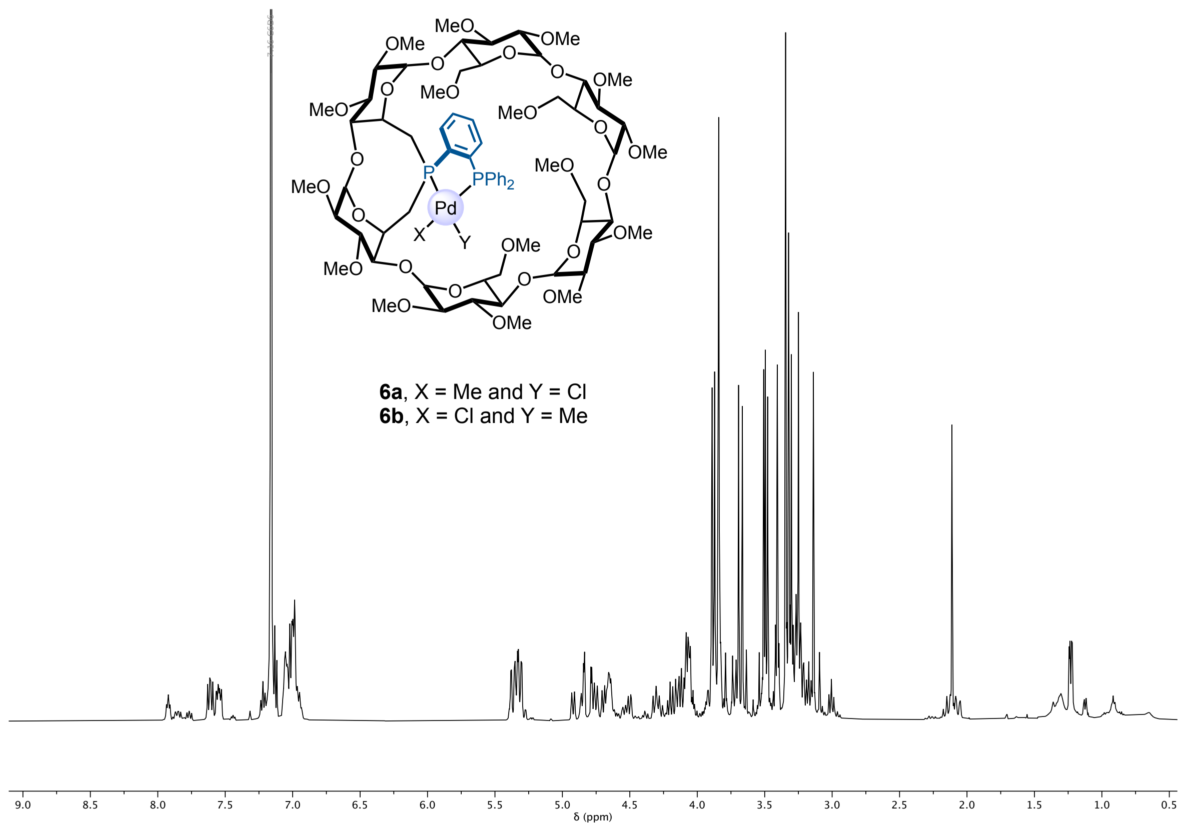

Figure S87. ^{1}H NMR (500 MHz, C_6D_6) spectrum of **6a,b** (25:75 mixture).

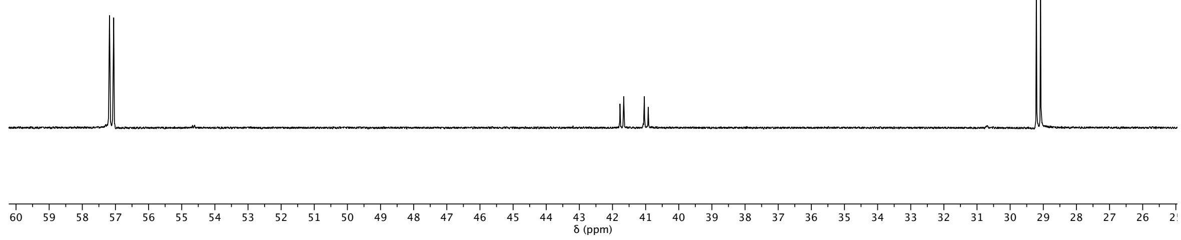

Figure S88. ^{31}P{^{1}H} NMR (202 MHz, C_6D_6) spectrum of **6a,b** (25:75 mixture).

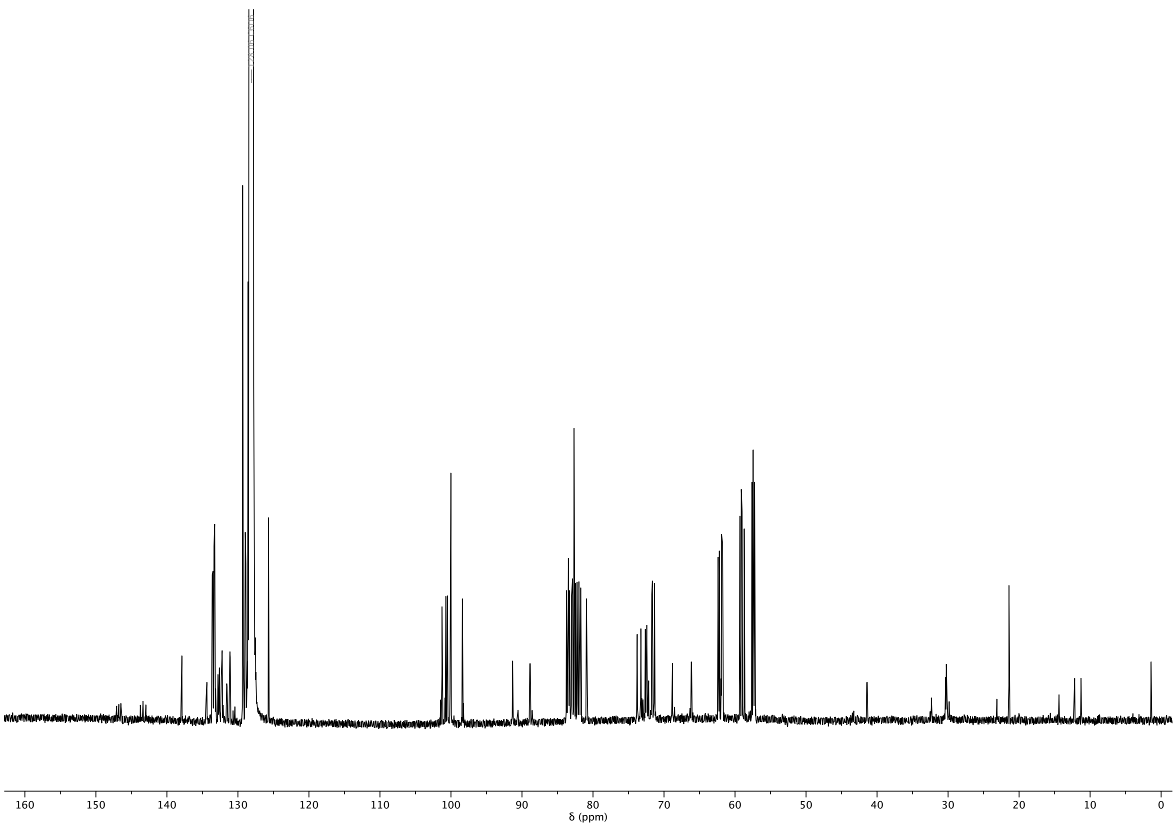

Figure S89. $^{13}C\{^1H\}$ NMR (126 MHz, C_6D_6) spectrum of **6a,b** (25/75 mixture).

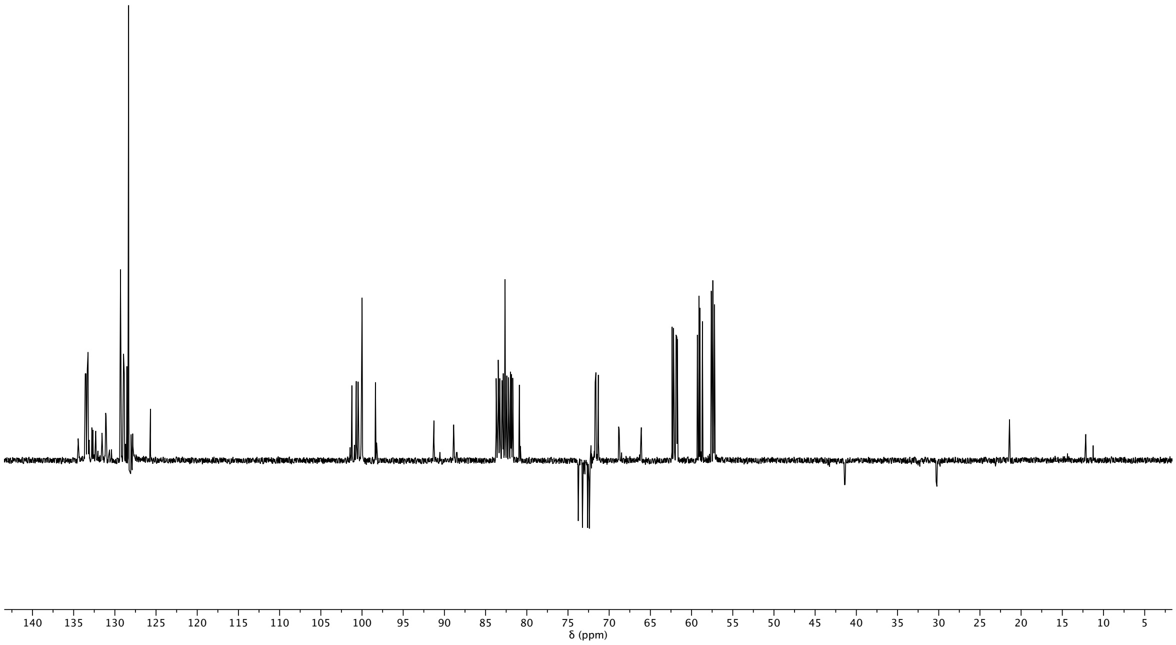

Figure S90. DEPT 135 NMR (126 MHz, C_6D_6) spectrum of **6a,b** (25:75 mixture).

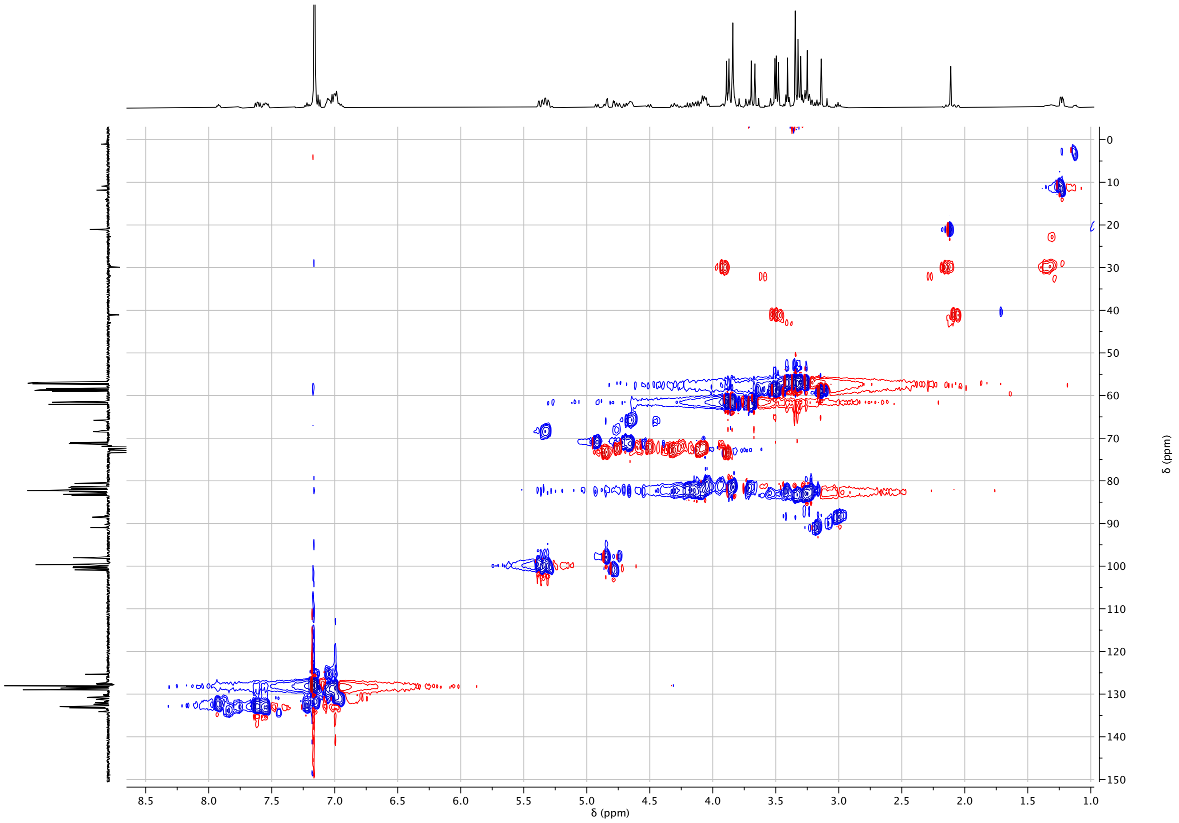

Figure S91. ^{1}H-$^{13}C\{^{1}H\}$ edited HSQC NMR (500 MHz, C_6D_6) spectrum of **6a,b** (25:75 mixture).

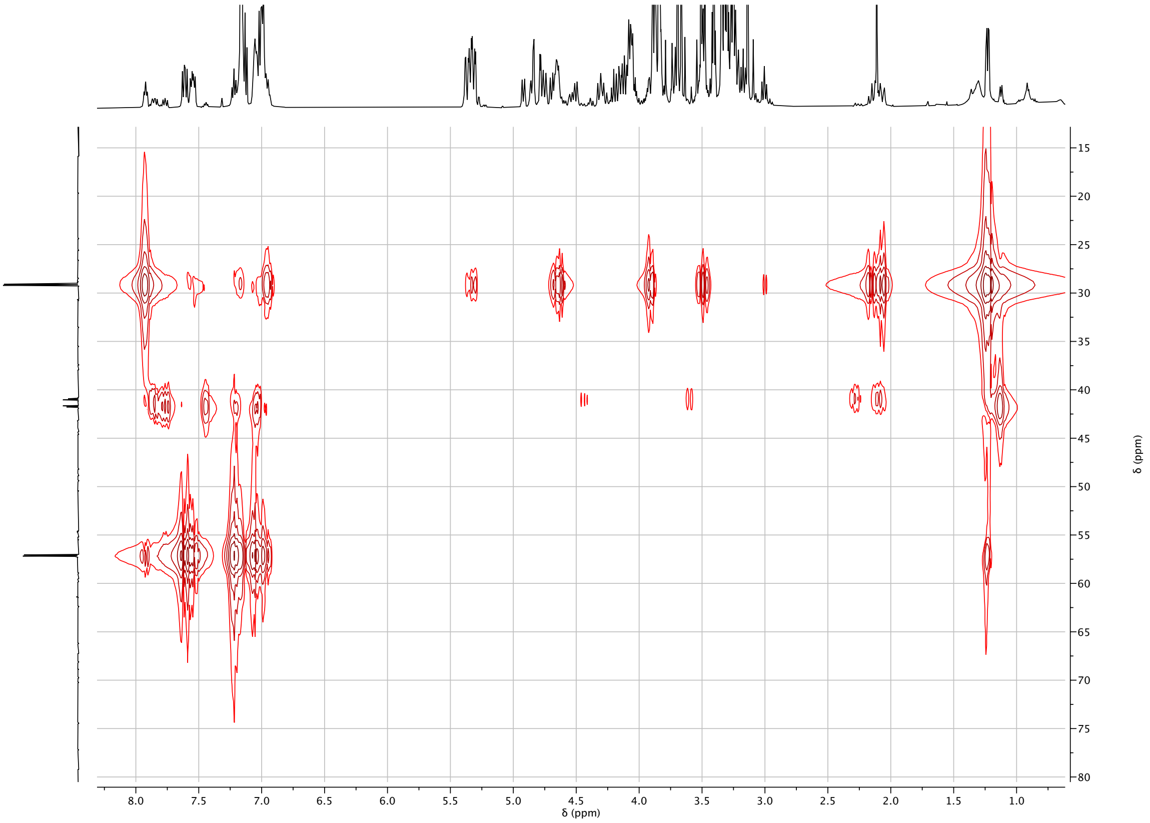

Figure S92. ^{1}H-^{31}P HMQC NMR (500 MHz, C_6D_6) spectrum of **6a,b** (25:75 mixture).

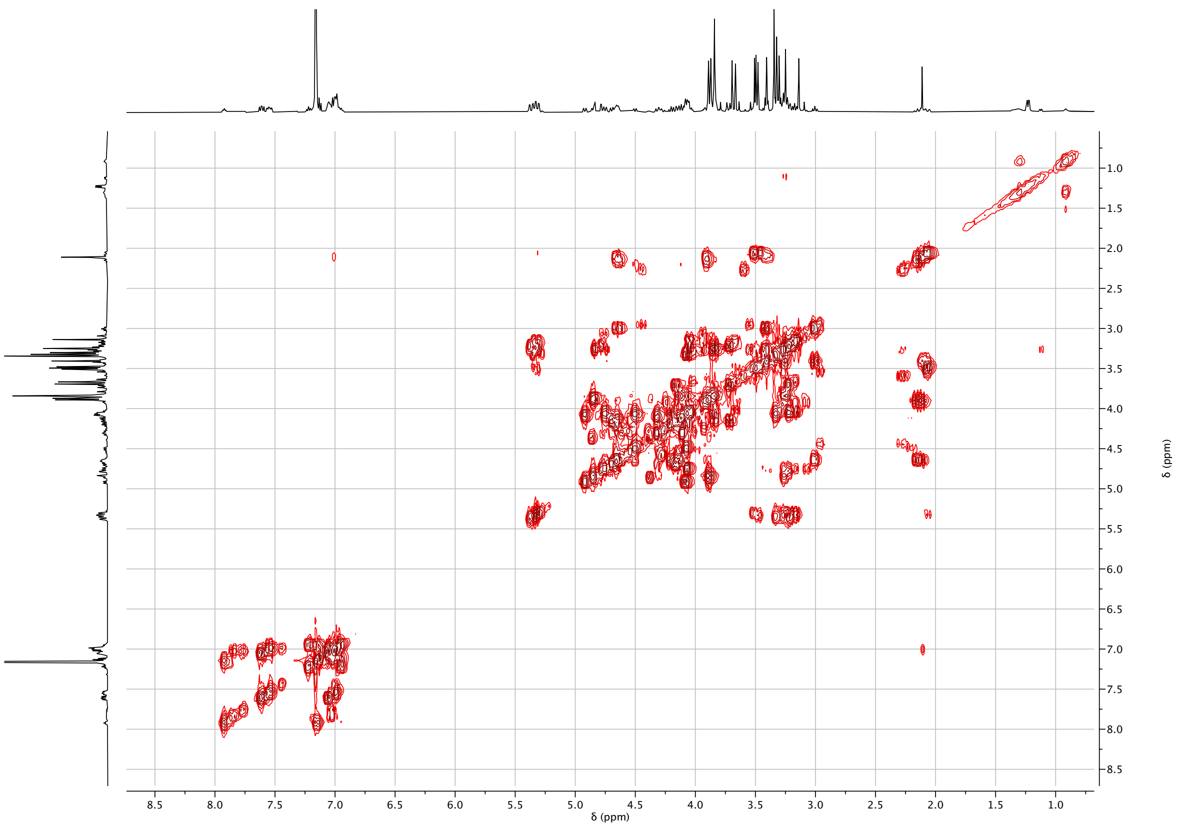

Figure S93. ^1H-^1H COSY NMR (500 MHz, C_6D_6) spectrum of **6a,b** (25:75 mixture).

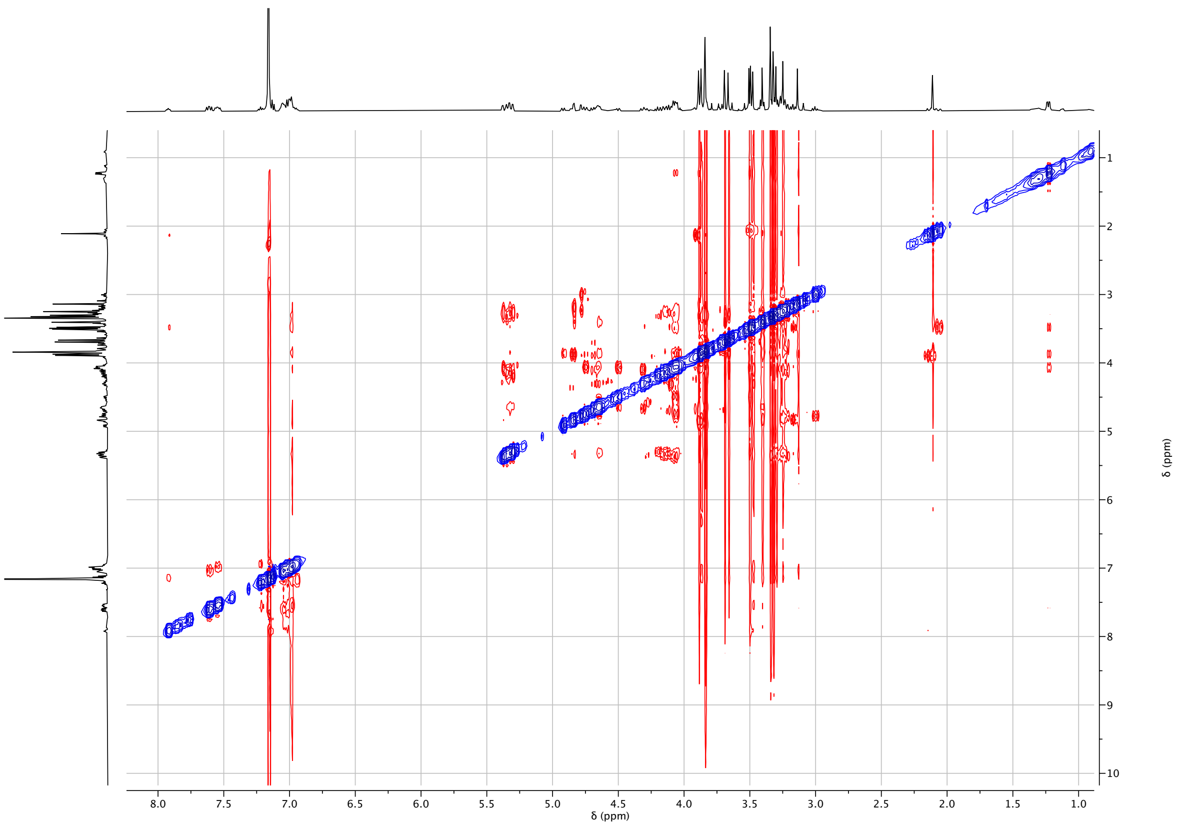

Figure S94. ^1H-^1H ROESY NMR (500 MHz, C_6D_6) spectrum of **6a,b** (25:75 mixture).

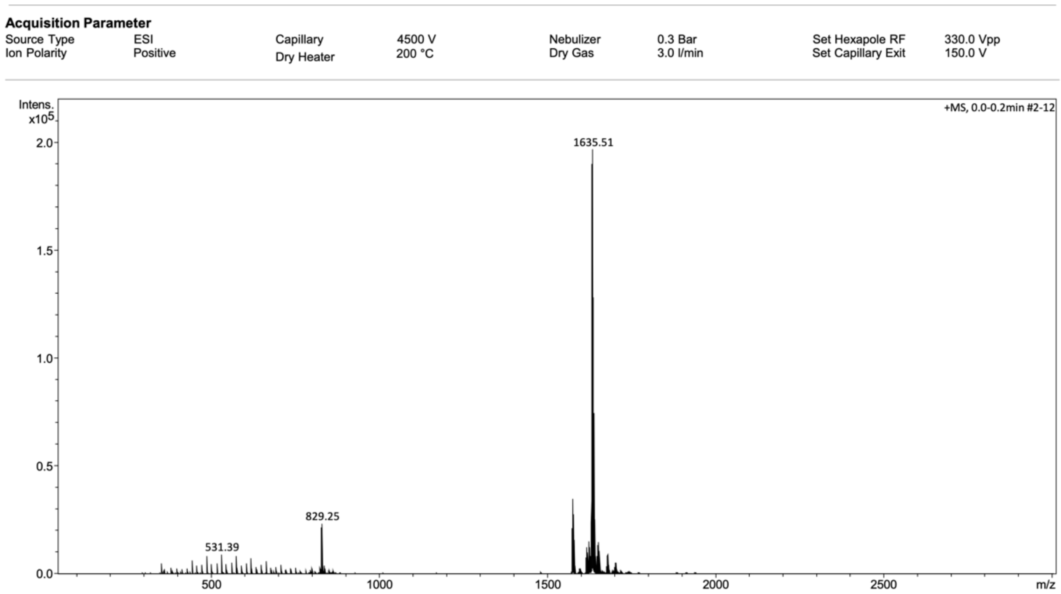

Figure S95. Full ESI-MS spectrum of **6a,b** (25:75 mixture).

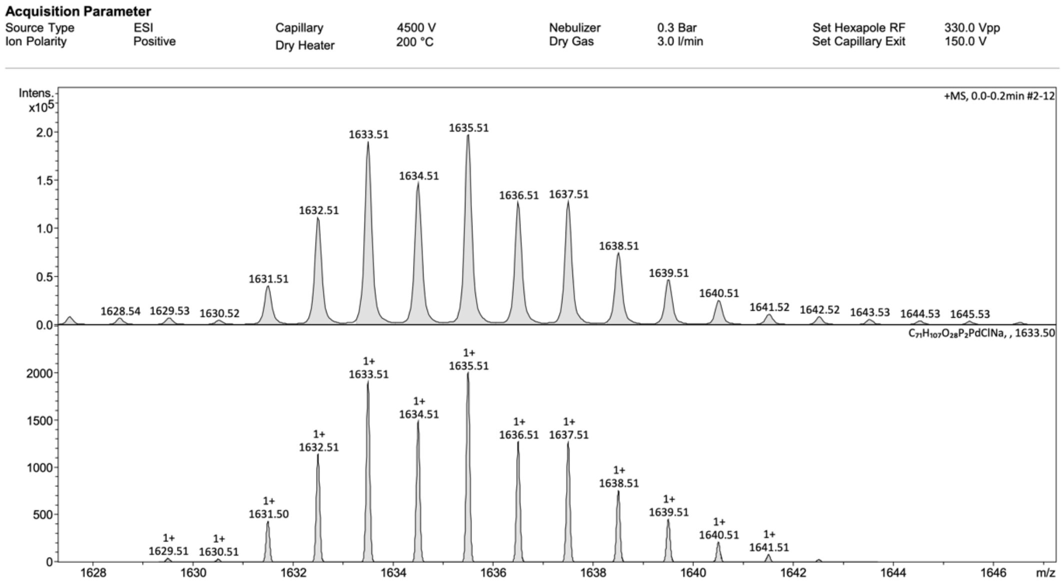

Figure S96. Experimental and simulated partial ESI-MS spectrum of **6a,b** (25:75 mixture).

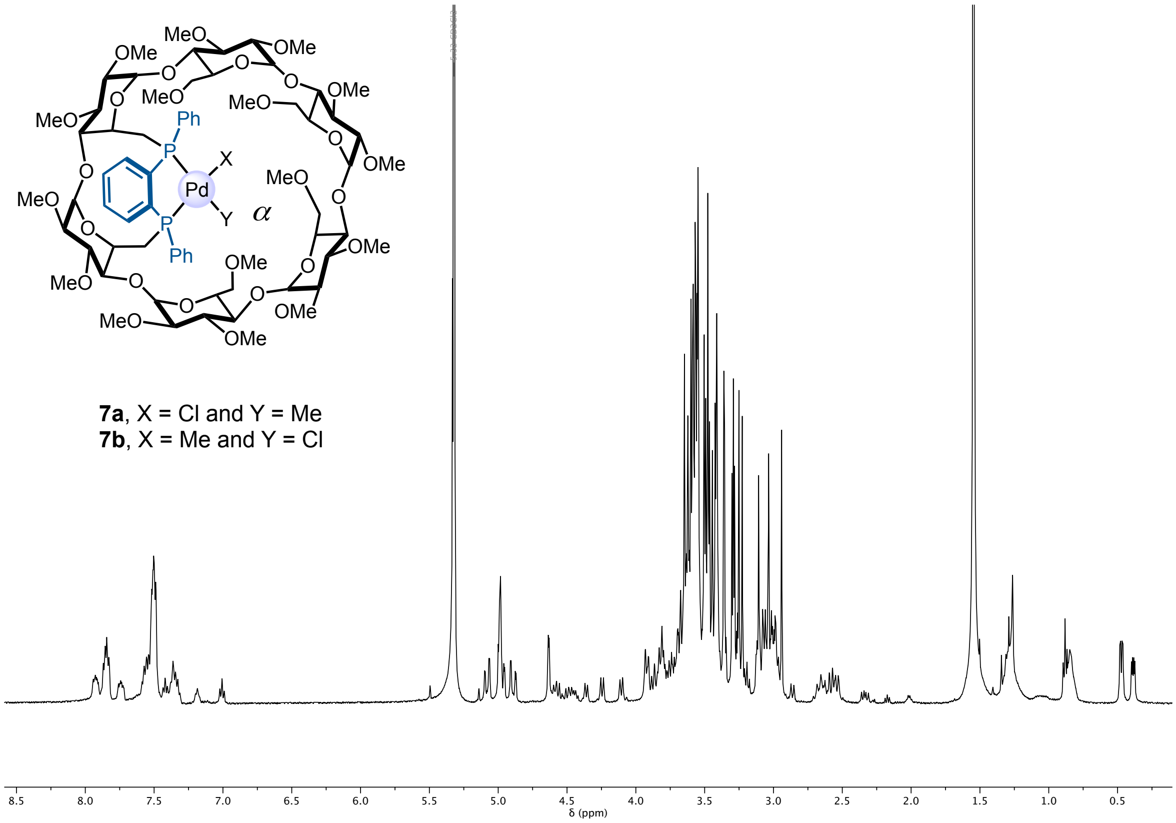

Figure S97. ^{1}H NMR (500 MHz, CD_2Cl_2) spectrum of **7a,b** (60:40 mixture).

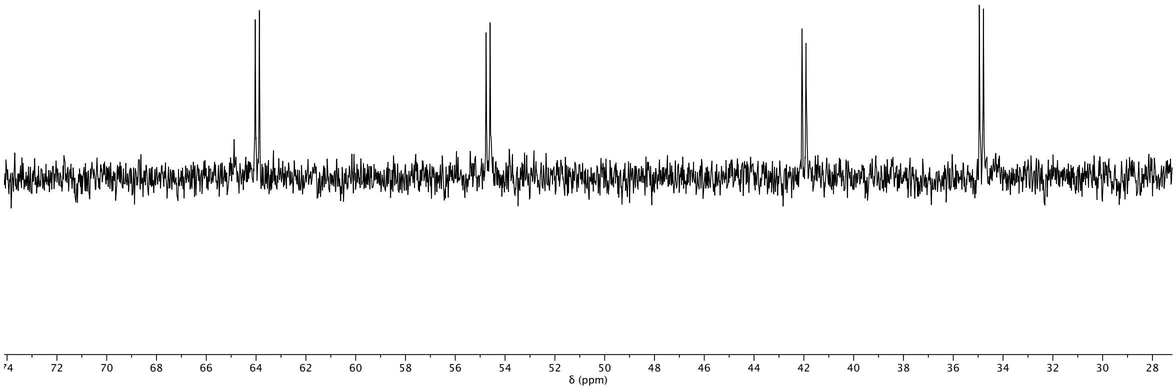

Figure S98. ^{31}P{^{1}H} NMR (162 MHz, CD_2Cl_2) spectrum of **7a,b** (60:40 mixture).

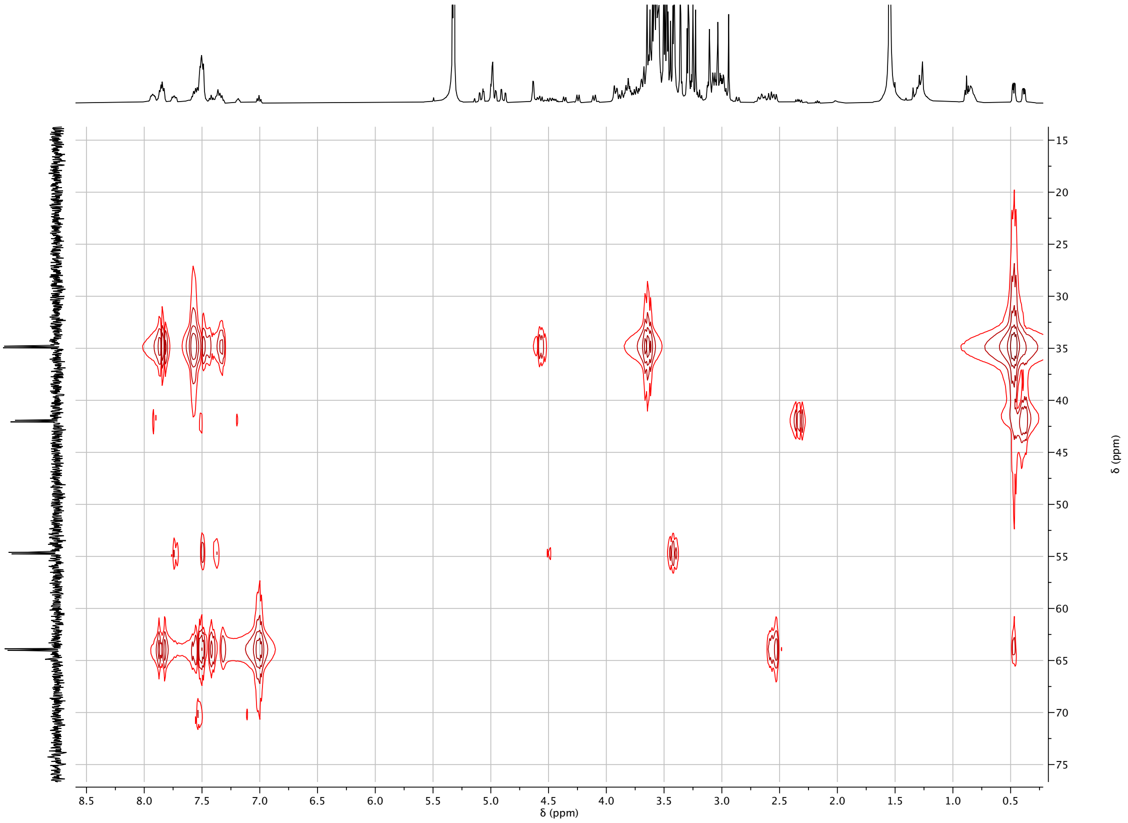

Figure S99. ^{1}H-^{31}P HMQC NMR (500 MHz, C_6D_6) spectrum of **7a,b** (60:40 mixture).

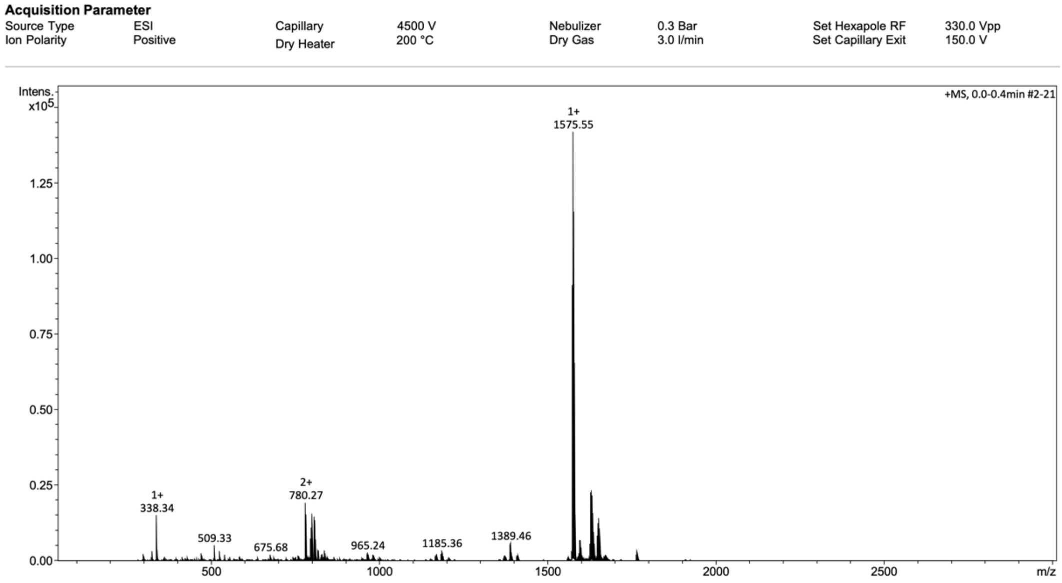

Figure S100. Full ESI-MS spectrum of **7a,b** (60/40 mixture).

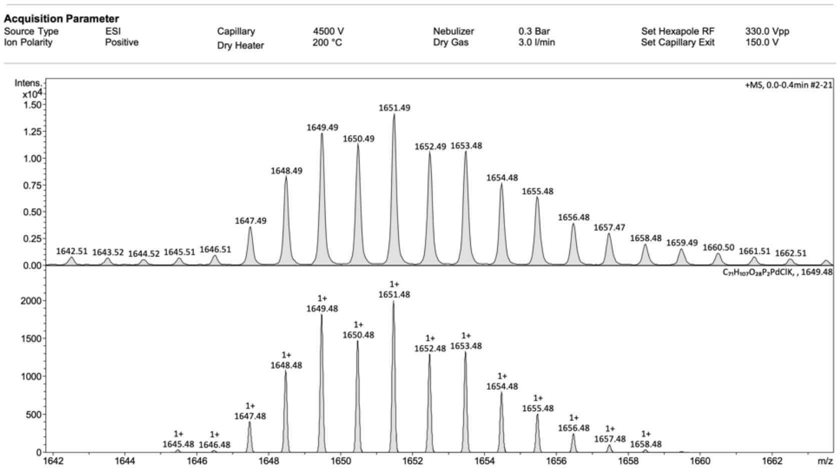

Figure S101. Experimental and simulated partial ESI-MS spectrum of **7a,b** (60:40 mixture).

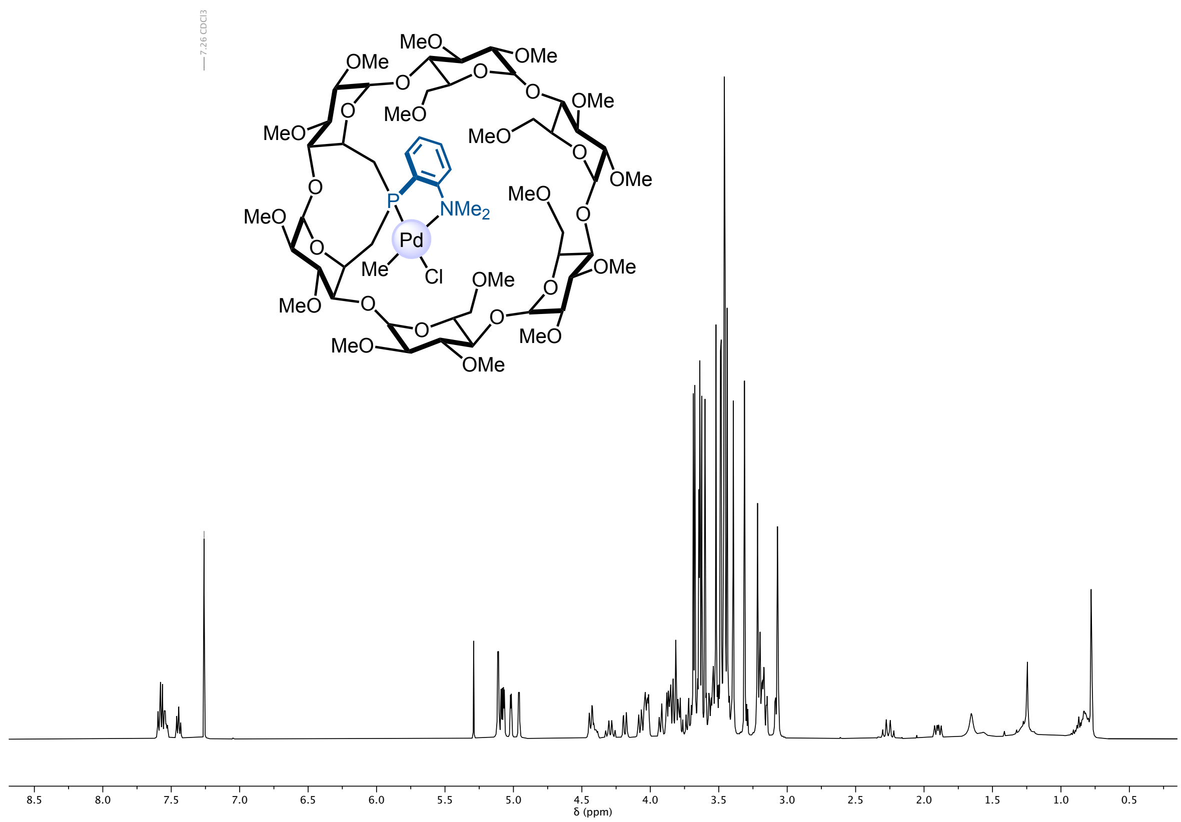

Figure S102. ^{1}H NMR (500 MHz, $CDCl_3$) spectrum of [PdCl(Me)(**L⁶**)].

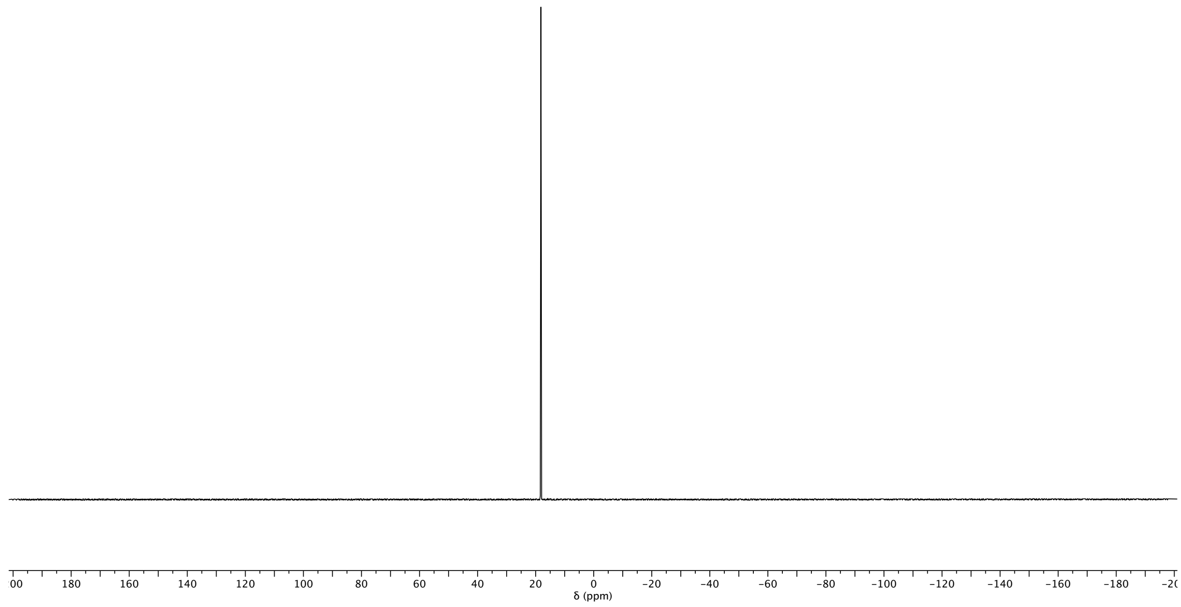

Figure S103. $^{31}P\{^{1}H\}$ NMR (162 MHz, $CDCl_3$) spectrum of [PdCl(Me)(**L⁶**)].

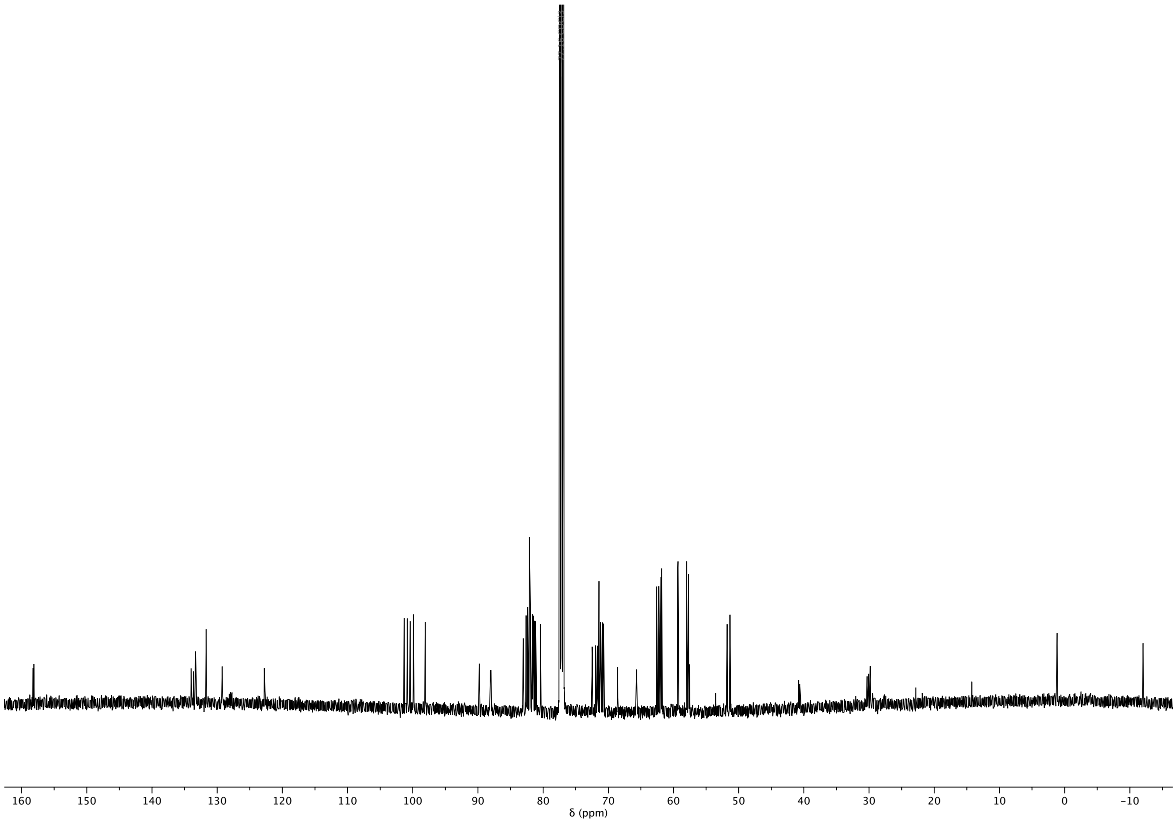

Figure S104. $^{13}C\{^{1}H\}$ NMR (126 MHz, $CDCl_3$) spectrum of [PdCl(Me)(**L⁶**)].

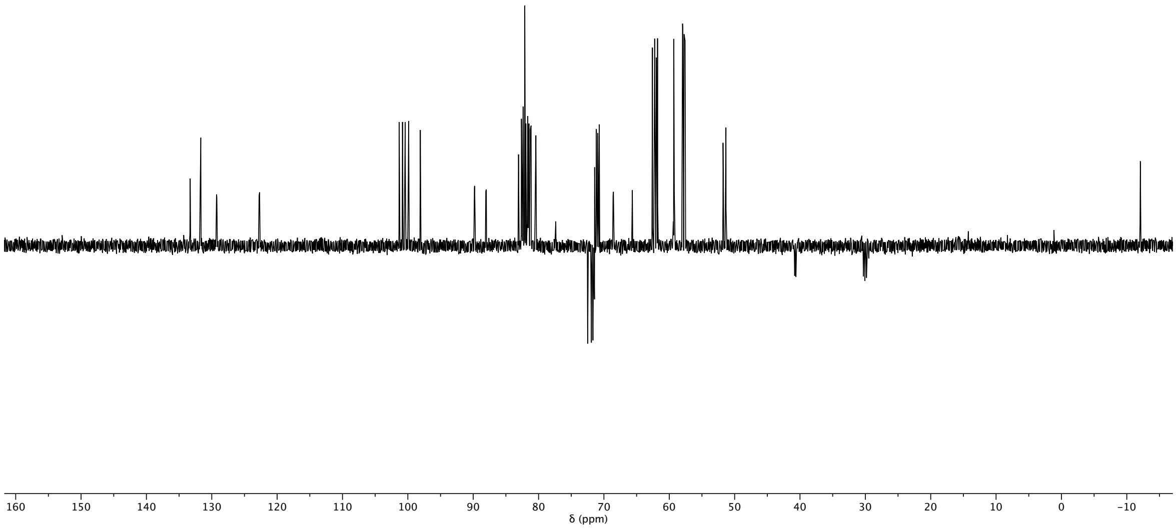

Figure S105. DEPT 135 NMR (126 MHz, $CDCl_3$) spectrum of [PdCl(Me)(**L⁶**)].

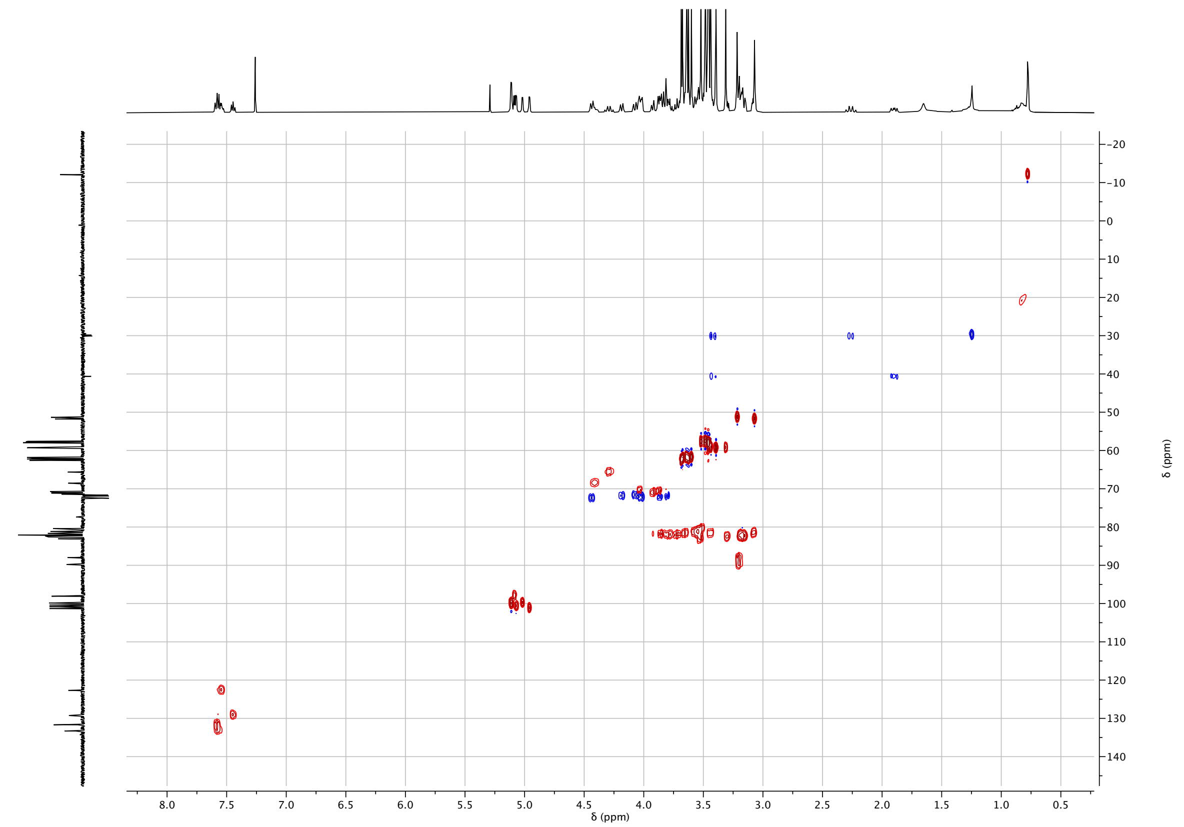

Figure S106. ^{1}H-$^{13}C\{^{1}H\}$ edited HSQC NMR (500 MHz, $CDCl_3$) spectrum of [PdCl(Me)(**L^{6}**)].

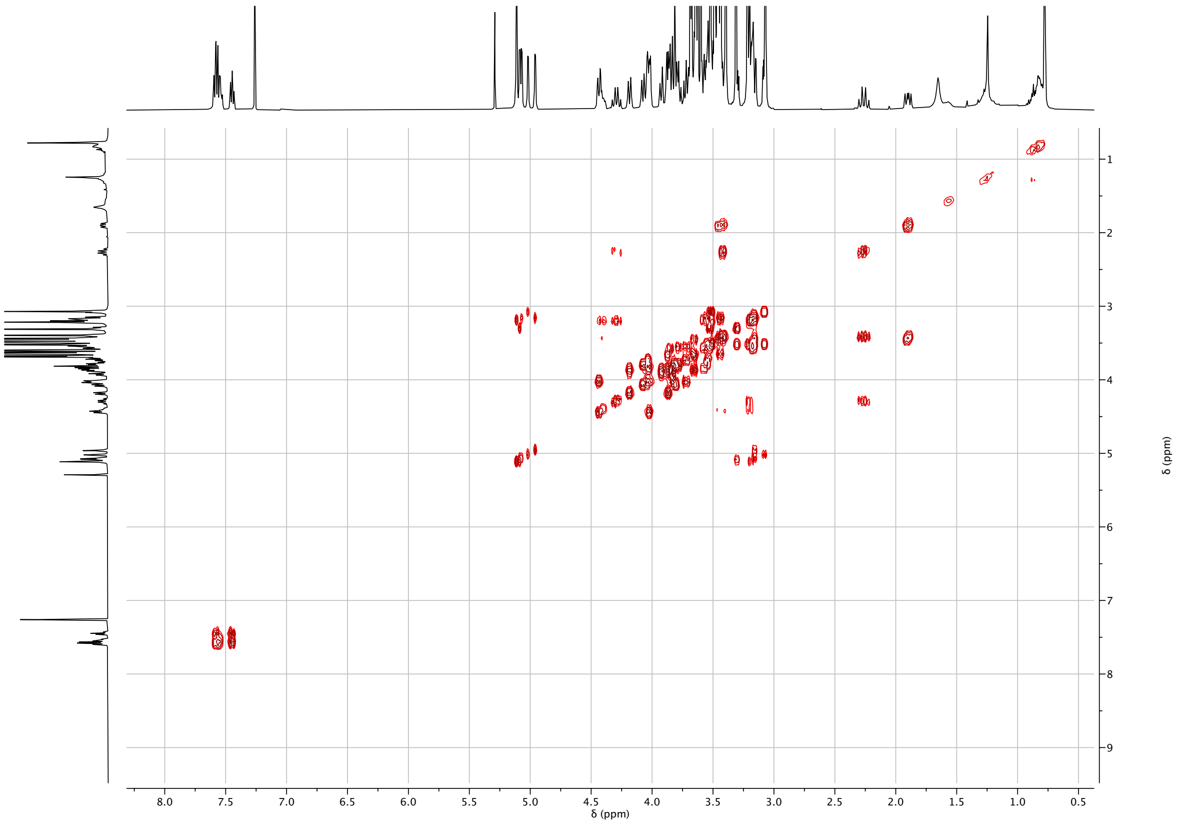

Figure S107. ^{1}H-^{1}H COSY NMR (500 MHz, $CDCl_3$) spectrum of [PdCl(Me)(**L^{6}**)].

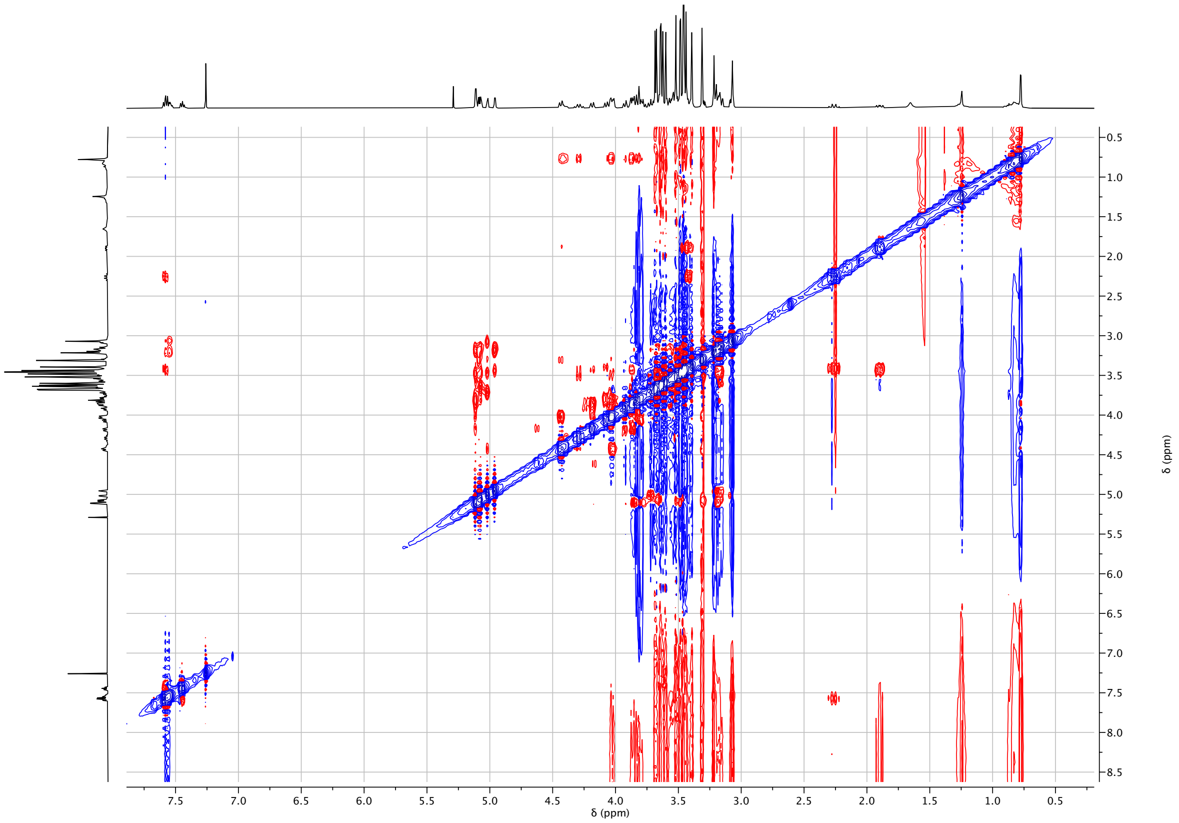

Figure S108. ^{1}H-^{1}H ROESY NMR (500 MHz, $CDCl_3$) spectrum of [PdCl(Me)(**L**^{6})].

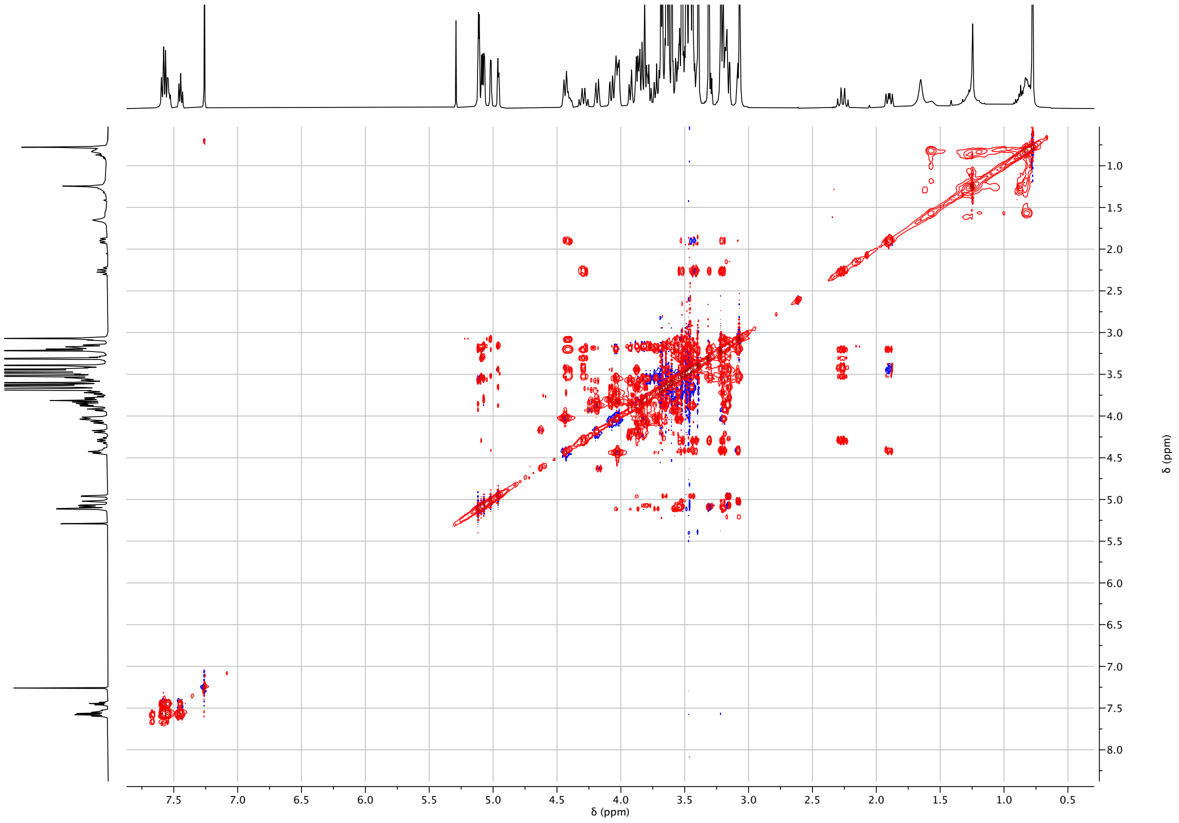

Figure S109. ^{1}H-^{1}H TOCSY NMR (500 MHz, $CDCl_3$) spectrum of [PdCl(Me)(**L**^{6})].

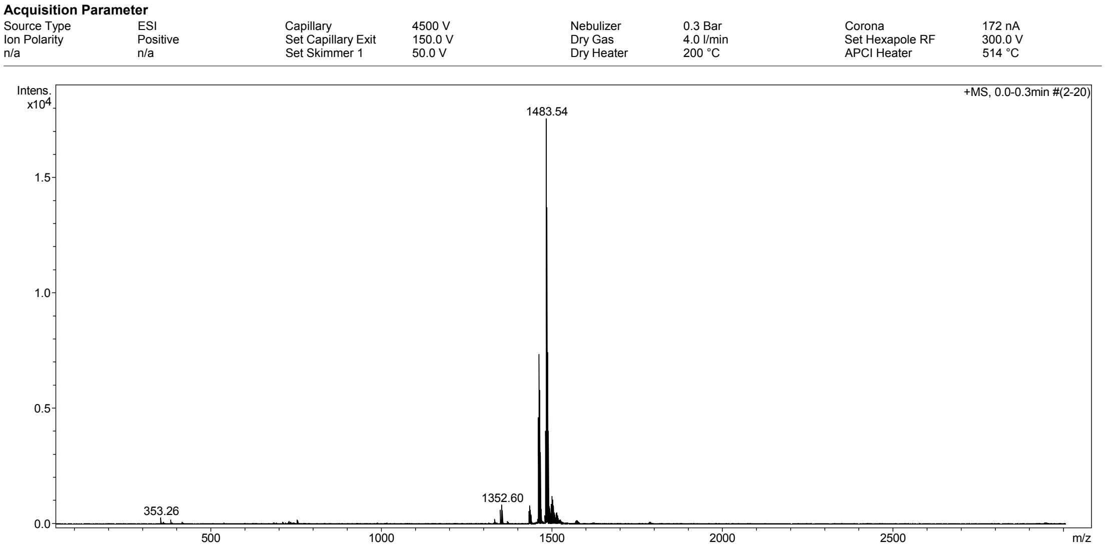

Figure S110. Full ESI-MS spectrum of [PdCl(Me)(**L**^{6})].

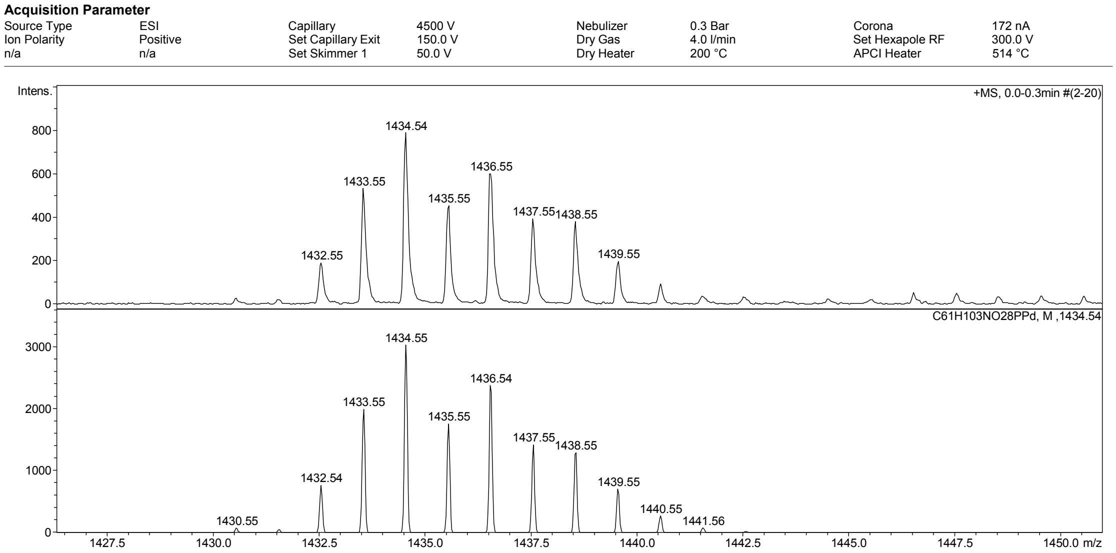

Figure S111. Experimental and simulated partial ESI-MS spectrum of [PdCl(Me)(**L**^{6})].

5. Crystal structure analyses

X-Ray crystal structure of complex [$NiBr_2$(**L^1**)]

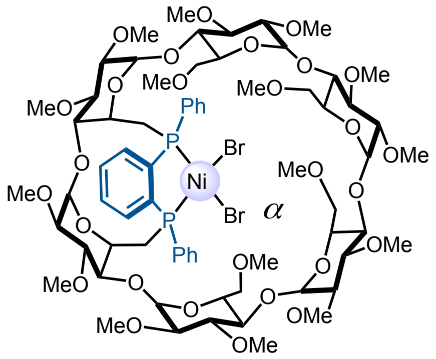

Crystal suitable for X-ray crystal-structure analysis of complex [$NiBr_2$(**L^1**)] was obtained by slow diffusion of pentane into a benzene solution of [$NiBr_2$(**L^1**)]. Data were collected at 120.15 K on a Bruker PHOTON-III CPAD diffractometer (Mo-Kα radiation, λ = 0.71073 Å). The structure was solved by direct methods (SHELXS-2013) and refined against F^2 using the SHELXL-2014 software.[6] The non-hydrogen atoms were refined anisotropically, using weighted full-matrix least-squares on F^2. The H-atoms were included in calculated positions and treated as riding atoms using SHELXL default parameters. Crystallographic data: formula: $C_{71}H_{107}O_{28}P_2ClPd$•7($C_6H_6$) ($M$ = 2220.839 g.mol^{-1}); orange crystals, 0.25 × 0.20 × 0.15 mm; crystal system: orthorhombic, space group $P2_12_12_1$; α = 14.2347(8) Å; β= 27.9371(17) Å; γ= 28.0821(15) Å; V = 11167.6(11) Å^3; Z = 4; F(000) = 4680; a total of 189194 reflections collected; 4.08° < θ < 55.86 °, 26682 independent reflections with 22632 having $I > 2\sigma(I)$; 1310 parameters; Final results : $R_1(F^2)$ = 0.0321; w$R_2(F^2)$ = 0.0634, Goof = 1.033. The asymmetric unit contains one molecule of metal complex together with seven benzene molecules. One of them is included in the CD cavity. Another benzene molecule is disordered over two positions with a ratio of 0.6/0.4. Full data collection parameters and structural data are available as CIF file (Cambridge Crystallographic Data Center deposition number CCDC 2352345).

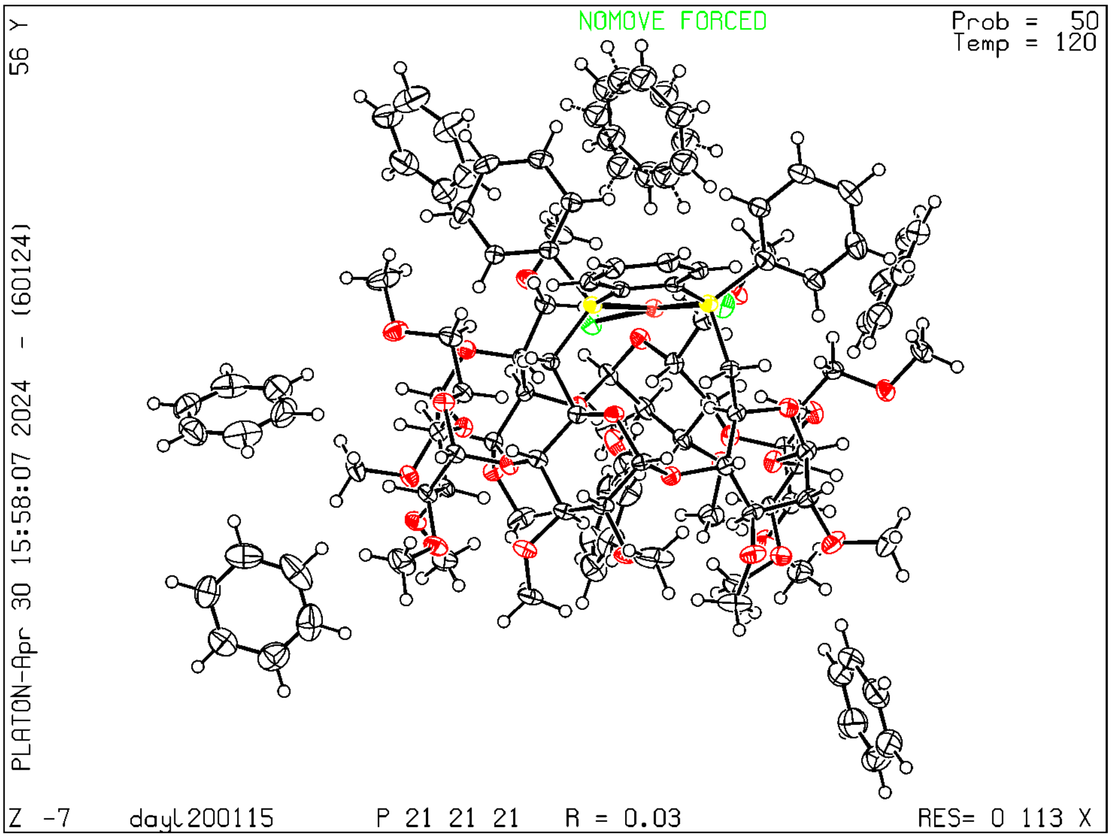

Figure S112. ORTEP structure of [$NiBr_2$(**L**[1])].

X-Ray crystal structure of complex [$NiBr_2$(**L**[2])]

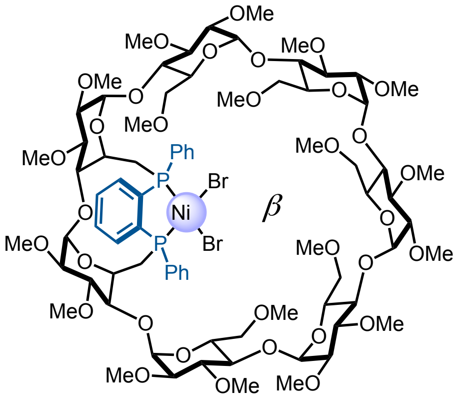

Crystal suitable for X-ray crystal-structure analysis of complex [$NiBr_2$(**L**[2])] was obtained by slow diffusion of *n*-pentane into a CH_2Cl_2 solution of [$NiBr_2$(**L**[2]). Data were collected at 120.15 K on a Bruker PHOTON-III CPAD diffractometer (Mo-Kα radiation, λ = 0.71073 Å). The structure was solved by direct methods (SHELXS-2013) and refined against F^2 using the SHELXL-2018 software.[6] The non-hydrogen atoms were refined anisotropically, using weighted full-matrix least-squares on F^2. The H-atoms were included in calculated positions and treated as riding atoms using SHELXL default parameters. Crystallographic data: formula: 2($C_{79}H_{120}Br_2NiO_{33}P_2$)•3($CH_2Cl_2$)•x(solvent) ($M$ = 4011.20 g.mol^{-1}); orange crystals, 0.12 × 0.12 × 0.10 mm; crystal system: orthorhombic, space group $P2_12_12_1$; a = 22.4866(10) Å; b = 24.0868(11) Å; c = 28.0821(15) Å; V = 19826.8(15) Å^3; Z = 4; F(000) = 8392; a total of 522226 reflections collected; 1.87° < θ < 27.95°, 47443

independent reflections with 36741 having I > 2σ(I); 2239 parameters; Final results : $R_1(F^2)$ = 0.0566; w$R_2(F^2)$ = 0.1620, Goof = 1.027. The asymmetric unit contains two molecules of metal complex together with three dichloromethane molecules. One of the methoxy groups is disordered (50% O143-C148 and 50% O14A-C14A) as well as a molecule of dichloromethane (50% Cl8 and 50% Cl9). The "squeeze" procedure[7] was implemented to eliminate residual density originating from unidentified solvent molecules. Full data collection parameters and structural data are available as CIF file (Cambridge Crystallographic Data Center deposition number CCDC 2352351).

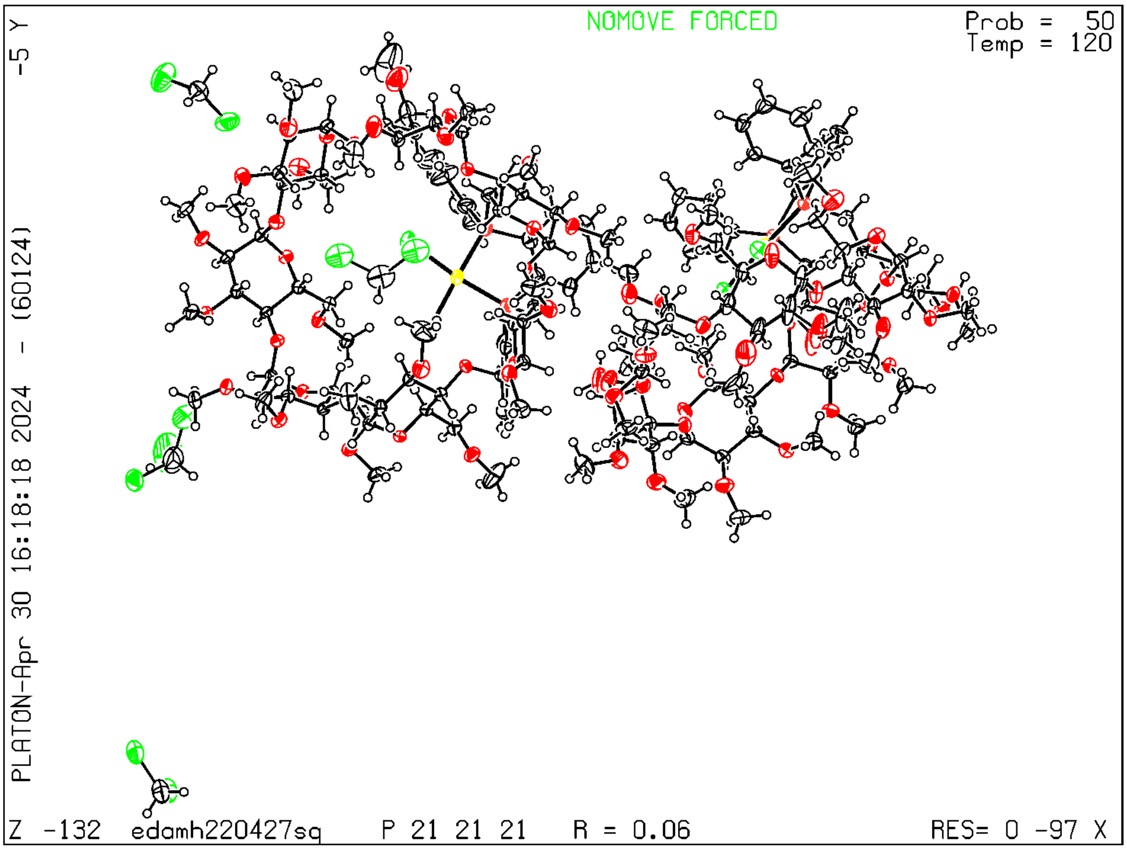

Figure S113. ORTEP structure of [$NiBr_2$(**L^2**).

X-Ray crystal structure of complex [$PdCl_2$(**L^1**)]

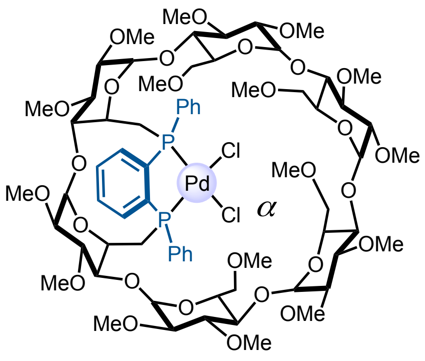

Crystal suitable for X-ray crystal-structure analysis of complex [$PdCl_2$(**L^1**)] was obtained by slow diffusion of *n*-pentane into a dichloromethane solution of [$PdCl_2$(**L^1**)]. Data were collected at 173(2) K on a CCD Saphire 3 Xcalibur diffractometer (Mo-Kα radiation, λ = 1.54178 Å). The structure was solved by direct methods (SHELXS-2013) and refined

against F^2 using the SHELXL-2018 software.[6] The non-hydrogen atoms were refined anisotropically, using weighted full-matrix least-squares on F^2. The H-atoms were included in calculated positions and treated as riding atoms using SHELXL default parameters. Crystallographic data: formula: $2(C_{70}H_{104}Cl_2O_{28}P_2Pd)\bullet11(CH_2Cl_2)$ (M = 4199.72 g.mol^{-1}); yellow crystals, 0.34 × 0.18 × 0.13 mm; crystal system: orthorhombic, space group $P2_12_12_1$; a = 14.1571(2) Å; b = 21.8506(4) Å; c = 32.0204(8) Å; V = 9905.2(3) Å^3; Z = 4; F(000) = 4336; a total of 84704 reflections collected; to 6.60° < θ < 54°, 21573 independent reflections with 11185 having $I > 2\sigma(I)$; 1081 parameters; Final results : $R_1(F^2)$ = 0.0965; w$R_2(F^2)$ = 0.2526, Goof = 1.035. The asymmetric unit contains two molecules of metal complex together with eleven dichloromethane molecules, one of them being included in the CD cavity. The external dichloromethane molecules are significantly disordered. Full data collection parameters and structural data are available as CIF file (Cambridge Crystallographic Data Center deposition number CCDC 2352375).

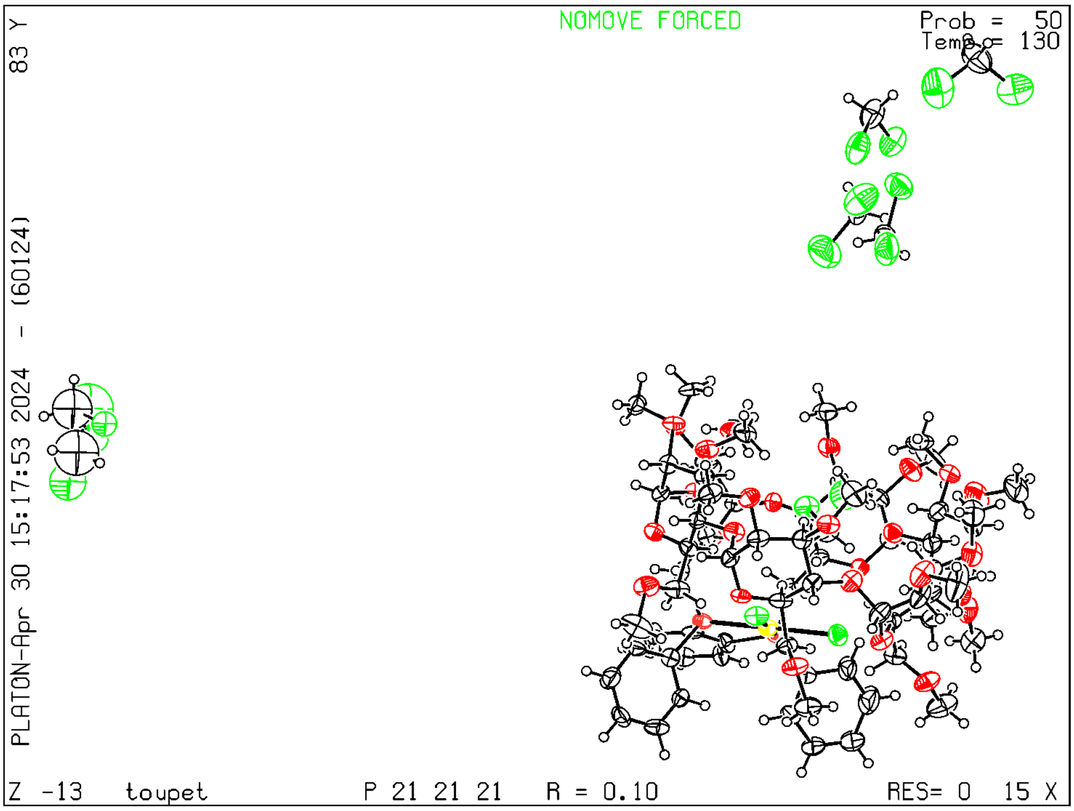

Figure S114. ORTEP structure of [PdCl$_2$(**L^1**)].

X-Ray crystal structure of complex [$PdCl_2$(**L**^{3})]

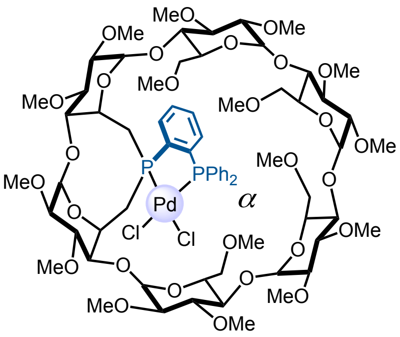

Crystal suitable for X-ray crystal-structure analysis of complex [$PdCl_2$(**L**^{3})] was obtained by slow diffusion of *n*-pentane into a benzene solution of [$PdCl_2$(**L**^{3})]. Data were collected at 173(2) K on a Bruker APEX-II Duo KappaCCD diffractometer (Cu-Kα radiation, λ = 1.54178 Å). The structure was solved by direct methods (SHELXS-2013) and refined against F^2 using the SHELXL-2014 software.[6] The non-hydrogen atoms were refined anisotropically, using weighted full-matrix least-squares on F^2. The H-atoms were included in calculated positions and treated as riding atoms using SHELXL default parameters. Crystallographic data: formula: $2(C_{70}H_{104}O_{28}P_2Cl_2Pd)\bullet 6(C_6H_6)\bullet H_2O$ (M = 3752.19 g.mol^{-1}); yellow crystals, 0.35 × 0.25 × 0.15 mm; crystal system: monoclinic, space group $P2_1$; a = 20.7357(5) Å; b = 21.4928(6) Å; c = 21.9876(6) Å; β = 104.586(2)°; V = 9483.4(4) Å^3; Z = 2; F(000) = 3956; a total of 170863 reflections collected; to 4.40° < θ < 138.48°, 33318 independent reflections with 29085 having $I > 2\sigma(I)$; 1998 parameters; Final results : $R_1(F^2)$ = 0.0530; $wR_2(F^2)$ = 0.1534, Goof = 1.063. The asymmetric unit contains two molecules of metal complex together with three benzene molecules and one water molecule. Constraints were implemented as a result of the presence of disordered benzene and water molecules. Full data collection parameters and structural data are available as CIF file (Cambridge Crystallographic Data Center deposition number CCDC 2352366).

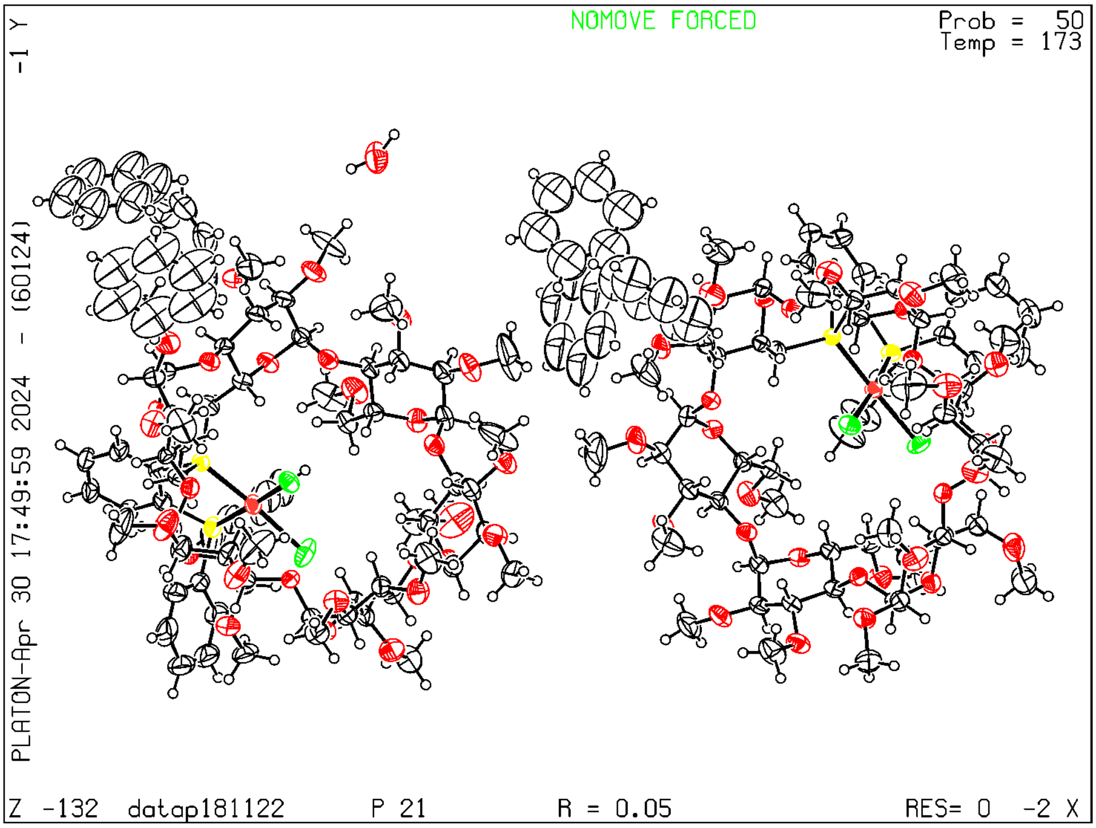

Figure S115. ORTEP structure of [$PdCl_2$(**L^3**)].

X-Ray crystal structure of complex 7a,b.

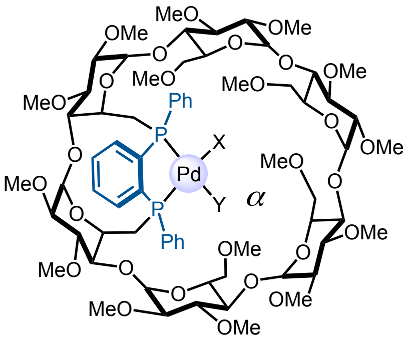

7a, X = Cl and Y = Me
7b, X = Me and Y = Cl

Crystal suitable for X-ray crystal-structure analysis of complexes **7a,b** was obtained by slow diffusion of *n*-pentane into a dichloromethane solution of **7a,b**. Data were collected at 120.15 K on a Bruker PHOTON-III CPAD diffractometer (Mo-Kα radiation, λ = 0.71073 Å). The structure was solved by direct methods (SHELXS-2013) and refined against F^2 using the SHELXL-2014 software.[6] The non-hydrogen atoms were refined anisotropically, using weighted full-matrix least-squares on F^2. The H-atoms were included in calculated positions and treated as riding atoms using SHELXL default parameters. Crystallographic data: formula: 2($C_{71}H_{107}ClO_{28}P_2Pd$)•5($CH_2Cl_2$)•x(solvent) ($M$ = 3649.33 g.mol^{-1}); orange crystals, 0.20 × 0.20 × 0.15 mm; crystal system: orthorhombic, space group $P2_12_12_1$; a = 14.1758(7) Å; b = 21.8479(13) Å; c = 31.863(2) Å; V = 9868.3(10) Å^3; Z = 2; $F(000)$ = 3820; a total of 154369 reflections collected; to 3.85° < θ < 55.96 °, 23661independent

reflections with 14710 having $I > 2\sigma(I)$; 1004 parameters; Final results : $R_1(F^2)$ = 0.0952; $wR_2(F^2)$ = 0.2920, Goof = 1.007. The asymmetric unit contains two molecules of metal complex together with five dichloromethane molecules, one of them being included in the CD cavity, as well as unidentified solvent molecules. The "squeeze" procedure[7] was implemented to eliminate residual density originating from the latter. The two complexes (**7a** and **7b**) are present in a 3/7 proportion in the asymmetric unit. The C78/Cl1A and C78A/Cl1 atoms are located at the same positions. The Pd-Cl1/Pd-C78 and Pd-Cl1A/Pd-C78A distances are therefore averaged. One of the methoxy groups is disordered (47% O23-C28 and 53% O23-C28A) Full data collection parameters and structural data are available as CIF file (Cambridge Crystallographic Data Center deposition number CCDC 2352367).

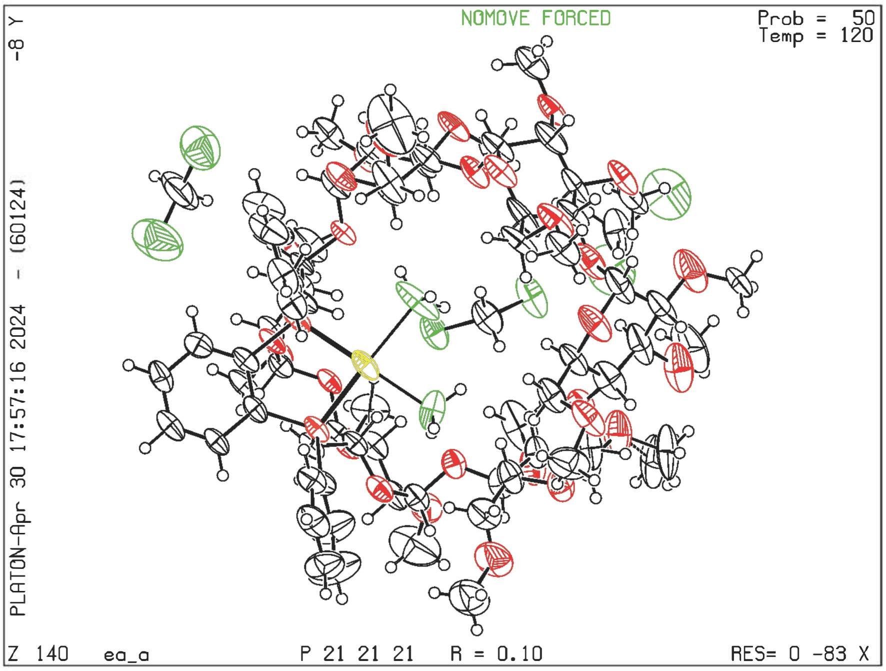

Figure S116. ORTEP structure of **7a,b**.

6. Computational details

Geometry optimization calculations for all stationary points were performed with ORCA[8] 5.0.4 version with both methods used. For the density functional theory (DFT) calculations, the Perdew-Burke-Ernzerhof (PBE)[9] functional was applied, along with the Grimme's dispersion corrections with Becke-Johnson damping D3(BJ)[10], and the def2-TZVP[11] basis set was used with the def2/J[12] auxiliary basis. The Conductor-like Polarizable Continuum Model (CPCM)[13] was employed as implicit solvent model, with toluene as solvent (ε = 2.40). The convergence criteria for the self-consistent field (SCF) were set to "VeryTightSCF", and the integration grid accuracy for the geometry optimization was set to "DefGrid3".[8] For the extended semiempirical tight-binding model, the GFN2-xTB[14] method was used with the analytical linearized Poisson–Boltzmann (ALPB)[15] implicit solvent model, with toluene as solvent. Different approaches were employed to search the transition states (TSs): either relaxed surface scan followed by TS optimization (Scan-TS) or Nudged Elastic Band Transition State (NEB-TS) was used to locate the TS geometry, followed by a new optimization of the transition state using the eigenvector following (EF) algorithm. The vibrational modes were analytically calculated for all geometry optimization and used to identify if the stationary points are energy minima or a saddle point, that is a transition state. The geometries were visualized with the Chemcraft package.[16]

6.1. Independent Gradient Model (IGM) analysis

The IGM[17] Δg^{inter} descriptors for the selected fragments were computed using the IGMPLOT[17] (version 3.10) program. The calculations were performed using the electron density computed from the wavefunctions obtained from single-point calculations at the PBE-D3(BJ)/def2-TZVP/CPCM(toluene) level of theory on the geometries optimized at the GFN2-xTB/ALPB(toluene) level of theory using the ORCA[8] 5.0.4 package. The images with the IGM surfaces were plotted with VMD.[18]

6.2. Benchmark studies

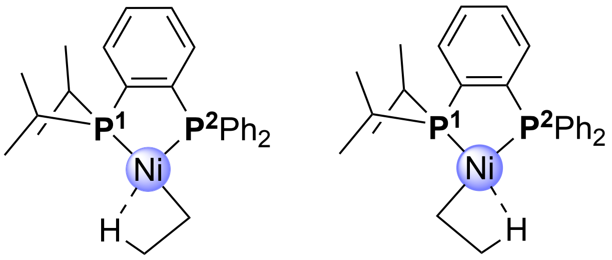

L^5-Ia **L^5-Ib**

Figure S117. Active species **L^5-Ia** (with ethyl group *trans* to P^1) and **L^5-Ib** (with ethyl group *trans* to P^2) complexes.

Cavity-free Ni^{II} complexes comprising ligand **L^5** were selected for the basic benchmark study. It consisted in comparing the calculations based on density functional theory (DFT) with those obtained from the semiempirical tight-binding GFN2-xTB[19] method, the steps of the first ethylene insertion into the Ni-C bond during the ethylene dimerization reaction were calculated. The active species with ethyl group *trans* to P^1 (**L^5-Ia**) or *trans* to P^2 (**L^5-Ib**) (see Scheme 3 for labeling) is an ethyl complex stabilized by a β-agostic interaction (Figure S117).

L^5-II represents the π-complex after ethylene coordination, that can occur *trans* to P^2 (**L^5-IIa**) which is less stable and more active or *trans* to P^1 (**L^5-IIb**) which is more stable and less reactive. Following the mechanism **L^5-IIIa** and **L^5-IIIb** corresponds to the π-complex with the ethylene oriented *syn* coplanar to the alkyl group as the "reactant complex" in relation to the transition state of the ethylene insertion into the Ni-C bond **L^5-TSII-IVa** and **L^5-TSII-IVb** (Figures S118). Following the insertion, **L^5-IVa** or **L^5-IVb** is the complex with butyl formed with a γ-agostic interaction, and **L^5-Va** or

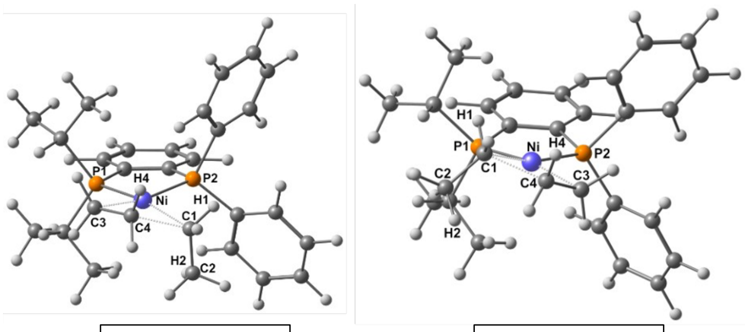

L^5-TSII-IVa **L^5-TSII-IVb**

Figure S118. Optimized geometries for **L^5-TSII-IVa** and **L^5-TSII-IVb** computed at the PBE-D3(BJ)/def2-TZVP/CPCM(toluene) level of theory. Color code for atoms: hydrogen (white), carbon (gray), phosphorus (orange), nitrogen (blue), and nickel (violet).

L^5-Vb is the complex with butyl formed with a β-agostic interaction. All energies and profiles for both methods are provided below (Figures S119-S122)

The comparison between the methods (Figures S119, and S120) demonstrated that the GFN2-xTB method underestimates the energies of the stationary points. This is possibly related to the method's tendency to overestimate covalent bond energies.[19] However, the energy underestimation being systematic, the overall trend in the energy profile was preserved. The geometric differences between the stationary points optimized with the two different methods were assessed through root-mean-square distances (RMSDs) calculated between two molecules. The RMSD calculations were performed including all atoms (except H) using the Chemcraft[16] application and presented values ranging between 0.205 Å and 0.871 Å (Table S1), which are reasonable compared to other systems.[20] Additionally, the GFN2-xTB method overall overestimates the bond lengths of some important bonds in the [$NiBr_2$(L^5)] complexes (**P^P** ligand) such as Ni–P1, Ni–N1, Ni–P2, Ni-C3, Ni-C4 and C1–C4, following the atom labels in Figure 3, as compared to DFT (Tables S22-S5). In contrast, the bond length of the C3–C4 bond was consistently underestimated. Ethylene coordination in the **L^5-IIa** and **L^5-IIb** complexes deviates from ideal square-plane geometry with both DFT and GFN2-xTB method. Similar out-of-plane geometry deviations on the ethylene coordination in M^{II}-α-diimines complexes were observed in previous studies.[21,22] The deviation from the expected square-planar geometry for ethylene coordination and insertion is attributed to strong steric interactions with the ligands. Even in the absence of CD cavity, this type of diphosphanes displays considerable hindrance. These strong interactions with the ligands may partially explain the deviations observed in **L^5-II**, even with DFT method.

Throughout the benchmark, GFN2-xTB proved suitable to the study of the Ni^{II}-catalyzed ethylene dimerization with an adequate compromise between computational cost and results' quality, with trends similar to DFT in which the difference between the activation barriers ($\Delta H^‡$) with DFT and GFN2-xTB method was around 1 kcal/mol.

Comparing enthalpy profile of the reaction with the [$NiBr_2$(L^5)] complexes (**P^P** ligand) with ethyl group coordinated *trans* to P^1 or *trans* to P^2, it was verified that the active complex **L^5-Ia** was ~1 kcal/mol more stable than **L^5-Ib** while **L^5-IIa** showed less stable than **L^5-IIb** with DFT (~3 kcal/mol) and GFN2-xTB (~4 kcal/mol) method (Figures S119 and S120, respectively). The activation barrier calculated with DFT method showed $\Delta H^‡$ = 14 kcal/mol for **L^5-TSII-IVa** and $\Delta H^‡$ = 20 kcal/mol for **L^5-TSII-IVb**

complex and $\Delta H^{\ddagger}$ = 13 kcal/mol for **L^5-TSII-IVa** and $\Delta H^{\ddagger}$ = 18 kcal/mol for **L^5-TSII-IVb** with GFN2-xTB method. This behavior demonstrates that PPh_2 acts as a STI with respect to P^iPr_2, which acts as WTI in this system once **L^5-IIb** is more stable and has the higher ethylene insertion barrier. As the active complex **L^5-Ia** is more stable and **L^5-IIa** is more reactive, the reaction path starting with the ethyl *trans* to P^1 is considered as the favored path for ethylene dimerization.

6.3. 1-butene rotation

Reaching the **L^5-VII** and **L^3-VII** intermediates requires rotating the olefin around the axis of the Ni-olefin bond. Single-point energy calculations were performed for 90° and 180° rotations around the P-Ni-X-C3 dihedral angle, where X represents the center of the C3-C4 bond. The energy analysis of these intermediates revealed that a 90° rotation in **L^5-VI** (**L^5-VI_0°)** leads to **L^5-VIa_90°** (-22 kcal/mol) and **L^5-VIb_90°** (-31 kcal/mol), making them 15 kcal/mol and 2 kcal/mol less stable than **L^5-VI_0°,** respectively. Similarly, a 180° rotation resulted in **L^5-VIa_180°** (-21 kcal/mol) and **L^5-VIb_180°** (-31 kcal/mol), where **L^5-VIa_180°** is 15 kcal/mol less stable, while **L^5-VIb_180°** is 2 kcal/mol more stable than their respective **L^5-VIa_0°** and **L^5-VIb_0°** conformers. Conversely, the cavity-shaped complexes **L^3-VIa_90°** (-4 kcal/mol) and **P^P^{CD}-VIb_90°** (90 kcal/mol) are 30 kcal/mol and 126 kcal/mol less stable than **L^3-VIa_0°** and **L^3-VIb_0°**, respectively. Similarly, **L^3-VIa_180°** (1892 kcal/mol) and **L^3-VIb_180°** (94 kcal/mol) are 1925 kcal/mol and 130 kcal/mol less stable than their respective **L^3-VIa_0°** and **L^3-VIb_0°** conformers. This demonstrates that the CD cavity of the **P^P^{CD}** ligand imposes considerable steric hindrance on the encapsulated 1-butene and that rotation of the latter is more energy-demanding within the CD cavity. As a result, isomerization is much less favorable in the supramolecular CD environment. This finding aligns with experimental results showing that the proportion of α-C4 olefin produced is higher when the CD environment is involved.

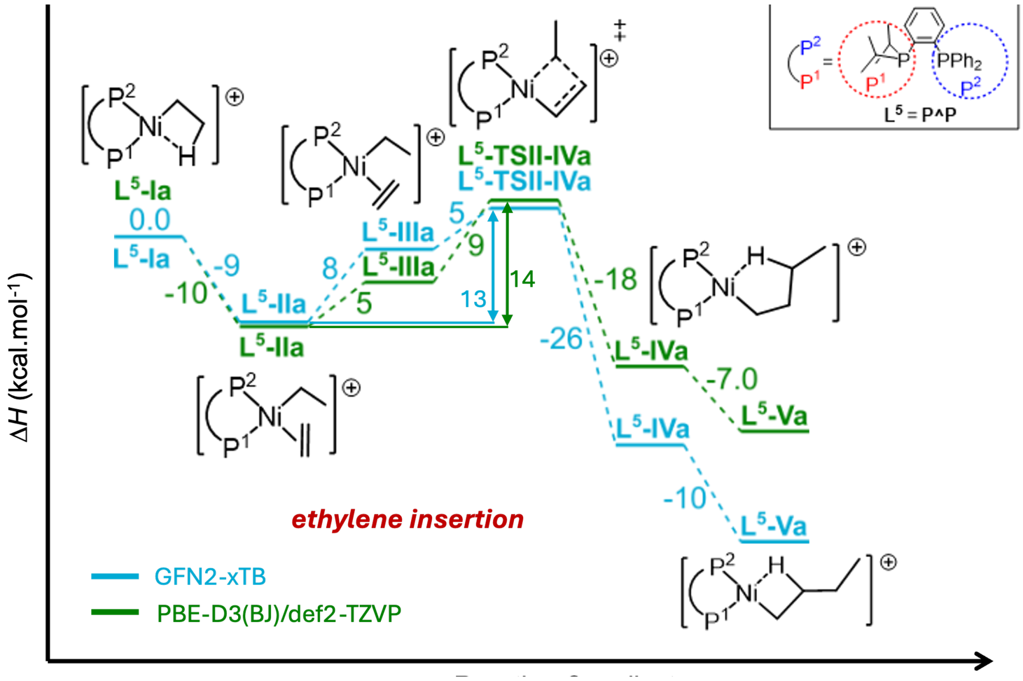

Figure S119. Energy profile for the first ethylene insertion into the Ni-C bond catalyzed by complex [NiBr$_2$(**L^5**)] (**P^P** ligand) for path **a**. Relative enthalpy values are given in kcal/mol relative to the respective active species (**L^5-Ia**) obtained from geometry optimizations at the PBE-D3(BJ)/def2-TZPV/CPCM(toluene) level of theory (in green) and at the GFN2-xTB/ALPB(toluene) level of theory (in light blue).

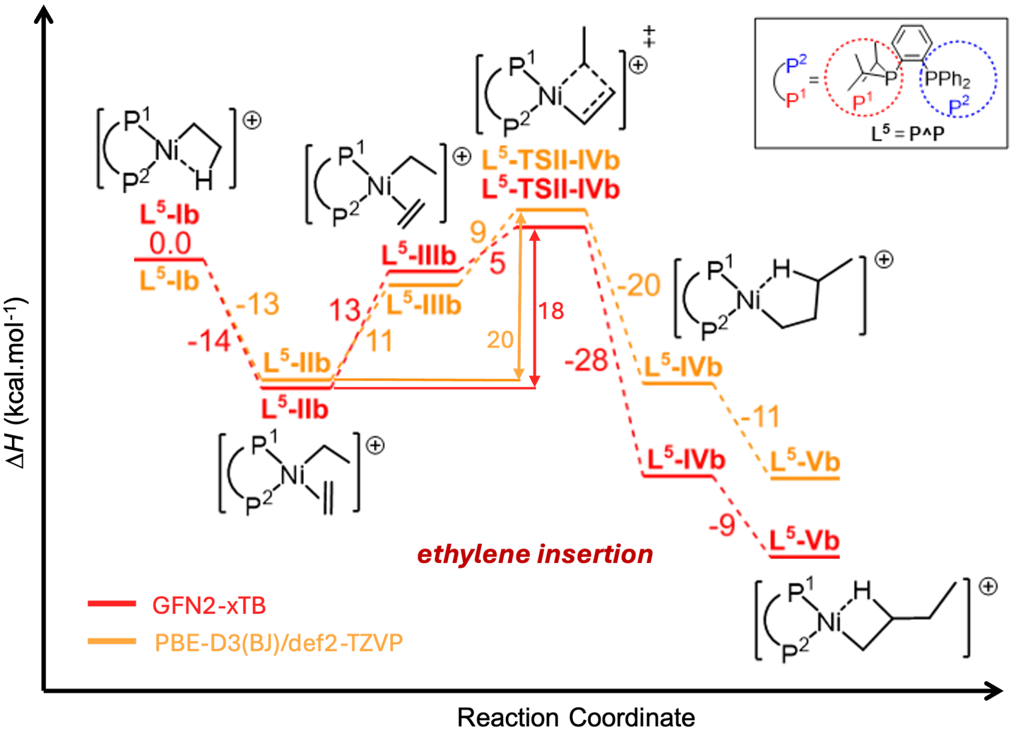

Figure S120. Energy profile for the first ethylene insertion into the Ni-C bond catalyzed with the complex [NiBr$_2$(**L^5**)] (**P^P** ligand) with the ethyl group coordinated in the active complex *trans* to P_2 = PPh_2 (**L^5-Ib**). Relative enthalpy values in kcal/mol in relation to their respective **L^5-Ib** computed at the PBE-D3(BJ)/def2-TZPV/CPCM(toluene) level of theory (in orange) and at the GFN2-xTB/ALPB(toluene) level of theory (in red).

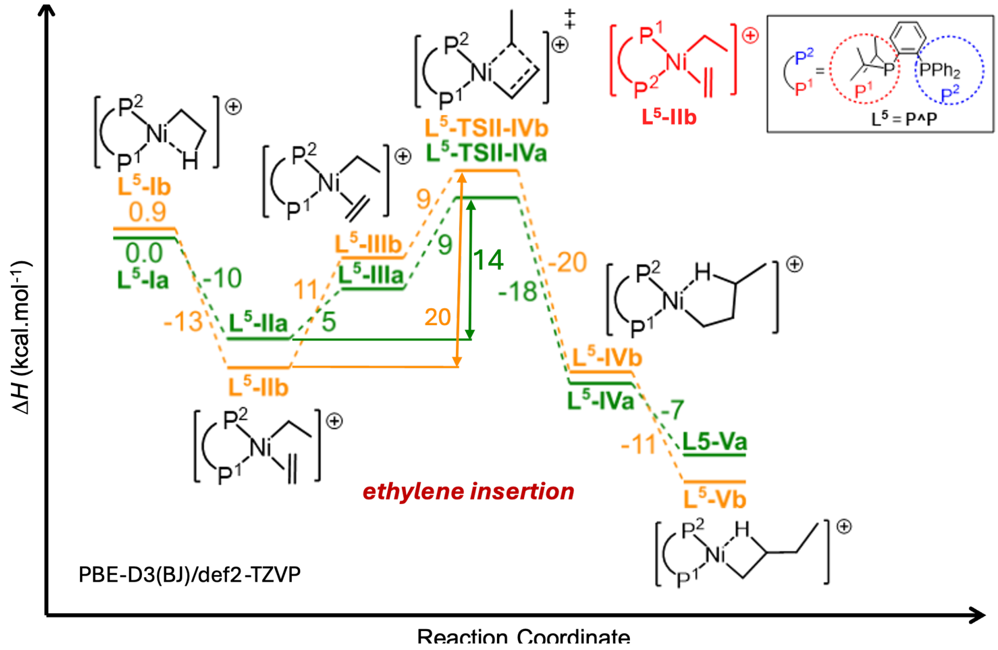

Figure S121. Energy profile for the first ethylene insertion into the Ni-C bond catalyzed with the complex [$NiBr_2$(**L⁵**)] (**P^P** ligand) with the ethyl group coordinated in the active complex *trans* to P_1 = P^iPr_2 (**L⁵-Ia**, in green) or *trans* to P_2 = PPh_2 (**L⁵-Ib**, in orange). Relative enthalpy values in kcal/mol in relation to **L⁵-Ia** computed at the PBE-D3(BJ)/def2-TZPV/CPCM(toluene) level of theory.

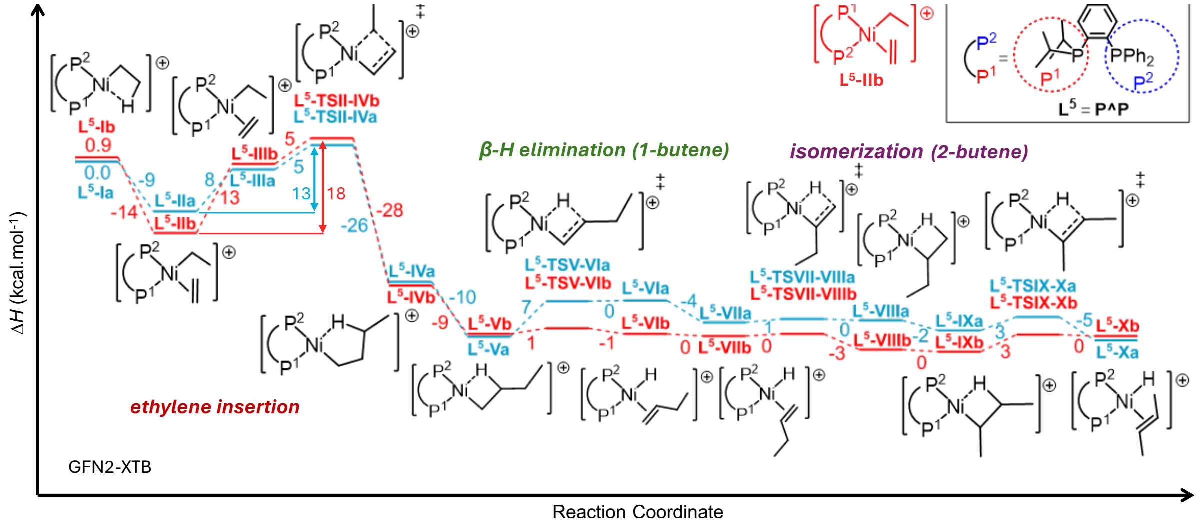

Figure S122. Energy profile for the first ethylene insertion into the Ni-C bond catalyzed by complex [$NiBr_2$(**L⁵**)] (**P^P** ligand) for path **a** in light blue or path **b** in red. Relative enthalpy values are given in kcal/mol relative to the respective active species (**L⁵-Ia)** obtained from geometry optimizations at the GFN2-xTB/ALPB(toluene) level of theory.

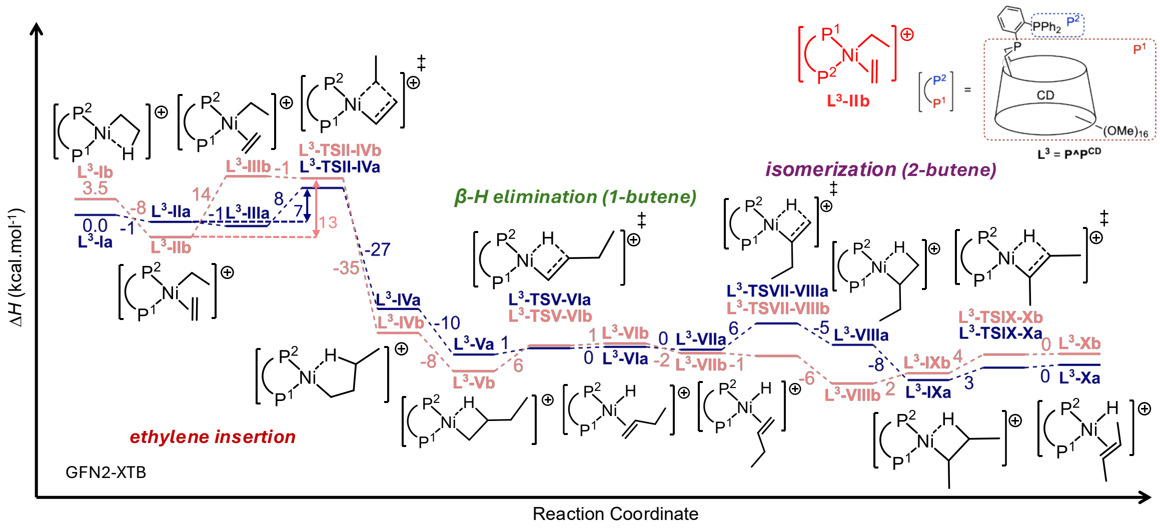

Figure S123. Energy profile for the first ethylene insertion into the Ni-C bond catalyzed by complex [NiBr2(**L3**)] (**P^PCD** ligand) for path **a** in dark blue or path **b** in light red). Relative enthalpy values are given in kcal/mol relative to the respective active species (**L3-Ia**) obtained from geometry optimizations at the GFN2-xTB/ALPB(toluene) level of theory.

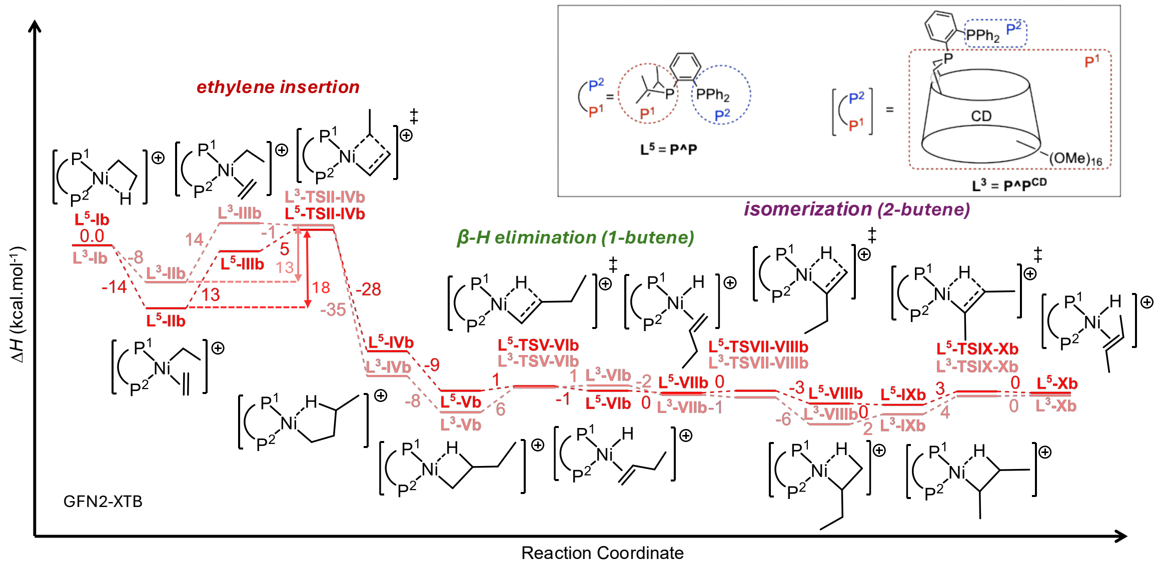

Figure S124. Energy profile for the ethylene dimerization catalyzed with the complex [NiBr2(**L5**)] (**P^P** ligand) in red and the complex [NiBr2(**L3**)] (**P^PCD** ligand) in light red. Relative enthalpy values are given in kcal/mol relative to the respective active species (**L5-Ib** for complex [NiBr2(**L5**)] and **L3-Ib** for complex [NiBr2(**L3**)]) obtained from geometry optimizations at the GFN2-xTB/ALPB(toluene) level of theory.

Table S1. Relative enthalpy values (Δ*H*) in kcal/mol of the stationary points of the ethylene insertion on the Ni-C1 bond catalyzed by complex [$NiBr_2$(**L⁵**)] (**P^P** ligand) for path **a** and path **b** obtained from geometry optimizations at the PBE-D3(BJ)/def2-TZVP/CPCM(toluene) level of theory (DFT) and at the GFN2-xTB/ALPB(toluene) level of theory. Root Mean Square Distances (RMSD) between the structures computed with different methods.

	DFT	GFN2-xTB		
Structure	Δ*H* (kcal/mol)	Δ*H* (kcal/mol)	RMSD[a] (Å)	RMSD[b] (Å)
**L^5-Ia**	0.0	0.0	0.714	0.858
**L^5-IIa**	-9.8	-9.3	0.205	0.286
**L^5-IIIa**	-5.0	-1.4	0.447	0.787
**L^5-TSII-IVa**	3.9	3.1	0.382	0.658
**L^5-IVa**	-14.1	-22.6	0.536	0.933
**L^5-Va**	-21.1	-33.0	0.548	0.676
**L^5-Ib**	0.0	0.0	0.629	0.740
**L^5-IIb**	-13.5	-14.4	0.368	0.518
**L^5-IIIb**	-2.9	-1.3	0.220	0.262
**L^5-TSII-IVb**	5.6	3.6	0.437	0.525
**L^5-IVb**	-13.9	-24.3	0.871	1.113
**L^5-Vb**	-24.6	-33.4	0.524	0.651

[a]RMSD for all atoms except H. [b]RMSD for all atoms including H.

Table S2. A-B distances in Å of the stationary points of the ethylene insertion in the Ni-C1 bond catalyzed by the complex [$NiBr_2$(**L⁵**)] (**P^P** ligand), for path **a**, computed at the PBE-D3(BJ)/def2-TZVP/CPCM(toluene) level of theory.

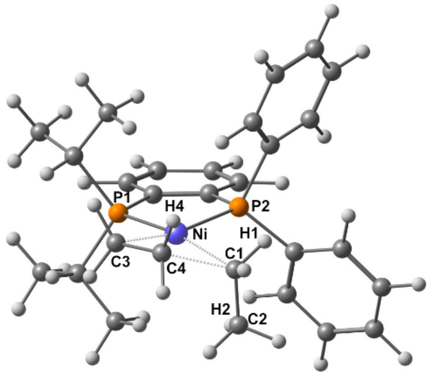

L^5-TSII-IVa

	A-B distance (Å)											
Structure	Ni-P1	Ni-P2	Ni-C1	Ni-C3	Ni-C4	C3-C4	C1-C4	C1-H1	C2-H2	C4-H4	Ni-H1	Ni-H2	Ni-H4
**L^5-Ia**	2.181	2.118	1.928	-	-	-	-	1.092	1.220	-	2.573	1.649	
**L^5-IIa**	2.250	2.189	1.966	2.102	2.068	1.390	2.909	1.091	1.174	1.092	2.647	1.794	2.604
**L^5-IIIa**	2.240	2.160	2.060	2.103	2.088	1.384	2.612	1.098	1.100	1.093	2.701	2.995	2.607
**L^5-TSII-IVa**	2.161	2.196	2.202	1.954	2.138	1.444	2.002	1.096	1.099	1.092	2.420	2.705	2.758
**L^5-IVa**	2.109	2.186	2.444	1.946	2.536	1.521	1.549	1.140	1.100	1.101	1.812	3.086	2.901
**L^5-Va**	2.122	2.161	3.305	1.939	2.244	1.499	1.533	1.100	1.100	1.189	3.827	4.545	1.828

Table S3. A-B distances in Å of the stationary points of the ethylene insertion in the Ni-C1 bond catalyzed by the complex [$NiBr_2$(**L⁵**)] (**P^P** ligand), for path **b**, computed at the PBE-D3(BJ)/def2-TZVP/CPCM(toluene) level of theory.

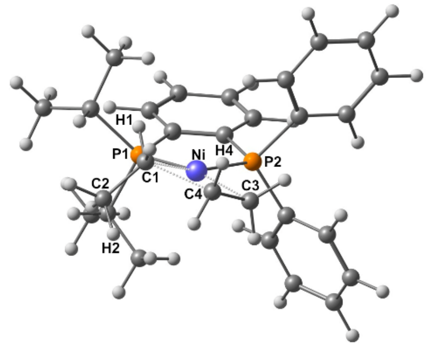

L^5-TSII-IVb

	A-B distance (Å)											
Structure	Ni-P1	Ni-P2	Ni-C1	Ni-C3	Ni-C4	C3-C4	C1-C4	C1-H1	C2-H2	C4-H4	Ni-H1	Ni-H2
**L^5-Ib**	2.139	2.171	1.929	-	-	-	-	1.092	1.212	-	2.583	1.650	-
**L^5-IIb**	2.194	2.223	1.966	2.115	2.095	1.387	2.936	1.090	1.160	1.092	2.674	1.815	2.629
**L^5-IIIb**	2.198	2.216	2.026	2.099	2.086	1.386	2.630	1.097	1.099	1.088	2.499	3.363	2.697
**L^5-TSII-IVb**	2.232	2.141	2.188	1.953	2.148	1.441	1.999	1.100	1.100	1.091	2.256	3.491	2.860
**L^5-IVb**	2.212	2.085	2.470	1.939	2.463	1.519	1.551	1.139	1.101	1.103	1.828	4.341	2.723
**L^5-Vb**	2.179	2.114	3.212	1.933	2.114	1.475	1.533	1.102	1.100	1.236	3.178	4.888	1.625

Table S4. A-B distances in Å of the stationary points of the ethylene insertion in the Ni-C1 bond catalyzed by the complex [$NiBr_2$(**L^5**)] (**P^P** ligand), for path **a**, computed at the GFN2-xTB/ALPB(toluene) level of theory.

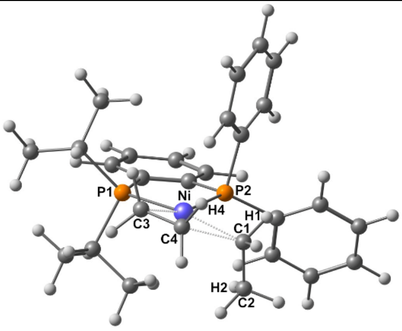

L^5-TSII-IVa

	A-B distance (Å)												
Structure	Ni-P1	Ni-P2	Ni-C1	Ni-C3	Ni-C4	C3-C4	C1-C4	C1-H1	C2-H2	C4-H4	Ni-H1	Ni-H2	Ni-H4
**L^5-Ia**	2.177	2.141	1.899	-	-	-	-	1.090	1.201	-	2.577	1.552	-
**L^5-IIa**	2.244	2.182	1.930	2.493	2.419	1.340	3.277	1.088	1.170	1.084	2.809	1.652	2.631
**L^5-IIIa**	2.298	2.162	2.037	2.210	2.231	1.352	2.792	1.097	1.091	1.083	2.694	3.088	2.770
**L^5-TSII-IVa**	2.239	2.203	2.129	1.968	2.141	1.408	2.199	1.099	1.093	1.084	2.457	2.773	2.794
**L^5-IVa**	2.171	2.213	2.281	1.899	2.500	1.518	1.552	1.128	1.100	1.095	1.765	2.324	3.428
**L^5-Va**	2.157	2.167	3.250	1.895	2.134	1.477	1.529	1.093	1.088	1.201	3.242	4.877	1.549

Table S5. A-B distances in Å of the stationary points of the ethylene insertion in the Ni-C1 bond catalyzed by the complex [NiBr$_2$(**L^5**)] (**P^P** ligand), for path **b**, computed at the GFN2-xTB/ALPB(toluene) level of theory.

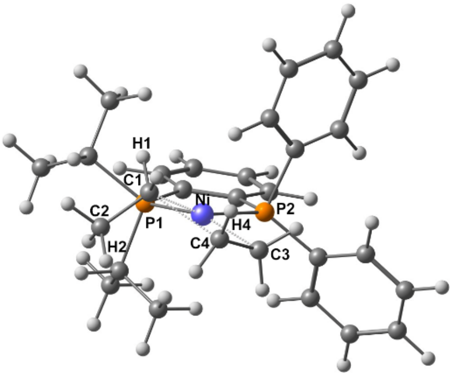

L^5-TSII-IVb

Structure	A-B distance (Å)											
	Ni-P1	Ni-P2	Ni-C1	Ni-C3	Ni-C4	C3-C4	C1-C4	C1-H1	C2-H2	C4-H4	Ni-H1	Ni-H2	Ni-H4
**L^5-Ib**	2.158	2.181	1.898	-	-	-	-	1.090	1.185	-	2.574	1.577	-
**L^5-IIb**	2.205	2.211	1.920	2.587	2.457	1.337	3.286	1.088	1.171	1.085	2.615	1.653	2.803
**L^5-IIIb**	2.244	2.245	1.993	2.243	2.222	1.354	2.787	1.099	1.087	1.082	2.509	3.329	2.778
**L^5-TSII-IVb**	2.270	2.184	2.097	1.971	2.109	1.414	2.184	1.106	1.088	1.082	2.269	3.493	2.837
**L^5-IVb**	2.224	2.119	2.497	1.915	2.550	1.520	1.549	1.126	1.089	1.095	1.761	4.326	2.941
**L^5-Vb**	2.186	2.134	3.257	1.901	2.128	1.476	1.529	1.093	1.088	1.210	3.263	4.869	1.534

Table S6. A-B distances in Å of the stationary points of the ethylene insertion in the Ni-C1 bond catalyzed by the complex [$NiBr_2$(**L^3**)] (**P^P^{CD}** ligand), for path **a**, computed at the GFN2-xTB/ALPB(toluene) level of theory.

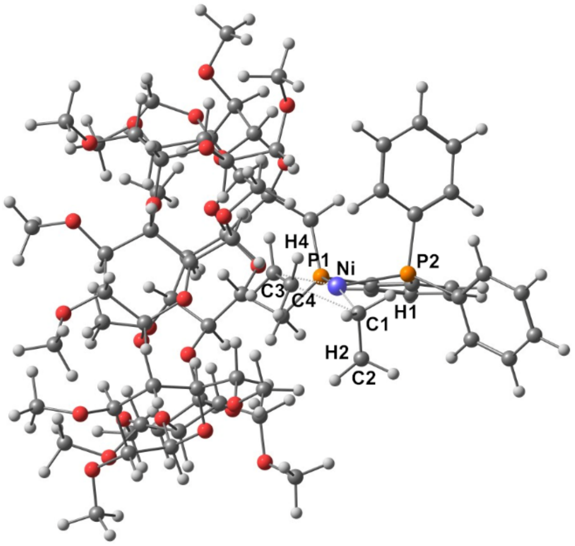

L^3-TSII-IVa

	A-B distance (Å)												
Structure	Ni-P1	Ni-P2	Ni-C1	Ni-C3	Ni-C4	C3-C4	C1-C4	C1-H1	C2-H2	C4-H4	Ni-H1	Ni-H2	Ni-H4
**L^3-Ia**	2.181	2.153	1.894	-	-	-	-	1.090	1.180	-	2.567	1.562	-
**L^3-IIa**	2.232	2.209	1.937	2.330	2.322	1.347	3.127	1.091	1.128	1.084	2.689	2.035	2.784
**L^3-IIIa**	2.245	2.186	1.934	2.447	2.491	1.340	3.064	1.090	1.150	1.082	2.607	1.747	2.931
**L^3-TSII-IVa**	2.213	2.263	2.080	1.985	2.152	1.401	2.216	1.098	1.110	1.085	2.462	2.243	2.762
**L^3-IVa**	2.151	2.234	2.255	1.905	2.533	1.519	1.548	1.130	1.113	1.095	1.824	2.08	3.363
**L^3-Va**	2.194	2.195	3.354	1.925	2.139	1.466	1.527	1.094	1.089	1.214	4.144	3.904	1.527

Table S7. A-B distances in Å of the stationary points of the ethylene insertion in the Ni-C1 bond catalyzed by complex [$NiBr_2$(**L^3**)] (**P^P^{CD}** ligand), for path **b**, computed at the GFN2-xTB/ALPB(toluene) level of theory.

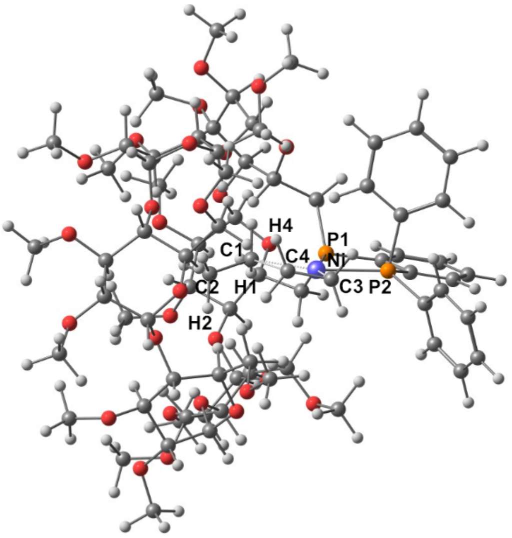

L^3-TSII-IVb

	A-B distance (Å)											
Structure	Ni-P1	Ni-P2	Ni-C1	Ni-C3	Ni-C4	C3-C4	C1-C4	C1-H1	C2-H2	C4-H4	Ni-H1	Ni-H2	Ni-H4
**L^3-Ib**	2.186	2.197	1.917	-	-	-	-	1.089	1.195	-	2.559	1.550	-
**L^3-IIb**	2.178	2.214	1.924	2.569	2.418	1.339	3.313	1.088	1.180	1.084	2.606	1.590	2.805
**L^3-IIIb**	2.314	2.273	2.049	2.206	2.177	1.366	2.619	1.095	1.087	1.084	2.457	3.714	2.716
**L^3-TSII-IVb**	2.335	2.248	2.073	2.068	2.109	1.389	2.407	1.096	1.089	1.084	2.480	3.653	2.712
**L^3-IVb**	2.234	2.144	2.279	1.926	2.571	1.527	1.546	1.125	1.089	1.093	1.708	4.447	3.346
**L^3-Vb**	2.186	2.158	3.351	1.908	2.128	1.477	1.527	1.093	1.090	1.189	4.172	3.809	1.547

Table S8. Electronic Energy (*E*). Enthalpy (*H*). and Gibbs free energy (*G*) in hartree for the stationary points of the ethylene insertion into the Ni-C1 bond catalyzed by the complex [$NiBr_2$(**L^5**)] (**P^P** ligand), for path **a**, computed at the PBE-D3(BJ)/def2-TZVP/CPCM(toluene) level of theory.

Structure	*E* (hartree)	*H* (hartree)	*G* (hartree)	Frequency (cm^{-1})
**Ethylene**	-78.50412298	-78.45063361	-78.47554572	
**L^5-Ia**	-3200.20026	-3199.662118	-3199.75253	
**L^5-IIa**	-3278.723462	-3278.128419	-3278.223205	
**L^5-IIIa**	-3278.717335	-3278.12071	-3278.216503	
**L^5-TSII-IVa**	-3278.702163	-3278.10654	-3278.201783	-362.31
**L^5-IVa**	-3278.732638	-3278.135226	-3278.230788	
**L^5-Va**	-3278.745555	-3278.146319	-3278.242682	

Table S9. Electronic Energy (*E*). Enthalpy (*H*). and Gibbs free energy (*G*) in hartree for the stationary points of the ethylene insertion into the Ni-C1 bond catalyzed by the complex [$NiBr_2$(**L⁵**)] (**P^P** ligand), for path **b**, computed at the PBE-D3(BJ)/def2-TZVP/CPCM(toluene) level of theory.

Structure	*E* (hartree)	*H* (hartree)	*G* (hartree)	Frequency (cm^{-1})
**Ethylene**	-78.50412298	-78.45063361	-78.47554572	
**L^5-Ib**	-3200.19898928	-3199.66067075	-3199.75127797	
**L^5-IIb**	-3278.72789228	-3278.13275627	-3278.22717440	
**L^5-IIIb**	-3278.71234509	-3278.11595157	-3278.21174464	
**L^5-TSII-IVb**	-3278.69792488	-3278.10233438	-3278.19690455	-347.35
**L^5-IVb**	-3278.73076222	-3278.13353000	-3278.22961078	
**L^5-Vb**	-3278.74645806	-3278.15055561	-3278.24667797	

Table S10. Electronic Energy (*E*). Enthalpy (*H*). and Gibbs free energy (*G*) in hartree for the stationary points of the ethylene insertion into the Ni-C1 bond catalyzed by the complex [$NiBr_2$(**L⁵**)] (**P^P** ligand), for path **a**, computed at the GFN2-xTB/ALPB(toluene) level of theory.

Structure	*E* (hartree)	*H* (hartree)	*G* (hartree)	Frequency (cm^{-1})
**Ethylene**	-6.27348526	-6.22125465	-6.24724294	
**L^5-Ia**	-82.05697676	-81.52062876	-81.61191807	
**L^5-IIa**	-88.35021411	-87.75676004	-87.85562759	
**L^5-IIIa**	-88.33994204	-87.74415713	-87.84090599	
**L^5-TSII-IVa**	-88.33136157	-87.73687361	-87.83223624	-338.91
**L^5-IVa**	-88.37871675	-87.77784828	-87.87335048	
**L^5-Va**	-88.38991974	-87.79442678	-87.89071751	
**L^5-TSV-VIa**	-88.37674212	-87.78370355	-87.87967902	-328.74
**L^5-VIa**	-88.37735607	-87.78341953	-87.88151063	
**L^5-VIIa**	-88.38362082	-87.7901272	-87.88744829	
**L^5-TSVII-VIIIa**	-88.38155592	-87.78907405	-87.88554035	-447.54
**L^5-VIIIa**	-88.38590852	-87.7894979	-87.88603687	
**L^5-IXa**	-88.38876966	-87.79260923	-87.88906215	
**L^5-TSIX-Xa**	-88.3809548	-87.78839378	-87.88433793	-443.47
**L^5-Xa**	-88.38873504	-87.79563154	-87.89330683	

Table S11. Electronic Energy (*E*). Enthalpy (*H*). and Gibbs free energy (*G*) in hartree for the stationary points of the ethylene insertion into the Ni-C1 bond catalyzed by the complex [$NiBr_2$(**L5**)] (**P^P** ligand), for path **b**, computed at the GFN2-xTB/ALPB(toluene) level of theory.

Structure	*E* (hartree)	*H* (hartree)	*G* (hartree)	Frequency (cm^{-1})
**Ethylene**	-6.27348526	-6.22125465	-6.24724294	
**L^5-Ib**	-82.05570396	-81.51914340	-81.61020222	
**L^5-IIb**	-88.35693084	-87.76330480	-87.86094452	
**L^5-IIIb**	-88.33856003	-87.74245680	-87.83995571	
**L^5-TSII-IVb**	-88.32909424	-87.73473239	-87.83070846	-342.71
**L^5-IVb**	-88.37843155	-87.77905567	-87.87465722	
**L^5-Vb**	-88.38911185	-87.79364439	-87.88981024	
**L^5-TSV-VIb**	-88.38420711	-87.79181950	-87.88772742	-539.75
**L^5-VIb**	-88.38708629	-87.79366661	-87.89069563	
**L^5-VIIb**	-88.38776999	-87.79433690	-87.89089946	
**L^5-TSVII-VIIIb**	-88.38597771	-87.79364436	-87.88924938	-484.05
**L^5-VIIIb**	-88.39402985	-87.79830393	-87.89424117	
**L^5-IXb**	-88.39442843	-87.79889686	-87.89523637	
**L^5-TSIX-Xb**	-88.38603205	-87.79385833	-87.88964126	-388.33
**L^5-Xb**	-88.38773752	-87.79437689	-87.89128301	

Table S12. Electronic Energy (*E*). Enthalpy (*H*). and Gibbs free energy (*G*) in hartree for the stationary points of the ethylene insertion into the Ni-C1 bond catalyzed by the complex [$NiBr_2$(**L3**)] (**P^P^{CD}** ligand), for path **a**, computed at the GFN2-xTB/ALPB(toluene) level of theory.

Structure	*E* (hartree)	*H* (hartree)	*G* (hartree)	Frequency (cm^{-1})
**Ethylene**	-6.27348526	-6.22125465	-6.24724294	
**L^3-Ia**	-333.33386935	-331.50233774	-331.74488268	
**L^3-IIa**	-339.61523976	-337.72590409	-337.97727222	
**L^3-IIIa**	-339.61654104	-337.72755663	-337.97937537	
**L^3-TSII-IVa**	-339.60315727	-337.71402149	-337.96424555	-340.61
**L^3-IVa**	-339.65105814	-337.75700713	-338.00545905	
**L^3-Va**	-339.66356450	-337.77327181	-338.02214124	
**L^3-TSV-VIa**	-339.65807929	-337.77088674	-338.01955673	-353.84
**L^3-VIa**	-339.65914279	-337.77058711	-338.01978423	
**L^3-VIIa**	-339.66168425	-337.77136605	-338.01864697	
**L^3-TSVII-VIIIa**	-339.65005344	-337.76212618	-338.01049068	-319.09
**L^3-VIIIa**	-339.66108520	-337.76970118	-338.01726184	
**L^3-IXa**	-339.67276792	-337.78215768	-338.02971109	
**L^3-TSIX-Xa**	-339.66505779	-337.77773647	-338.02587043	-328.56
**L^3-Xa**	-339.66611899	-337.77696081	-338.02530649	

Table S13. Electronic Energy (*E*). Enthalpy (*H*). and Gibbs free energy (*G*) in hartree for the stationary points of the ethylene insertion into the Ni-C1 bond catalyzed by the complex [$NiBr_2$(**L^3**)] (**P^P^{CD}** ligand), for path **b**, computed at the GFN2-xTB/ALPB(toluene) level of theory.

Structure	*E* (hartree)	*H* (hartree)	*G* (hartree)	Frequency (cm^{-1})
**Ethylene**	-6.27348526	-6.22125465	-6.24724294	
**L^3-Ib**	-333.32770910	-331.49673500	-331.73941386	
**L^3-IIb**	-339.61949078	-337.73147068	-337.98357866	
**L^3-IIIb**	-339.59950333	-337.70984444	-337.95846982	
**L^3-TSII-IVb**	-339.59930755	-337.71071862	-337.95790972	-119.49
**L^3-IVb**	-339.65958597	-337.76572306	-338.01178700	
**L^3-Vb**	-339.66997087	-337.77906623	-338.02591305	
**L^3-TSV-VIb**	-339.65770569	-337.77006388	-338.01642583	-54.92
**L^3-VIb**	-339.65770084	-337.76909592	-338.01703128	
**L^3-VIIb**	-339.66109701	-337.77288125	-338.02190136	
**L^3-TSVII-VIIIb**	-339.66102517	-337.77382753	-338.02183456	-72.29
**L^3-VIIIb**	-339.67467675	-337.78351538	-338.03169413	
**L^3-IXb**	-339.67130966	-337.77979646	-338.02666992	
**L^3-TSIX-Xb**	-339.66108095	-337.77322339	-338.01975670	-324.16
**L^3-Xb**	-339.66188244	-337.77300088	-338.02103541	

Table S14. Relative Electronic Energy (ΔE). Enthalpy (ΔH). and Gibbs free energy (ΔG) in kcal/mol for the stationary points of the ethylene insertion into the Ni-C1 bond catalyzed by the [$NiBr_2$(**L^5**)] complex (**P^P** ligand), for path **a**, computed at the GFN2-xTB/ALPB(toluene) level of theory.

Structure	ΔE (kcal/mol)	ΔH (kcal/mol)	ΔG (kcal/mol)
**L^5-Ia**	0.0	0.0	0.0
**L^5-IIa**	-12.4	-9.3	2.2
**L^5-IIIa**	-5.9	-1.4	11.5
**L^5-TSII-IVa**	-0.6	3.1	16.9
**L^5-IVa**	-30.3	-22.6	-8.9
**L^5-Va**	-37.3	-33.0	-19.8
**L^5-TSV-VIa**	-29.0	-26.2	-12.9
**L^5-VIa**	-29.4	-26.1	-14.0
**L^5-VIIa**	-33.4	-30.3	-17.8
**L^5-TSVII-VIIIa**	-32.1	-29.6	-16.6
**L^5-VIIIa**	-34.8	-29.9	-16.9
**L^5-IXa**	-36.6	-31.8	-18.8
**L^5-TSIX-Xa**	-31.7	-29.2	-15.8
**L^5-Xa**	-36.6	-33.7	-21.4

Table S15. Relative Electronic Energy (Δ*E*). Enthalpy (Δ*H*). and Gibbs free energy (Δ*G*) in kcal/mol for the stationary points of the ethylene insertion in the Ni-C1 bond catalyzed by the [$NiBr_2$(**L[5]**)] complex (**P^P** ligand), for path **b**, computed at the GFN2-xTB/ALPB(toluene) level of theory.

Structure	Δ*E* (kcal/mol)	Δ*H* (kcal/mol)	Δ*G* (kcal/mol)
**L^5-Ib**	0.0	0.0	0.0
**L^5-IIb**	-17.4	-14.4	-2.2
**L^5-IIIb**	-5.9	-1.3	11.0
**L^5-TSII-IVb**	0.1	3.6	16.8
**L^5-IVb**	-30.9	-24.3	-10.8
**L^5-Vb**	-37.6	-33.4	-20.3
**L^5-TSV-VIb**	-34.5	-32.3	-19.0
**L^5-VIb**	-36.3	-33.4	-20.9
**L^5-VIIb**	-36.8	-33.9	-21.0
**L^5-TSVII-VIIIb**	-35.6	-33.4	-20.0
**L^5-VIIIb**	-40.7	-36.3	-23.1
**L^5-IXb**	-40.9	-36.7	-23.7
**L^5-TSIX-Xb**	-35.7	-33.5	-20.2
**L^5-Xb**	-36.7	-33.9	-21.2

Table S16. Relative Electronic Energy (Δ*E*). Enthalpy (Δ*H*). and Gibbs free energy (Δ*G*) in kcal/mol for the stationary points of the ethylene insertion into the Ni-C1 bond catalyzed by the [$NiBr_2$(**L^3**)] complex (**$P^\wedge P^{CD}$** ligand), for path **b**, computed at the GFN2-xTB/ALPB(toluene) level of theory.

Structure	Δ*E* (kcal/mol)	Δ*H* (kcal/mol)	Δ*G* (kcal/mol)
**L^3-Ia**	0.0	0.0	0.0
**L^3-IIa**	-4.9	-1.5	9.3
**L^3-IIIa**	-5.8	-2.5	8.0
**L^3-TSII-IVa**	2.6	6.0	17.5
**L^3-IVa**	-27.4	-21.0	-8.4
**L^3-Va**	-35.3	-31.2	-18.8
**L^3-TSV-VIa**	-31.8	-29.7	-17.2
**L^3-VIa**	-32.5	-29.5	-17.4
**L^3-VIIa**	-34.1	-30.0	-16.6
**L^3-TSVII-VIIIa**	-26.8	-24.2	-11.5
**L^3-VIIIa**	-33.7	-28.9	-15.8
**L^3-IXa**	-41.0	-36.8	-23.6
**L^3-TSIX-Xa**	-36.2	-34.0	-21.2
**L^3-Xa**	-36.9	-33.5	-20.8

Table S17. Relative Electronic Energy (Δ*E*). Enthalpy (Δ*H*). and Gibbs free energy (Δ*G*) in kcal/mol for the stationary points of the ethylene insertion into the Ni-C1 bond catalyzed by the [NiBr$_2$(**L^3**)] complex (**P^P^{CD}** ligand), for path **b**, computed at the GFN2-xTB/ALPB(toluene) level of theory.

Structure	Δ*E* (kcal/mol)	Δ*H* (kcal/mol)	Δ*G* (kcal/mol)
**L^3-Ib**	0.0	0.0	0.0
**L^3-IIb**	-11.5	-8.5	1.9
**L^3-IIIb**	1.1	5.1	17.7
**L^3-TSII-IVb**	1.2	4.6	18.0
**L^3-IVb**	-36.6	-30.0	-15.8
**L^3-Vb**	-43.2	-38.3	-24.6
**L^3-TSV-VIb**	-35.5	-32.7	-18.7
**L^3-VIb**	-35.5	-32.1	-19.1
**L^3-VIIb**	-37.6	-34.4	-22.1
**L^3-TSVII-VIIIb**	-37.5	-35.0	-22.1
**L^3-VIIIb**	-46.1	-41.1	-28.3
**L^3-IXb**	-44.0	-38.8	-25.1
**L^3-TSIX-Xb**	-37.6	-34.7	-20.8
**L^3-Xb**	-38.1	-34.5	-21.6

Table S18. List of Δ*g*^{inter} scores for the Z-L, Ni-L, Ni-Bu interactions of the remarkable geometries for ethylene dimerization catalyzed with complex [NiBr$_2$(**L^5**)] (**P^P** ligand) and complex [NiBr$_2$(L^3)] (**P^P^{CD}** ligand). Data obtained from geometries optimized at the GFN2-xTB/ALPB(toluene) level of theory.

Structure	Δg^{inter}_{Bu-L}^{a} (a_0^{-1})	Δg^{inter}_{Ni-L}^{a} (a_0^{-1})	Δg^{inter}_{Ni-Bu}^{a} (a_0^{-1})	Δ$g^{inter}_{Ni-C2H4}$^{a} (a_0^{-1})	Δ$g^{inter}_{Et-C2H4}$^{a} (a_0^{-1})
**L^5-IIa**	0.845	1.888	2.169	0.737	0.209
**L^5-IIb**	0.907	1.932	2.131	0.711	0.181
**L^5-Va**	0.479	2.254	1.599		
**L^5-Vb**	0.469	2.266	1.615		
**L^5-VIIa**	0.802	2.183	1.699		
**L^5-VIIb**	0.845	2.220	1.680		
**L^3-IIa**	1.483	1.925	2.082	0.925	0.239
**L^3-IIb**	1.661	2.015	1.655	0.722	0.188
**L^3-Va**	1.299	2.344	1.687		
**L^3-Vb**	1.688	2.487	1.700		
**L^3-VIIa**	1.933	2.146	1.641		
**L^3-VIIb**	1.559	2.382	1.790		

^{a}Bu = substrates (ethyl + ethylene, butyl, or butene + H) coordinated to Ni; L = ligand (**P^P** or **P^P^{CD}**).

Table S19. List of Δg^{inter}(attractive), and Δg^{inter}(repulsive) scores for the Bu-L and Ni-L interaction of the remarkable geometries for ethylene dimerization catalyzed with complex [$NiBr_2$(**L^5**)] (**P^P** ligand) and complex [$NiBr_2$(**L^3**)] (**P^P^{CD}** ligand). Data obtained from geometries optimized at the GFN2-xTB/ALPB(toluene) level of theory.

	$\Delta g^{inter}_{Bu\text{-}L}$[a] ($a_0^{-1}$)		$\Delta g^{inter}_{Ni\text{-}Bu}$[a] ($a_0^{-1}$)		$\Delta g^{inter}_{Ni\text{-}C2H4}$[a] ($a_0^{-1}$)		$\Delta g^{inter}_{Et\text{-}C2H4}$[a] ($a_0^{-1}$)	
Structure	Δg^{inter}(attr.)	Δg^{inter}(rep.)	Δg^{inter}(attr.)	Δg^{inter}(rep.)	Δg^{inter}(attr.)	Δg^{inter}(rep.)	Δg^{inter}(attr.)	Δg^{inter}(rep.)
**L^5-IIa**	0.452	0.393	1.410	0.759	0.487	0.250	0.127	0.082
**L^5-IIb**	0.488	0.419	1.376	0.755	0.461	0.250	0.107	0.075
**L^5-Va**	0.265	0.214	1.026	0.573				
**L^5-Vb**	0.255	0.214	1.037	0.578				
**L^5-VIIa**	0.439	0.363	1.167	0.532				
**L^5-VIIb**	0.426	0.420	1.170	0.510				
**L^3-IIa**	0.780	0.703	1.375	0.707	0.619	0.307	0.150	0.089
**L^3-IIb**	0.879	0.782	1.069	0.586	0.466	0.256	0.113	0.075
**L^3-Va**	0.684	0.615	1.081	0.606				
**L^3-Vb**	0.889	0.799	1.108	0.592				
**L^3-VIIa**	1.047	0.886	1.146	0.495				
**L^3-VIIb**	0.803	0.756	1.252	0.538				

[a]Bu = substrates (ethyl + ethylene, butyl, or butene + H) coordinated to Ni; L = ligand (**P^P** or **P^P^{CD}**).

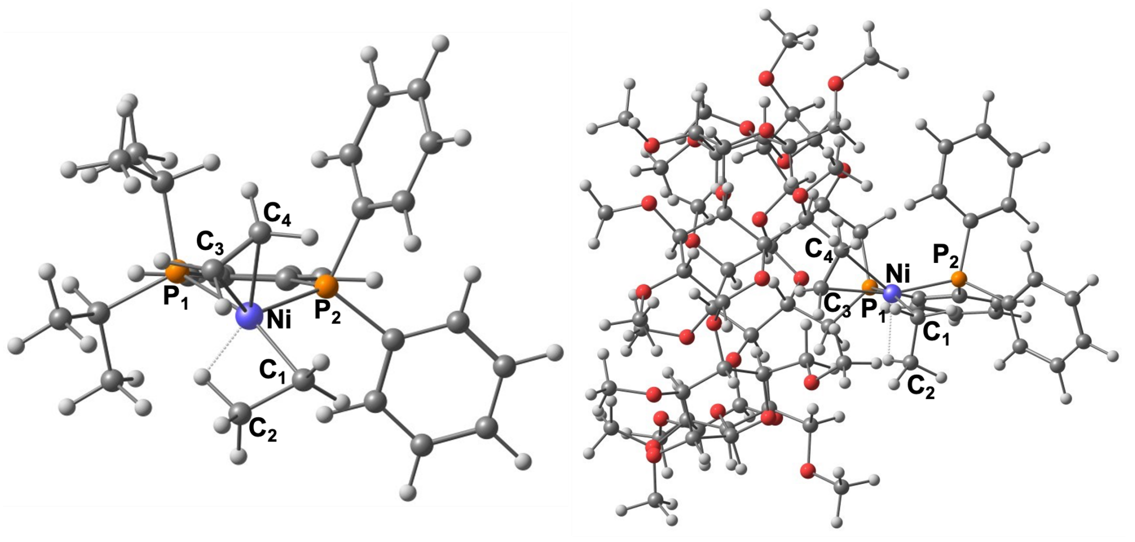

L^5-IIa **L^3-IIa**

Figure S125. Optimized geometry of **L^5-IIa** (left) and **L^3-IIa** (right) computed at the GFN2-xTB/ALPB(toluene) level of theory.

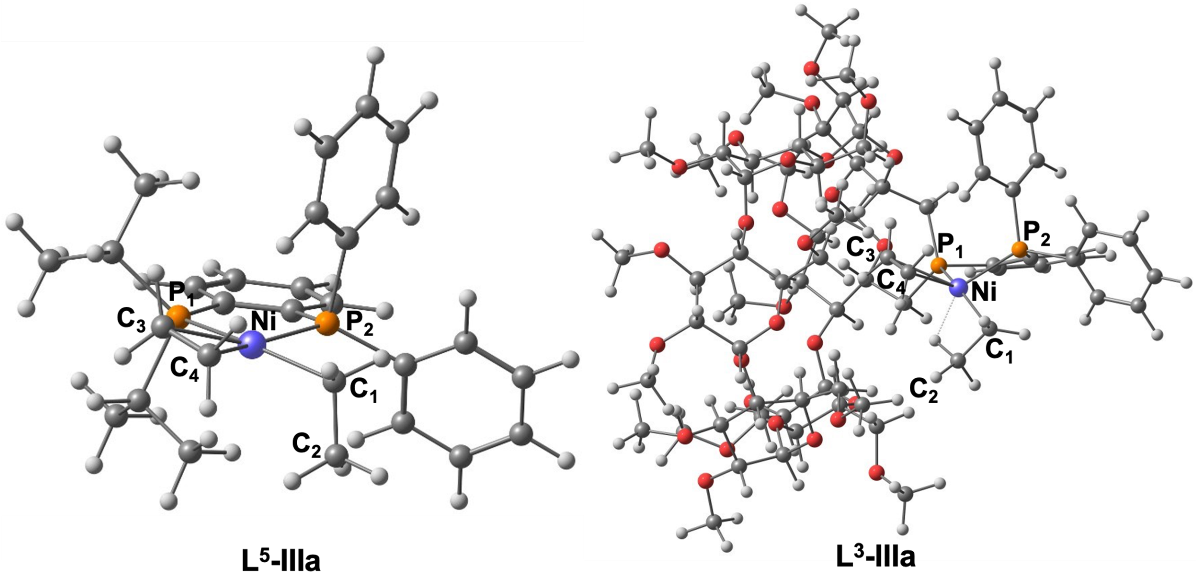

L^5-IIIa **L^3-IIIa**

Figure S126. Optimized geometry of **L^5-IIIa** (left) and **L^3-IIIa** (right) computed at the GFN2-xTB/ALPB(toluene) level of theory.

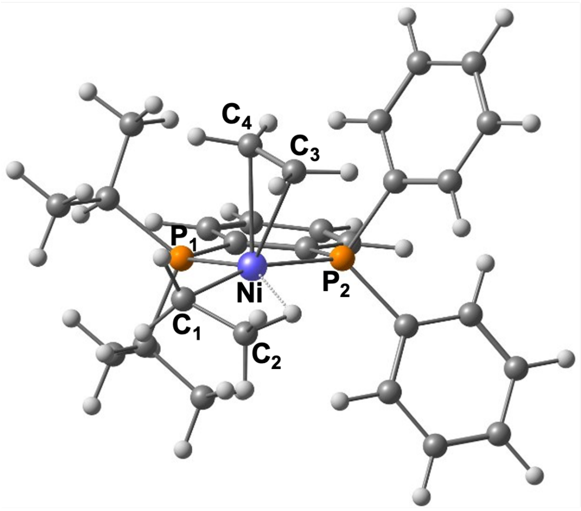

L⁵-IIb

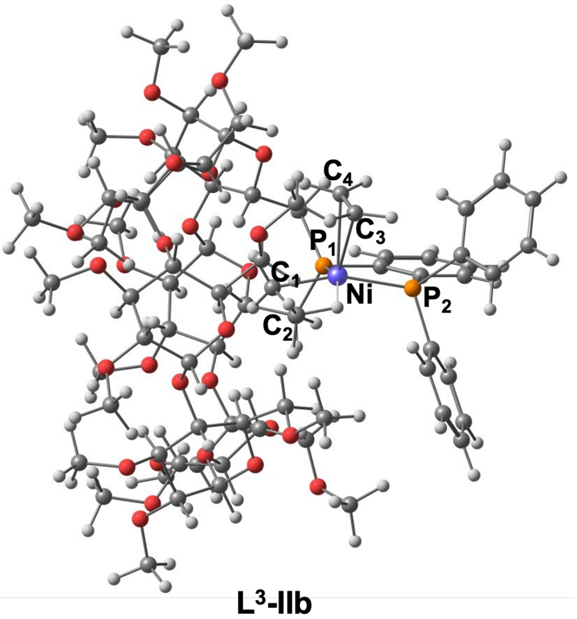

L³-IIb

Figure S127. Optimized geometry of **L⁵-IIb** (left) and **L³-IIb** (right) computed at the GFN2-xTB/ALPB(toluene) level of theory.

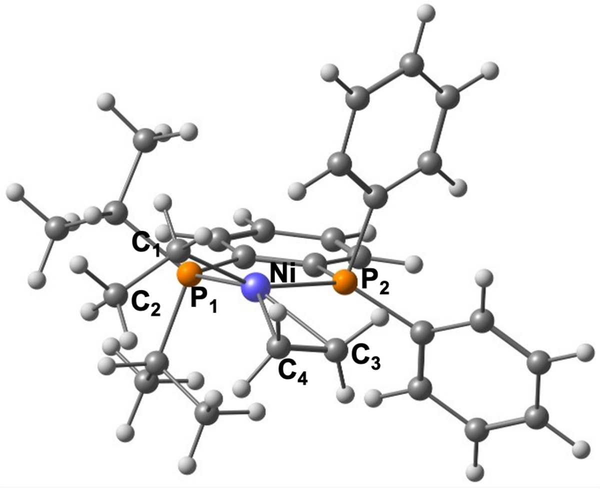

L⁵-IIIb

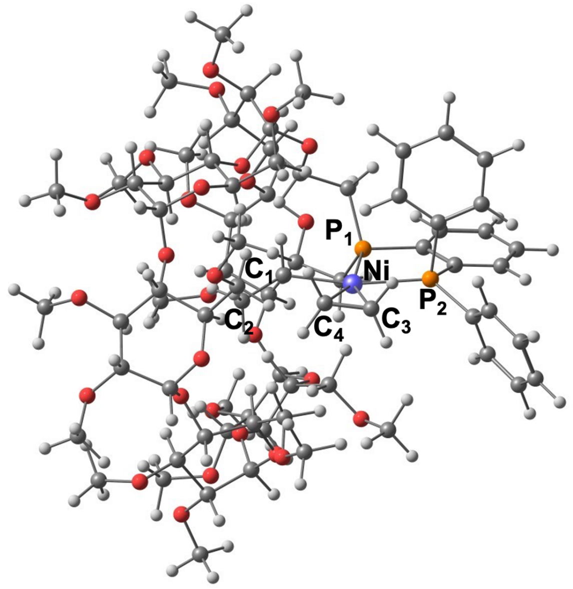

L³-IIIb

Figure S128. Optimized geometry of **L⁵-IIIb** (left) and **L³-IIIa** (right) computed at the GFN2-xTB/ALPB(toluene) level of theory.

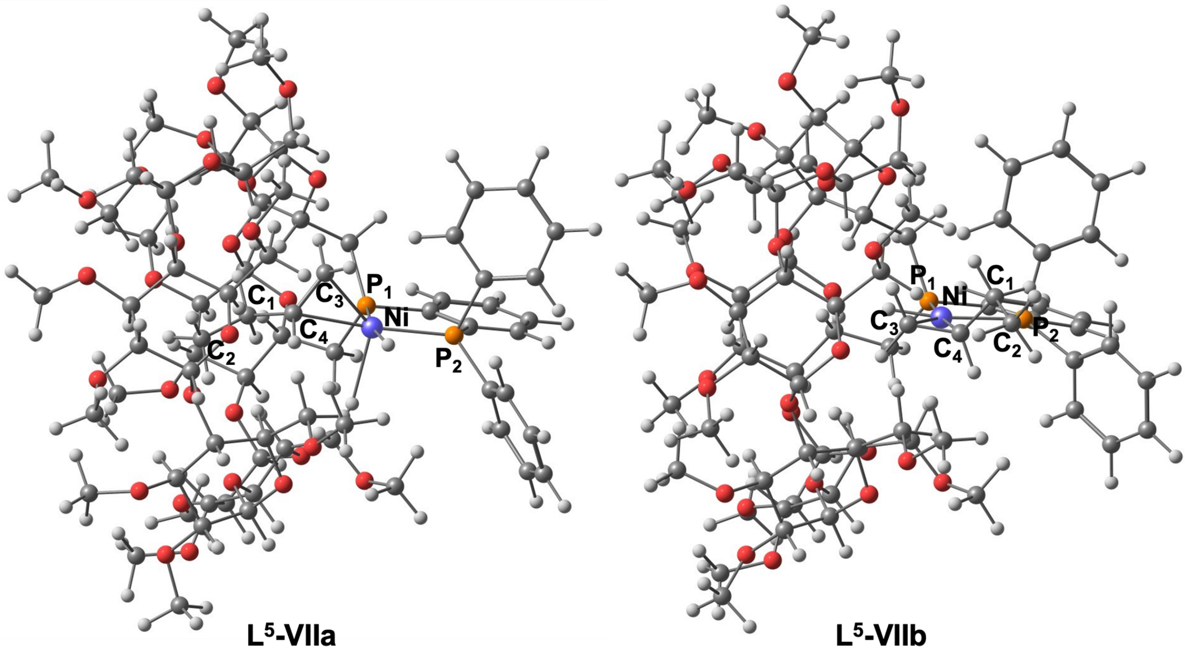

L^5-VIIa L^5-VIIb

Figure S129. Optimized geometry of **L^3-VIIa** (left) and **L^3-VIIb** (right) computed at the GFN2-xTB/ALPB(toluene) level of theory.

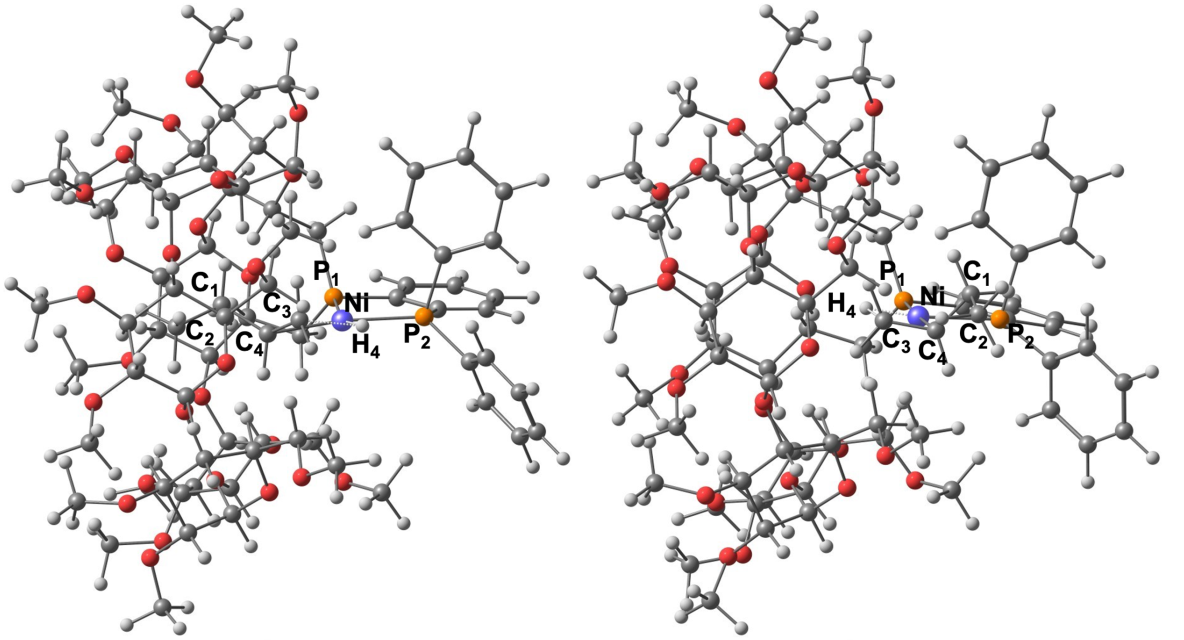

L^5-TSVII-VIIIa L^5-TSVII-VIIIb

Figure S130. Optimized geometry of **L^3-TSVII-VIIIa** (left) and **L^3-TSVII-VIIIb** (right) computed at the GFN2-xTB/ALPB(toluene) level of theory.

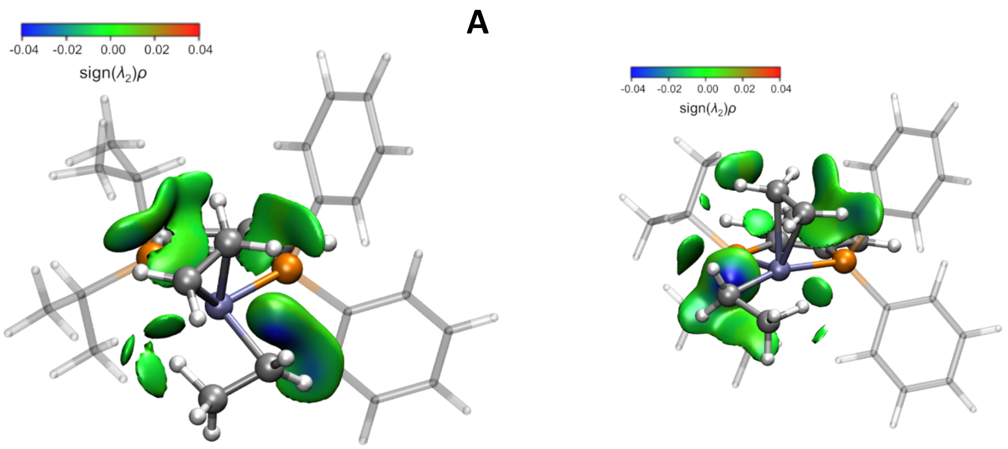

Figure S131. $\delta g^{\mathrm{inter}}$ isosurface plot (cutoff: $\delta g^{\mathrm{inter}} = 0.005\ a_0^{-4}$ and BGR color scale in the range $0.04 < \mathrm{sign}(\lambda_2)\rho < 0.04\ a_0^{-3}$) of the interaction between Bu and **P^P** ligand in **L^5-IIa** (A) and **L^5-IIb** (B). Electron density was computed at the PBE-D3(BJ)/def2-TZVP/CPCM(toluene) level of theory from geometry optimized at the GFN2-xTB/ALPB(toluene) level of theory. Surface color code: blue = attractive interaction, green = nonbonding interactions, red = repulsive interactions. Atom color code: hydrogen (white), carbon (silver), phosphorus (orange), and nickel (ice blue).

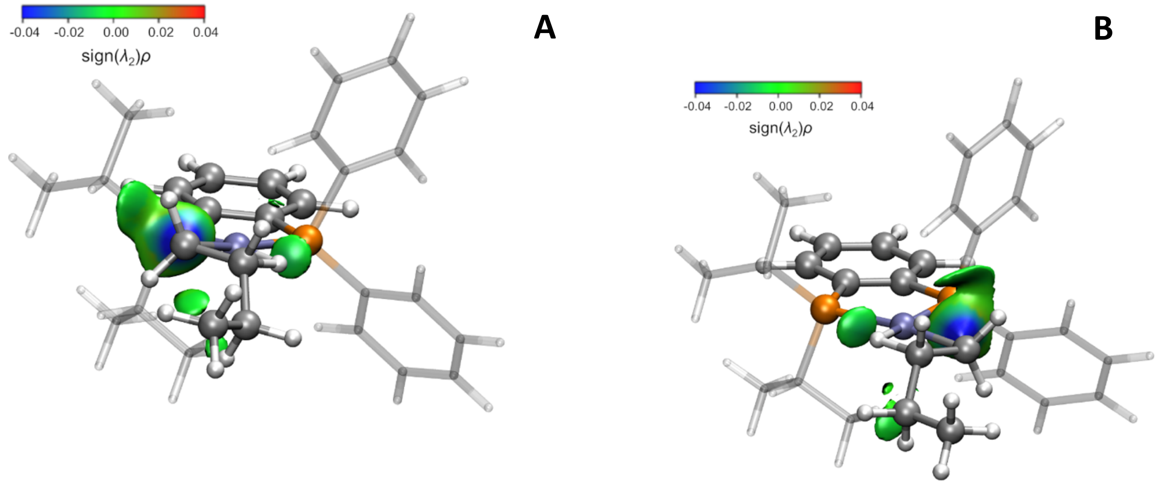

Figure S132. $\delta g^{\mathrm{inter}}$ isosurface plot (cutoff: $\delta g^{\mathrm{inter}} = 0.005\ a_0^{-4}$ and BGR color scale in the range $0.04 < \mathrm{sign}(\lambda_2)\rho < 0.04\ a_0^{-3}$) of the interaction between Bu and **P^P** ligand in **L^5-Va** (A) and **L^5-Vb** (B). Electron density was computed at the PBE-D3(BJ)/def2-TZVP/CPCM(toluene) level of theory from geometry optimized at the GFN2-xTB/ALPB(toluene) level of theory. Surface color code: blue = attractive interaction, green = nonbonding interactions, red = repulsive interactions. Atom color code: hydrogen (white), carbon (silver), phosphorus (orange), and nickel (ice blue).

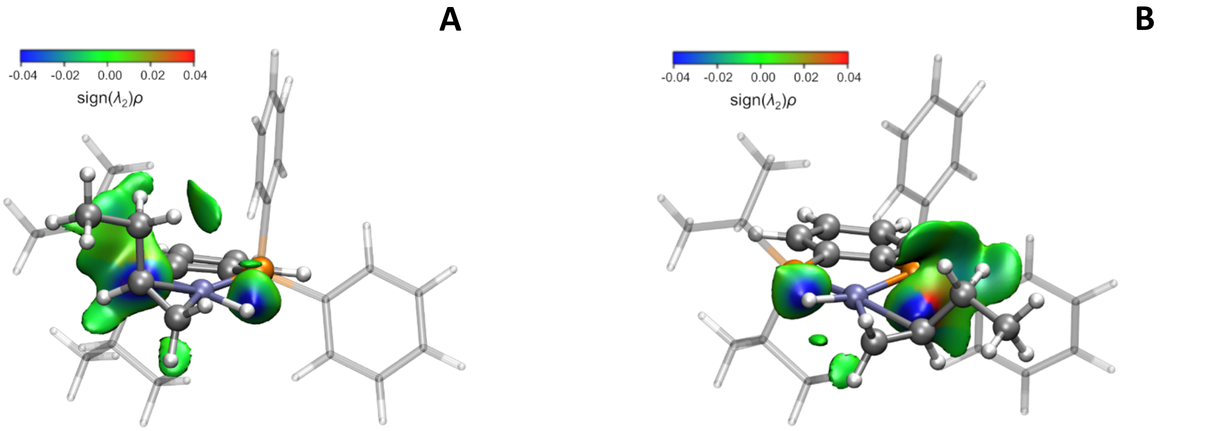

Figure S133. δg^{inter} isosurface plot (cutoff: δg^{inter}= 0.005 a_0^{-4} and BGR color scale in the range 0.04 < sign(λ_2)ρ < 0.04 a_0^{-3}) of the interaction between Bu and **P^P** ligand in **L⁵-VIIa** (A) and **L⁵-VIIb** (A). Electron density was computed at the PBE-D3(BJ)/def2-TZVP/CPCM(toluene) level of theory from geometry optimized at the GFN2-xTB/ALPB(toluene) level of theory. Surface color code: blue = attractive interaction, green = nonbonding interactions, red = repulsive interactions. Atom color code: hydrogen (white), carbon (silver), phosphorus (orange), and nickel (ice blue).

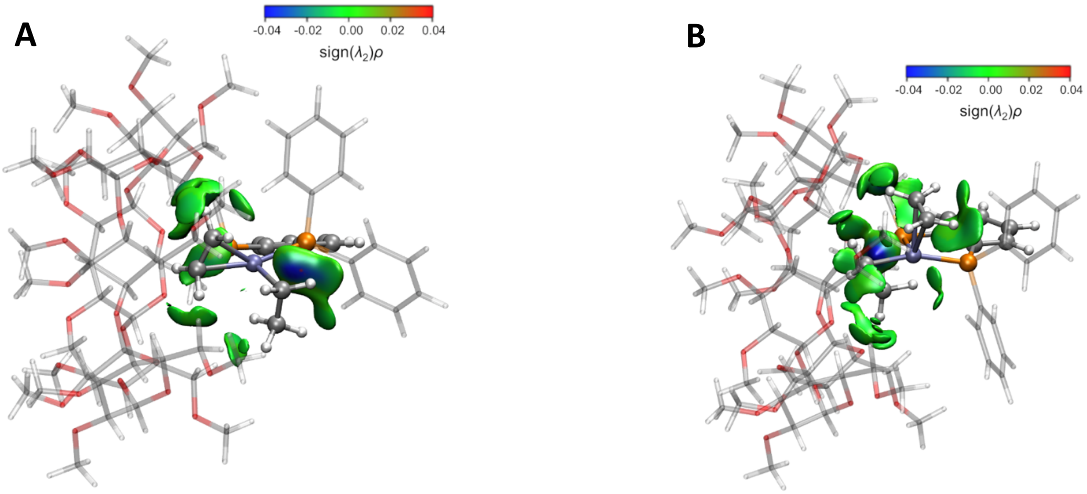

Figure S134. δg^{inter} isosurface plot (cutoff: δg^{inter} = 0.005 a_0^{-4} and BGR color scale in the range 0.04 < sign(λ_2)ρ < 0.04 a_0^{-3}) of the interaction between Bu and **P^P^{CD}** ligand in **L³-IIa** (A) and **L³-IIb** (B). Electron density was computed at the PBE-D3(BJ)/def2-TZVP/CPCM(toluene) level of theory from geometry optimized at the GFN2-xTB/ALPB(toluene) level of theory. Surface color code: blue = attractive interaction, green = nonbonding interactions, red = repulsive interactions. Atom color code: hydrogen (white), carbon (silver), phosphorus (orange), and nickel (ice blue).

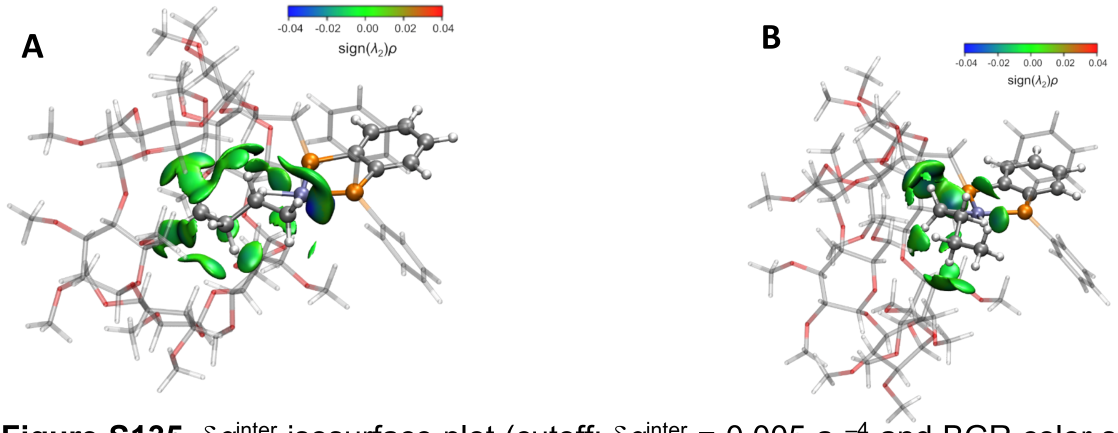

Figure S135. δg^{inter} isosurface plot (cutoff: δg^{inter} = 0.005 a_0^{-4} and BGR color scale in the range 0.04 < sign(λ_2)ρ < 0.04 a_0^{-3}) of the interaction between Bu and **P^P^{CD}** ligand in **L^3-Va** (A) and **L^3-Vb** (B). Electron density was computed at the PBE-D3(BJ)/def2-TZVP/CPCM(toluene) level of theory from geometry optimized at the GFN2-xTB/ALPB(toluene) level of theory. Surface color code: blue = attractive interaction, green = nonbonding interactions, red = repulsive interactions. Atom color code: hydrogen (white), carbon (silver), phosphorus (orange), and nickel (ice blue).

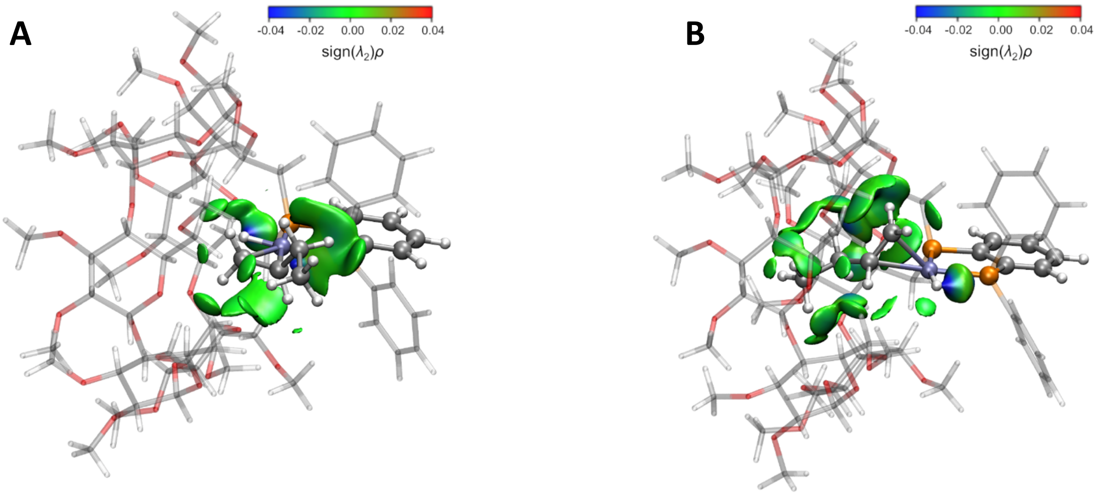

Figure S136. δg^{inter} isosurface plot (cutoff: δg^{inter} = 0.005 a_0^{-4} and BGR color scale in the range 0.04 < sign(λ_2)ρ < 0.04 a_0^{-3}) of the interaction between Bu and **P^P^{CD}** ligand in **L^3-VIIa** (A) and **L^3-VIIb** (A). Electron density was computed at the PBE-D3(BJ)/def2-TZVP/CPCM(toluene) level of theory from geometry optimized at the GFN2-xTB/ALPB(toluene) level of theory. Surface color code: blue = attractive interaction, green = nonbonding interactions, red = repulsive interactions. Atom color code: hydrogen (white), carbon (silver), phosphorus (orange), and nickel (ice blue).

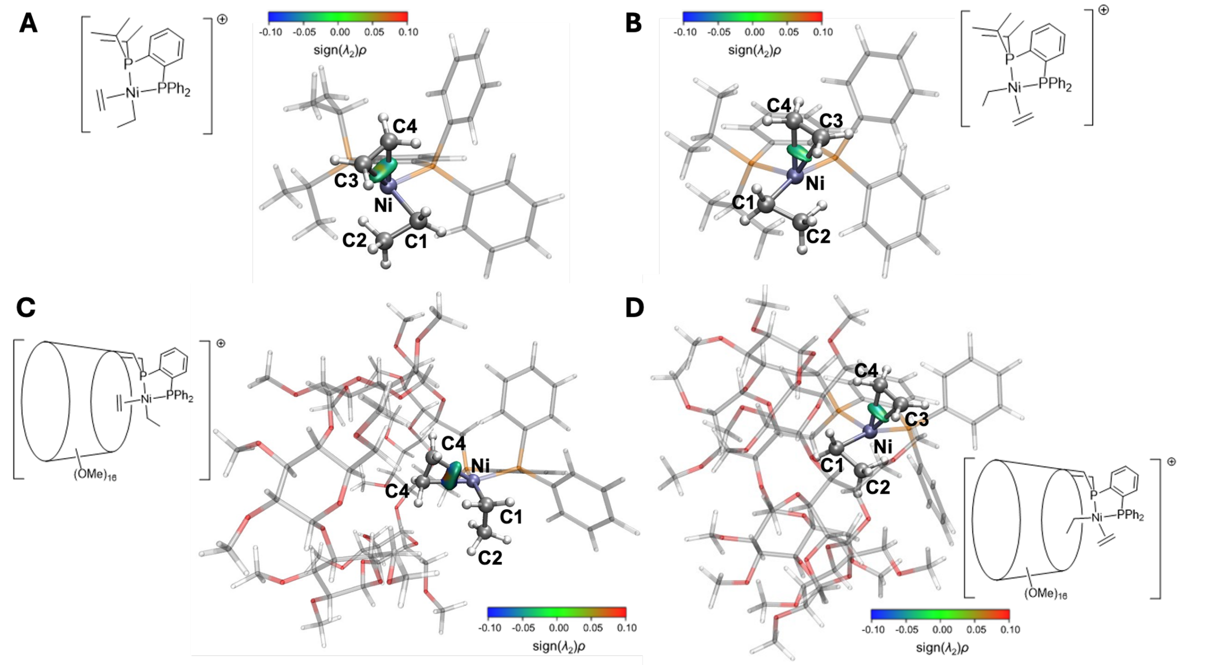

Figure S137. $\delta g^{\mathrm{inter}}$ isosurface plot (cutoff: $\delta g^{\mathrm{inter}} = 0.04\ a_0^{-4}$ and Blue Green Red color scale in the range $0.1 < \mathrm{sign}(\lambda_2)\rho < 0.1\ a_0^{-3}$) displaying the interaction between Ni and C_2H_4 for (A) **L^5-IIa**, (B) **L^5-IIb**, (C) **L^3-IIa**, and (D) **L^3-IIb**. Electron density was computed at the PBE-D3(BJ)/def2-TZVP/CPCM(toluene) level of theory from geometry optimized at the GFN2-xTB/ALPB(toluene) level of theory. Surface color code: blue = attractive interaction, green = nonbonding interactions, red = repulsive interactions. Atom color code: hydrogen (white), carbon (silver), phosphorus (orange), and nickel (ice blue).

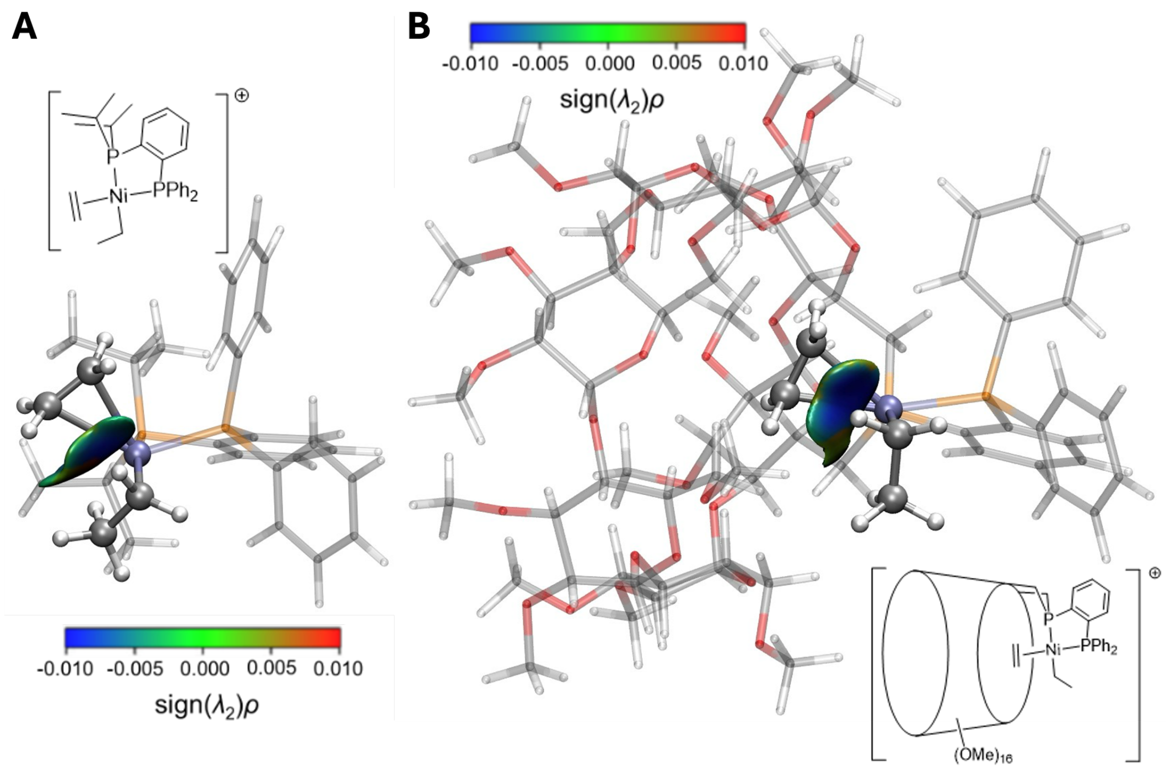

Figure S138. δg^{inter} isosurface plot (cutoff: δg^{inter} = 0.005 a_0^{-4} and Blue Green Red color scale in the range 0.01 < sign(λ_2)ρ < 0.01 a_0^{-3}) displaying the interaction between C_2H_4 and ethyl ligand for (A) **L[5]-IIa**, and (B) **L[3]-IIa**. Electron density was computed at the PBE-D3(BJ)/def2-TZVP/CPCM(toluene) level of theory from geometry optimized at the GFN2-xTB/ALPB(toluene) level of theory. Surface color code: blue = attractive interaction, green = nonbonding interactions, red = repulsive interactions. Atom color code: hydrogen (white), carbon (silver), phosphorus (orange), and nickel (iceblue).

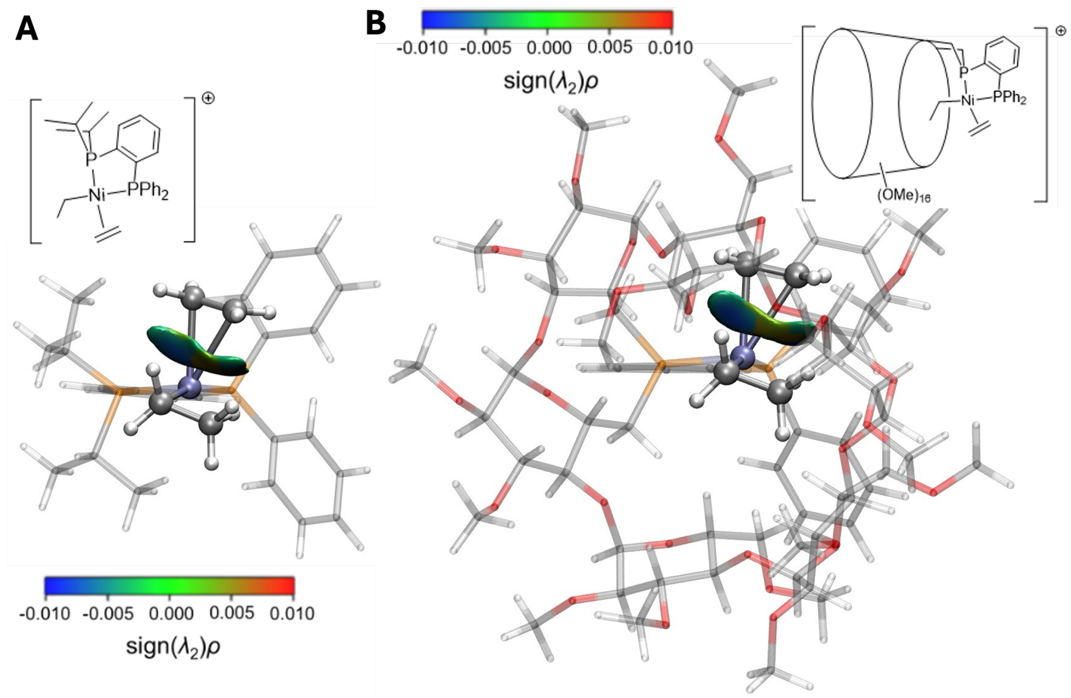

Figure S139. δg^{inter} isosurface plot (cutoff: δg^{inter} = 0.005 a_0^{-4} and Blue Green Red color scale in the range 0.01 < sign(λ_2)ρ < 0.01 a_0^{-3}) displaying the interaction between C_2H_4 and ethyl ligand for (A) **L⁵-IIb**, and (B) **L³-IIb**. Electron density was computed at the PBE-D3(BJ)/def2-TZVP/CPCM(toluene) level of theory from geometry optimized at the GFN2-xTB/ALPB(toluene) level of theory. Surface color code: blue = attractive interaction, green = nonbonding interactions, red = repulsive interactions. Atom color code: hydrogen (white), carbon (silver), phosphorus (orange), and nickel (ice blue).

References

[1] a) E. Engeldinger, L. Poorters, D. Armspach, D. Matt, L. Toupet, *Chem. Commun.* **2004**, 634-635; b) D. Armspach, L. Poorters, D. Matt, B. Benmerad, F. Balegroune, L. Toupet, *Org. Biomol. Chem.* **2005**, *3*, 2588-2592.

[2] T. A. Phan, N. Armaroli, A. S. Moncada, E. Bandini, B. Delavaux-Nicot, J. F. Nierengarten, D. Armspach, *Angew. Chem. Int. Ed.* **2023**, *62,* e2022146.

[3] T. A. Phan, M. Jouffroy, D. Matt, N. Armaroli, A. S. Moncada, E. Bandini, B. Delavaux-Nicot, J. F. Nierengarten, D. Armspach, *Chem. Eur. J.* **2024**, *30*, e202302750.

[4] Y. M. Li, S. Chakrabarty, C. Mück-Lichtenfeld, A. Studer, *Angew. Chem. Int. Ed.* **2016**, *55*, 802-806.

[5] Y. Li, K. Pelzer, D. Sechet, G. Creste, D. Matt, P. Braunstein, D. Armspach, *Dalton Trans.* **2022**, *51*, 11226-11230.

[6] G. M. Sheldrick, *Acta Crystallogr. A* **2008**, *A64*, 112-122.

[7] A. L. Spek, *Acta Crystallogr.* **2009**, *D65*, 148-155.

[8] F. Neese, *WIREs Comput. Mol. Sci.* **2022**, *12*, e1606.

[9] J. P. Perdew, K. Burke, M. Ernzerhof, *Phys. Rev. Lett.* **1996**, *77*, 3865-3868.

[10] (a) S. Grimme, S. Ehrlich, L. Goerigk, *J. Comput. Chem.* **2011**, *32*, 1456-1465; (b) S. Grimme, J. Antony, S. Ehrlich, H. Krieg, *J. Chem. Phys.* **2010**, *132*, 154104.

[11] F. Weigend, R. Ahlrichs, *Phys. Chem. Chem. Phys.* **2005**, *7*, 3297-3305.

[12] F. Weigend, *Phys. Chem. Chem. Phys.* **2006**, *8*, 1057-1065.

[13] V. Barone, M. Cossi, *J. Phys. Chem. A.* **1998**, *102*, 1995-2001.

[14] C. Bannwarth, S. Ehlert, S. Grimme, *J. Chem. Theory Comput.* **2019**, *15*, 1652-1671.

[15] S. Ehlert, M. Stahn, S. Spicher, S. Grimme, *J. Chem. Theory Comput.* **2021**, *17*, 4250-4261.

[16] G. A. Zhurko, Chemcraft, Version 1.8, https://www.chemcraftprog.com.

[17] (a) C. Lefebvre, G. Rubez, H. Khartabil, J. C. Boisson, J. Contreras-García, E. Hénon, *Phys. Chem. Chem. Phys.* **2017**, *19*, 17928-17936; (b) C. Lefebvre, J. Klein, H. Khartabil, J.-C. Boisson, E. Hénon, *J. Comput. Chem.* **2023**, 44, 1750-1766.

[18] W. Humphrey, A. Dalke, K. Schulten, *J. Mol. Graph.* **1996**, *14*, 33-38.

[19] C. Bannwarth, S. Ehlert, S. Grimme, *J. Chem. Theory Comput.* **2019**, *15*, 1652-1671.

[20] M. Bursch, A. Hansen, S. Grimme, *Inorg. Chem.* **2017**, *56*, 12485-12491.

[21] D. A. C. Ferreira, S. F. D. Morais, S. M. P. Meneghetti, M. R. Meneghetti, *J. Mol. Catal. A Chem.* **2012**, *363*, 1-9.

[22] T. K. Woo, T. Ziegler, *J. Organomet. Chem.* **1999**, *591*, 204-213.
